# Vitamin D receptor suppresses proliferation and metastasis in renal cell carcinoma cell lines via regulating the expression of the epithelial Ca^2+^ channel TRPV5

**DOI:** 10.1371/journal.pone.0195844

**Published:** 2018-04-16

**Authors:** YongMing Chen, XinYu Liu, FaBiao Zhang, ShanFan Liao, XiYuan He, DeXiang Zhuo, HuaiBin Huang, YongYang Wu

**Affiliations:** 1 Department of Urology, Affiliated Sanming First Hospital, Fujian Medical University, Sanming, Fujian, China; 2 State Key Laboratory of Biotherapy and Cancer Center, West China Hospital, Sichuan University and Collaborative Innovation Center for Biotherapy, Chengdu, Sichuan, China; University of South Alabama Mitchell Cancer Institute, UNITED STATES

## Abstract

We previously demonstrated that transient receptor potential vanilloid subfamily 5 (TRPV5) expression was decreased in renal cell carcinoma (RCC) compared with that in normal kidney tissues, a finding that was correlated with vitamin D receptor (VDR) expression, but further investigations is warranted. The aim of this study was to elucidate whether VDR could regulate the expression of TRPV5 and affect proliferation and metastasis in RCC. In this study, we used lentivirus to conduct the model of VDR overexpression and knockdown caki-1 and 786-O RCC cell lines *in vitro*. The results demonstrated that VDR overexpression significantly inhibited RCC cells proliferation, migration and invasion, and promoted apoptosis by the MTT, transwell cell migration/invasion and flow cytometry assays, respectively. However, VDR knockdown in RCC cells had the opposite effect. The RNA-sequence assay, which was assessed in caki-1 cells after VDR overexpression and knockdown, also indicated that significantly differentially expressed genes were associated with cell apoptotic, differentiation, proliferation and migration. RT-PCR and western blot analysis showed that VDR knockdown increased TRPV5 expression and VDR overexpression decreased TRPV5 expression in caki-1 cells. Furthermore, knockdown of TRPV5 expression suppressed the VDR knockdown-induced change in the proliferation, migration and invasion in caki-1 cells. Taken together, these findings confirmed that VDR functions as a tumour suppressor in RCC cells and suppresses the proliferation, migration and invasion of RCC through regulating the expression of TRPV5.

## Introduction

Renal cell carcinoma (RCC) is the most common form of kidney cancer [1] with an estimated 65,340 new cases diagnosed and 14,970 deaths in 2017 in the United States [2]. Despite significant advances in RCC treatment, the prognosis remains poor, and the mortality rate remains high [3]. Consequently, it is urgently needed to study the molecular mechanisms of proliferation and metastasis in RCC, which may provide new treatment methods.

Recently, several lines of evidence showed that 1,25-dihydroxyvitamin D (1,25(OH_)2_D), whose action is mediated through binding to the Vitamin D receptor (VDR) and is the active metabolite of vitamin D, could reduce the risk of RCC [4–7]. On the other hand, VDR expressed in the normal kidney was decreased during malignant transformation to RCC and was associated with RCC prognosis [8–10]. These findings confirmed that VDR may play a key role in RCC development and progression.

The transient receptor potential vanilloid subfamily 5 (TRPV5) is a new type of highly selective calcium channel proteins that is mainly found in the kidney, is related to calcium transport, and plays an important role in maintaining the stability of the intracellular calcium concentration [11, 12]. TRPV5 was detected in human carcinoma of the colon, parathyroid glands, lung, and kidney [13–16]. Previous studies in animal and cell models revealed that TRPV5 transcription is tightly regulated by and correlated with 1,25(OH)_2_D and VDR, Moreover, the human TRPV5 promoter contains several consensus Vitamin D-responsive elements [17–19]. In a previous study we also found that TRPV5 expression was correlated with VDR [16]. This evidence likely suggests that VDR is probably involved in the development and progression of RCC via regulating the transcription of TRPV5.

In this study, we first analysed the expression level of VDR in the RCC cell lines caki-1 and 786-O cells. Nest, we used lentivirus to conduct the model of VDR overexpression and knockdown RCC cell lines *in vitro*. Several functional experiments confirmed that VDR overexpression decreased cell proliferation, migration, invasion and promoted apoptosis in RCC cells, whereas knockdown of VDR expression led to a completely opposite effect. Next, we found that the TRPV5 expression level was negatively correlated with VDR, whose overexpression down-regulated TRPV5 expression and knockdown up-regulated TRPV5 expression in caki-1 cells. Finally, we further demonstrated that knockdown of TRPV5 suppressed proliferation, migration and invasion induced by VDR knockdown in caki-1 cells. Taken together, our data demonstrated that VDR could act as a tumour suppressor to suppress proliferation, migration and invasion in RCC cell lines. Furthermore, VDR could regulate the expression of TRPV5, and the knockdown of TRPV5 could reverse the carcinogenesis induced by VDR knockdown in RCC cell lines. These findings might indicate a novel target for the biological treatment of RCC.

## Materials and methods

### Cell culture

The human RCC cell lines caki-1 and 786-O were purchased from the Type Culture Collection of the Chinese Academy of Sciences (Shanghai, China).Two cell lines were routinely cultured in DMEM (HyClone, Shanghai, China) and RPMI-1640 (HyClone, Shanghai, China), respectively, containing 10% FBS (Natocor, Córdoba, Argentina) supplemented with 1% penicillin/streptomycin. Cells were grown at 37°C in a humidified incubator containing 5% CO_2_ (Thermo Scientific Forma).

### Lentivirus vector production and cell infection

Lentivirus carrying the VDR gene (leVDR) was generated in pLent-EF1a-FH-CMV-GP vector, and the empty vector served as the control (leCtrl). Short hairpin RNAs targeting VDR or TRPV5 were generated in pLent-U6-GFP-Puro vectors, and empty pLent-U6-GFP-Puro vectors were used as controls. All lentiviruses were packaged by Vigene Biosciences Company, Ltd. (Shandong, China). The Caki-1 and 786-O cells were infected with the lentivirus at an MOI of 50 and 20, respectively. At 72 h post-infection, the cells were observed for the presence of the GFP marker suing a fluorescence microscope. Nest, puromycin was added to screen the stable cell lines, and the screening process was contined for a week with a screening concentration of 3 μg/ml. Stable VDR-overexpression, VDR knockdown and/or TRPV5 knockdown RCC cell lines were successfully constructed.

### Quantitative real-time RT-PCR

Total RNA was extracted from cells using TRIzol reagent (Invitrogen Life Technologies, Carlsbad, CA, USA) according to the manufacturer’ s protocol and then was reverse transcribed using the 5× All-in-one RT MasterMix Kit (Abm), real-time RT-PCR was performed using SYBR® Green-I as the fluorogenic dye. Nest, 3 μL of cDNA was added to a 20 μL reaction system of EvaGreen 2× qPCR Mastermix version with 0.6μM of each pair of gene specific primers. mRNA expression was normalized as the ratio to that of β-actin in each sample. The RT-PCR primers used were as follows: 5’-TGGAGACTTTGACCGGAACG-3’; VDR reverse: 5’-GGGCAGGTGAATAGTGCCTT-3’; TRPV5 forward: 5’-TGGCACTGTTCACCACCTTT-3’; TRPV5 reverse: 5’-CAATGATGGCGAAGGCGAAG-3’; β-actin forward: 5’-CAGGGCGTGATGGTGGGCA-3’; β-actin reverse: 5’-CAAACATCATCTGGGTCATCTTCTC-3’.

### Western blot analysis

The cells were harvested and lysed in RIPA buffer (Sigma-Vetec, St. Louis, MO, USA) supplemented with a 1% phenylmethylsulfonyl fluoride (PMSF) on ice, and then they were centrifuged at 12000 × g for 15 minutes at 4°C, followed by collection of the supernatants. Next, the protein concentrations were determined using the BCA protein assay kit (Thermo Fisher Scientific, USA). The proteins were blended with loading buffer and boiling water for 5 minutes. Equal amounts of protein from each sample were fractionated using 10% SDS-PAGE gels and were electrotransferred onto polyvinylidene difluoride (PVDF) membranes. The membranes were blocked for 2 hours with 5% skim milk in TBST buffer at room temperature and were incubated overnight with the relevant primary antibodies at 4°C, washing and incubation for 90 minutes with secondary antibodies at 37°C. Finally, the membranes were incubated with enhanced chemiluminescence (ECL) reagent, and the proteins were detected using the chemiluminescence imaging system (Shanghai, China). The expression of β-actin was used as the control. Anti-VDR was purchased from Thermo Scientific (Rockford, USA). Anti-TRPV5 was purchased from Santa Cruz Biotechnology (CA, USA). Anti-β-Actin, goat anti-mouse secondary antibodies (511103) and goat anti-rabbit secondary antibodies (511203) were purchased from Zen Bioscience (Chengdu, China). Goat anti-rat antibodies were purchased from Biosharp (Chengdu, China).

### Cell proliferation assay

Cells were seeded at a concentration of 2,000 or 3,000 cells/well in 96-well plates and then were incubated at 37°C in a 5% CO_2_ humidified atmosphere. At the indicated time points, the cells were incubated with thiazolyl blue tetrazolium bromide (MTT; 5 mg/ml; Sigma-Aldrich Co, St. Louis, MO, USA) at a final concentration of 0.5 mg/ml for 4 h. After discarding the supernatants, 150 μL of dimethyl sulfoxide (DMSO; Sigma-Aldrich Co, St. Louis, MO, USA) was added to each well. The plates were read at 570 nm using an ELISA reader (Bio-Rad). All experiments were performed in triplicate.

### Cell apoptosis assay

Cell apoptosis analysis was performed by flow cytometry using an Annexin V-FITC/PI apoptosis detection kit (keyGEN BioTECH). Cells were seeded in 6-well plates; after 72 h post-infection, the cells were harvested and washed with ice-cold PBS and then were blended with 500 μL of binding buffer. The cell suspensions were thoroughly mixed with 5 μL of Annexin V-FITC and 5 μL of PI in the dark at room temperature for 10 min, and all samples were subjected to FCM within 1 h. All experiments were performed in triplicate.

### Transwell assay

Cells were serum starved for 6 h. For the cell migration assay, 30,000 or 70,000 cells in 200 μL of serum-free media were seeded into the upper chamber of a transwell with polycarbonate membranes (8.0-μm pore size; Corning Incorporated, ME, USA), The lower chamber contained 500 μL of medium supplemented with 10% FBS. For the cell invasive assay, the upper chamber of the transwell was coated with Matrigel (Corning, Bedford, MA, USA), similar to that described above. At the indicated time points, the non-migrated cells on the upper surface were removed by wiping with a cotton swab, and the migrated cells on the lower surface were fixed with 4% paraformaldehyde for 30 min, stained with crystal violet solution for 20 min, and then were rinsed in PBS. The number of cells was counted in 5 randomly chosen fields (magnification, ×200). All experiments were performed in triplicate.

### Transcriptome sequencing analysis

The VDR overexpression and knockdown of caki-1 cells were used for experiments. Total RNA was extracted using TRIzol reagent, Transcriptome sequencing and data analysis by Chengdu Basebio Company completed. P<0.05 was considered statistically significant.

### Statistical analyses

Statistical analyses were performed using GraphPad Prism 6 and SPSS 22, all the data were presented as means ± SD. P<0.05 was considered statistically significant.

## Results

### Lentiviral-mediated knockdown and overexpression of VDR in RCC cells

**Fig 1:**
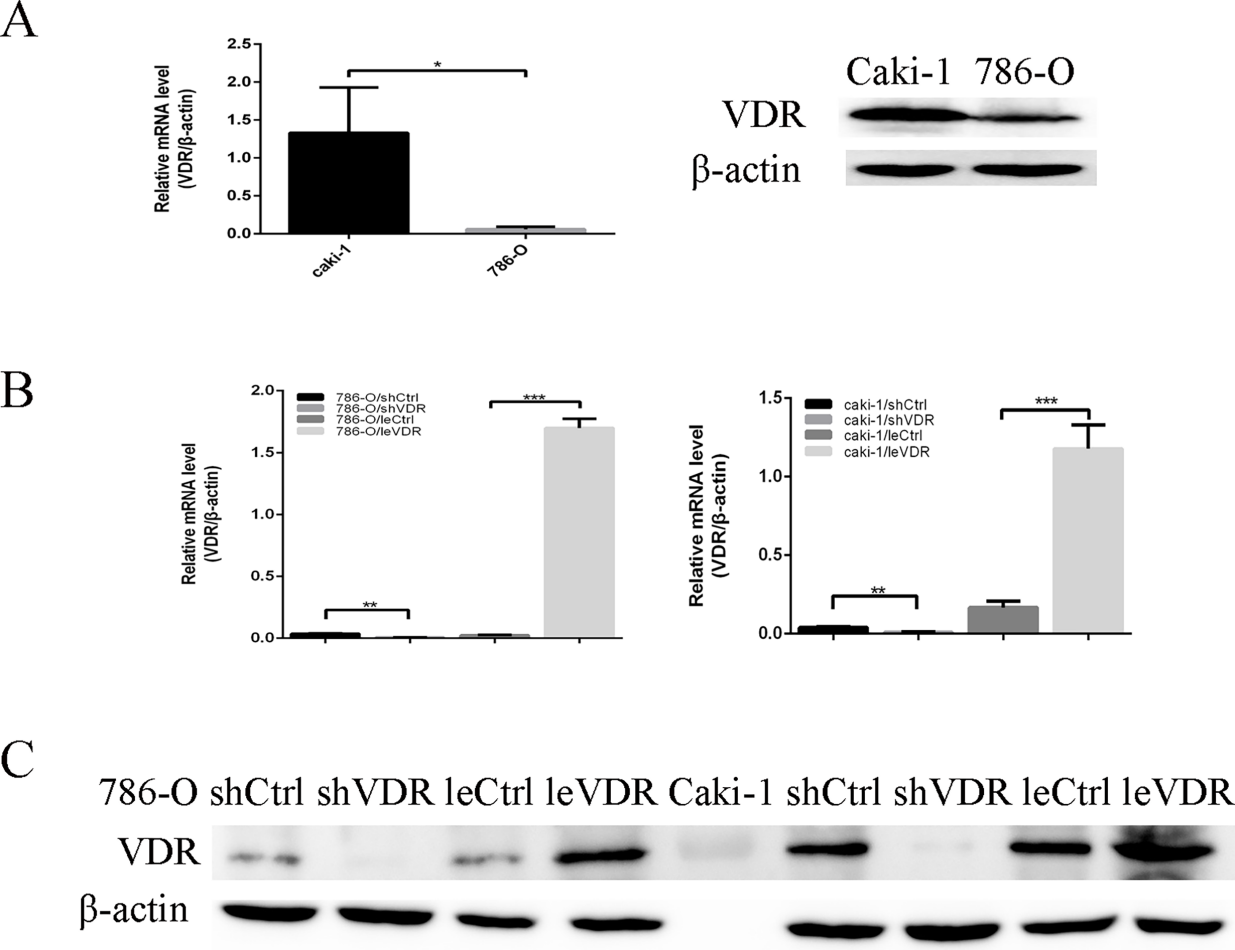
VDR expression levels in the caki-1 and 786–0 RCC cell lines with different treatments. VDR mRNA expression was measured by RT-PCR and was normalized to that of β-actin. VDR protein expression was analysed by WB, and β-actin was used as a loading control. *p <0.05, **p <0.01, ***p <0.001. (A) VDR expression levels in caki-1 cells were higher in 786-O cells. (B) & (C) Overexpression of VDR by infection with the VDR-expressing lentivirus (leVDR) and knockdown of VDR with shRNA lentivirus (shVDR) in caki-1 and 786-O cells.

To examine the expression level of VDR in RCC cell lines caki-1 and 786-O, VDR expression levels were measured by RT-PCR and WB. VDR expression in caki-1 cells was higher than that in 786-O cells (Fig 1A).To further characterize VDR function, we used lentivirus to conduct the model of VDR overexpression and knockdown in RCC caki-1 and 786-O cells. The efficiency of treatments was measured by RT-PCR (Fig 1B) and WB (Fig 1C). VDR expression was significantly down-regulated following infection with shVDR lentiviral vector that efficiently knocked down VDR expression compared with the control vector (shVDR vs shCtrl). However, VDR expression was significantly up-regulated following infection with leVDR lentiviral vector that efficiently generated VDR overexpression compared with empty control vector (leVDR vs leCtrl).

### VDR inhibits caki-1 and 786-O cell proliferation

**Fig 2:**
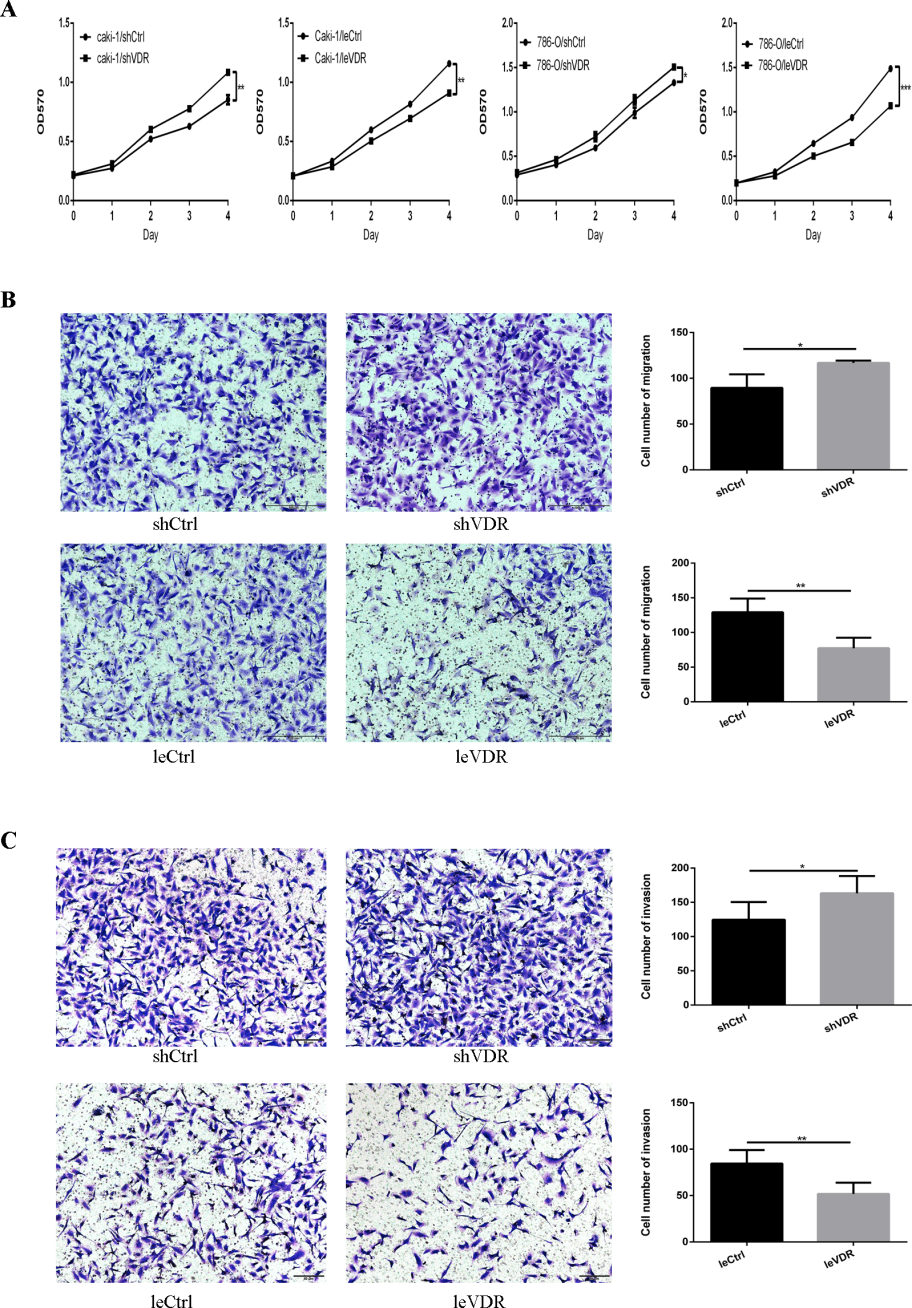
VDR inhibits RCC proliferation, migration and invasion. (A) The MTT assay showed that the proliferation of caki-1 and 786-O cells was significantly inhibited by VDR overexpression and promoted by knockdown of VDR compared with their respective control cells. Each data point represents the mean ± SD of absorbance values. Transwell migration (B) and invasion (C) assays (magnification, ×100) showed that the migration and invasion of 786-O cells were significantly decreased following infection with leVDR lentivirus and were increased following infection with shVDR lentivirus compared with each vector control. The graph indicates the mean ± SD of the number of cells from 5 random high-power fields (magnification, ×200). *p <0.05, **p <0.01, ***p <0.001.

The decreased expression of VDR in RCC tissue suggested that it might play an inhibitory role in tumourigenesis. Thus, we investigated the effect of VDR on the cell proliferation of caki-1 and 786-O cells using the MTT assay. VDR overexpression significantly inhibited the proliferation of caki-1 and 786-O cells compared with that in LeCtrl cells. By contrast, VDR knockdown promoted caki-1 and 786-O cell proliferation (Fig 2A).

### VDR inhibits 786-O cell migration and invasion

Migration and invasion are fatal steps in cancer progression. We performed transwell migration and invasion assays to examine the role of VDR in 786-O cells. The transwell migration assay results (Fig 2B) showed that VDR overexpression significantly decreased the number of migrated cells, and VDR knockdown increased the number of 786-O cells that traversed the filter compared with their respective controls. Transwell invasion assay results (Fig 2C) demonstrated that VDR overexpression in 786-O cells remarkably reduced the number of cells that passed through Matrigel compared with the vector control. The VDR knockdown groups had fewer cells passed through the Matrigel than the vector control. These results suggest that VDR inhibits 786-O cell migration and invasion.

### VDR promotes caki-1 cell apoptosis

**Fig 3:**
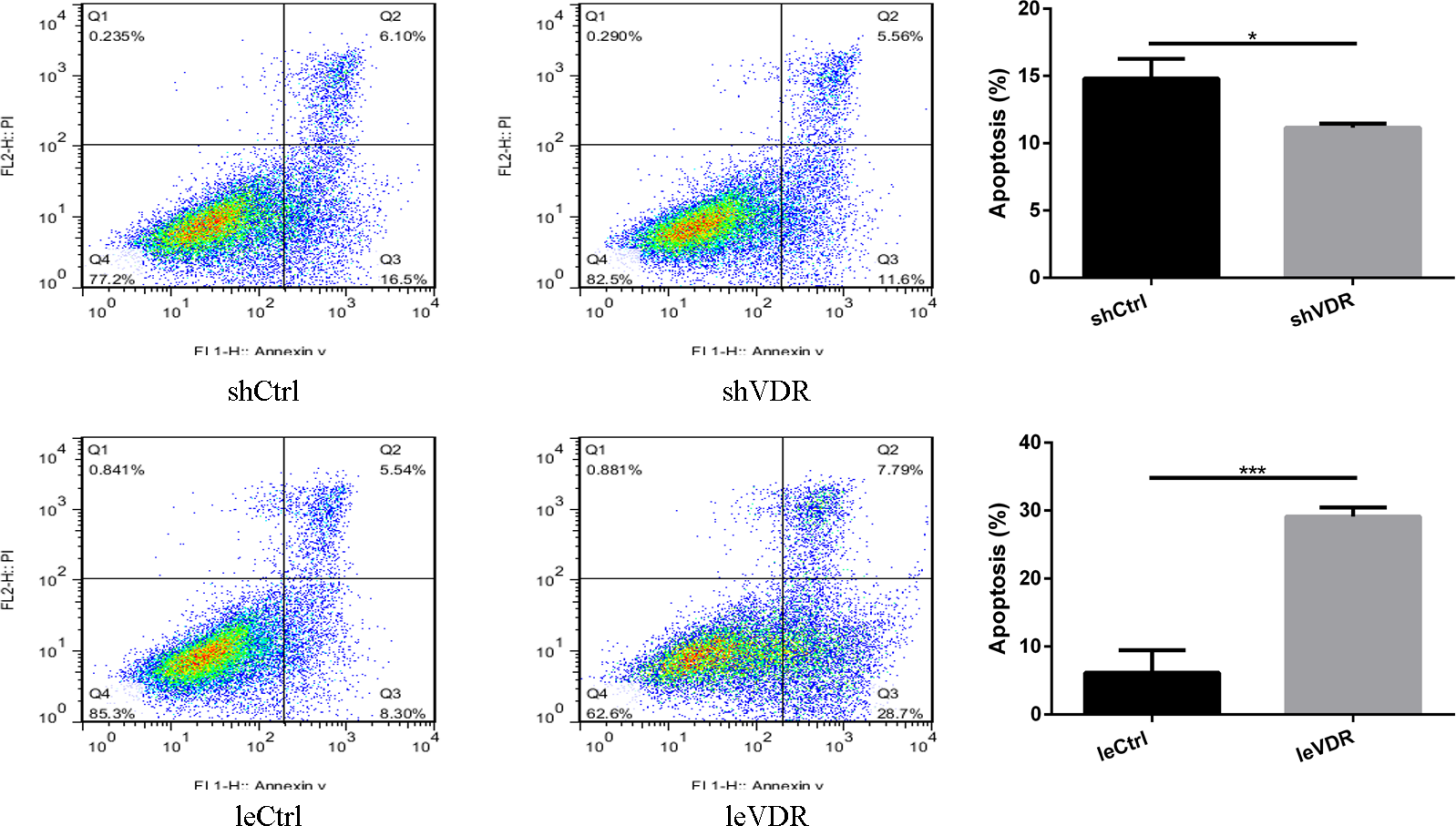
VDR promotes RCC apoptosis. Flow cytometry indicated that VDR overexpression increases caki-1 cell apoptosis, and VDR knockdown decreases caki-1 cell apoptosis. Graphs for each treatment group and the percentage of cell apoptosis are shown. X-axis: PL1-H Annexin V; Y-axis: PL2-H PI. The data were expressed as means ± SD of three independent experiments. *p <0.05, ***p <0.001.

Cell apoptosis assays were performed using flow cytometry. Caki-1 cell apoptosis was significantly increased in the VDR-overexpressed caki-1 cells compared with that in the control group cells. However, apoptosis was decreased in the VDR knockdown caki-1 cells compared with that in the control cells (Fig 3). These results indicate that VDR expression is an important determinant of apoptosis in caki-1 cells.

### Changes in VDR regulate the expression of TRPV5

**Fig 4:**
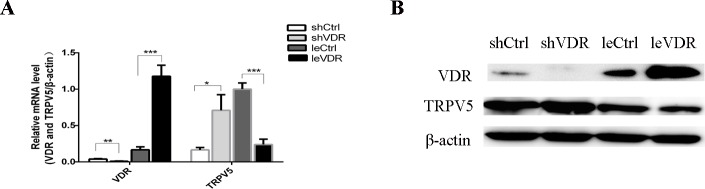
VDR regulates TRPV5 expression. (A) RT-PCR and (B) WB were performed to examine the expression of TRPV5 following VDR overexpression or knockdown in caki-1 cells. Changes in TRPV5 expression were increased following infection with shVDR lentivirus and were decreased following infection with leVDR lentivirus. *p <0.05, **p <0.01, ***p <0.001.

To understand the relationship between VDR and TRPV5, we performed RT-PCR and WB analyses to measure TRPV5 expression after VDR overexpression and knockdown in caki-1 cells. The shVDR lentiviral-infected group had higher levels of TRPV5 expression than the shCtrl group, whereas the leVDR lentivirus-infected group had lower levels of TRPV5 expression than the le-Ctrl group (Fig 4). These results suggest that changes in VDR alter the expression of TRPV5 at the mRNA and protein levels, and the TRPV5 expression level was negatively correlated with VDR expression.

### TRPV5 regulates caki-1 RCC cell proliferation and metastasis via TRPV5

**Fig 5:**
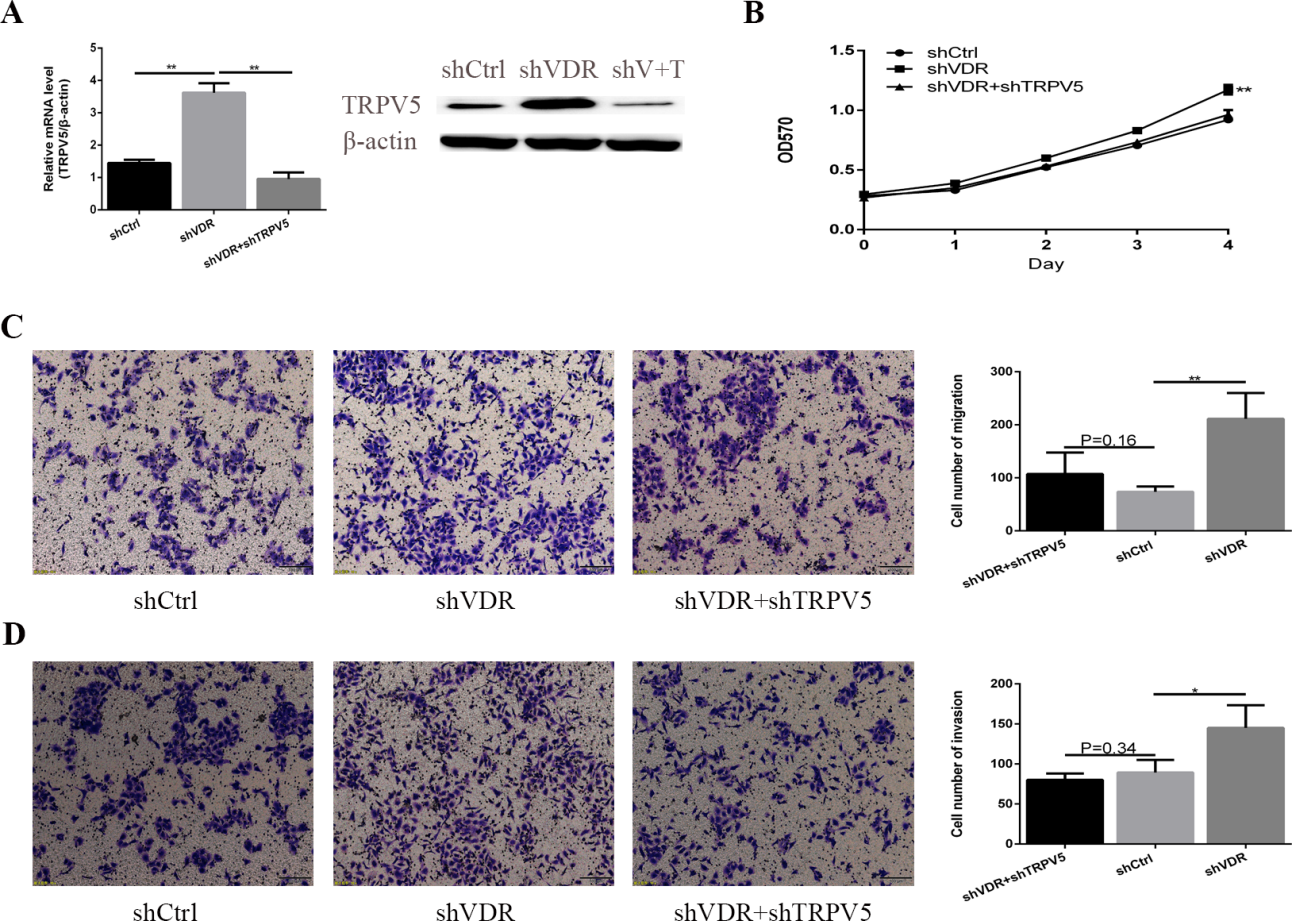
TRPV5 knockdown reverses the effect of VDR knockdown on caki-1 cell proliferation, migration and invasion. (A) TRPV5 mRNA expression was detected by RT-PCR and was normalized to that of β-actin. TRPV5 protein expression was detected by WB using β-actin as a loading control. shV+T is shVDR+shTRPV5 co-treated. (B) Cell proliferation ability was analysed by the MTT assay in control, shVDR-treated, or shVDR+shTRPV5-treated caki-1 cells; (C-D) Cell migration and invasion ability were measured by the transwell assay. The graph indicates the mean ± SD of the number of cells from 5 random high-power fields (magnification, ×200). *p <0.05, **p <0.01.

To further investigate the mechanism underlying TRPV5 and VDR expression changes in RCC, we next constructed a shTRPV5 lentivirus vector to knockdown TRPV5 expression. After co-treating caki-1 cells with shVDR and shTRPV5, we analysed the TRPV5 expression level by RT-PCR and WB, revealing that TRPV5 expression was decreased (Fig 5A). The MTT, transwell migration and invasion assays revealed that VDR knockdown significantly promoted the proliferation, migration and invasion of caki-1 cells compared with that of the control vector in caki-1 cells. However, knockdown of TRPV5 reversed the effect of VDR knockdown on caki-1 cell proliferation, migration and invasion, and there was no significantly change in the migration, cell number regarding migration (p = 0.16) and invasion (p = 0.34) in shVDR+shTRPV5 co-treated caki-1 cells compared with that in the control lentiviral vector control cells (Fig 5B–5D). These data suggest that VDR regulated caki-1 cell proliferation and metastasis via TRPV5.

### Bioinformatics analysis of differently expressed genes in VDR-overexpression and -knockdown caki-1 cells

**Fig 6:**
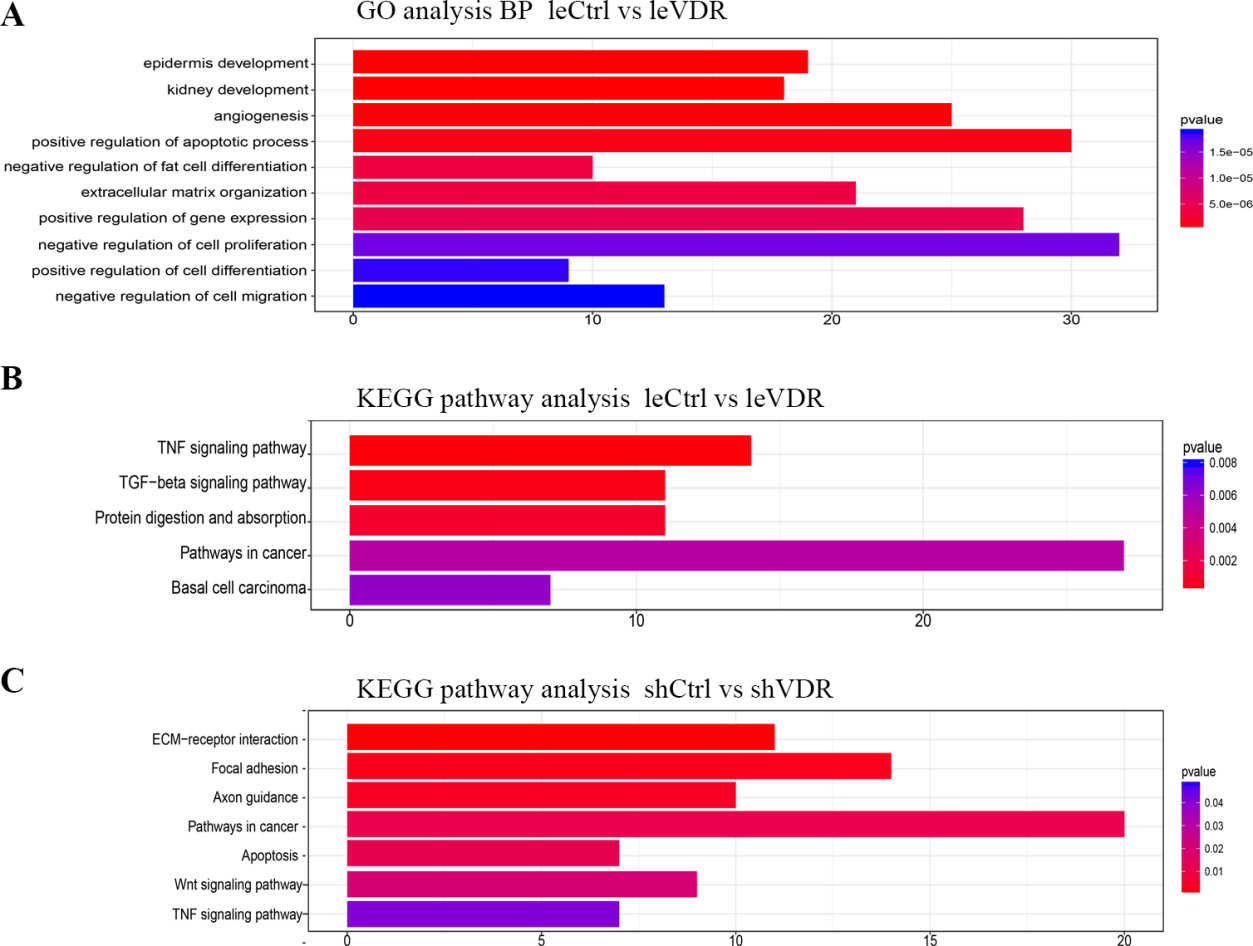
Bioinformatics analysis of differently expressed genes after VDR overexpression and knockdown in caki-1 cells. After VDR overexpression, GO analysis of biological process (A) indicated differentially expressed genes were associated with cell apoptotic, differentiation, proliferation and migration. KEGG pathway enrichment analysis (B) indicated differentially expressed genes were associated with the TNF signalling pathway, TGF-beta signalling pathway and carcinogenesis pathway. In the VDR knockdown cells, (C) KEGG pathway enrichment analysis indicated that significantly differentially expressed genes were associated with the TNF signalling pathway, apoptosis, Wnt signalling pathway and the carcinogenesis pathway.

To investigate the effect of the overexpression and knockdown of VDR on gene expression in caki-1 cells, we performed the RNA-sequence assay on different infected cells. Seven hundred eighty-four significantly differentially expressed genes, including 539 up-regulated and 245 down-regulated genes, were detected after VDR overexpression in caki-1 cells compared with those in control cells. Gene Ontology (GO) analysis of biological processes indicated significantly differentially expressed genes were associated with cell apoptosis, differentiation, proliferation and migration (Fig 6A). KEGG pathway enrichment analysis indicated that significantly differentially expressed genes were associated with the TNF signalling pathway, TGF-beta signalling pathway and carcinogenesis pathway (Fig 6B). However, 681 significantly differentially expressed genes, including 244 up-regulated and 437 down-regulated genes, were detected after VDR knockdown in caki-1 cells compared with that in the control cells. KEGG pathway enrichment analysis indicated this significantly differentially expressed genes were associated with the TNF signalling pathway, apoptosis, the Wnt signalling pathway and the carcinogenesis pathway (Fig 6C). These results suggest that VDR plays an important role in the biological functions and signalling pathways of caki-1 cells.

## Discussion

As the biologically active metabolite of vitamin D, 1,25(OH)_2_D exerts strong anti-proliferative, pro-differentiation and pro-apoptotic actions in some cells, tissues and cancers [20,21]. In addition to its ability to reduce the risk of RCC, 1,25(OH)_2_D levels are negatively correlated with the risk of colorectal cancer, breast cancer, lung cancer and bladder cancer [22–26], and its intervention could also improve the prognosis of patients with haematological malignancies [27]. Most of the known biological effects of 1,25(OH)_2_D are mediated through binding to VDR, a nucleoprotein that belongs to the steroid hormone receptor superfamily [20,21]. Thus, VDR may be involve in cancer development and might reduce the risk of cancer associated with 1,25(OH)_2_D. However, the expression and role of VDR in tumours have been reported previously for other types of cancer. The high expression of VDR in prostate tumours reduces the risk of lethal cancer [28]. Additionally, VDR knock-out mice show increased sensitivity to carcinogen challenge [20]. Furthermore, VDR expression was associated with clinical prognostic factors such as tumour size and lymph node involvement in breast cancer, suggesting that VDR expression may be clinically significant and VDR may be a factor with prognostic relevance [29]. These lines of evidence demonstrated that VDR may interact with 1,25(OH)_2_D to reduce the risk of cancer and play a potential antitumour role in the development and progression of cancer.

The present study found that VDR was co-expressed in the caki-1 and 786-O RCC cell lines, and VDR was expressed at a high level in caki-1 cells with high metastatic potential and at a low level in 786-O cells with low metastatic potential. To determine the functional roles of VDR in RCC cell lines, we used the shVDR lentivirus vector, which efficiently silenced VDR expression, or lentivirus carrying the VDR gene, which efficiently generated VDR overexpression in the RCC cell lines. Next, we performed a series of functional experiments. As expected, VDR overexpression significantly inhibited cell proliferation, migration, and invasion and promoted apoptosis in RCC cell lines. Conversely, VDR knockdown promoted cell proliferation, migration, and invasion and inhibited apoptosis. Taken together, our findings confirmed that VDR functions as a tumour suppressor in RCC cell lines. Similar to these functional experiments, our RNA-sequence analysis results showed significantly differentially expressed genes were associated with cell apoptosis, proliferation and migration by GO analysis in VDR-overexpression caki-1 cells compared with those in the vector control cells.

Several studies have examined how VDR exerts antitumour efficacy. For instance, VDR and 1,25(OH)_2_D can inhibit cell proliferation through up-regulation of the cyclin-dependent kinase inhibitor p21 and p27 [30,31]. Additionally, VDR inhibits cell migration and invasion *via* inhibiting the Wnt/β-catenin signalling pathway and increasing the expression of E-cadherin [32–34]. VDR can also play a pro-apoptotic role by inhibiting the expression of anti-apoptotic proteins Bcl-2 and Bcl-XL [35]. The Wnt signalling pathway and apoptosis pathway were detected in this study by KEGG pathway enrichment analysis of RNA-sequence analysis, which was performed on VDR-overexpression and -knockdown caki-1 cells. In addition, the TGF-β signalling pathway was related to VDR and 1,25(OH)_2_D [36,37], which were also detected in this study and could act as tumour suppressors [38]. Thus, VDR may function through these pathways to exert antitumour efficacy in RCC cell lines.

VDR and 1,25(OH)_2_D_3_ were reported to play a regulatory role in TRPV5 activity. The mRNA and protein expression levels of TRPV5 were decreased in the kidneys of vitamin D-deficient or VDR knock-out mice, and the injection of 1,25(OH)_2_D_3_ could significantly increase the mRNA expression of *TRPV5* in kidneys. Thus, the expression of TRPV5 is strongly dependent on the intake of vitamin D. Moreover, the human TRPV5 promoter contains several consensus vitamin D-responsive elements [18,19]. Our previous study also found that the expression of TRPV5 was associated with VDR. In this study, we further confirmed that the TRPV5 mRNA and protein expression levels were regulated by VDR, in which VDR overexpression down-regulated TRPV5 expression whereas VDR knockdown up-regulated TRPV5 expression. The above studies suggest that VDR could regulate the transcription of TRPV5.

Several studies showed that TRPV5 is involved in tumours. TRPV5 is poorly expressed or not expressed in normal colon tissues but is highly expressed in colon adenoma and adenocarcinoma [13]. TRPV5 expression was also found to be increased in adenoma samples compared with that in normal parathyroid glands [14]. On the other hand, decreased expression of TRPV5 in tumour tissues was observed in non-small cell lung cancer patients and was associated with a shorter median survival time after surgical resection [15], and different expression levels of TRPV5 were detected among the different RCC histopathological subtypes that arise from different origins [16]. Furthermore, the present study demonstrated that knockdown of TRPV5 expression in caki-1 cells suppressed VDR knockdown-induced changes in proliferation, migration and invasion ability. These findings likely suggest that altered TRPV5 expression may be associated with RCC carcinogenesis. At the same time, we verified that VDR could regulate the transcription of TRPV5. Therefore, we presume that VDR could suppress the proliferation and metastasis of RCC cell lines *via* regulation of TRPV5 expression.

As a cellular Ca^2+^ channel, TRPV5 is predominantly expressed in response to the Ca^2+^ influx step in the process of transcellular Ca^2+^ transport in the kidney [11]. The role of Ca^2+^ in the overall cancer-related cell signalling pathways is uncontested. Alterations in Ca^2+^ homoeostasis increase proliferation and induce differentiation or apoptosis [39,40]. The calcium signalling pathway may be the link between VDR and TRPV5. Vitamin D interacts with VDR to regulate the transcription of TRPV5, and then TRPV5 modulates the cellular calcium concentration and affects the biological behaviour of RCC cells.

There were several limitations in our present study. A negative correlation between TRPV5 and VDR was shown in RCC cell lines; however, the precise mechanism by which VDR suppresses migration and invasion *via* TRPV5 remains clear. In addition, additional pathways may be involved in the VDR regulation of biological processes in RCC and warrant further investigation.

In conclusion, VDR could suppress RCC carcinogenesis, whereas VDR knockdown led to promoting effects. Moreover, TRPV5 expression levels were negatively correlated with VDR, and VDR could suppress the proliferation, migration and invasion of RCC *via* regulation of TRPV5 expression. A better understanding of the role and relationship of VDR and TRPV5 in tumourigenesis might provide new gene therapy strategies for RCC.

## Supporting information

S1 Fig VDR expression levels in the caki-1 and 786–0 RCC cell lines with different treatments. (ZIP)

S2 Fig VDR inhibits RCC proliferation, migration and invasion. (ZIP)

S3 Fig VDR promotes RCC apoptosis. (ZIP)

S4 Fig VDR regulates TRPV5 expression. (ZIP)

S5 Fig TRPV5 knockdown reverses the effect of VDR knockdown on caki-1 cell proliferation, migration and invasion. (ZIP)

S6 Fig Bioinformatics analysis of differently expressed genes after VDR overexpression and knockdown in caki-1 cells. (ZIP)
