# Supplementary figures and images for: Vitamin D receptor suppresses proliferation and metastasis in renal cell carcinoma cell lines via regulating the expression of the epithelial Ca2+ channel TRPV5 (part 1 of 2)
Source: PLoS One. 2018 Apr 16;13(4):e0195844. doi: 10.1371/journal.pone.0195844 (PMC5901920; doi:10.1371/journal.pone.0195844)

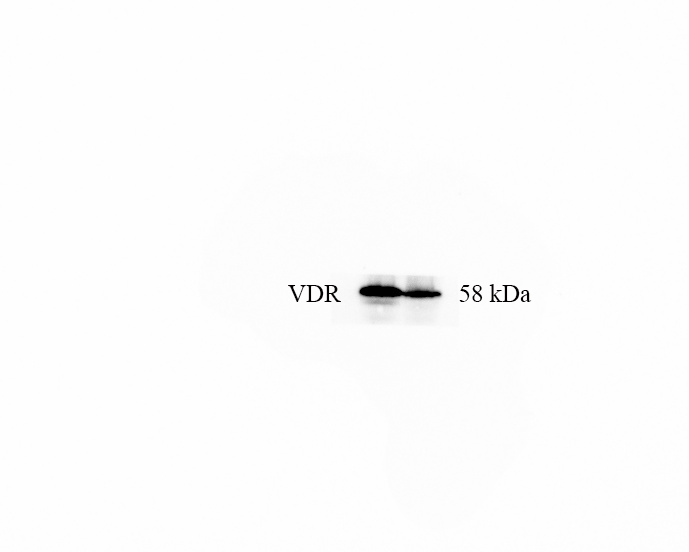

Supplement: S1 Fig — (ZIP) [file pone.0195844.s001.zip › S1 Appendix/S1_Fig1A-WB-VDR.tif]

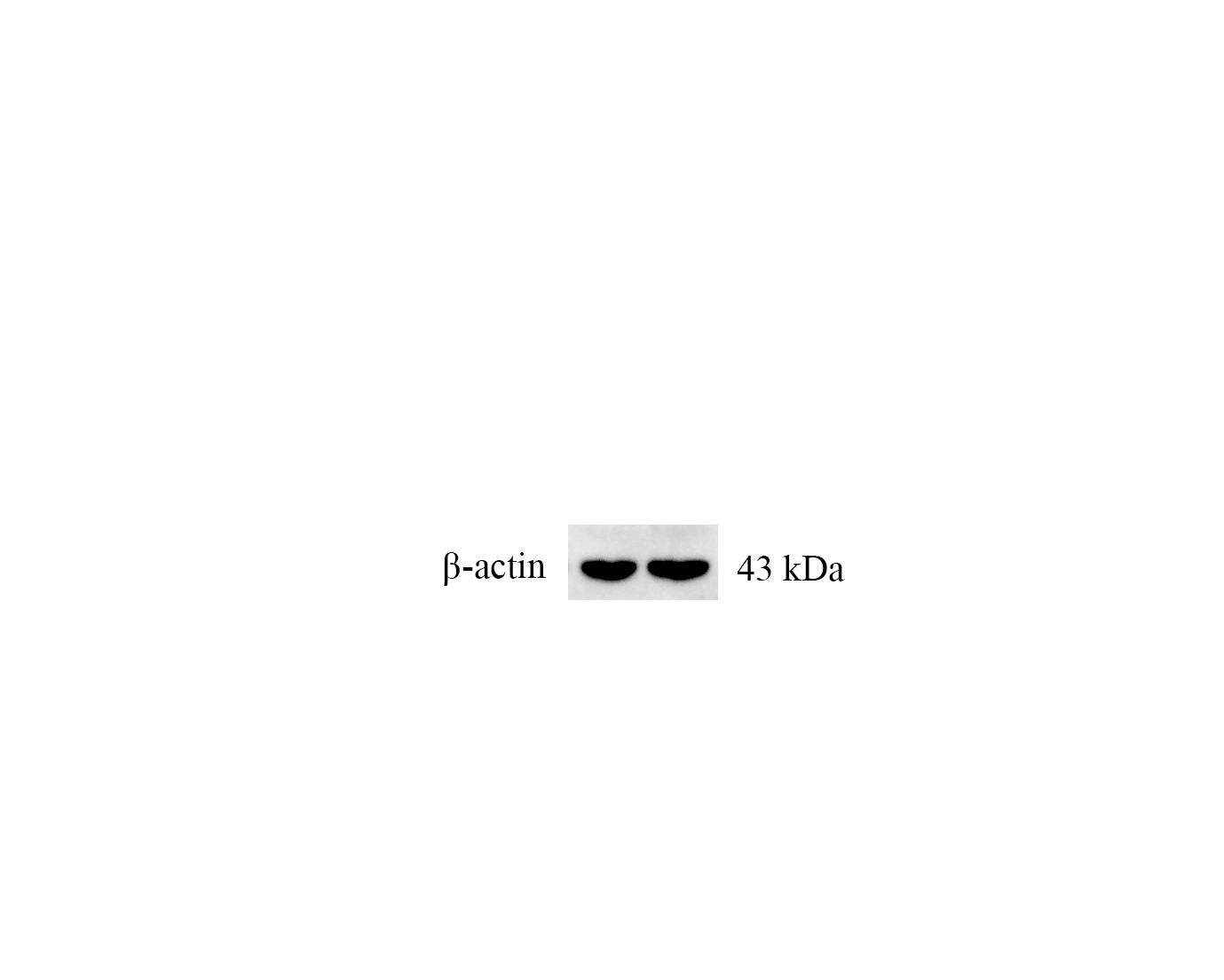

Supplement: S1 Fig — (ZIP) [file pone.0195844.s001.zip › S1 Appendix/S1_Fig1A-WB-a┬-actin.tif]

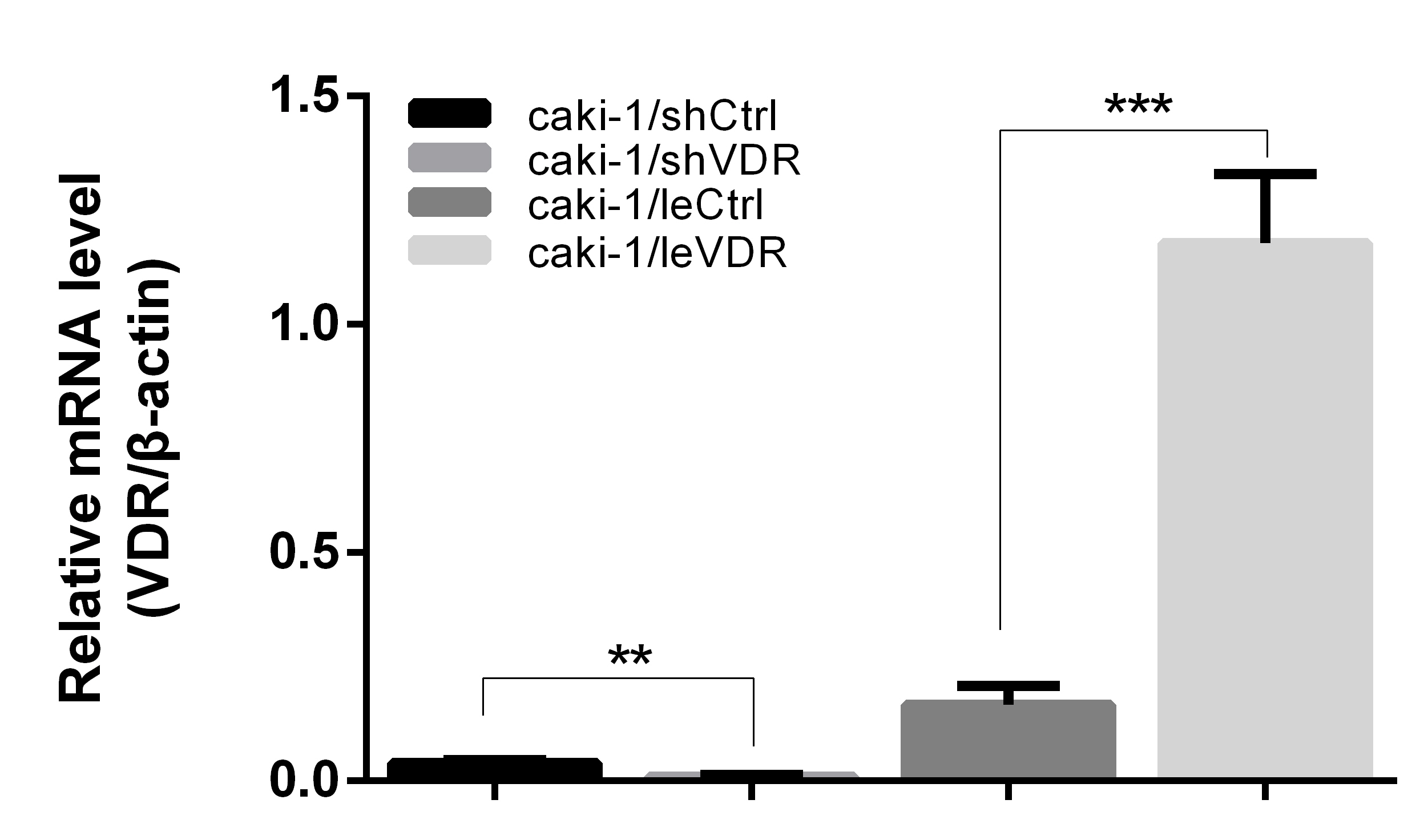

Supplement: S1 Fig — (ZIP) [file pone.0195844.s001.zip › S1 Appendix/S1_Fig1B-Caki-1-RTPCR-VDR.jpg]

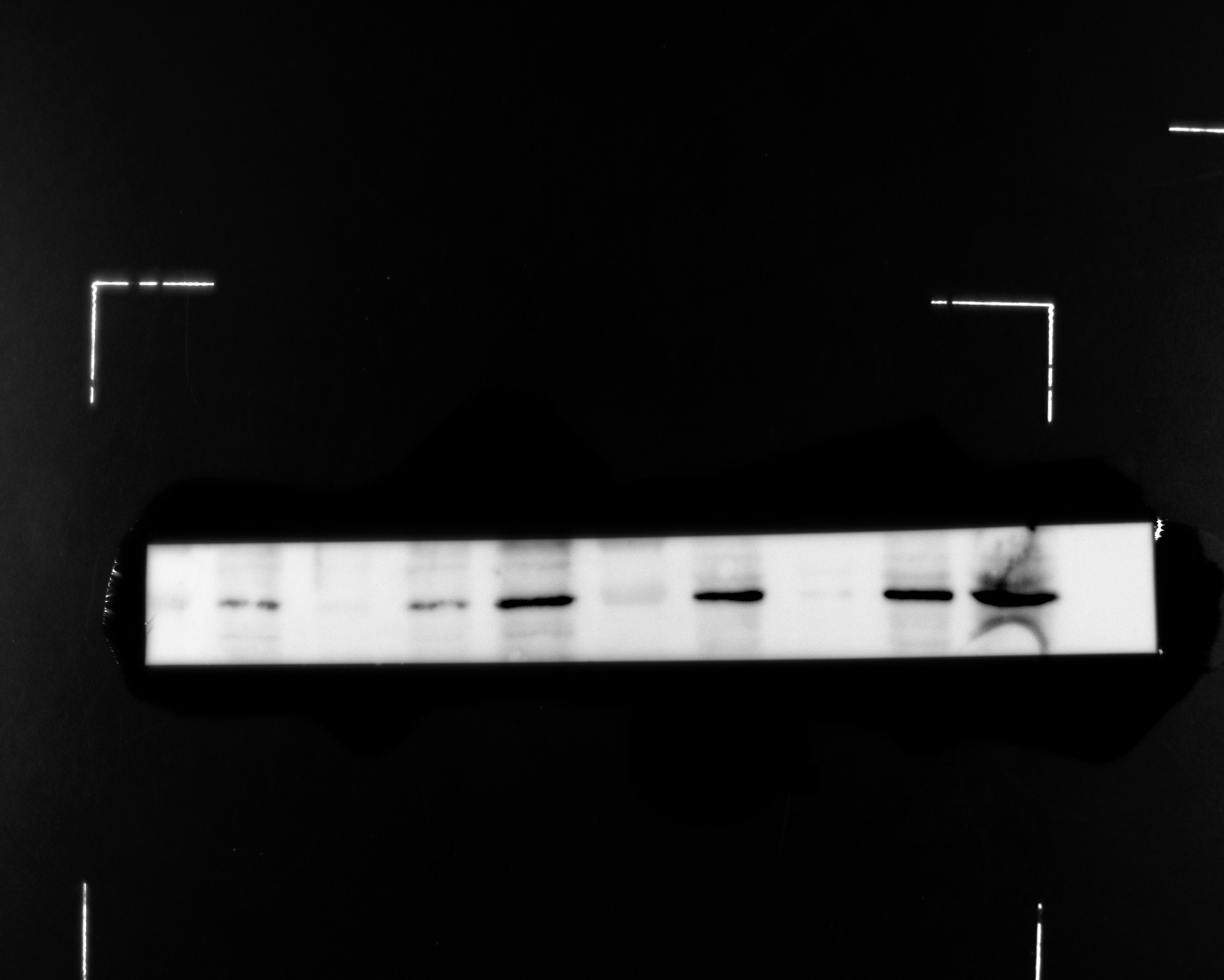

Supplement: S1 Fig — (ZIP) [file pone.0195844.s001.zip › S1 Appendix/S1_Fig1C-WB-VDR.jpg]

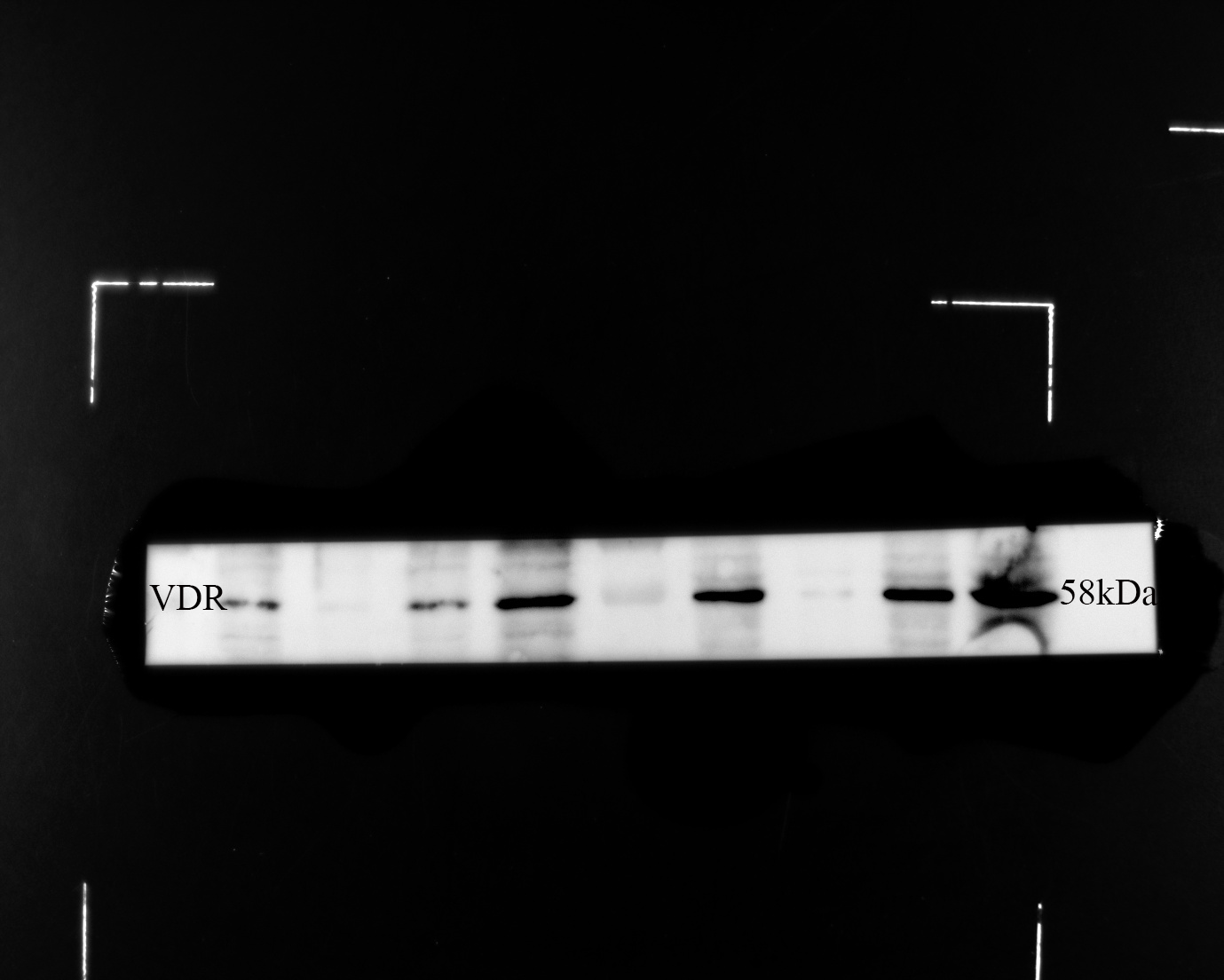

Supplement: S1 Fig — (ZIP) [file pone.0195844.s001.zip › S1 Appendix/S1_Fig1C-WB-VDR.tif]

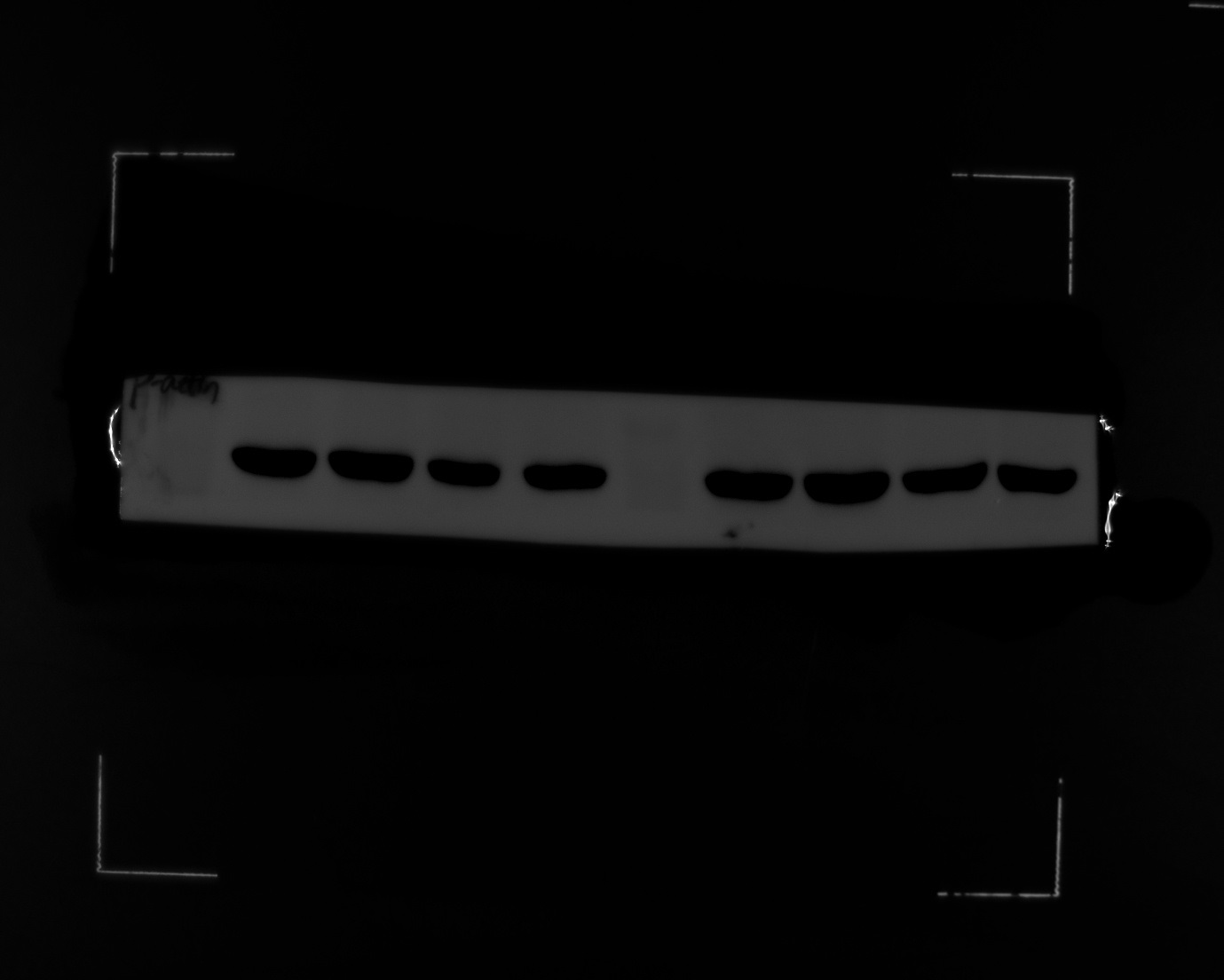

Supplement: S1 Fig — (ZIP) [file pone.0195844.s001.zip › S1 Appendix/S1_Fig1C-WB-a┬-actin.jpg]

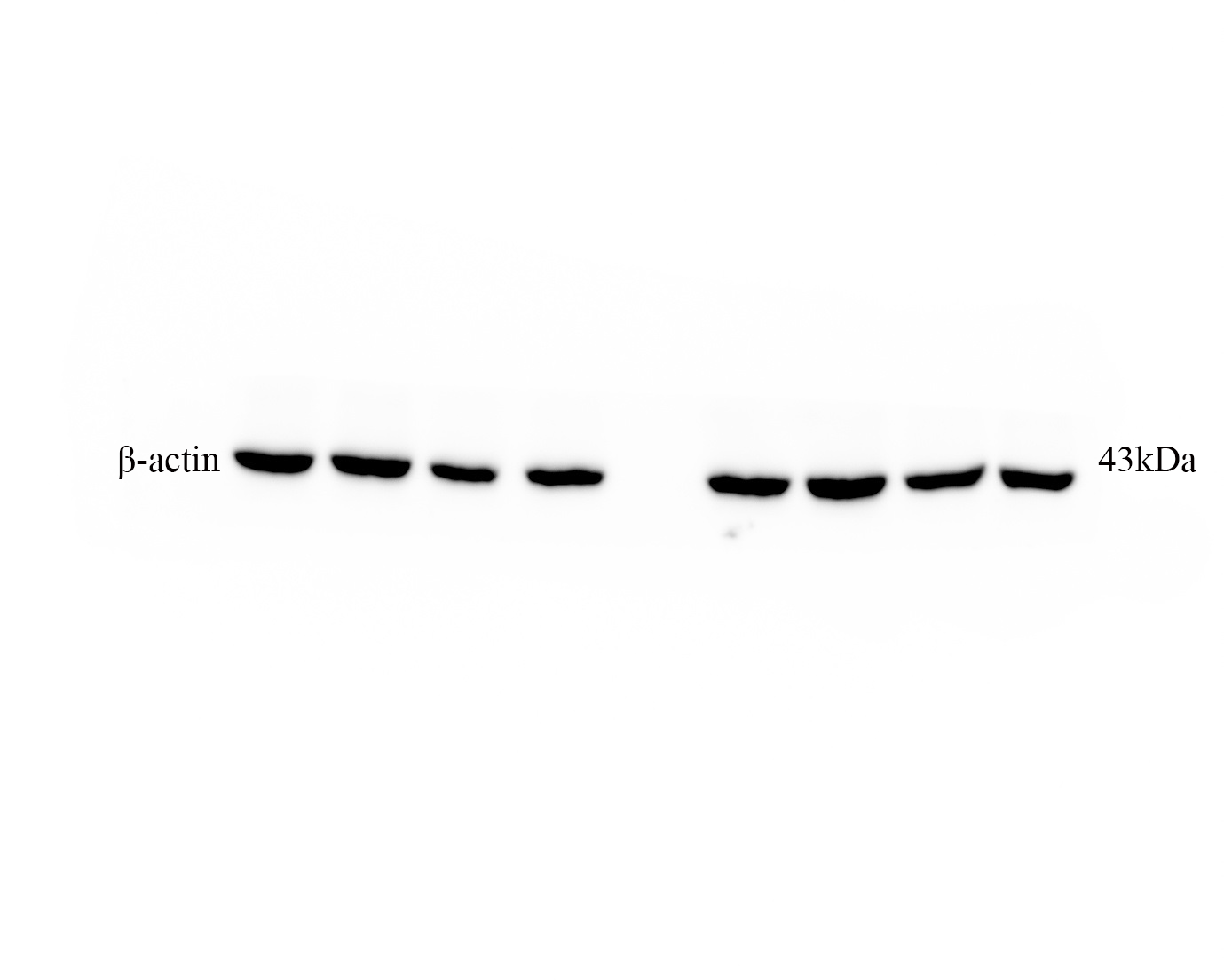

Supplement: S1 Fig — (ZIP) [file pone.0195844.s001.zip › S1 Appendix/S1_Fig1C-WB-a┬-actin.tif]

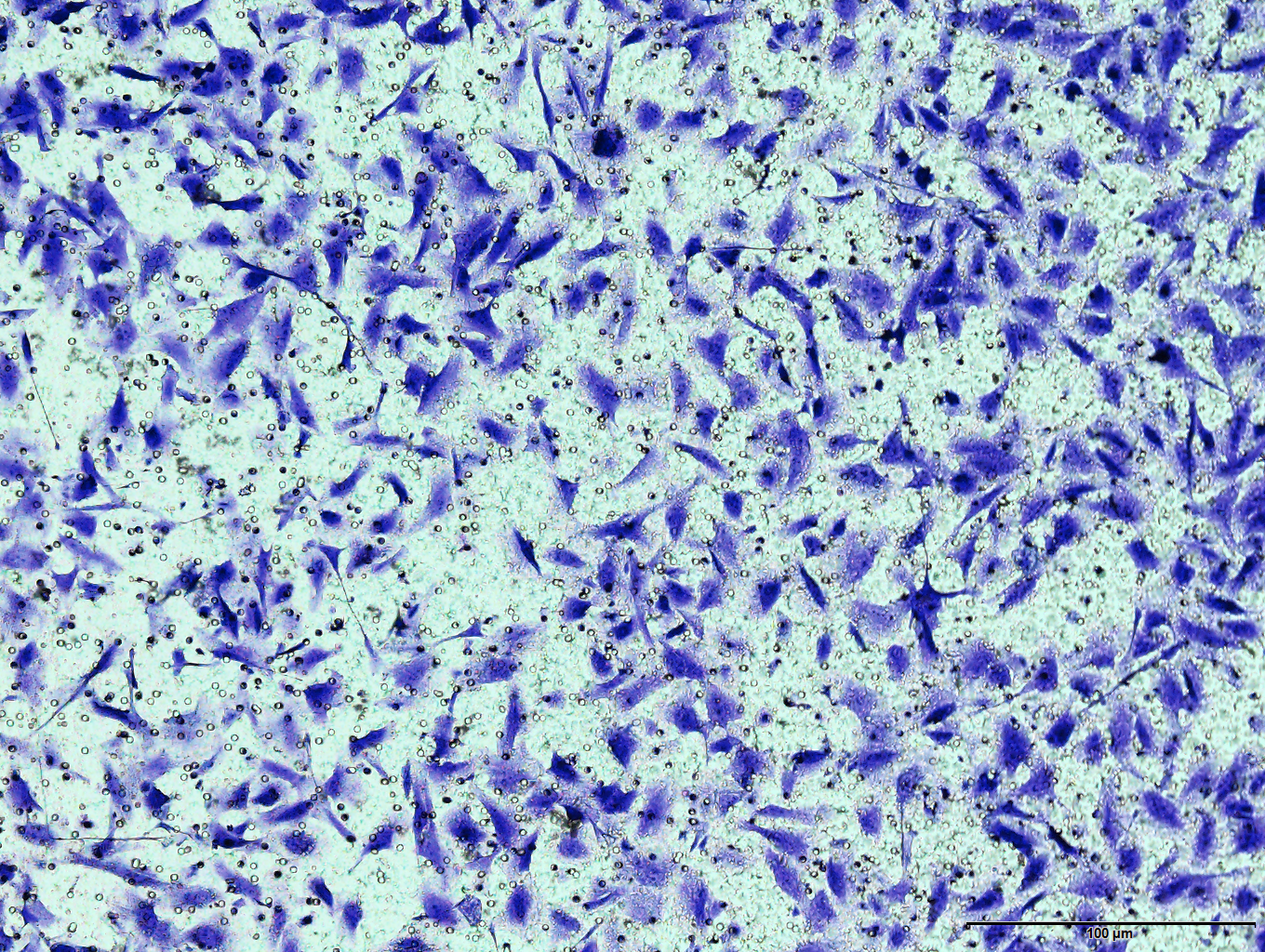

Supplement: S2 Fig — (ZIP) [file pone.0195844.s002.zip › S2 Appendix/S2_Fig2B-Mig-786O leCtrl í┴100.tif]

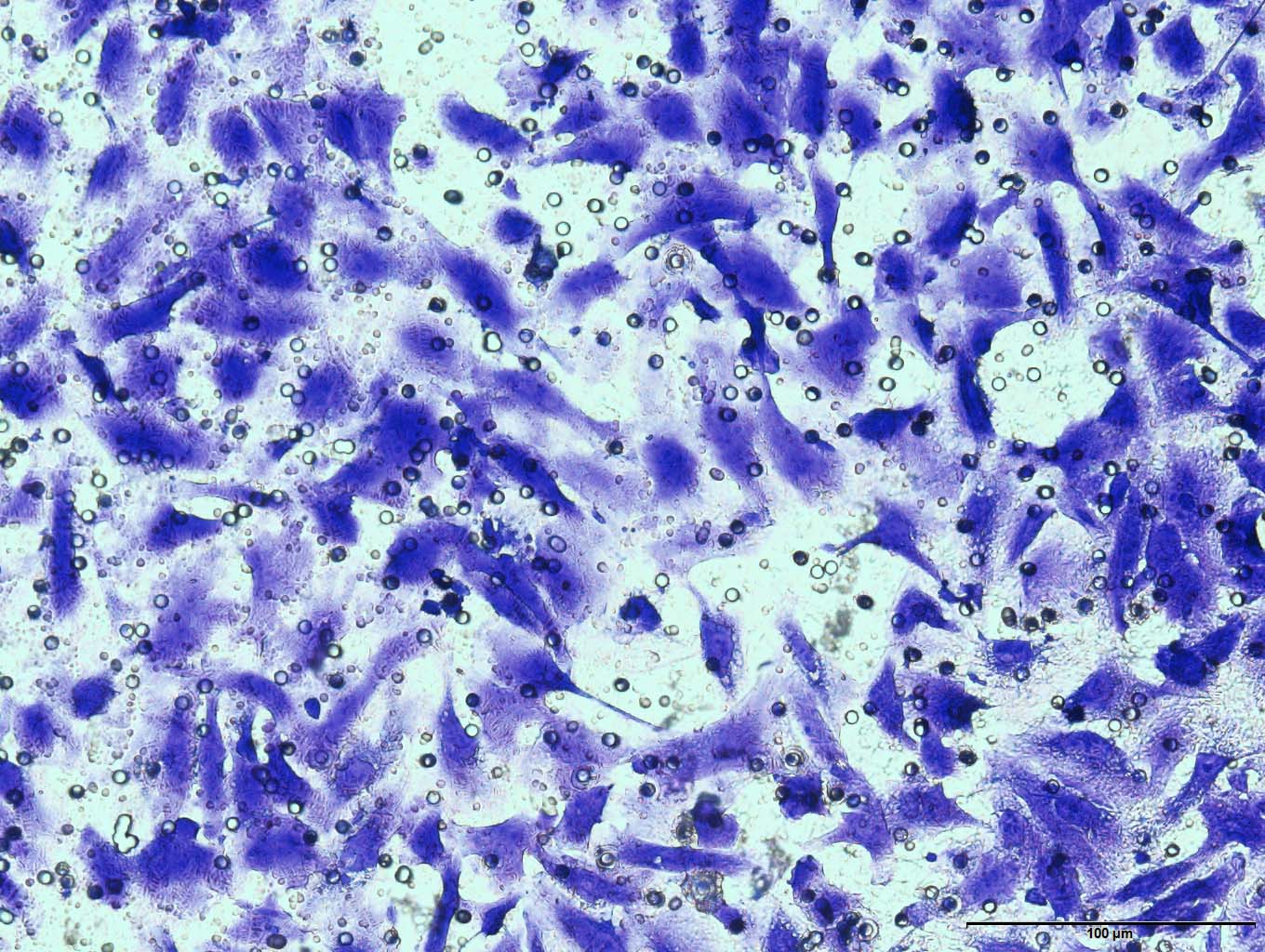

Supplement: S2 Fig — (ZIP) [file pone.0195844.s002.zip › S2 Appendix/S2_Fig2B-Mig-786O leCtrl í┴200 (1).jpg]

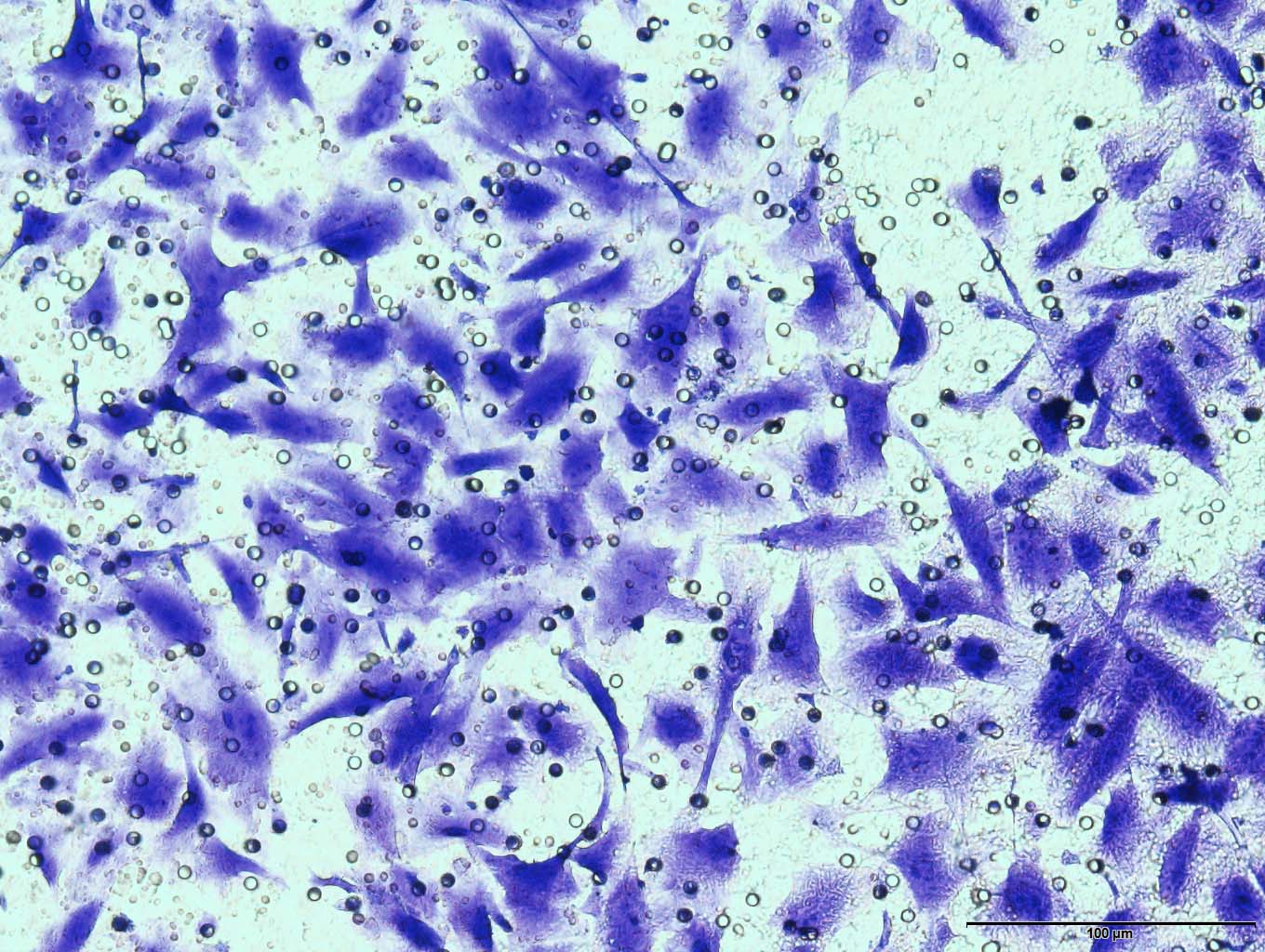

Supplement: S2 Fig — (ZIP) [file pone.0195844.s002.zip › S2 Appendix/S2_Fig2B-Mig-786O leCtrl í┴200 (2).jpg]

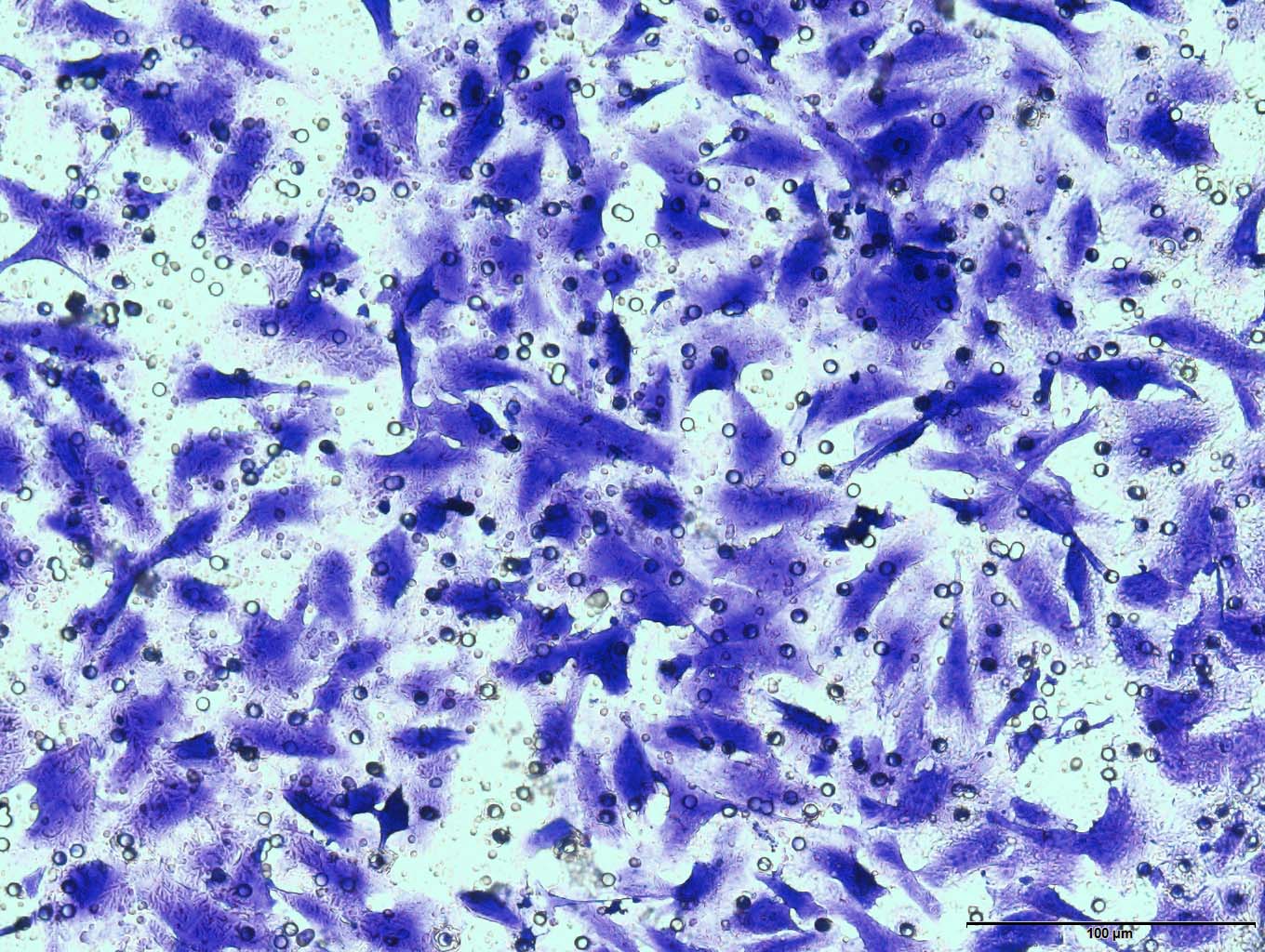

Supplement: S2 Fig — (ZIP) [file pone.0195844.s002.zip › S2 Appendix/S2_Fig2B-Mig-786O leCtrl í┴200 (3).jpg]

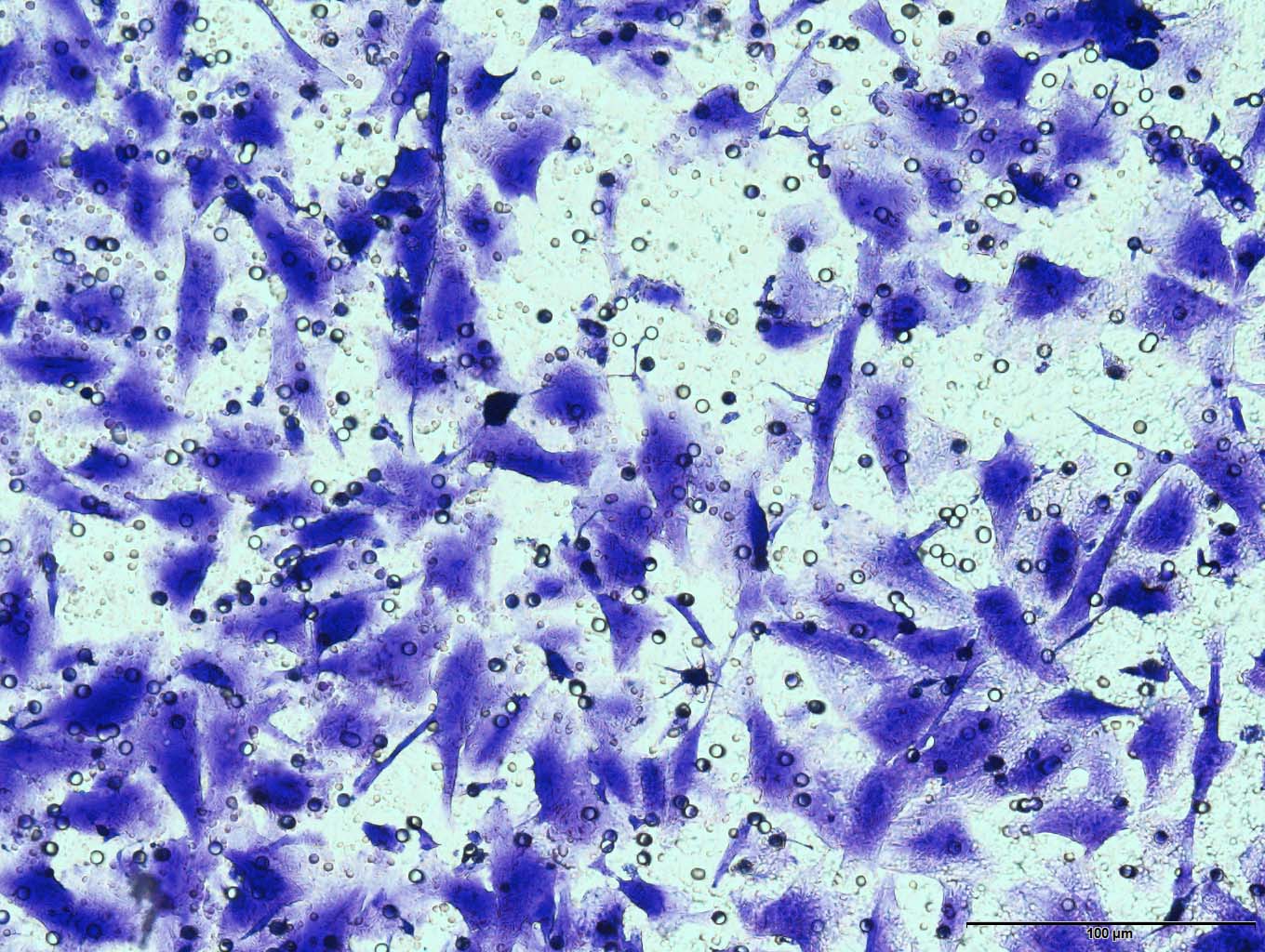

Supplement: S2 Fig — (ZIP) [file pone.0195844.s002.zip › S2 Appendix/S2_Fig2B-Mig-786O leCtrl í┴200 (4).jpg]

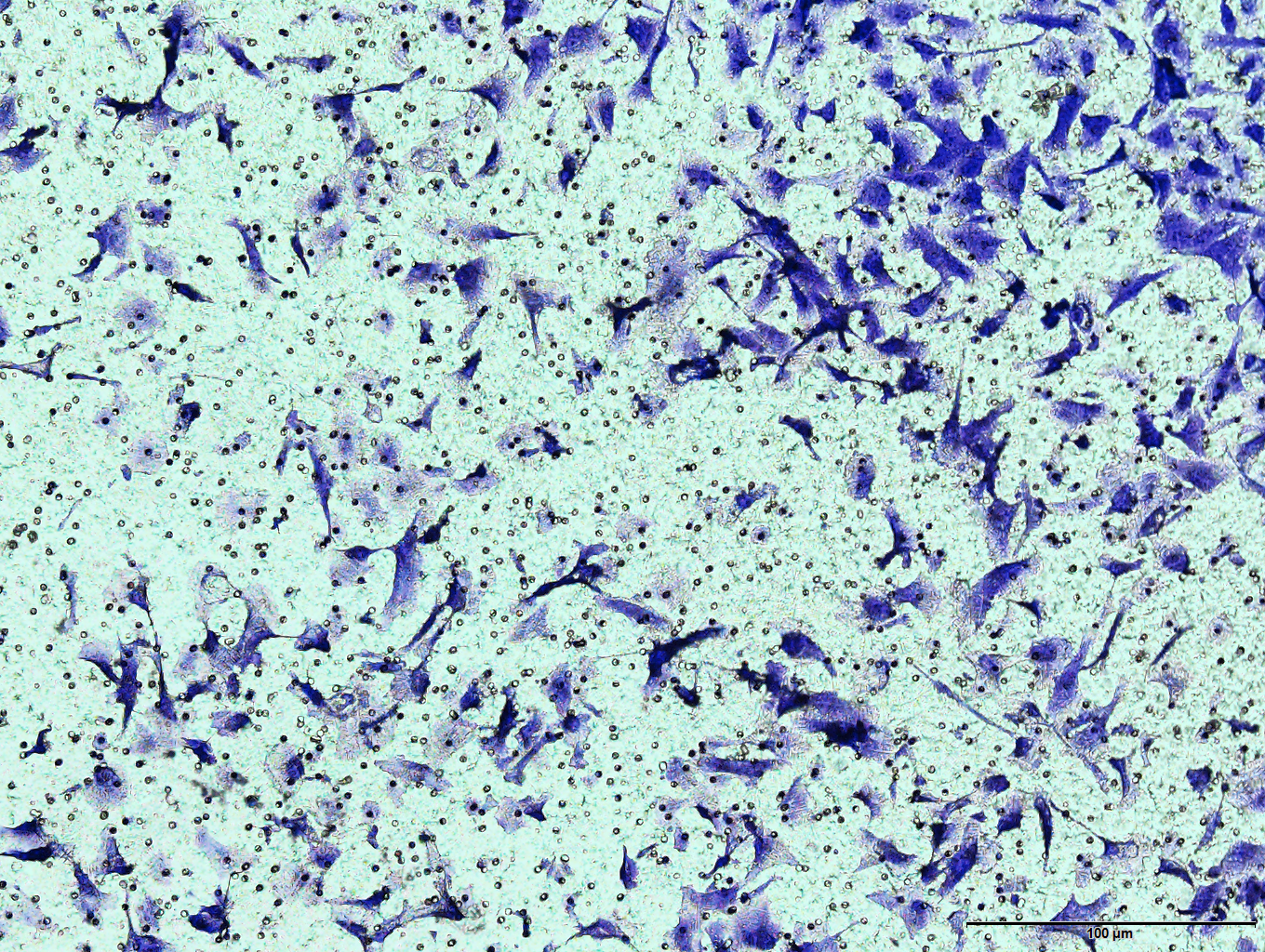

Supplement: S2 Fig — (ZIP) [file pone.0195844.s002.zip › S2 Appendix/S2_Fig2B-Mig-786O leVDR í┴100.tif]

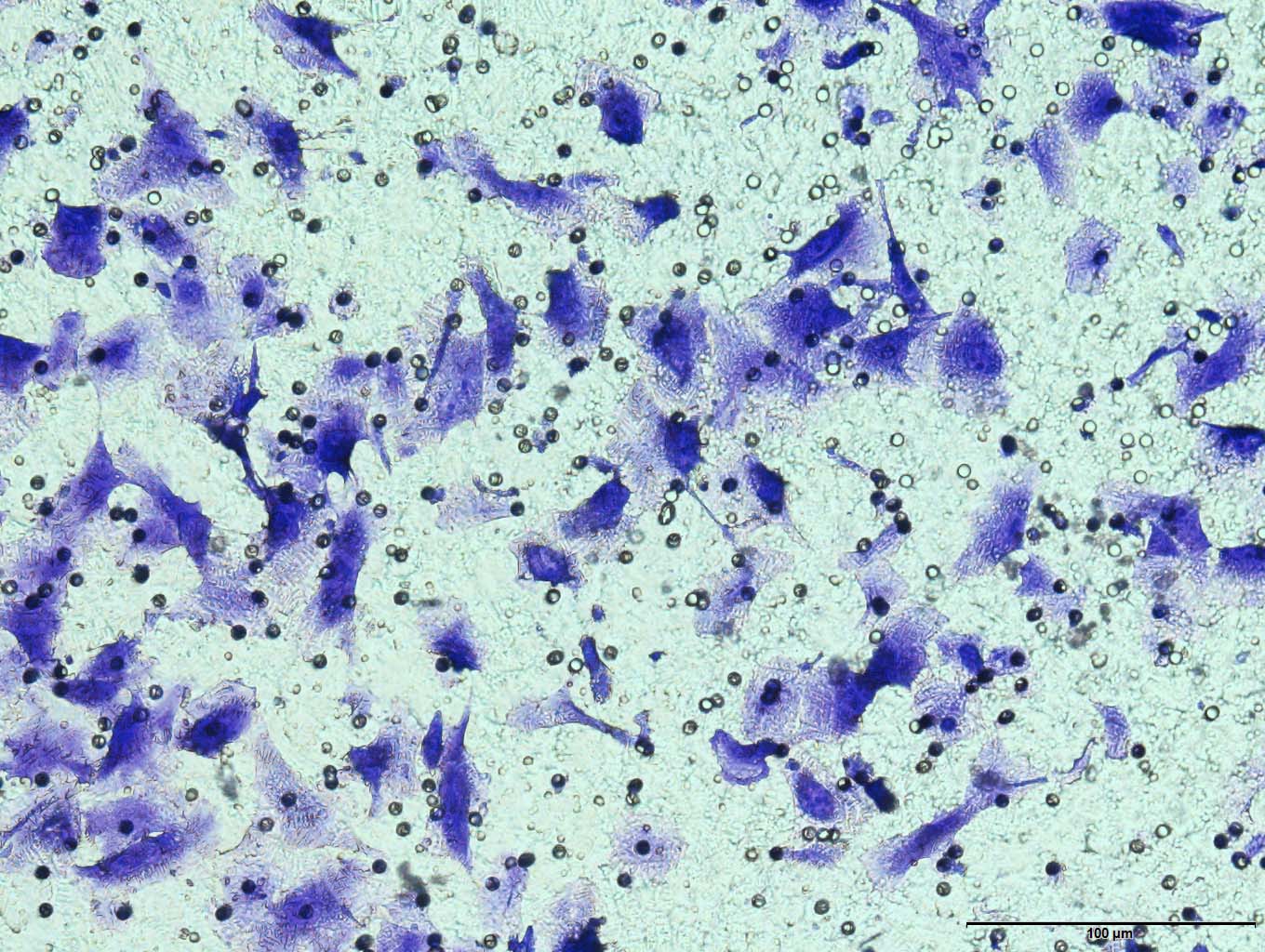

Supplement: S2 Fig — (ZIP) [file pone.0195844.s002.zip › S2 Appendix/S2_Fig2B-Mig-786O leVDRí┴200 (1).jpg]

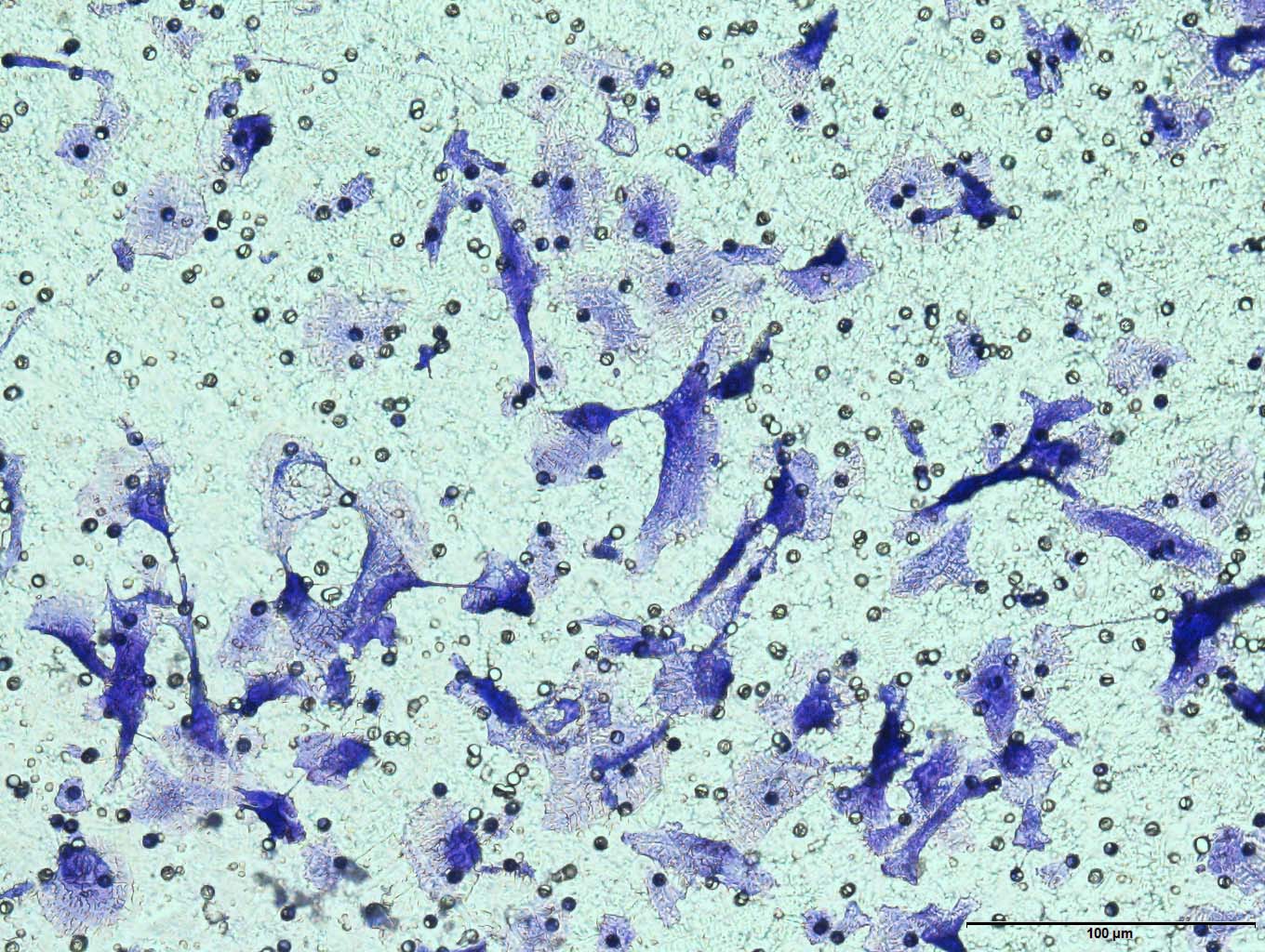

Supplement: S2 Fig — (ZIP) [file pone.0195844.s002.zip › S2 Appendix/S2_Fig2B-Mig-786O leVDRí┴200 (2).jpg]

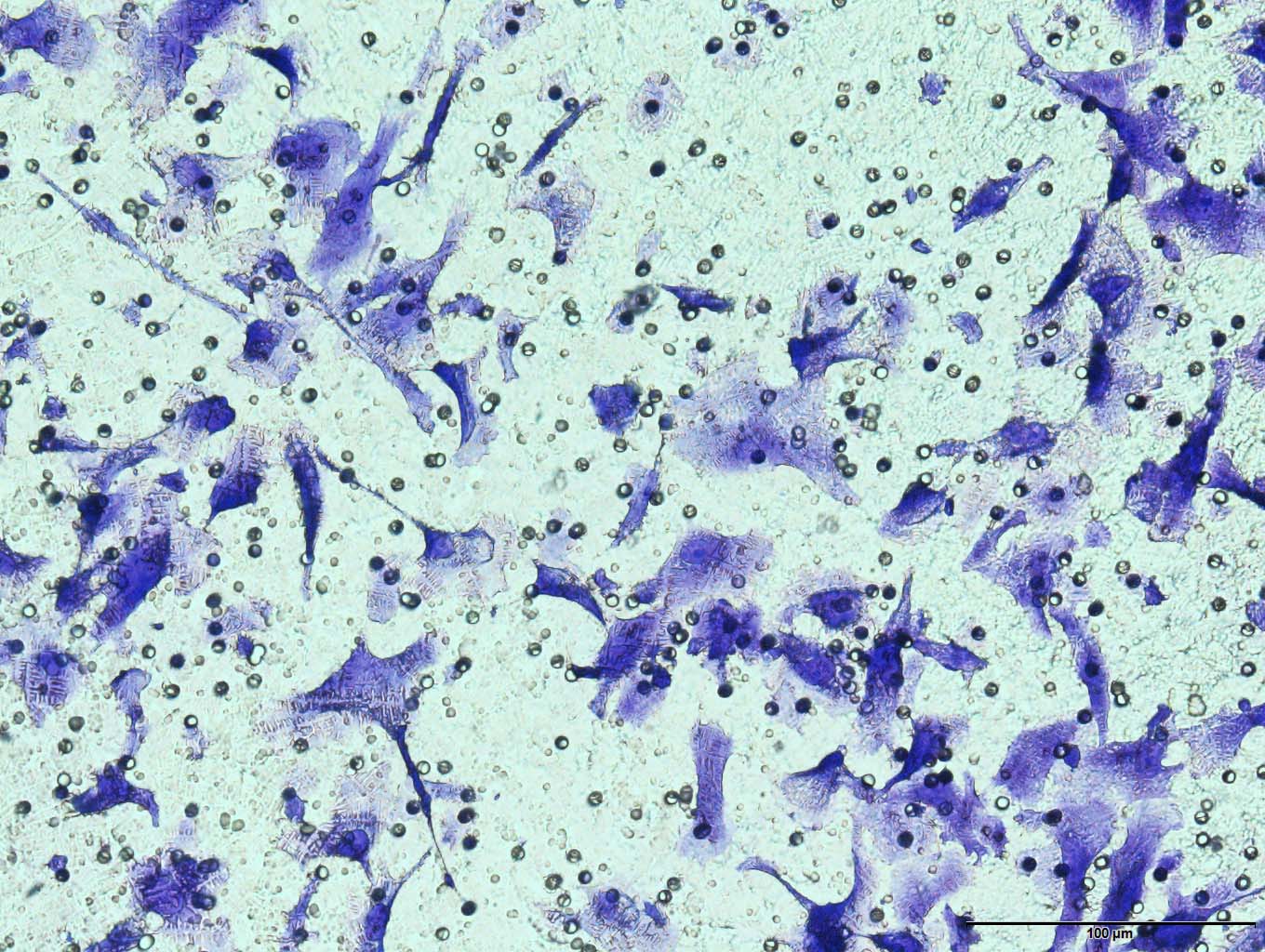

Supplement: S2 Fig — (ZIP) [file pone.0195844.s002.zip › S2 Appendix/S2_Fig2B-Mig-786O leVDRí┴200 (3).jpg]

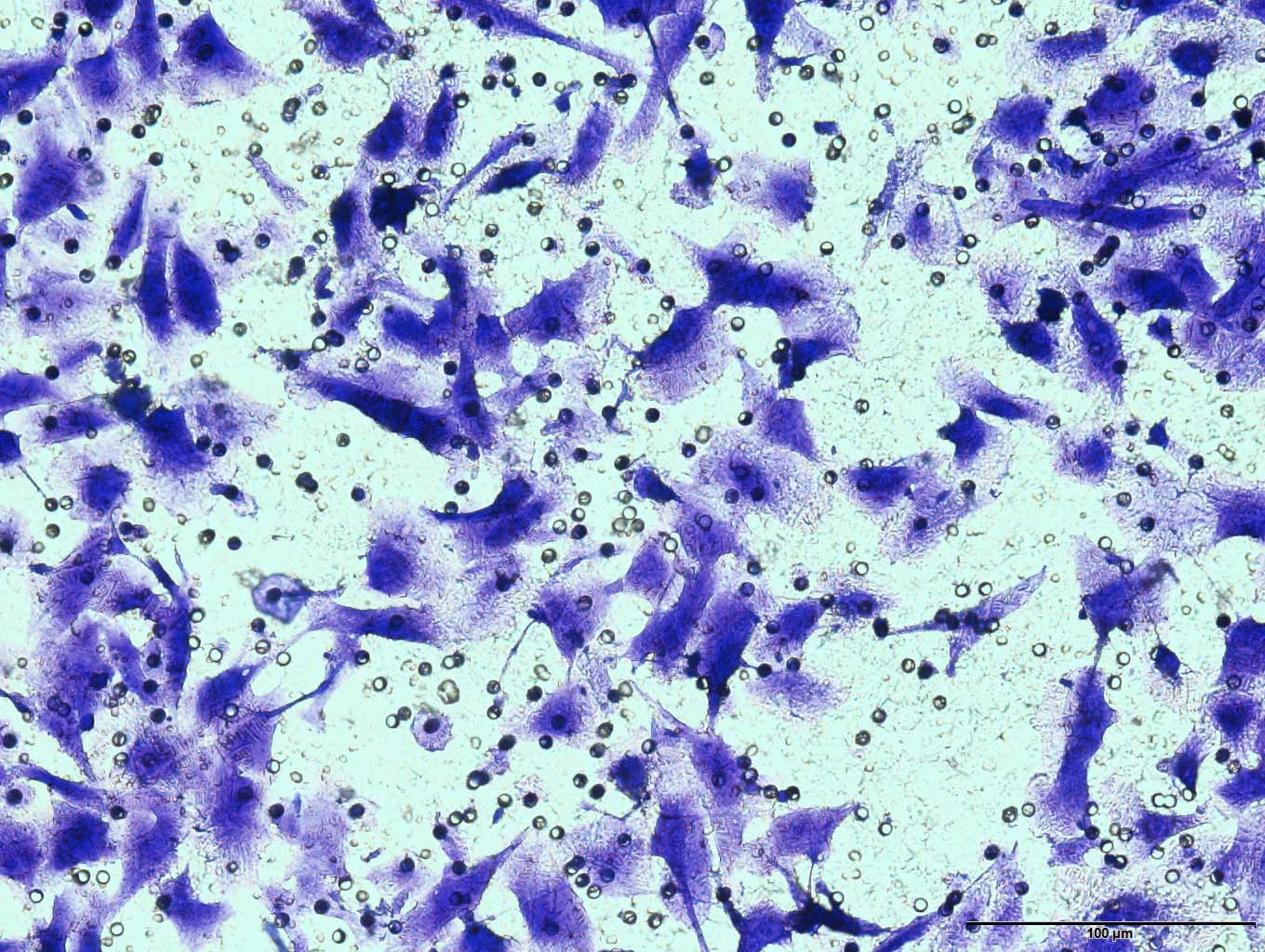

Supplement: S2 Fig — (ZIP) [file pone.0195844.s002.zip › S2 Appendix/S2_Fig2B-Mig-786O leVDRí┴200 (4).jpg]

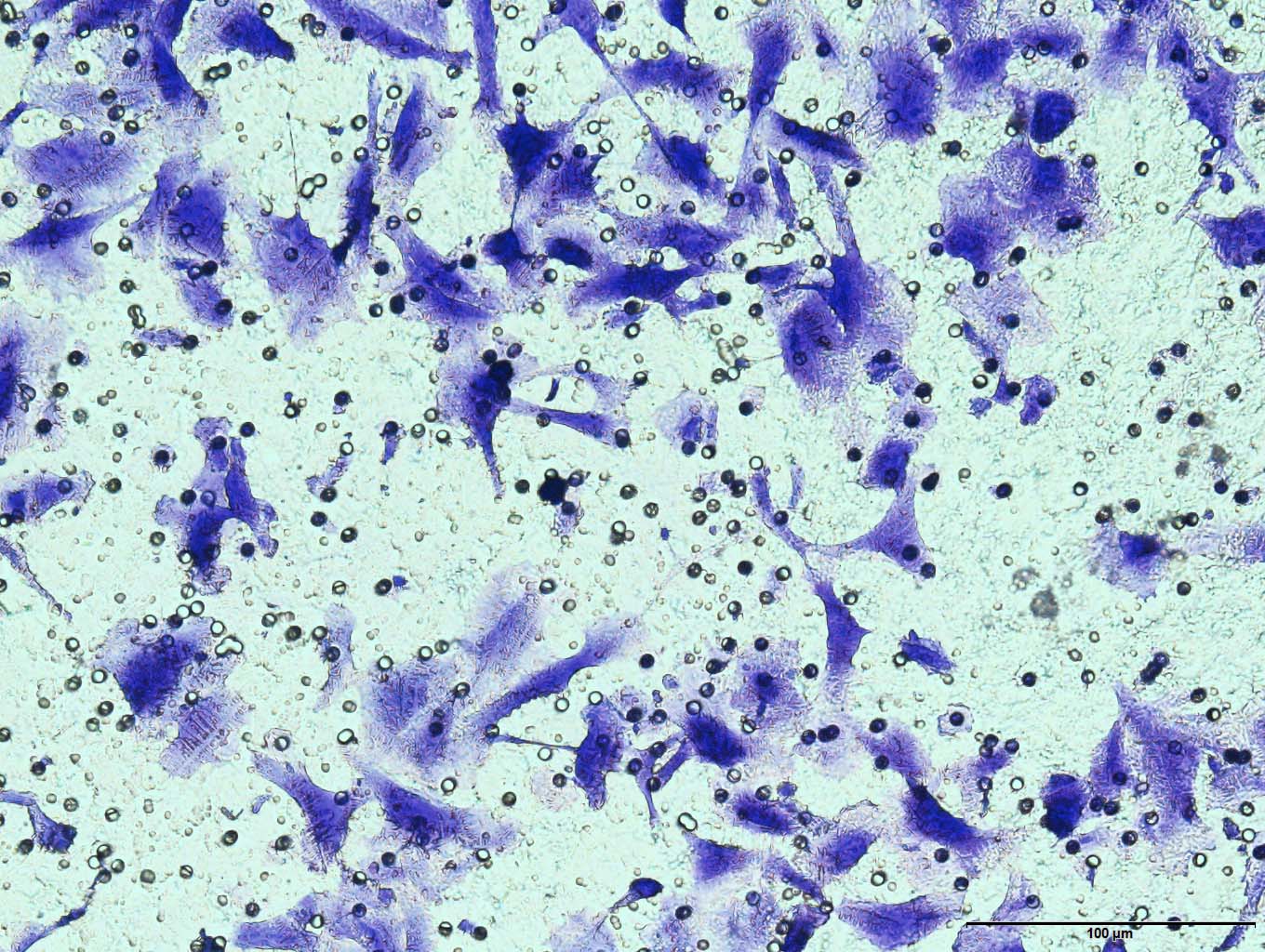

Supplement: S2 Fig — (ZIP) [file pone.0195844.s002.zip › S2 Appendix/S2_Fig2B-Mig-786O leVDRí┴200 (5).jpg]

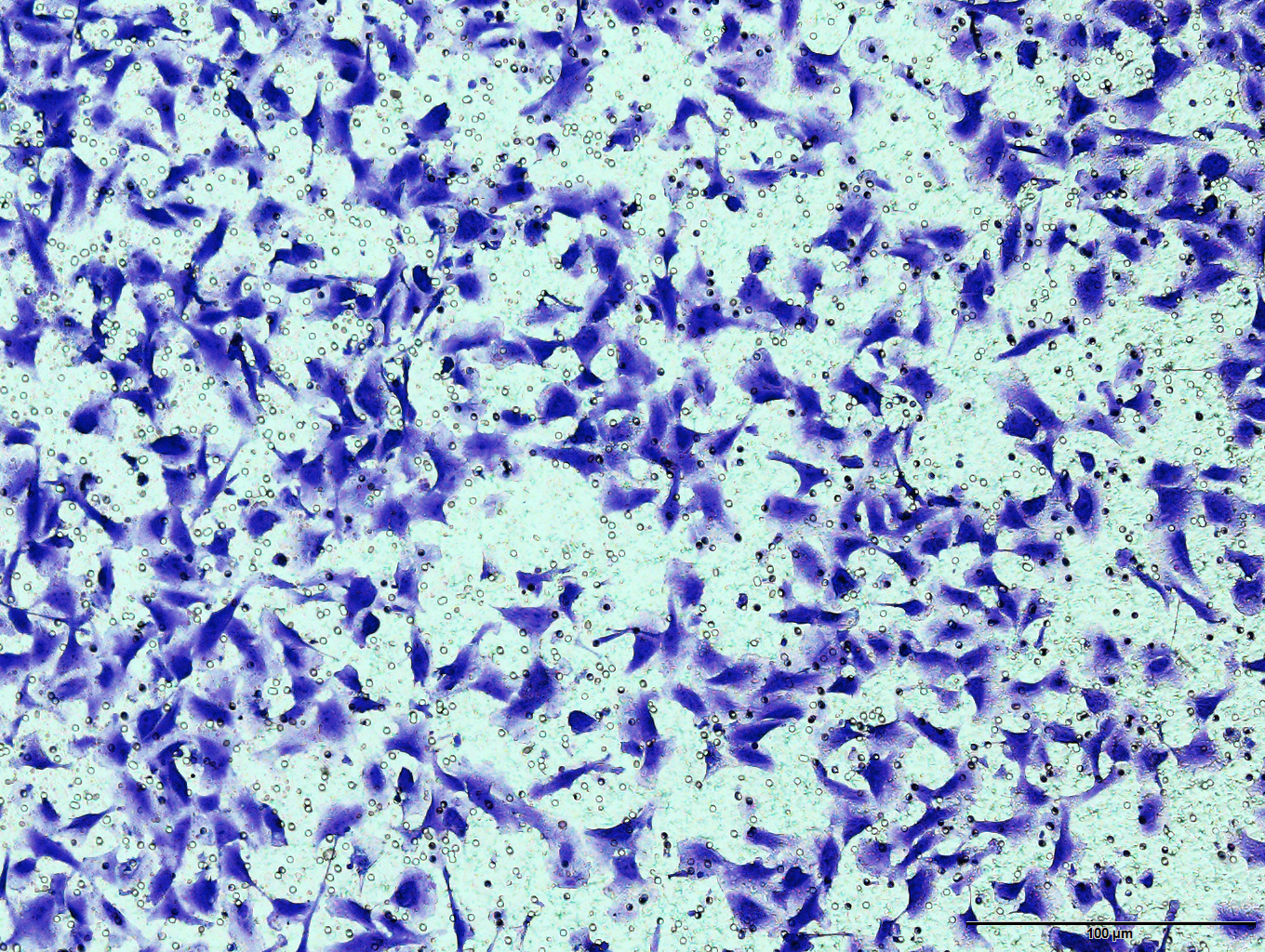

Supplement: S2 Fig — (ZIP) [file pone.0195844.s002.zip › S2 Appendix/S2_Fig2B-Mig-786O shCtrl í┴100.tif]

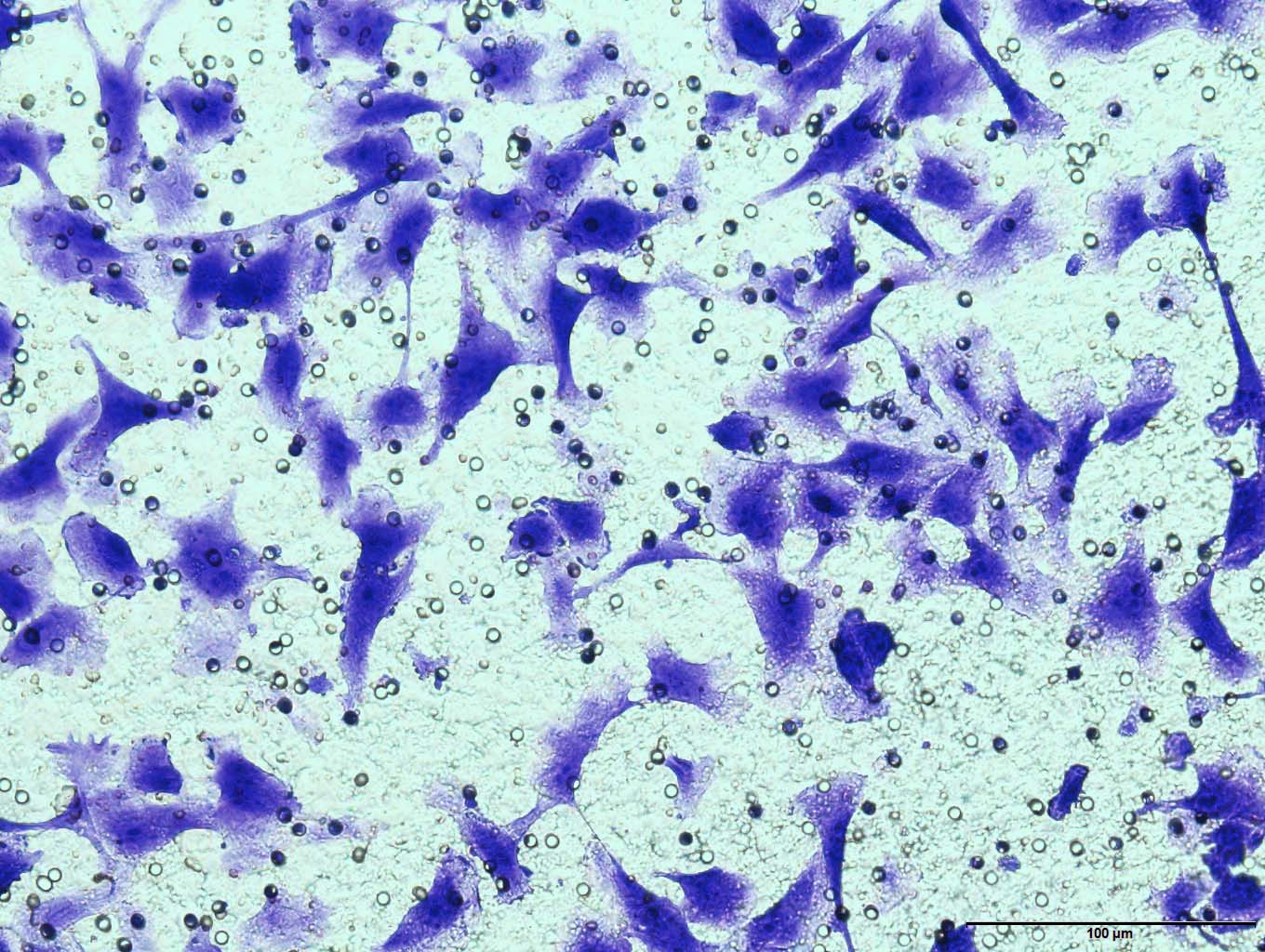

Supplement: S2 Fig — (ZIP) [file pone.0195844.s002.zip › S2 Appendix/S2_Fig2B-Mig-786O shCtrl í┴200 (1).jpg]

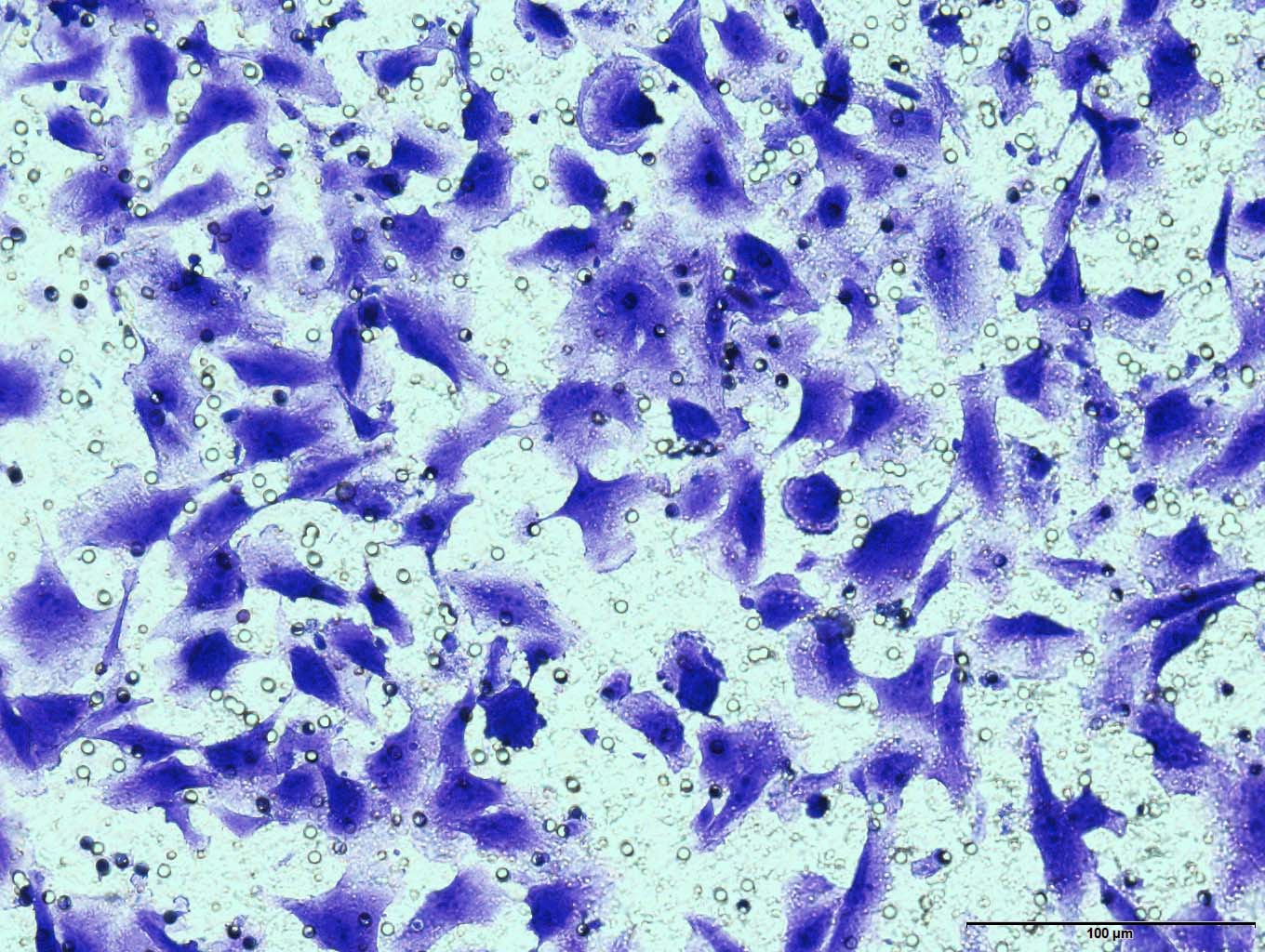

Supplement: S2 Fig — (ZIP) [file pone.0195844.s002.zip › S2 Appendix/S2_Fig2B-Mig-786O shCtrl í┴200 (2).jpg]

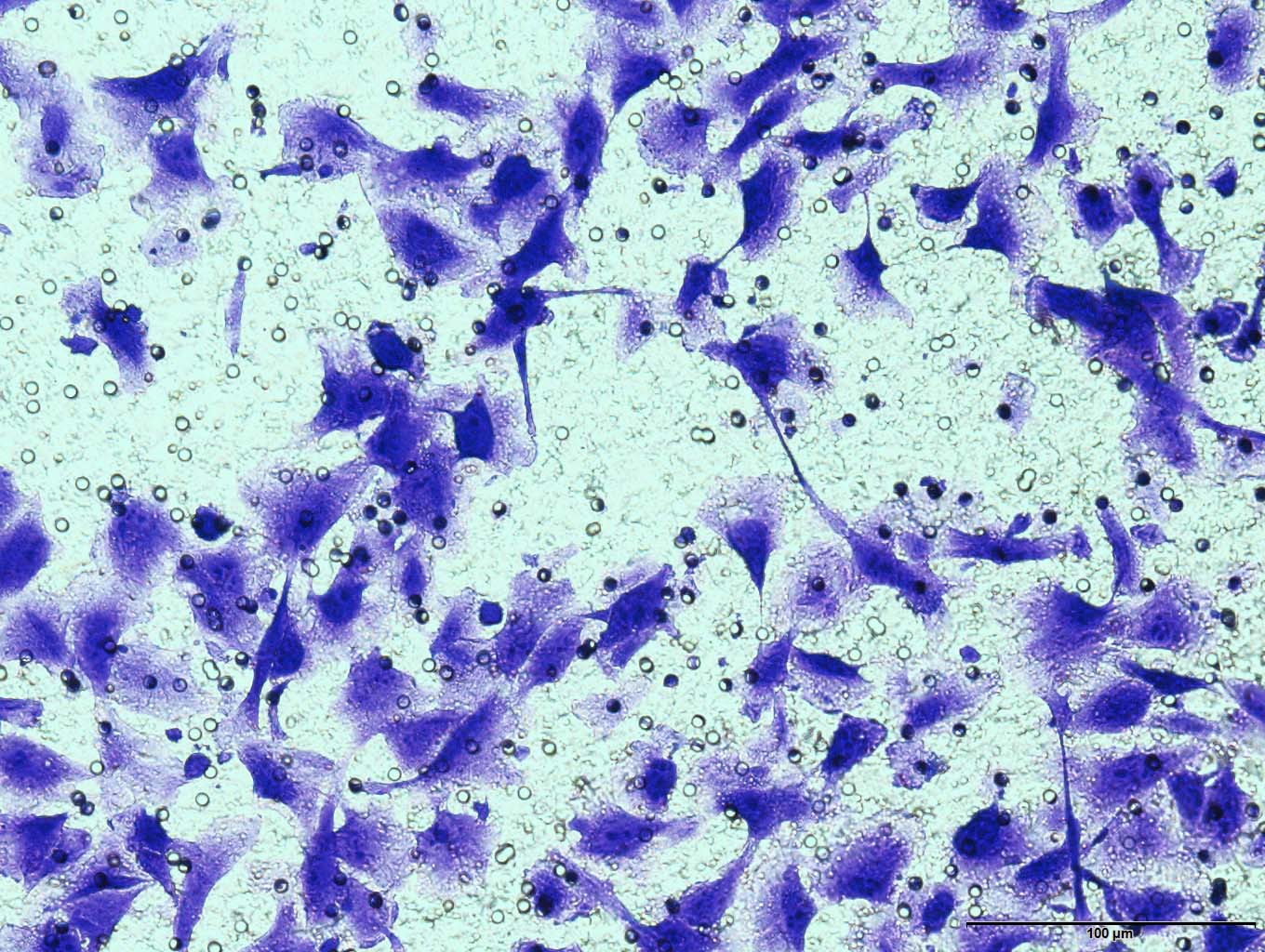

Supplement: S2 Fig — (ZIP) [file pone.0195844.s002.zip › S2 Appendix/S2_Fig2B-Mig-786O shCtrl í┴200 (3).jpg]

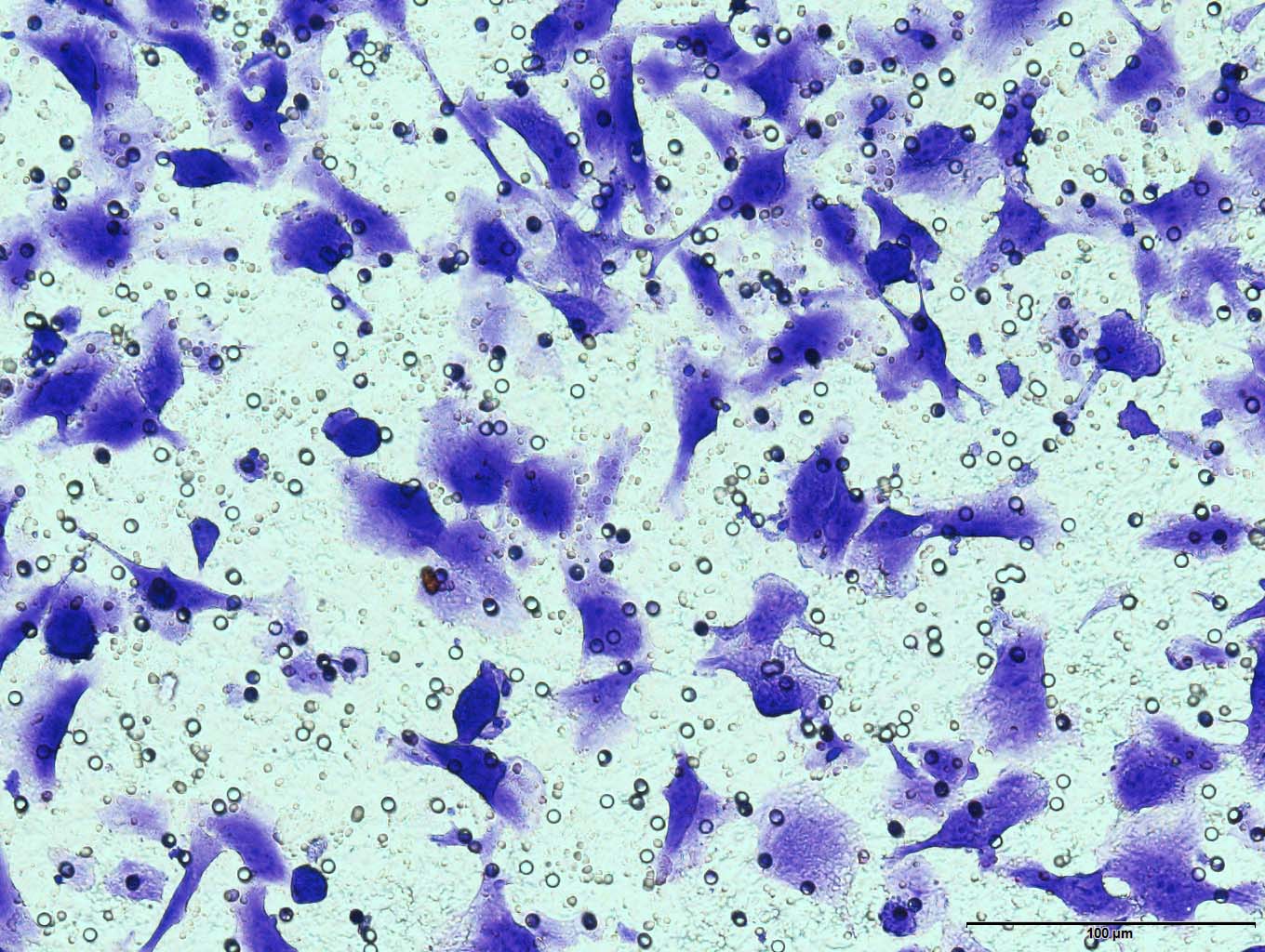

Supplement: S2 Fig — (ZIP) [file pone.0195844.s002.zip › S2 Appendix/S2_Fig2B-Mig-786O shCtrl í┴200 (4).jpg]

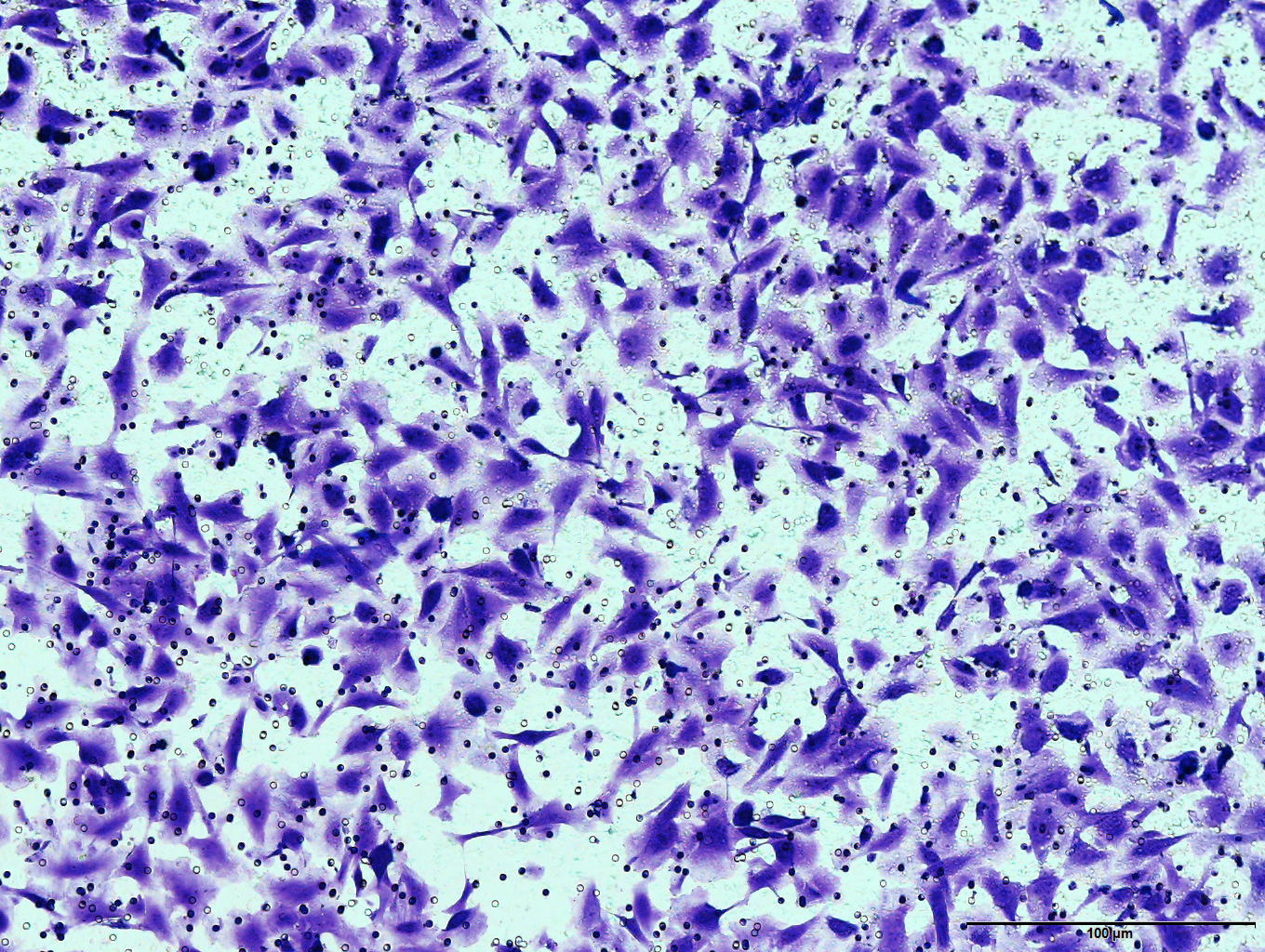

Supplement: S2 Fig — (ZIP) [file pone.0195844.s002.zip › S2 Appendix/S2_Fig2B-Mig-786O shVDR í┴100.tif]

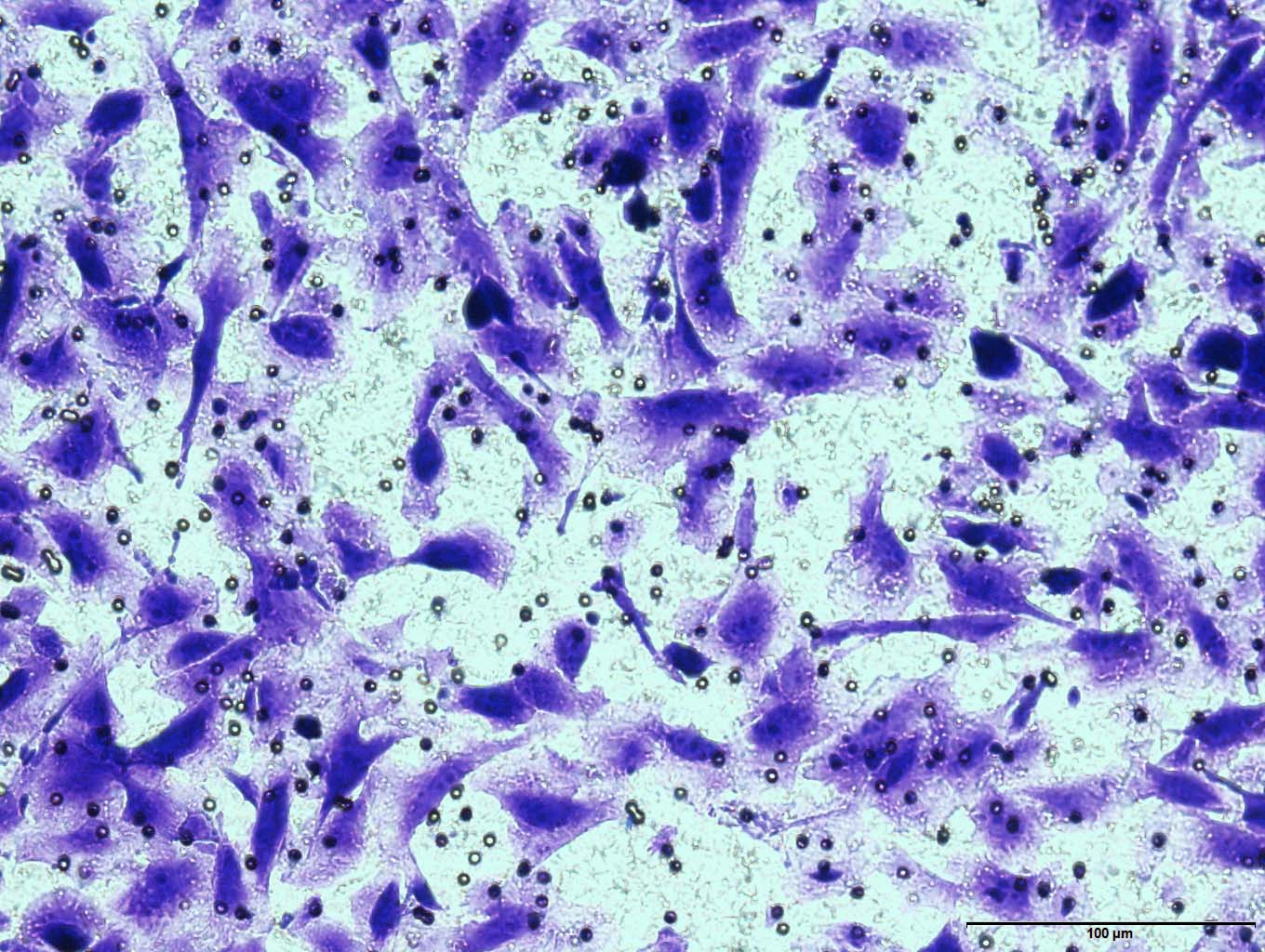

Supplement: S2 Fig — (ZIP) [file pone.0195844.s002.zip › S2 Appendix/S2_Fig2B-Mig-786O shVDR í┴200 (1).jpg]

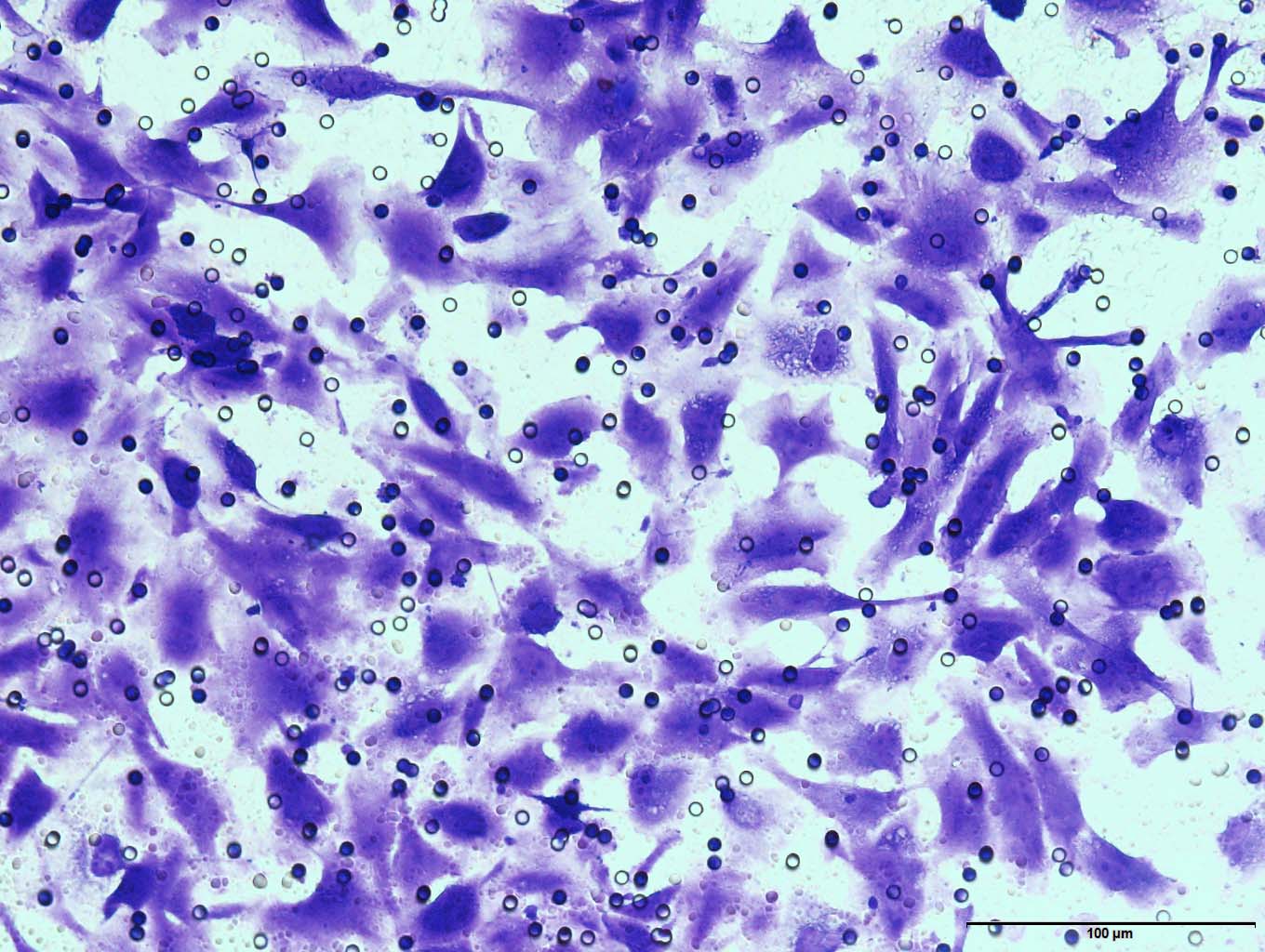

Supplement: S2 Fig — (ZIP) [file pone.0195844.s002.zip › S2 Appendix/S2_Fig2B-Mig-786O shVDR í┴200 (2).jpg]

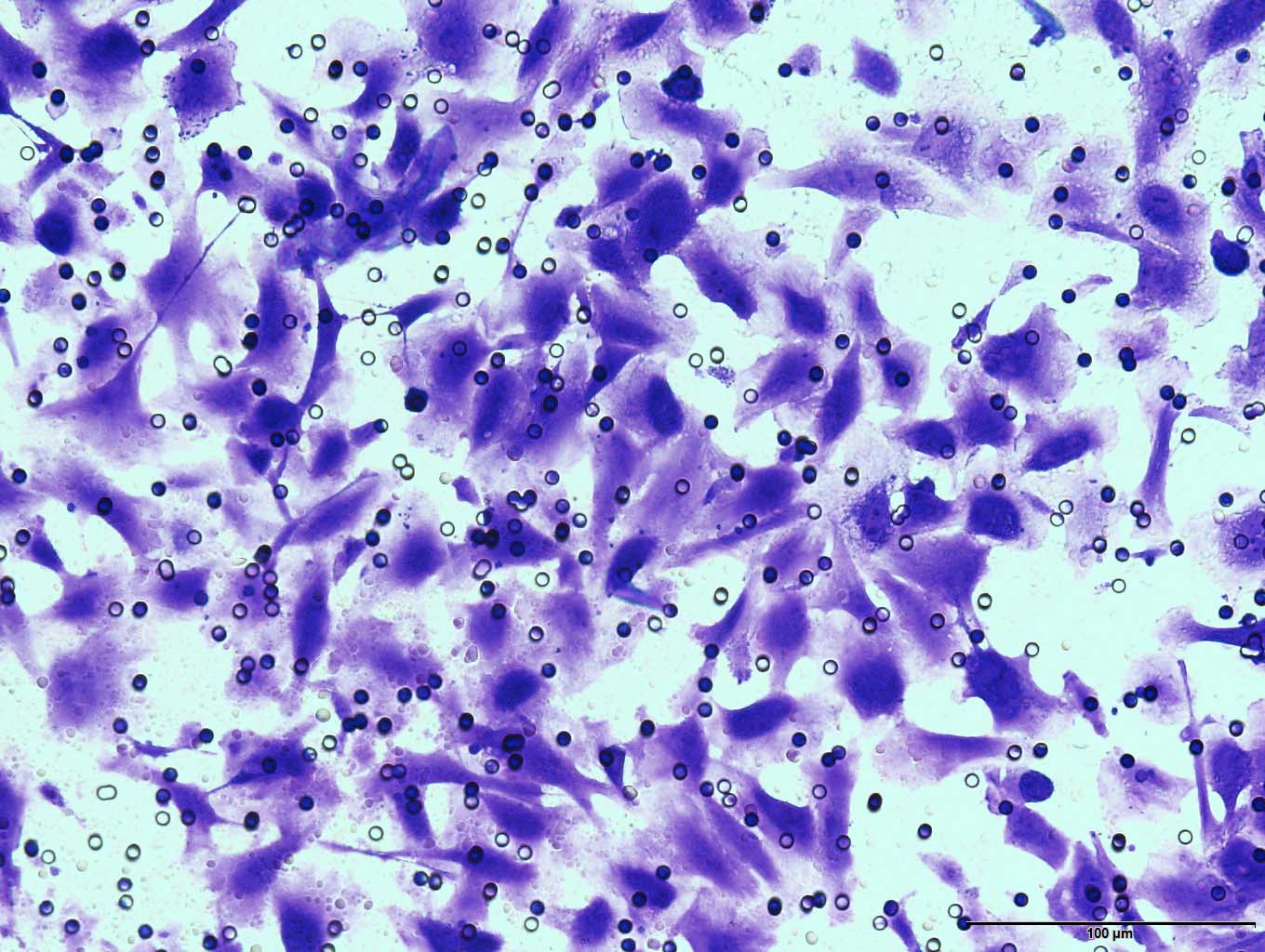

Supplement: S2 Fig — (ZIP) [file pone.0195844.s002.zip › S2 Appendix/S2_Fig2B-Mig-786O shVDR í┴200 (3).jpg]

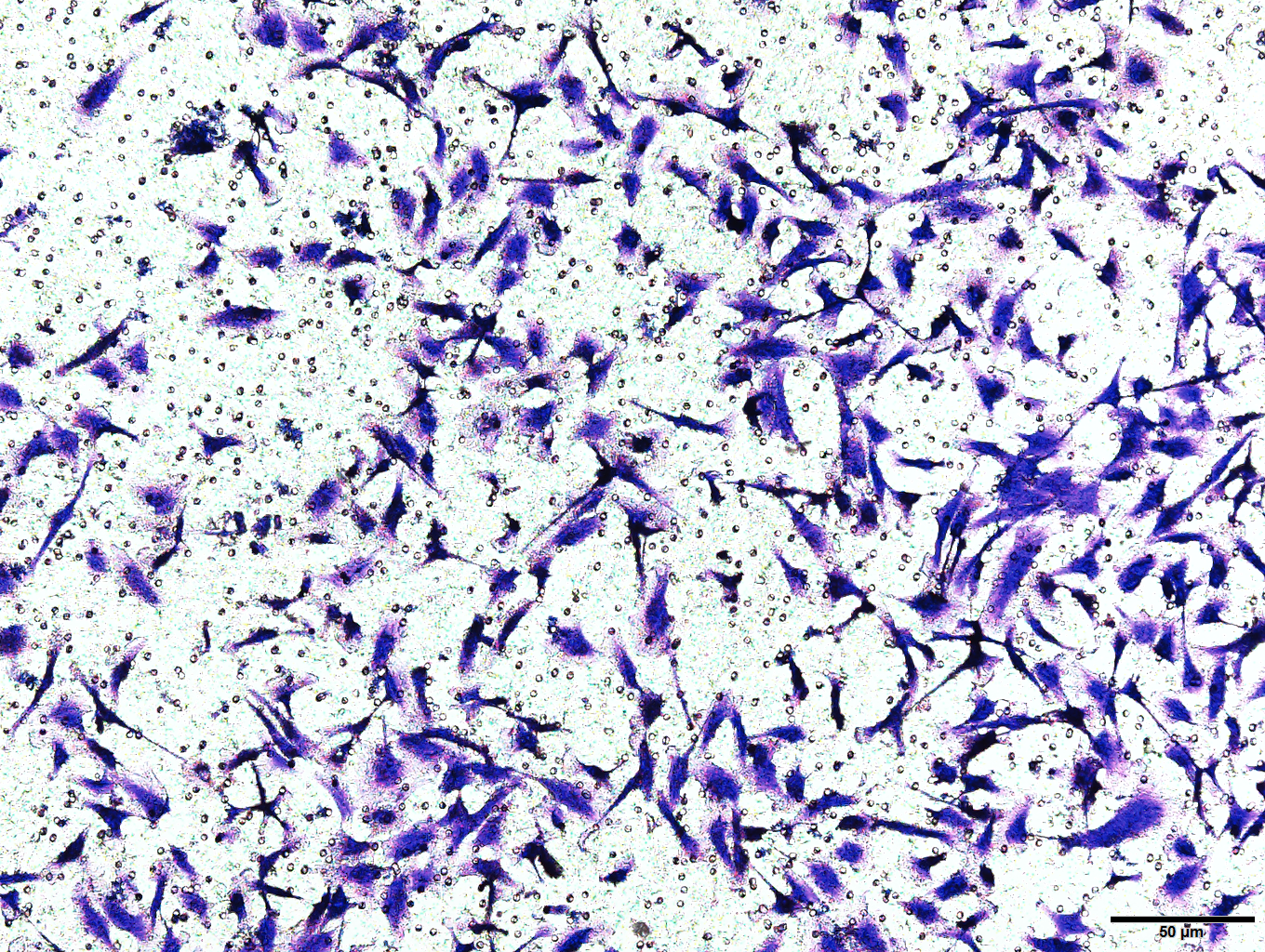

Supplement: S2 Fig — (ZIP) [file pone.0195844.s002.zip › S2 Appendix/S2_Fig2c-Inv-786O leCtrl í┴100.tif]

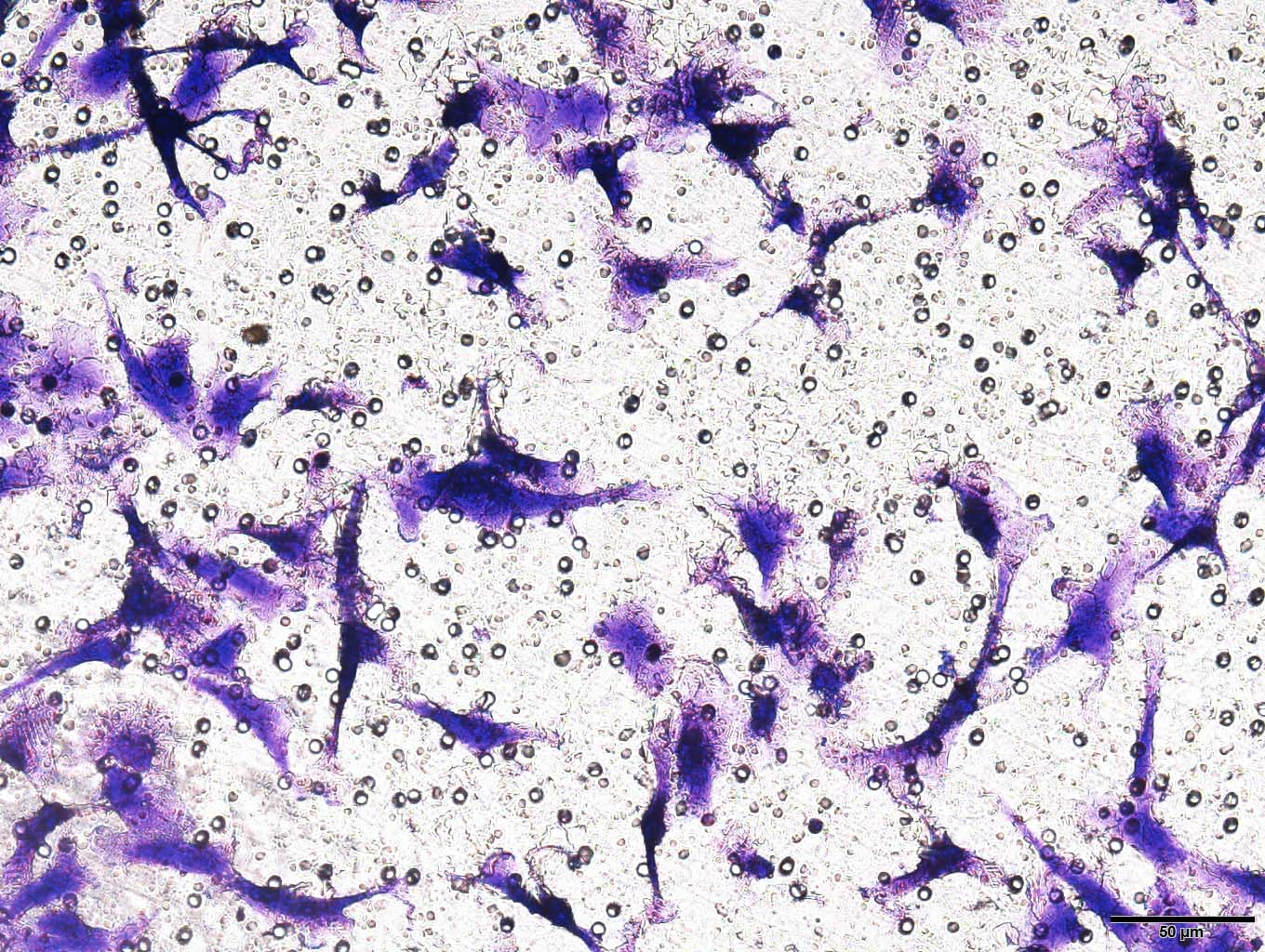

Supplement: S2 Fig — (ZIP) [file pone.0195844.s002.zip › S2 Appendix/S2_Fig2c-Inv-786O leCtrl í┴200 (1).jpg]

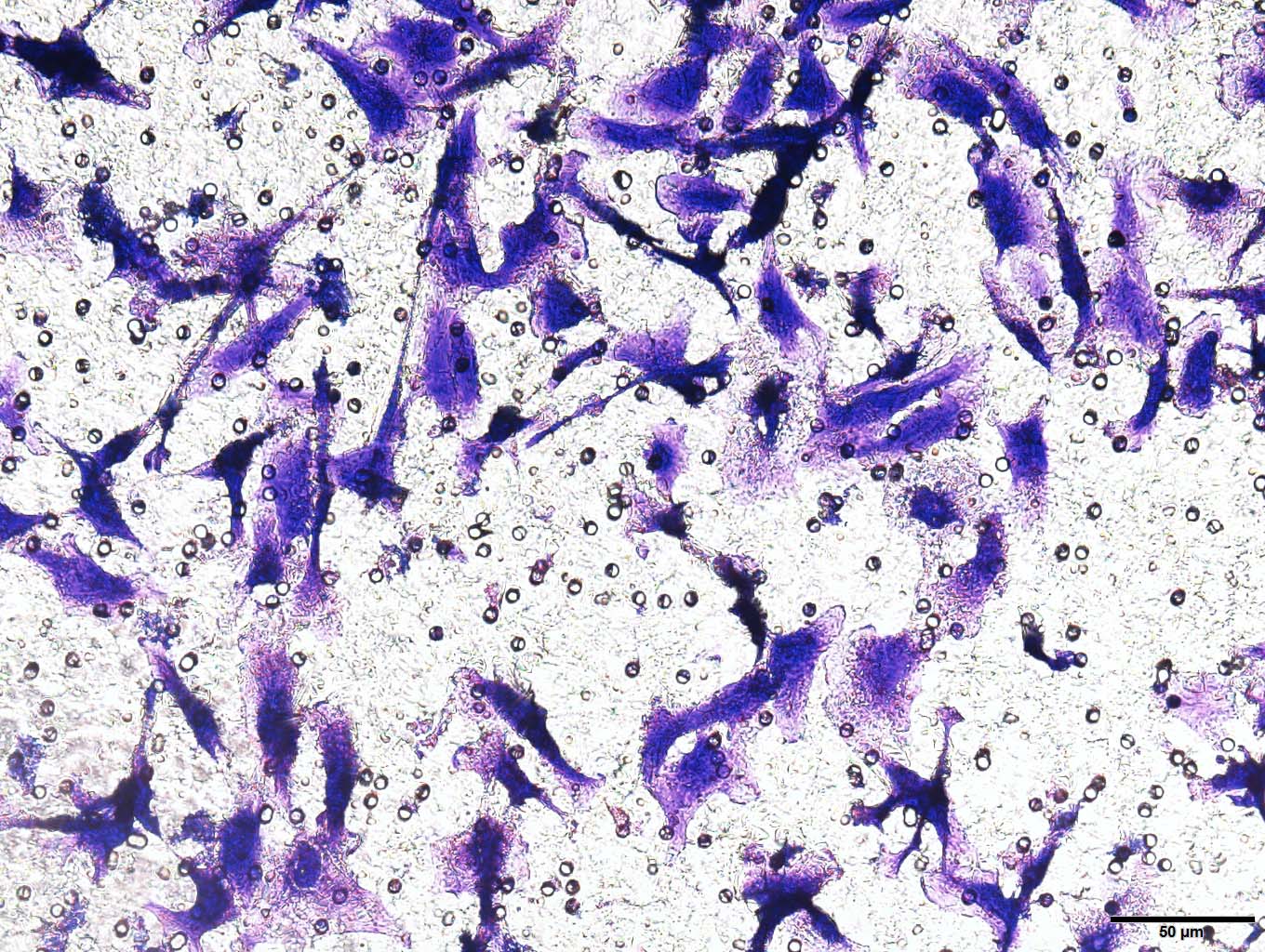

Supplement: S2 Fig — (ZIP) [file pone.0195844.s002.zip › S2 Appendix/S2_Fig2c-Inv-786O leCtrl í┴200 (2).jpg]

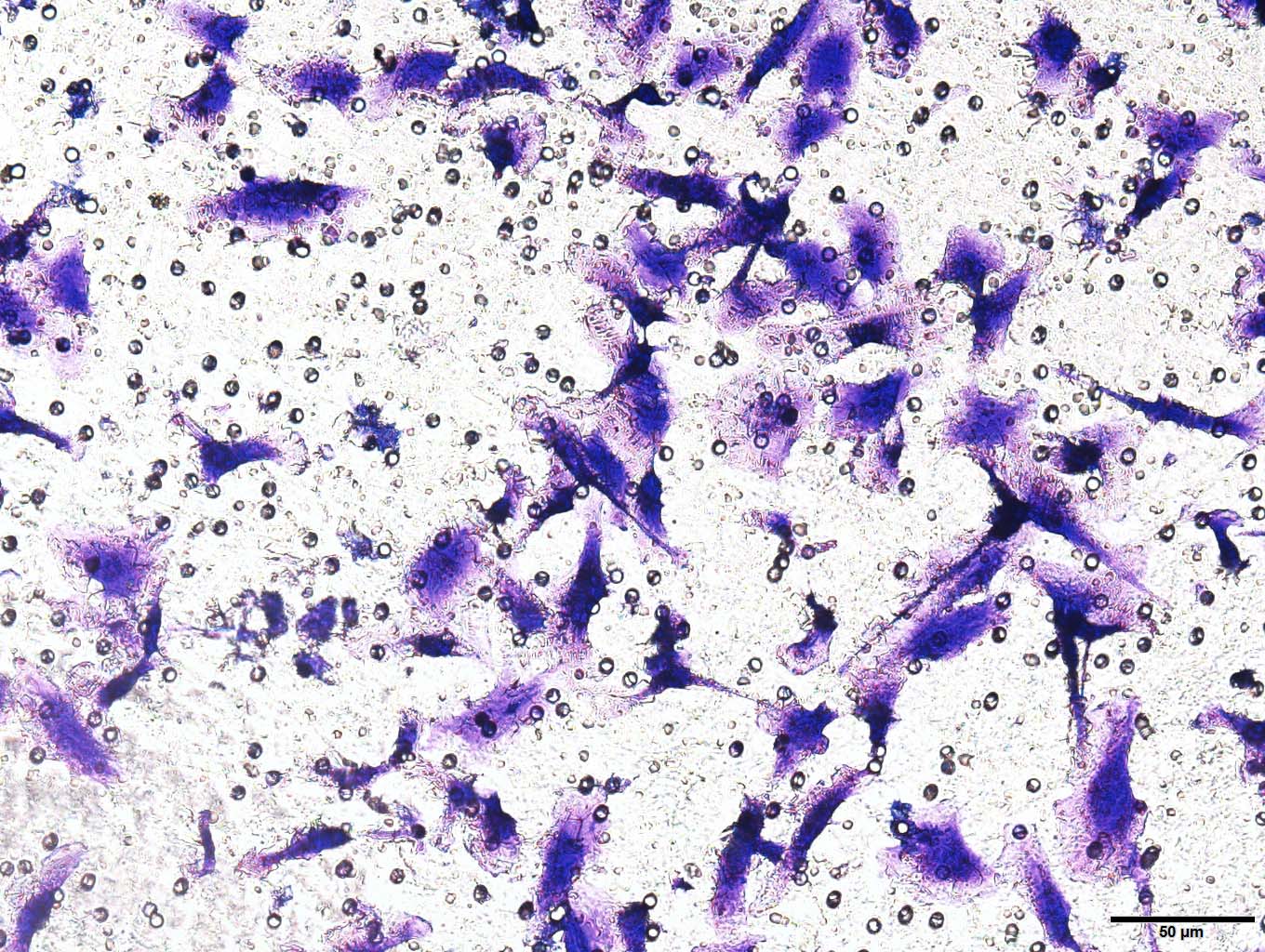

Supplement: S2 Fig — (ZIP) [file pone.0195844.s002.zip › S2 Appendix/S2_Fig2c-Inv-786O leCtrl í┴200 (3).jpg]

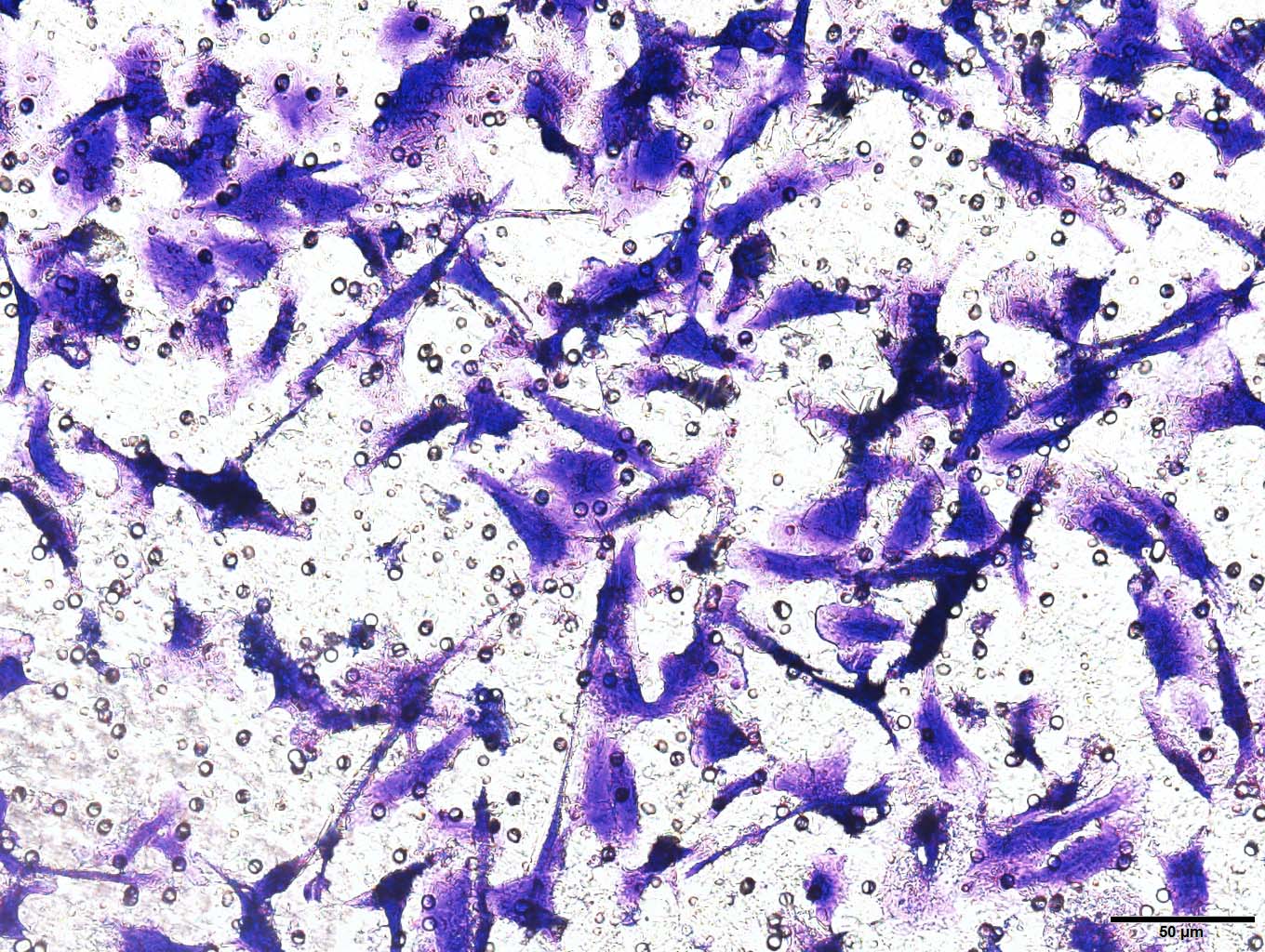

Supplement: S2 Fig — (ZIP) [file pone.0195844.s002.zip › S2 Appendix/S2_Fig2c-Inv-786O leCtrl í┴200 (4).jpg]

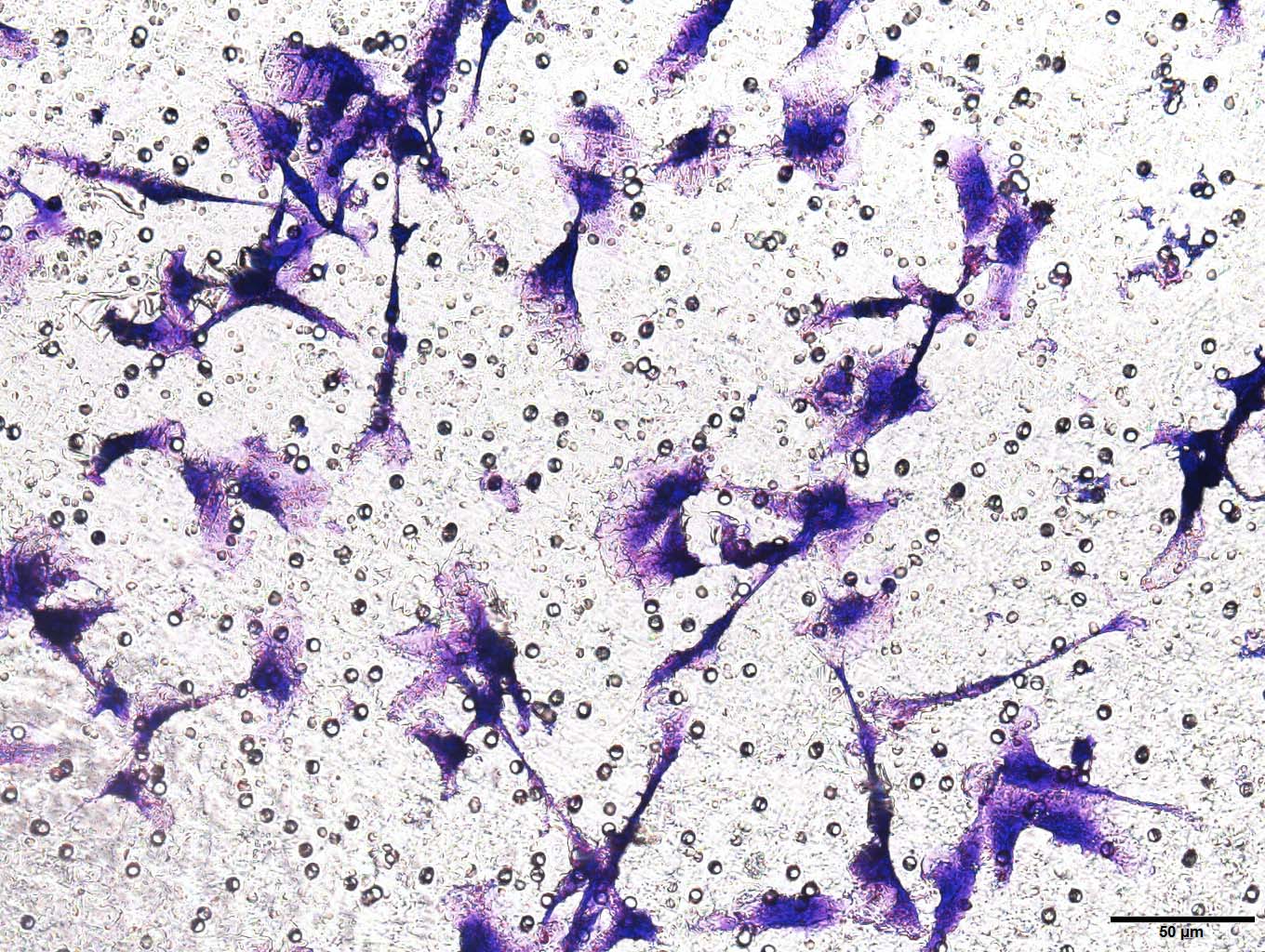

Supplement: S2 Fig — (ZIP) [file pone.0195844.s002.zip › S2 Appendix/S2_Fig2c-Inv-786O leCtrl í┴200 (5).jpg]

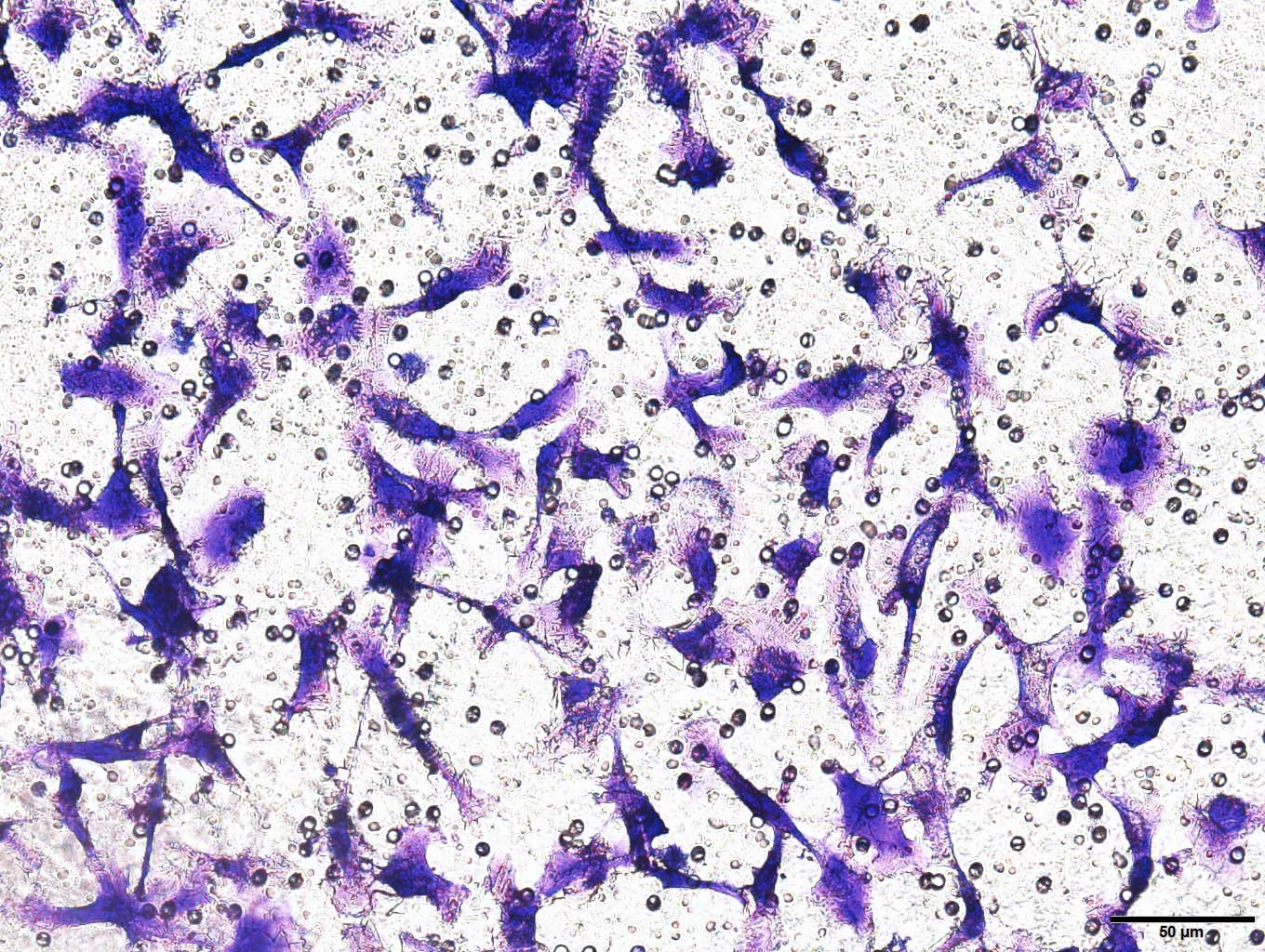

Supplement: S2 Fig — (ZIP) [file pone.0195844.s002.zip › S2 Appendix/S2_Fig2c-Inv-786O leCtrl í┴200 (6).jpg]

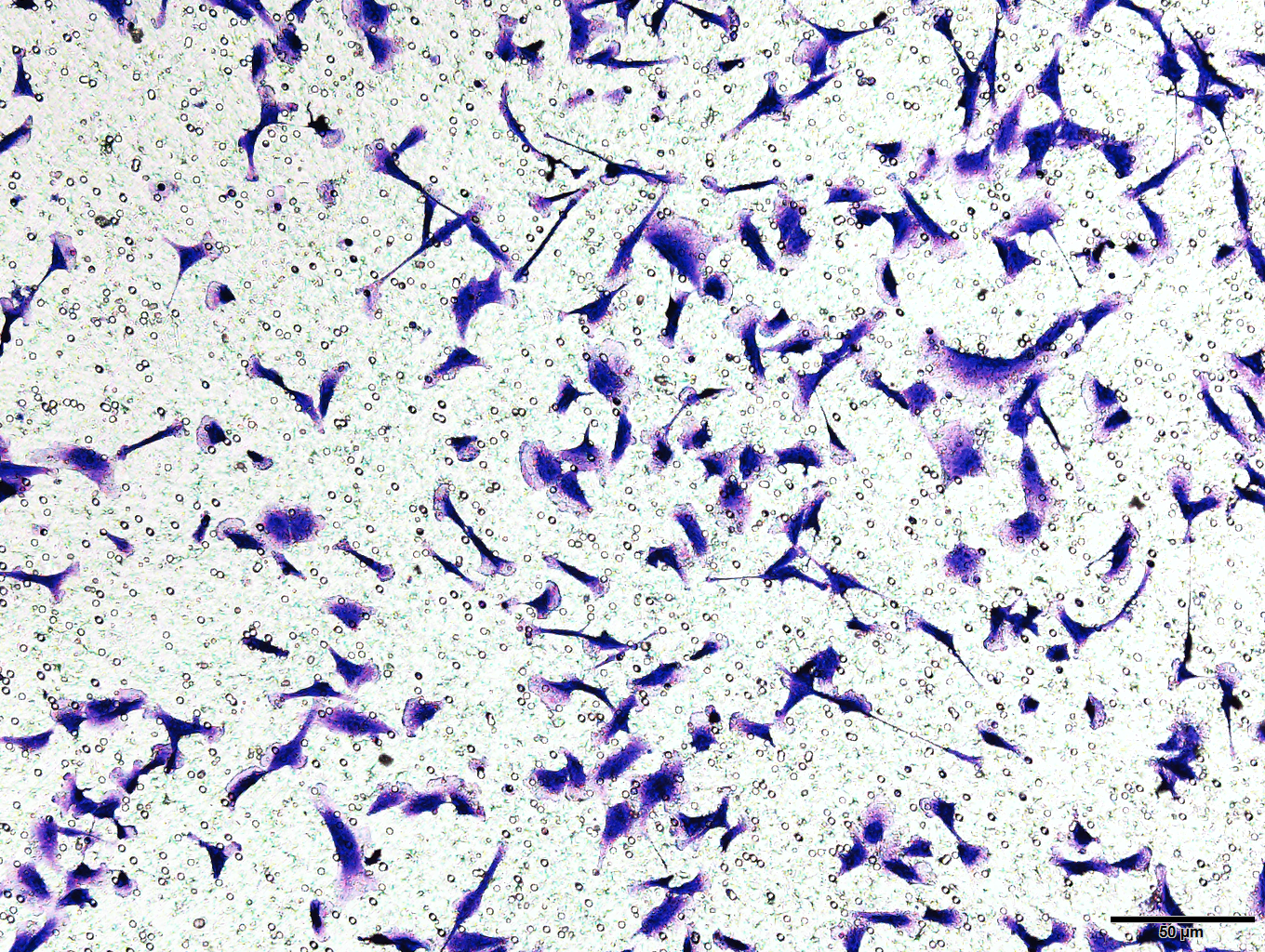

Supplement: S2 Fig — (ZIP) [file pone.0195844.s002.zip › S2 Appendix/S2_Fig2c-Inv-786O leVDR í┴100.tif]

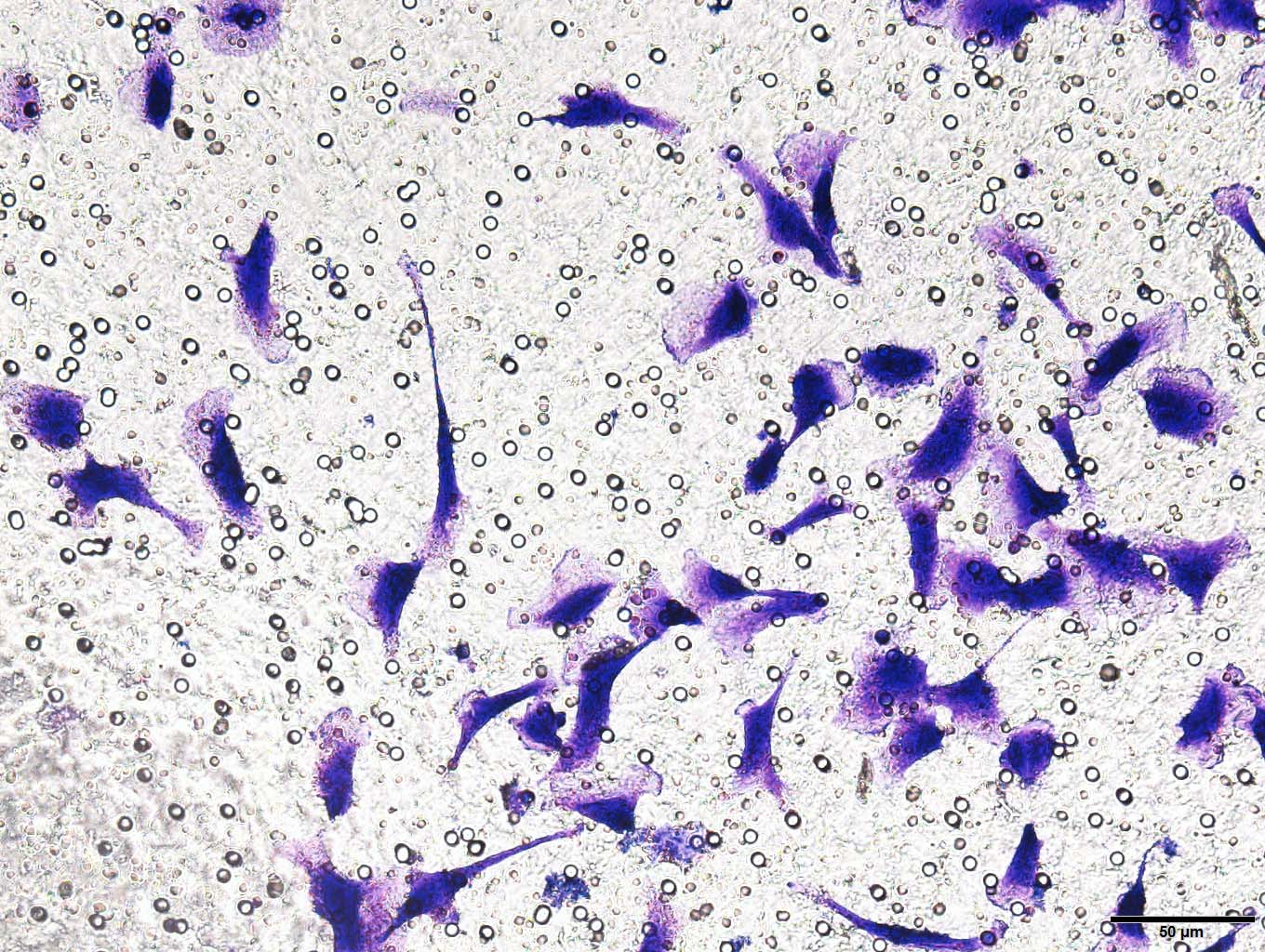

Supplement: S2 Fig — (ZIP) [file pone.0195844.s002.zip › S2 Appendix/S2_Fig2c-Inv-786O leVDR í┴200 (1).jpg]

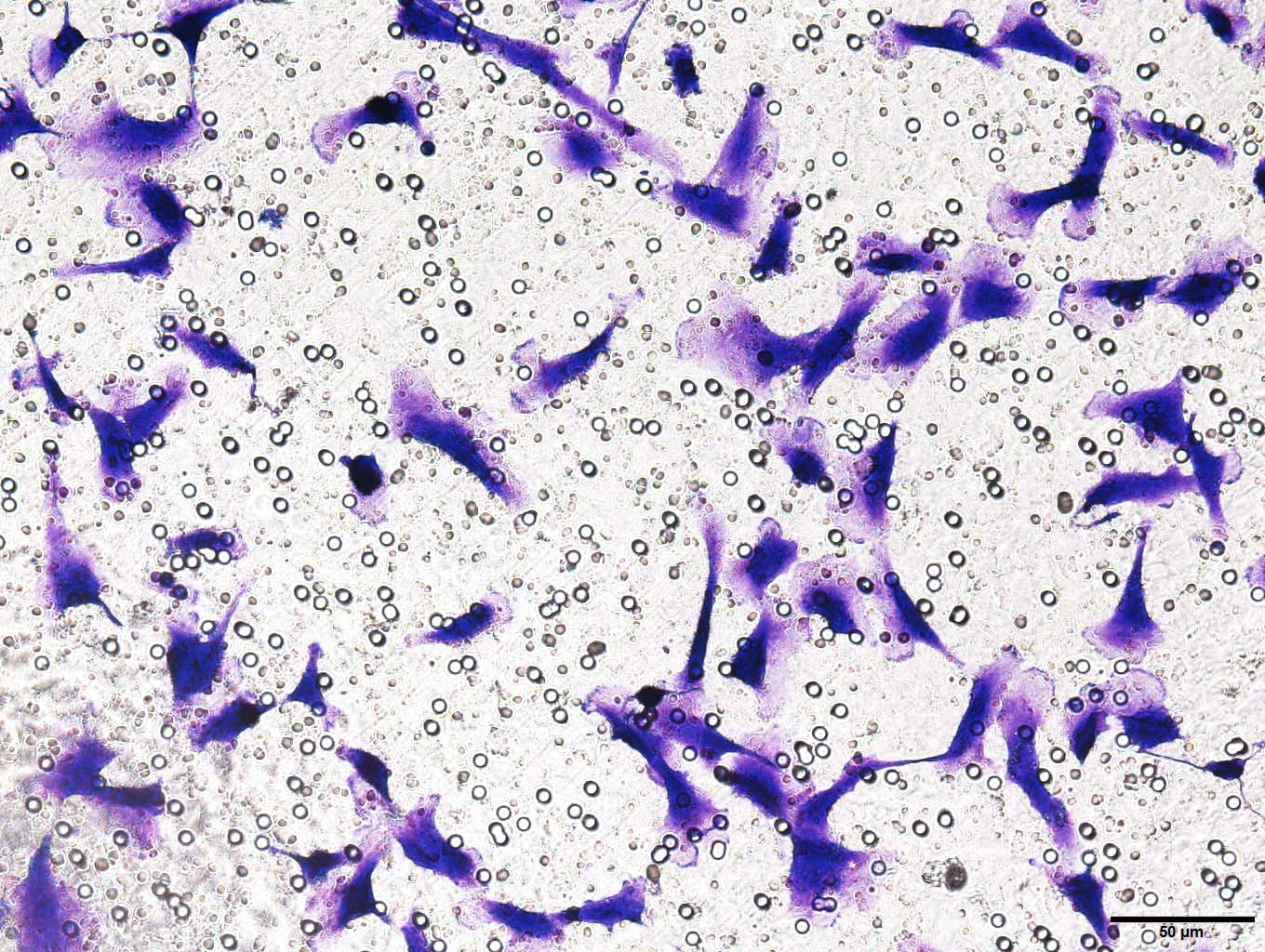

Supplement: S2 Fig — (ZIP) [file pone.0195844.s002.zip › S2 Appendix/S2_Fig2c-Inv-786O leVDR í┴200 (2).jpg]

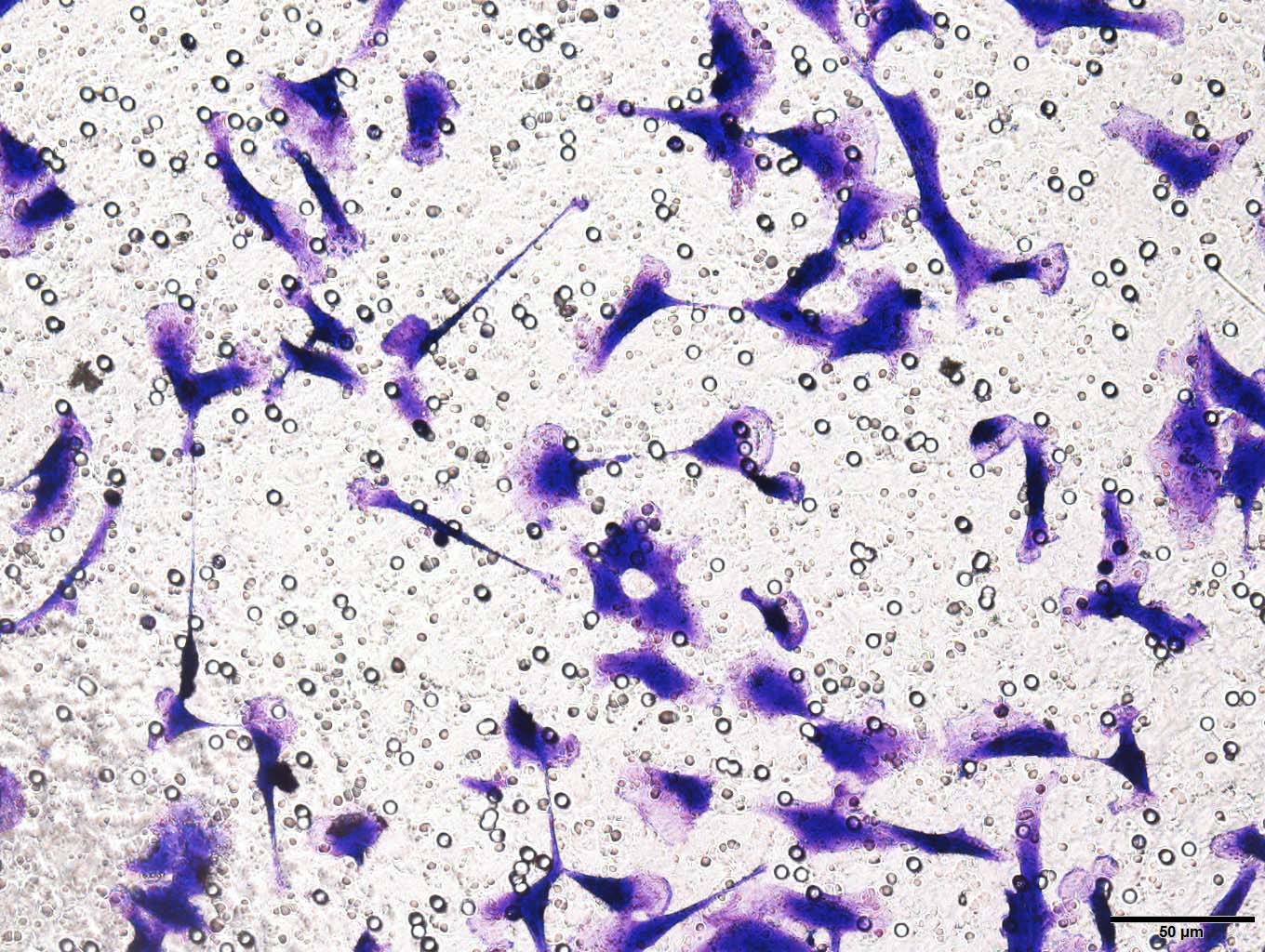

Supplement: S2 Fig — (ZIP) [file pone.0195844.s002.zip › S2 Appendix/S2_Fig2c-Inv-786O leVDR í┴200 (3).jpg]

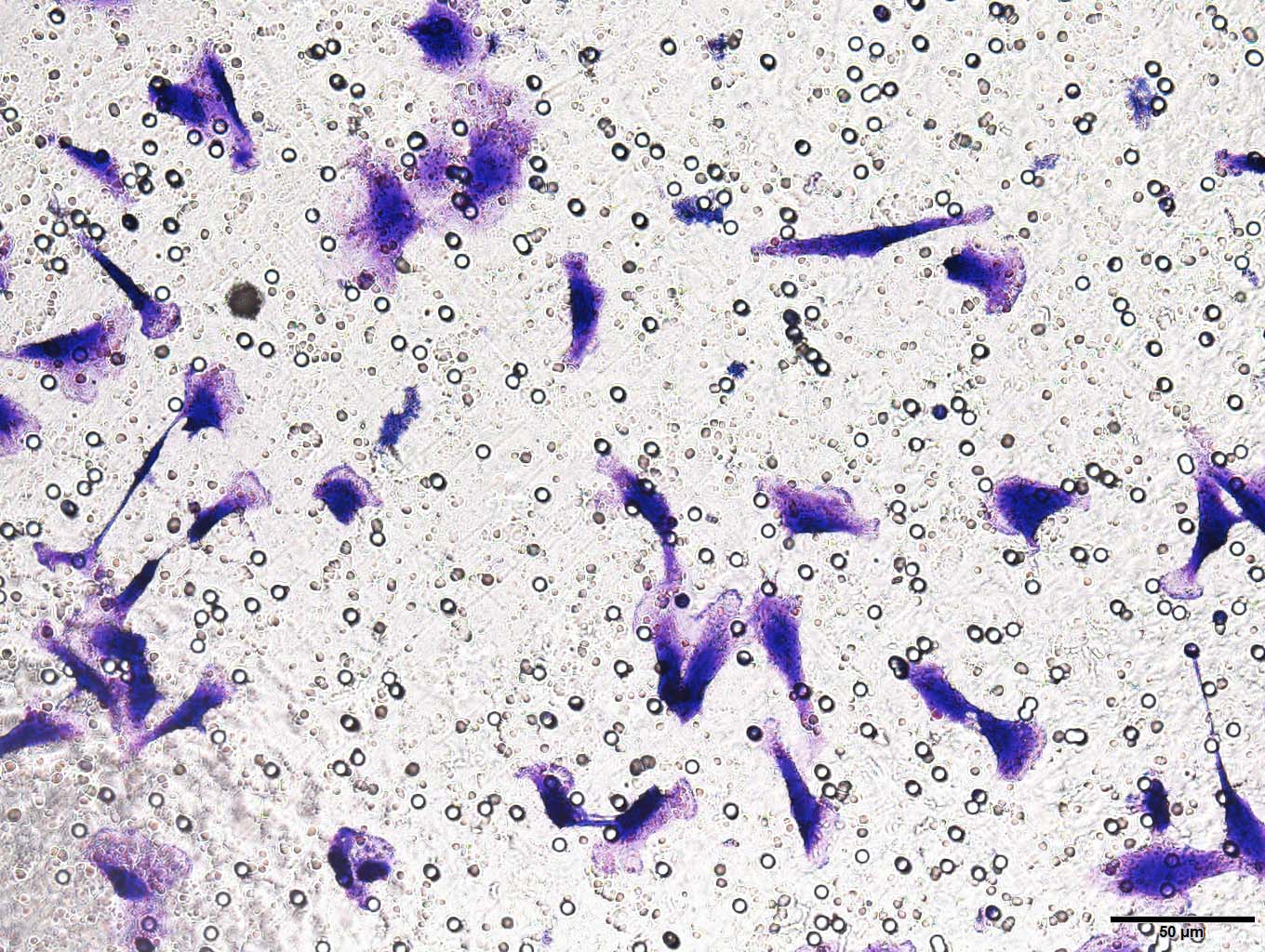

Supplement: S2 Fig — (ZIP) [file pone.0195844.s002.zip › S2 Appendix/S2_Fig2c-Inv-786O leVDR í┴200 (4).jpg]

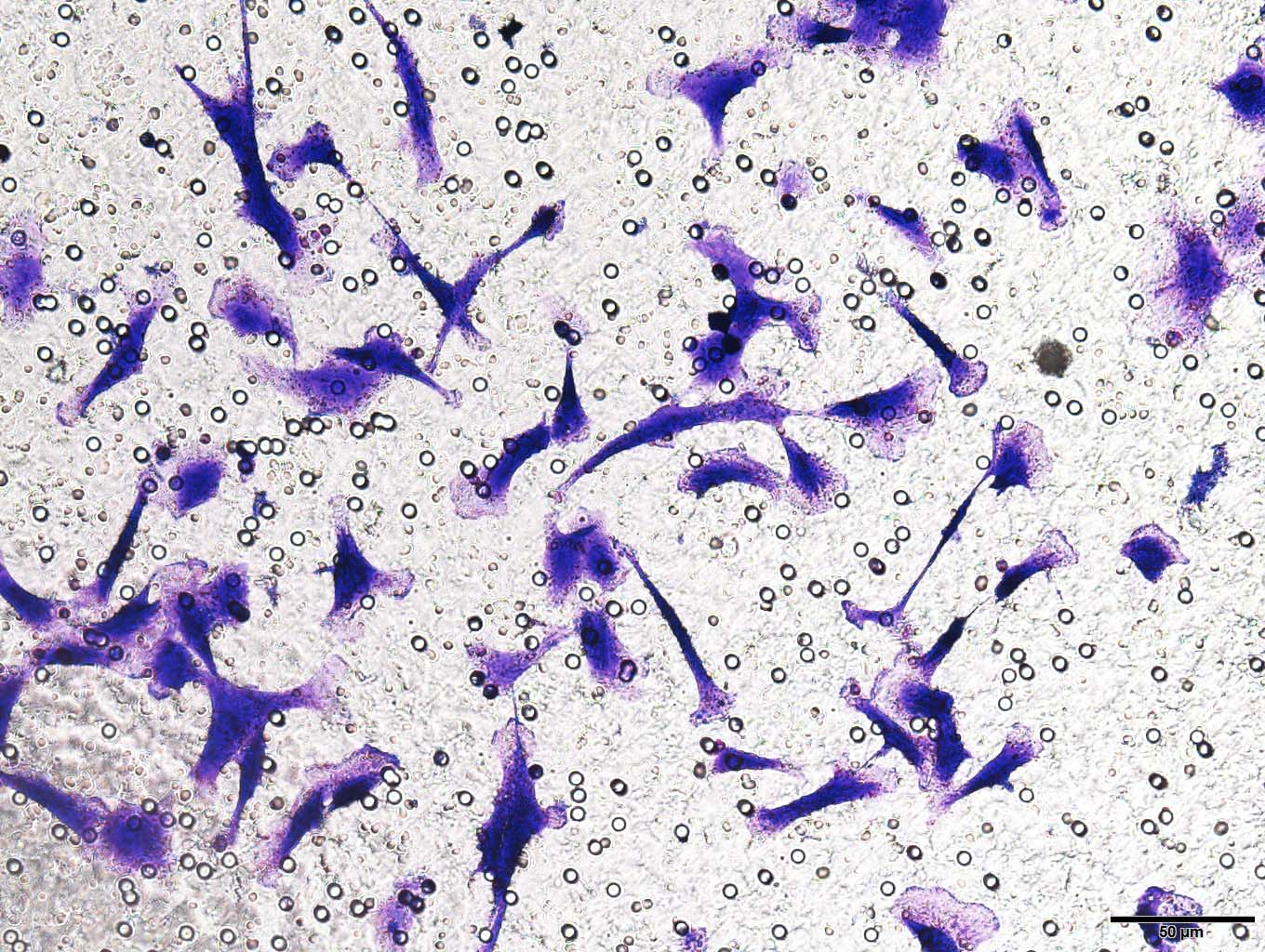

Supplement: S2 Fig — (ZIP) [file pone.0195844.s002.zip › S2 Appendix/S2_Fig2c-Inv-786O leVDR í┴200 (5).jpg]

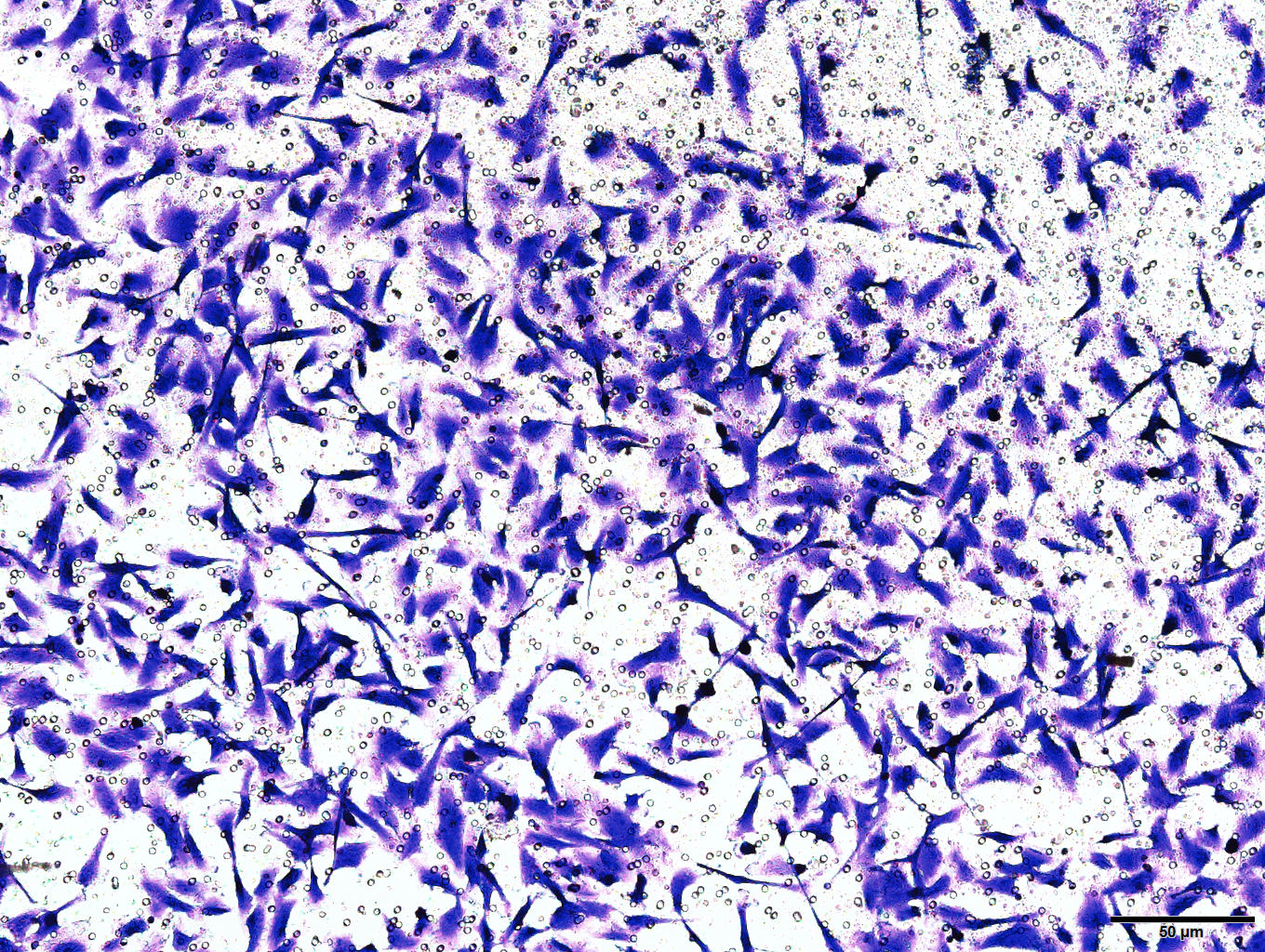

Supplement: S2 Fig — (ZIP) [file pone.0195844.s002.zip › S2 Appendix/S2_Fig2c-Inv-786O shCtrl í┴100.tif]

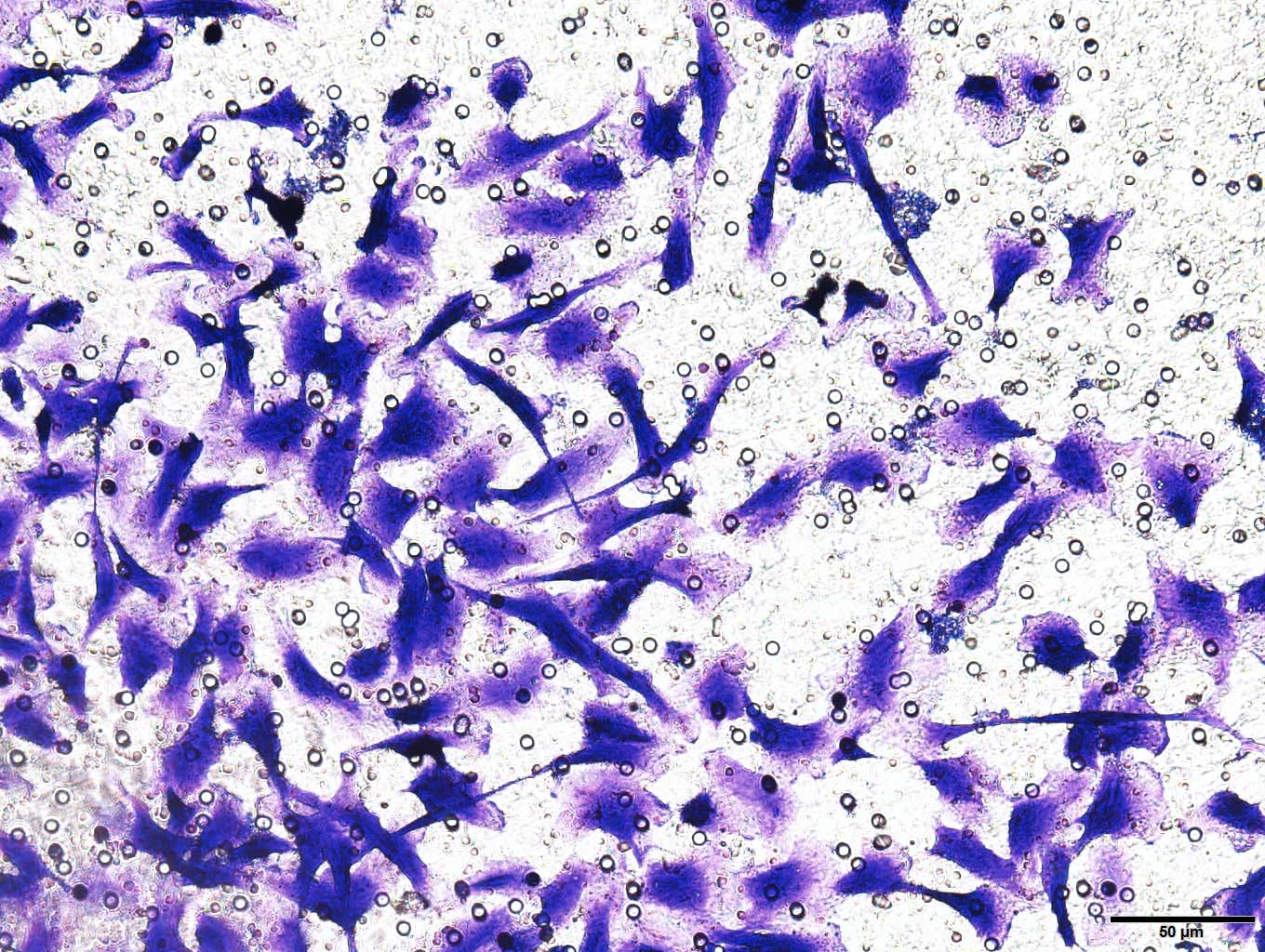

Supplement: S2 Fig — (ZIP) [file pone.0195844.s002.zip › S2 Appendix/S2_Fig2c-Inv-786O shCtrl í┴200 (1).jpg]

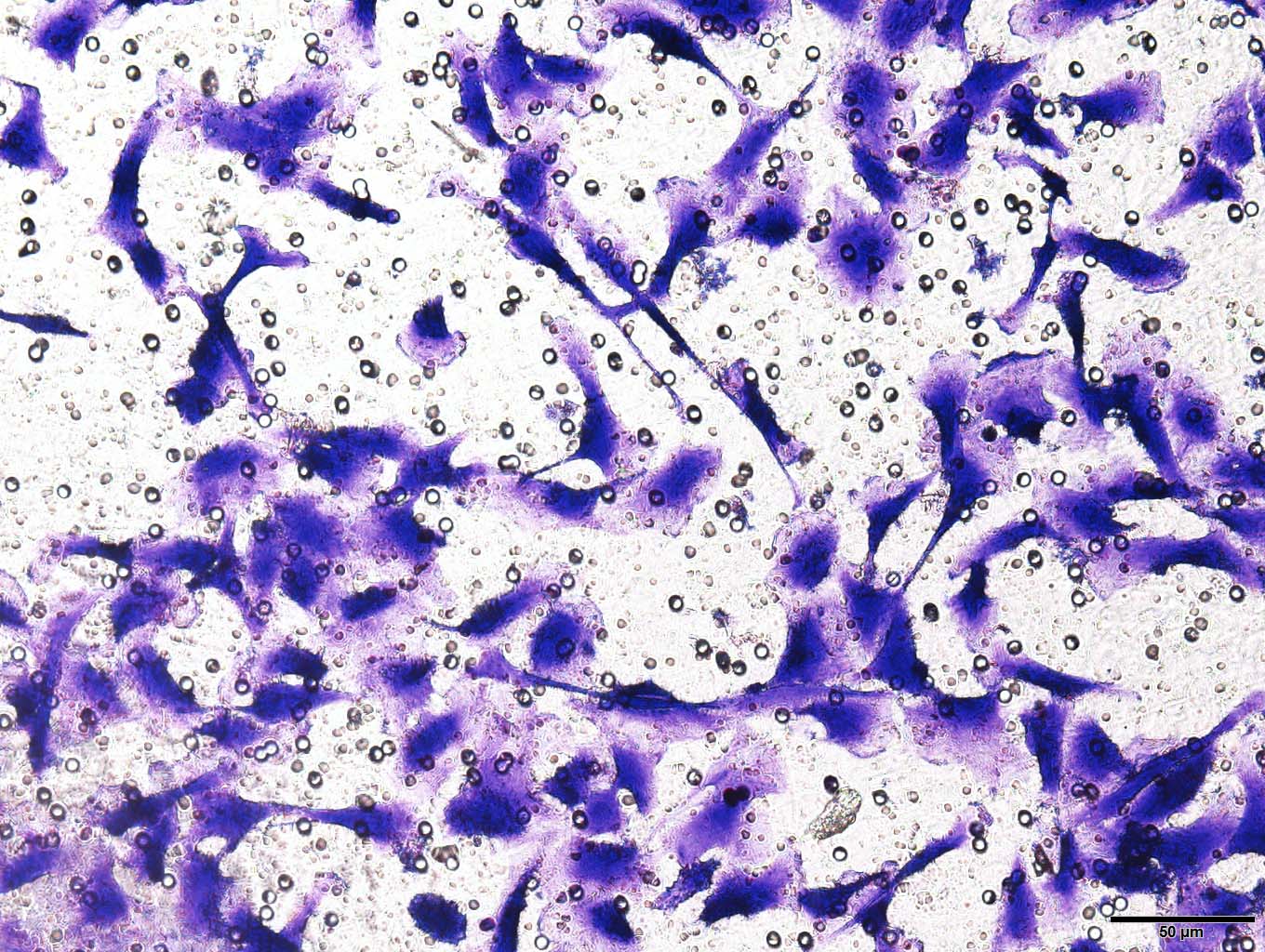

Supplement: S2 Fig — (ZIP) [file pone.0195844.s002.zip › S2 Appendix/S2_Fig2c-Inv-786O shCtrl í┴200 (2).jpg]

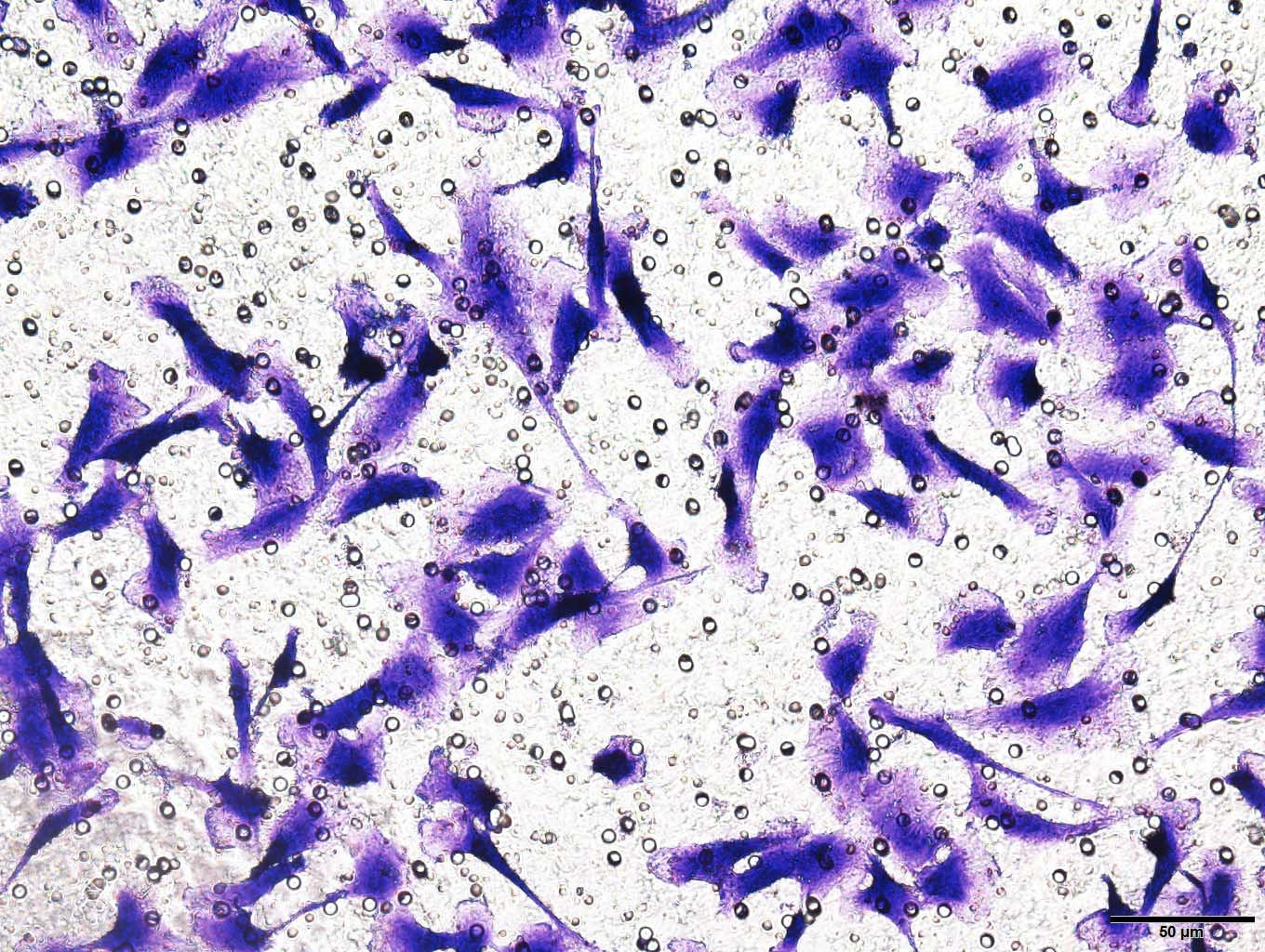

Supplement: S2 Fig — (ZIP) [file pone.0195844.s002.zip › S2 Appendix/S2_Fig2c-Inv-786O shCtrl í┴200 (3).jpg]

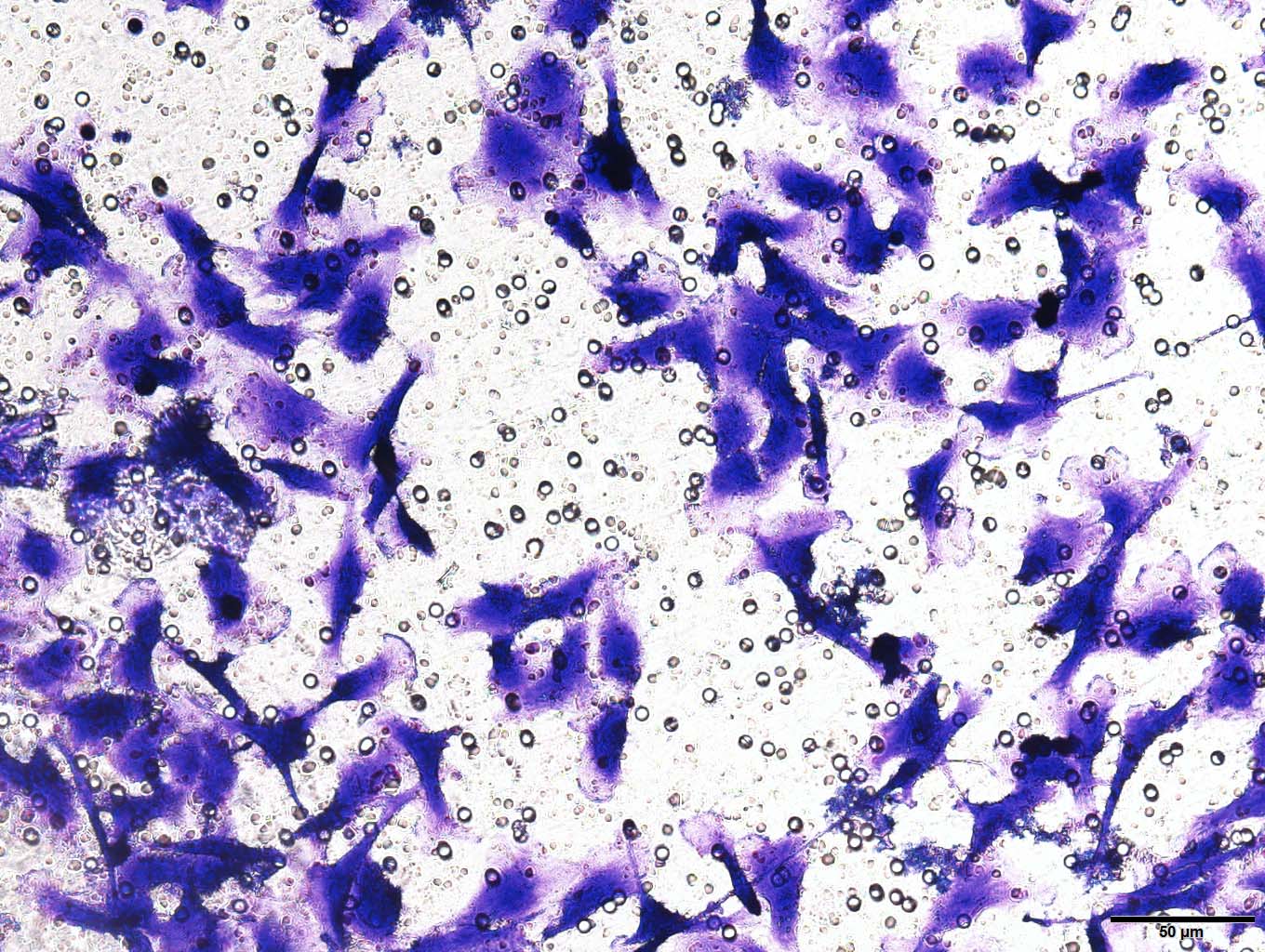

Supplement: S2 Fig — (ZIP) [file pone.0195844.s002.zip › S2 Appendix/S2_Fig2c-Inv-786O shCtrl í┴200 (4).jpg]

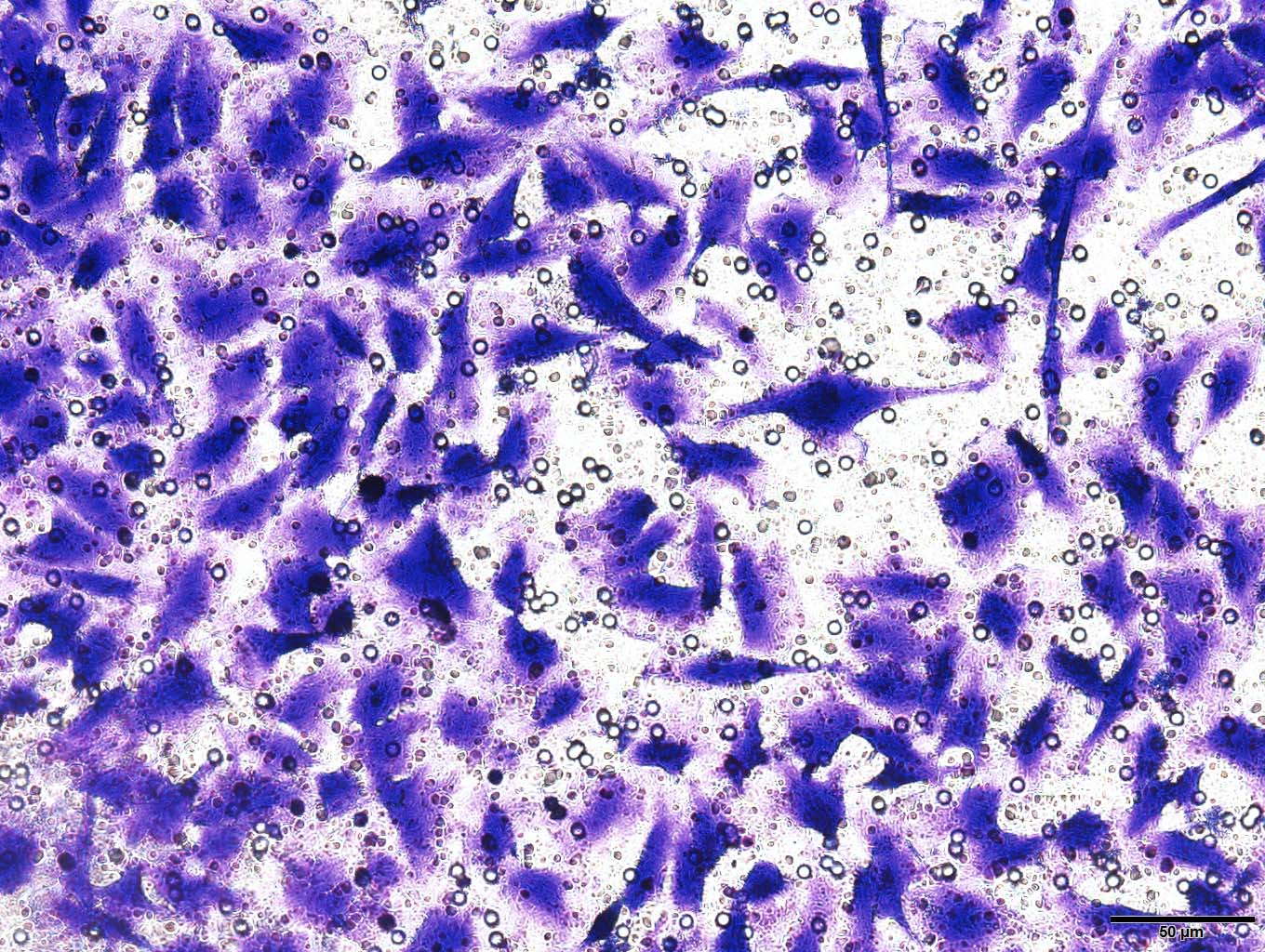

Supplement: S2 Fig — (ZIP) [file pone.0195844.s002.zip › S2 Appendix/S2_Fig2c-Inv-786O shCtrl í┴200 (5).jpg]

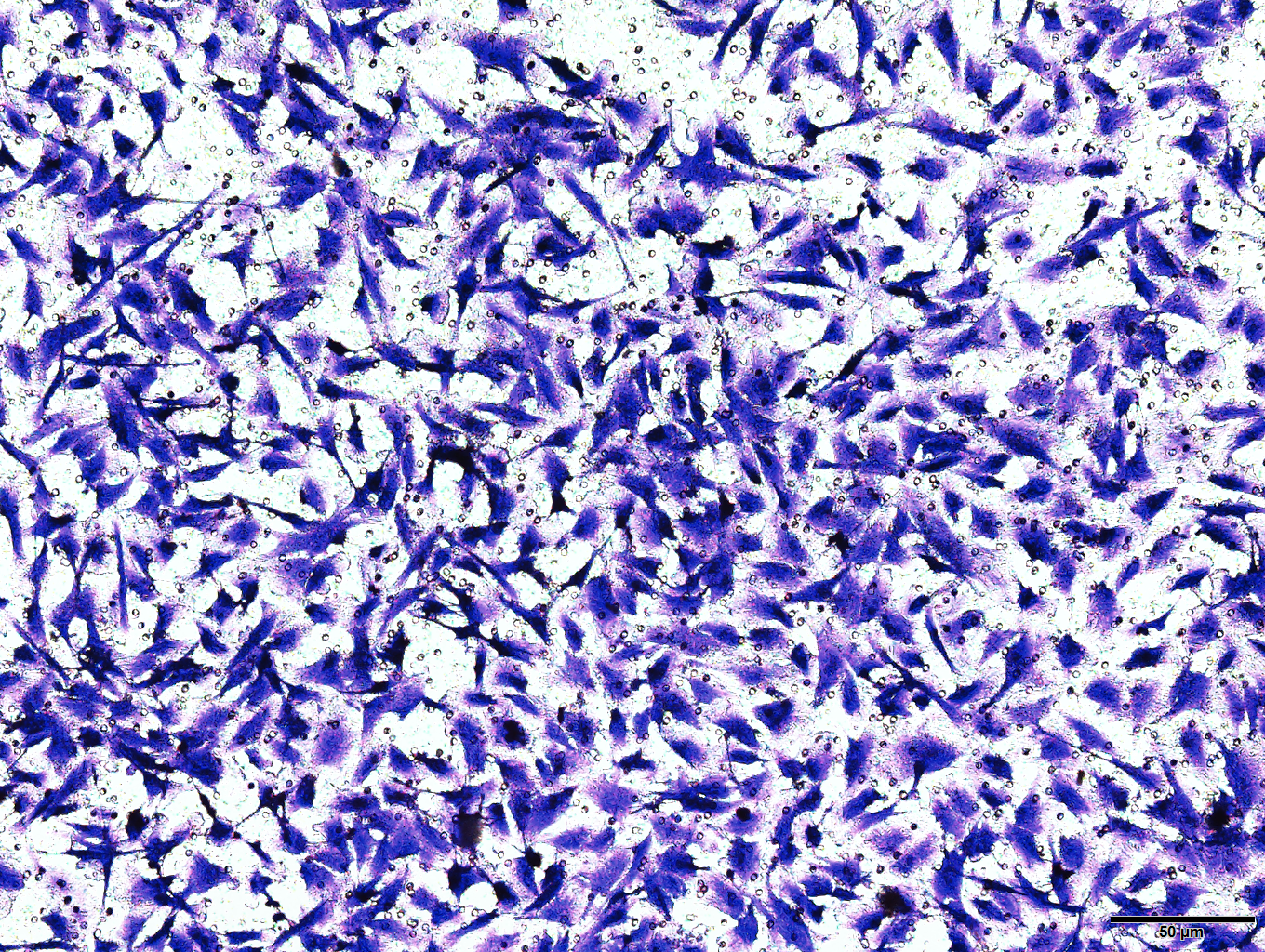

Supplement: S2 Fig — (ZIP) [file pone.0195844.s002.zip › S2 Appendix/S2_Fig2c-Inv-786O shVDR í┴100.tif]

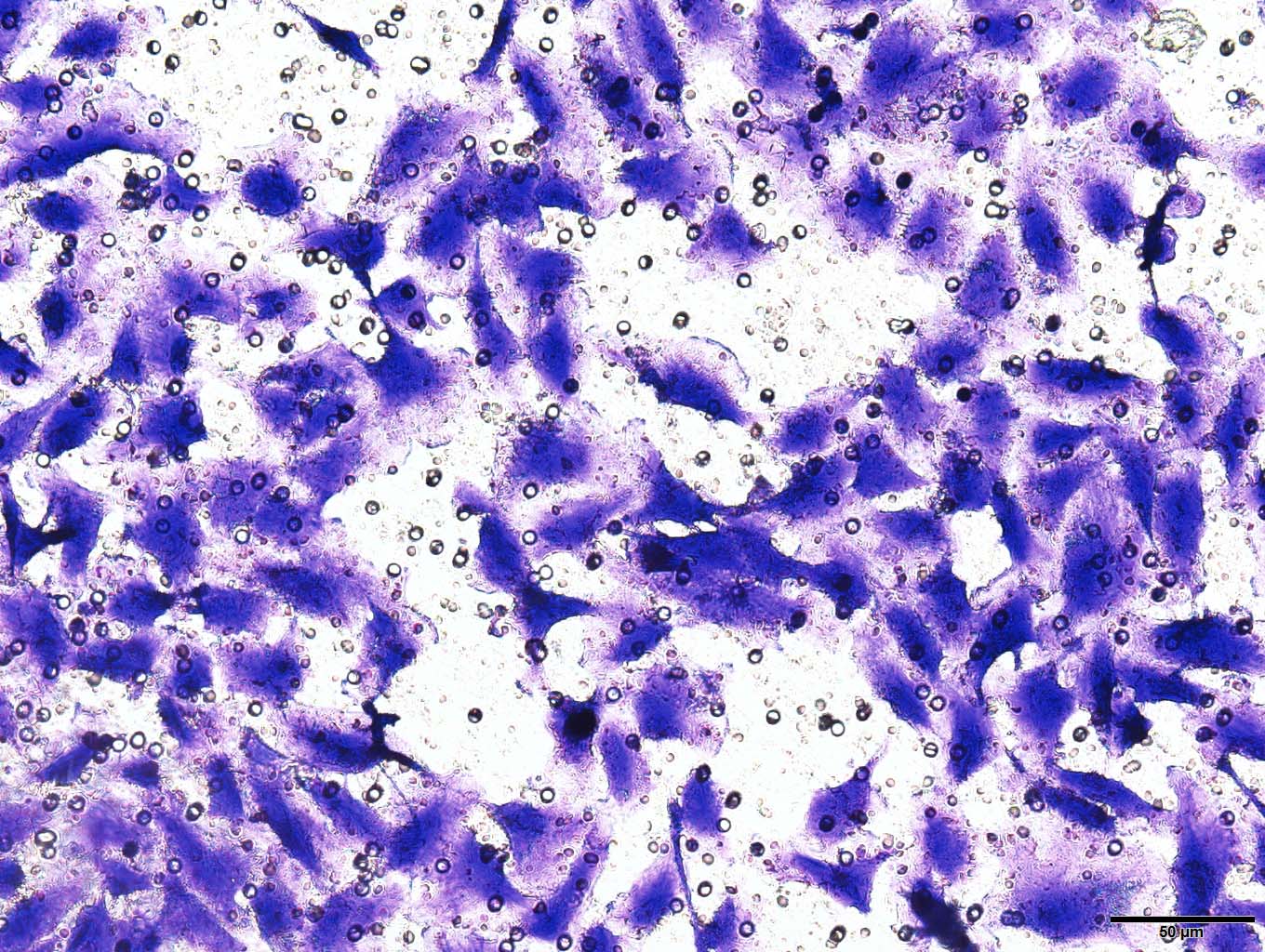

Supplement: S2 Fig — (ZIP) [file pone.0195844.s002.zip › S2 Appendix/S2_Fig2c-Inv-786O shVDR í┴200 (1).jpg]

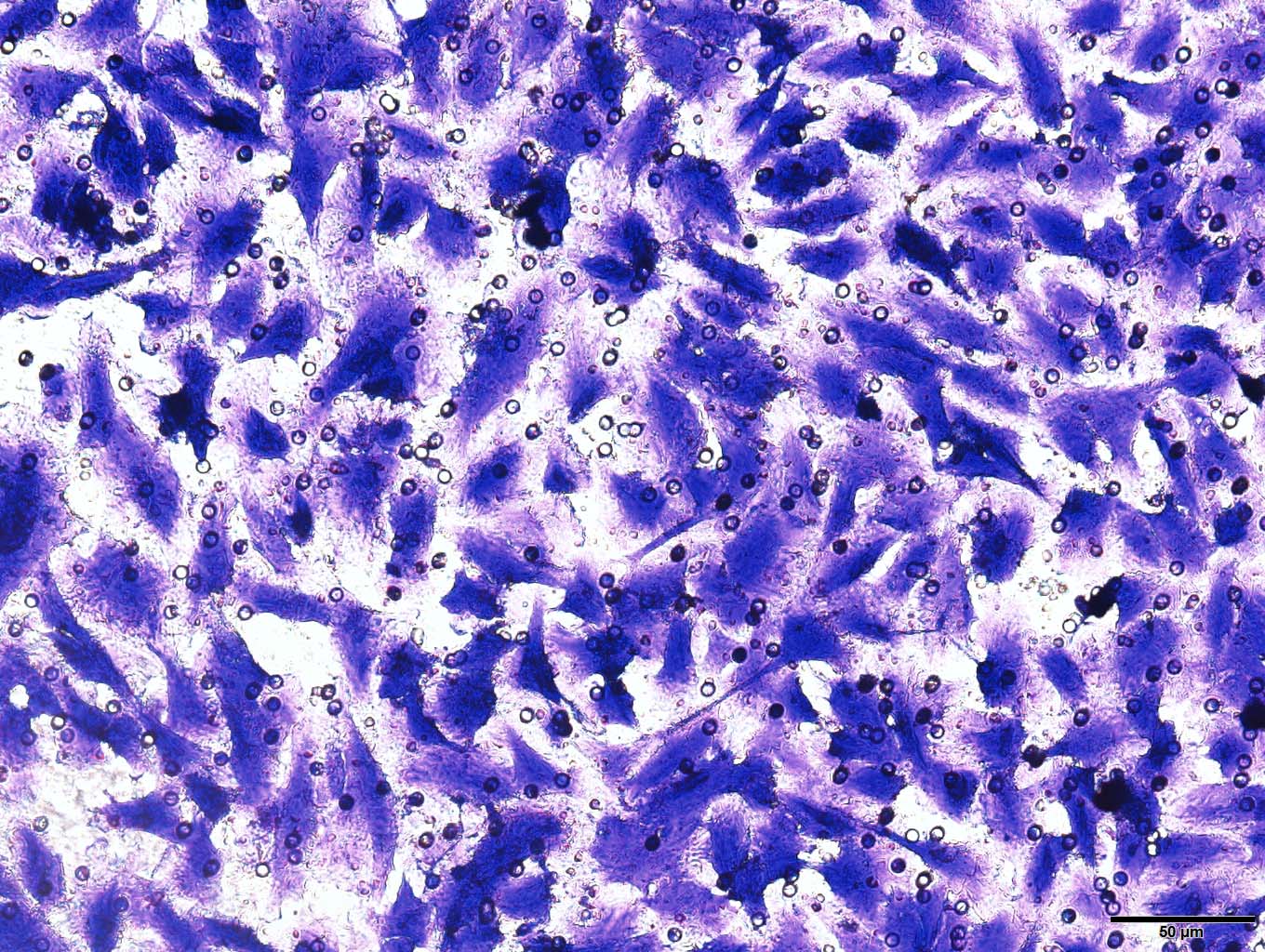

Supplement: S2 Fig — (ZIP) [file pone.0195844.s002.zip › S2 Appendix/S2_Fig2c-Inv-786O shVDR í┴200 (2).jpg]

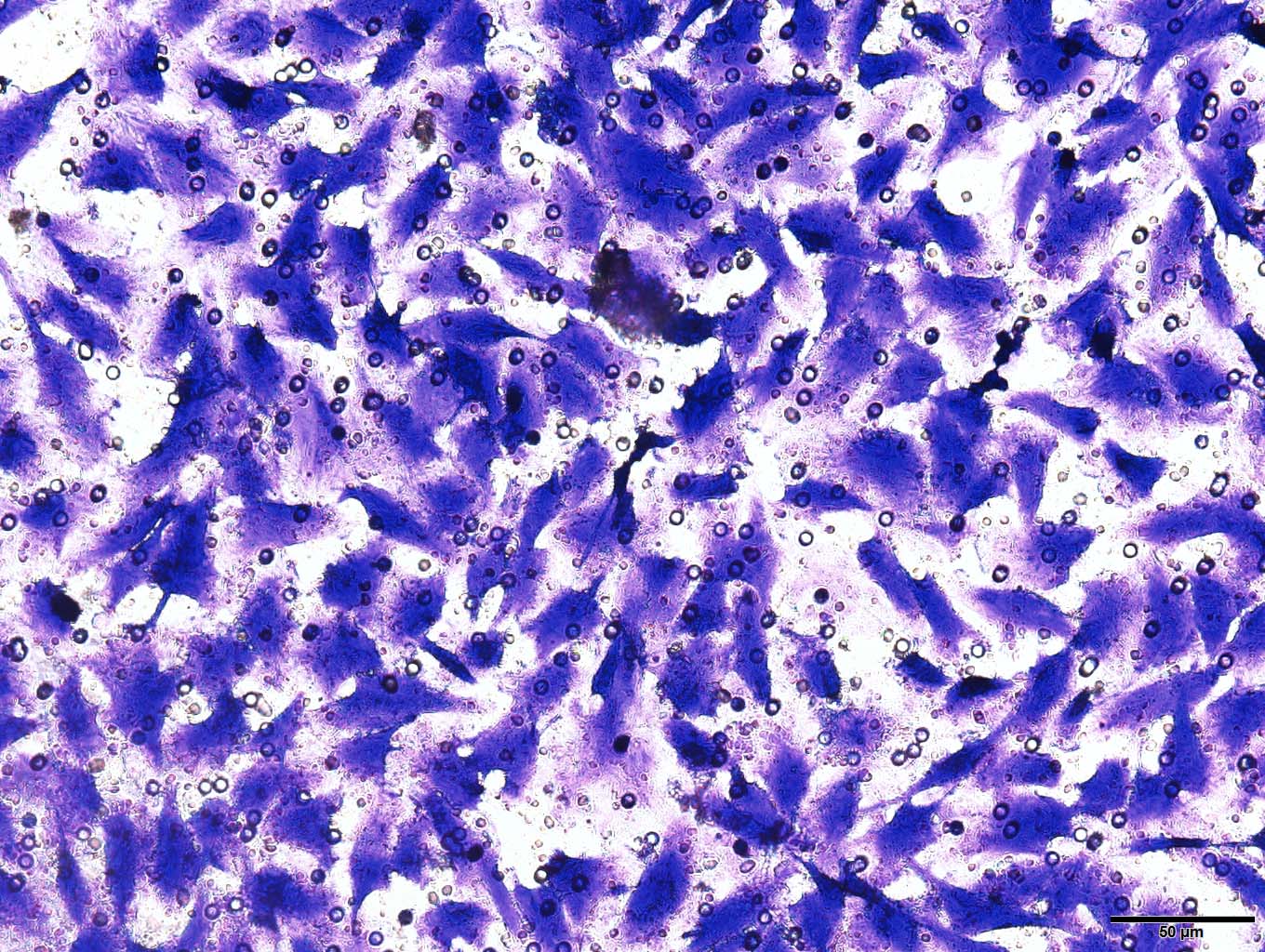

Supplement: S2 Fig — (ZIP) [file pone.0195844.s002.zip › S2 Appendix/S2_Fig2c-Inv-786O shVDR í┴200 (3).jpg]

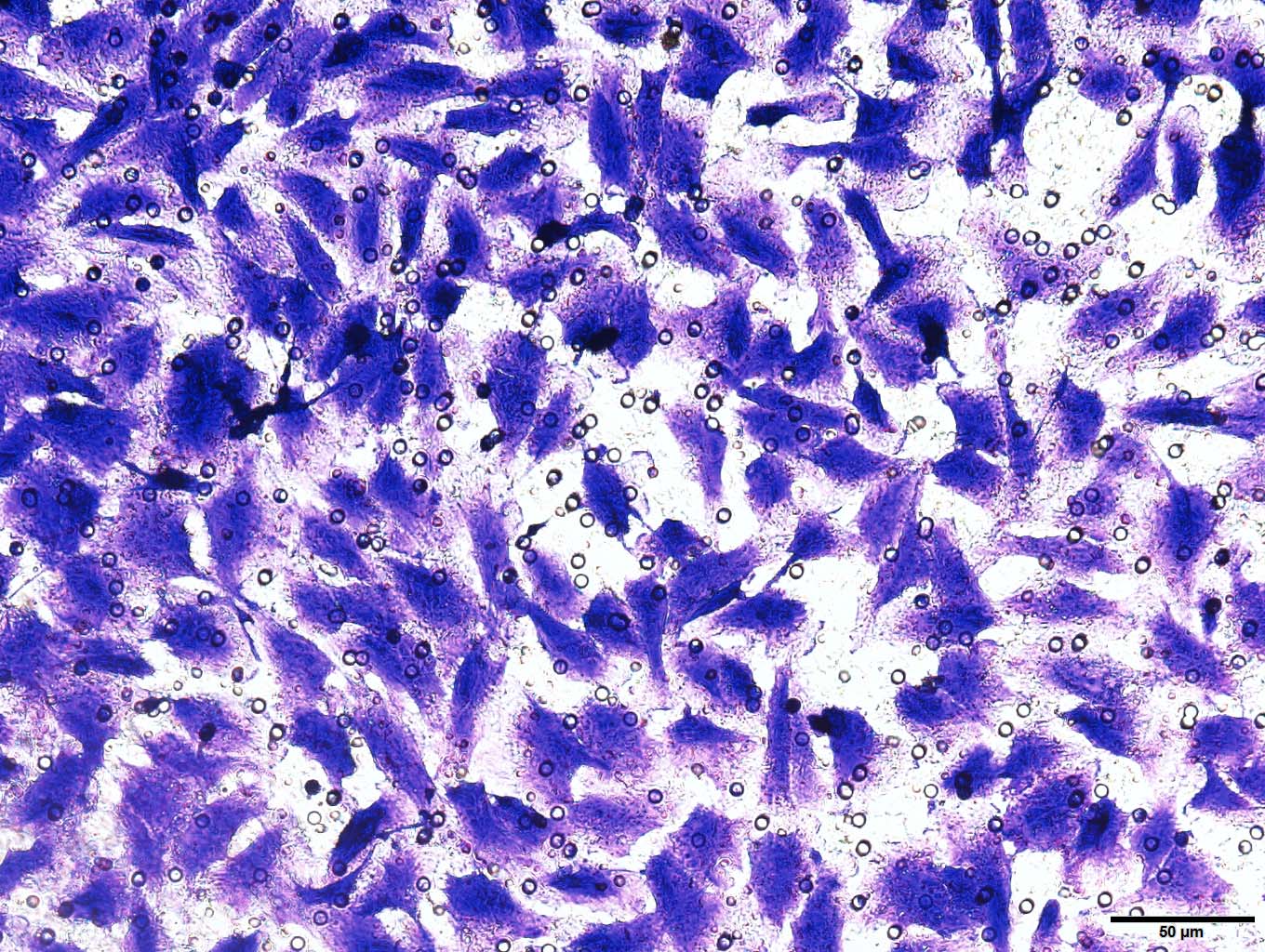

Supplement: S2 Fig — (ZIP) [file pone.0195844.s002.zip › S2 Appendix/S2_Fig2c-Inv-786O shVDR í┴200 (4).jpg]

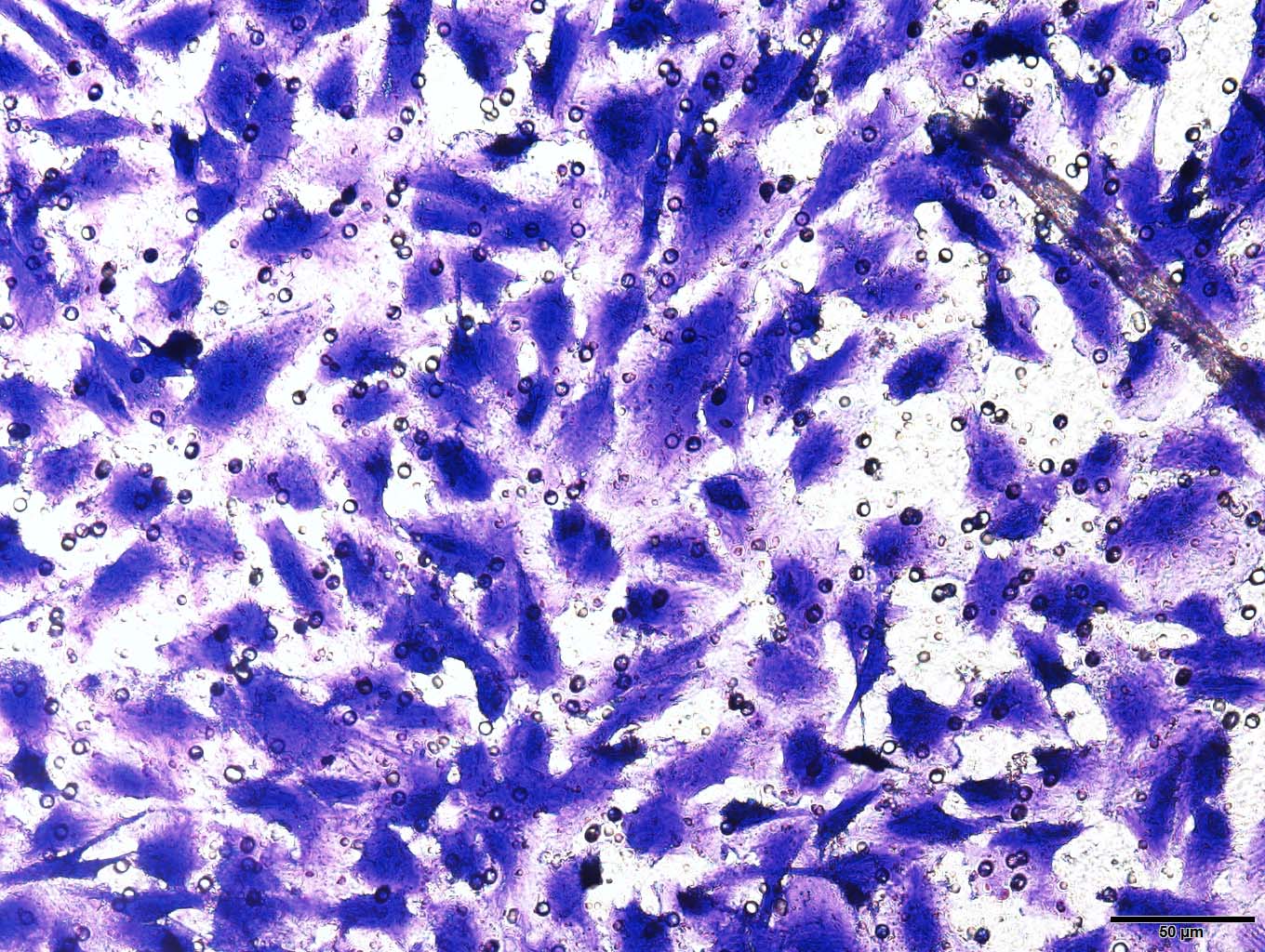

Supplement: S2 Fig — (ZIP) [file pone.0195844.s002.zip › S2 Appendix/S2_Fig2c-Inv-786O shVDR í┴200 (5).jpg]

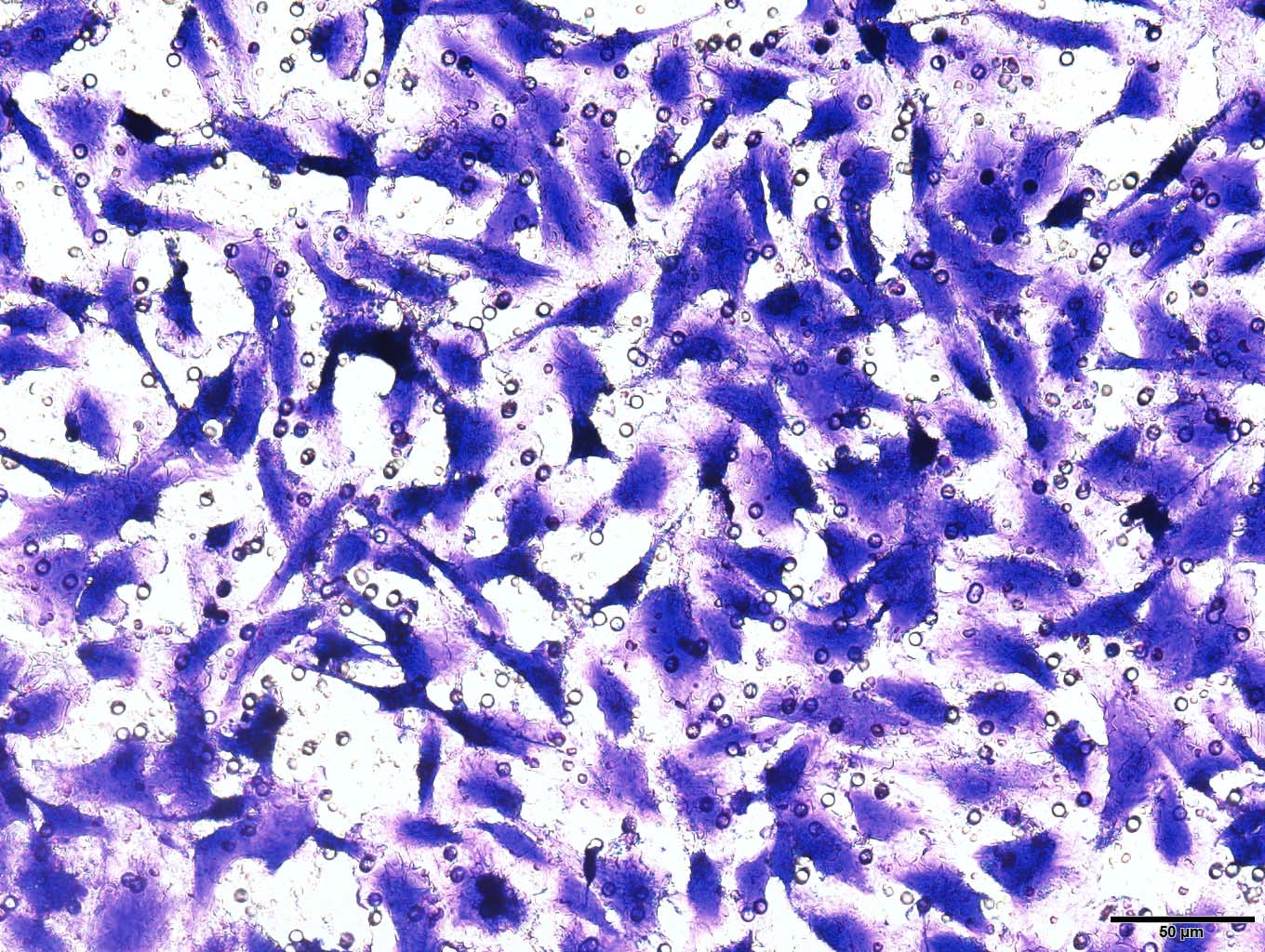

Supplement: S2 Fig — (ZIP) [file pone.0195844.s002.zip › S2 Appendix/S2_Fig2c-Inv-786O shVDR í┴200 (6).jpg]

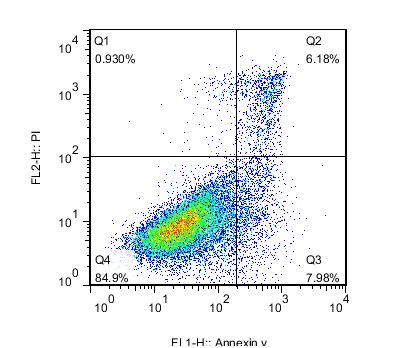

Supplement: S3 Fig — (ZIP) [file pone.0195844.s003.zip › S3 Appendix/FSC-H,_SSC-H_subset leCtrl-1.PNG]

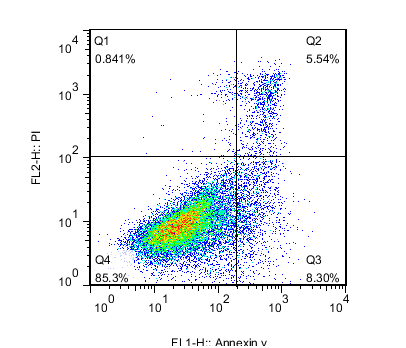

Supplement: S3 Fig — (ZIP) [file pone.0195844.s003.zip › S3 Appendix/FSC-H,_SSC-H_subset leCtrl-2.PNG]

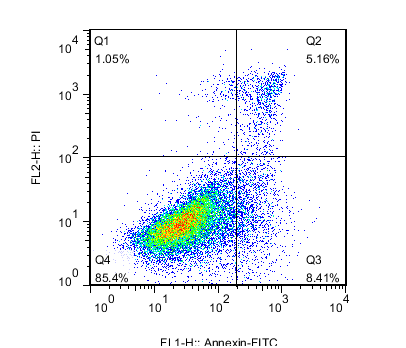

Supplement: S3 Fig — (ZIP) [file pone.0195844.s003.zip › S3 Appendix/FSC-H,_SSC-H_subset leCtrl-3.PNG]

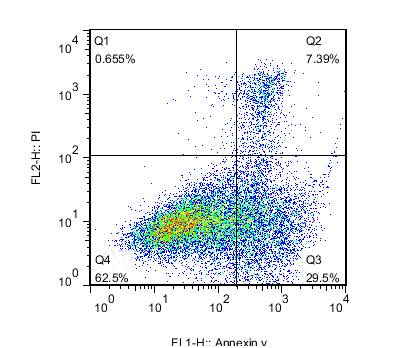

Supplement: S3 Fig — (ZIP) [file pone.0195844.s003.zip › S3 Appendix/FSC-H,_SSC-H_subset leVDR -1.PNG]

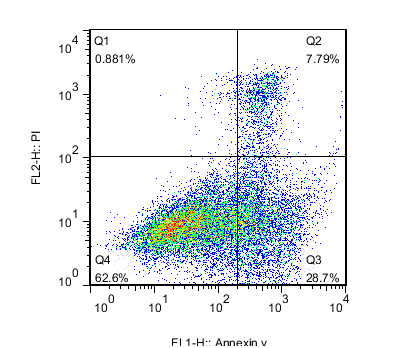

Supplement: S3 Fig — (ZIP) [file pone.0195844.s003.zip › S3 Appendix/FSC-H,_SSC-H_subset leVDR-2.PNG]

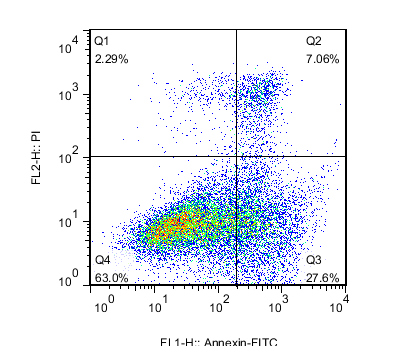

Supplement: S3 Fig — (ZIP) [file pone.0195844.s003.zip › S3 Appendix/FSC-H,_SSC-H_subset leVDR-3.PNG]

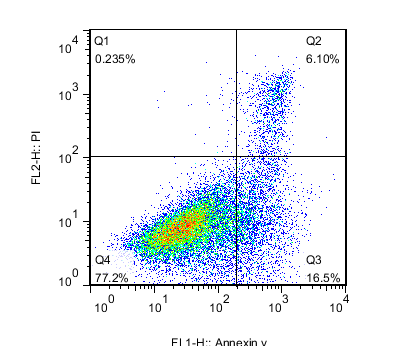

Supplement: S3 Fig — (ZIP) [file pone.0195844.s003.zip › S3 Appendix/FSC-H,_SSC-H_subset shCtrl-1.PNG]

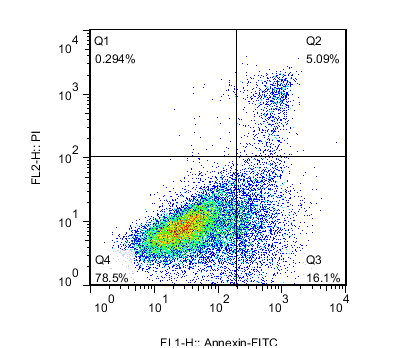

Supplement: S3 Fig — (ZIP) [file pone.0195844.s003.zip › S3 Appendix/FSC-H,_SSC-H_subset shCtrl-3.PNG]

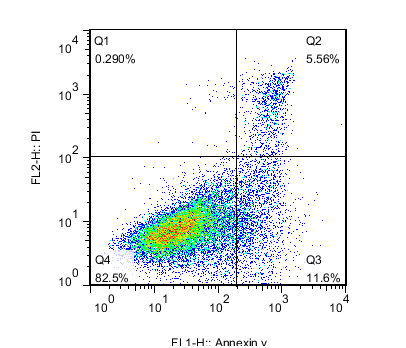

Supplement: S3 Fig — (ZIP) [file pone.0195844.s003.zip › S3 Appendix/FSC-H,_SSC-H_subset shVDR-1.PNG]

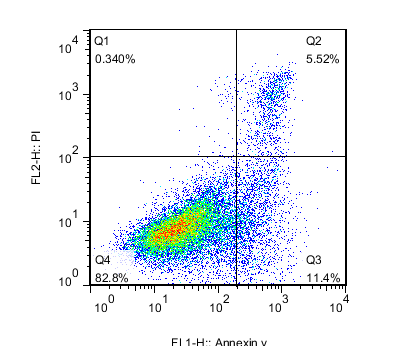

Supplement: S3 Fig — (ZIP) [file pone.0195844.s003.zip › S3 Appendix/FSC-H,_SSC-H_subset shVDR-2.PNG]

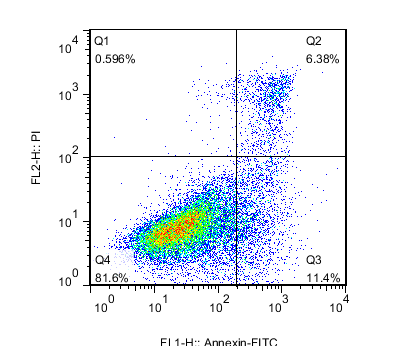

Supplement: S3 Fig — (ZIP) [file pone.0195844.s003.zip › S3 Appendix/FSC-H,_SSC-H_subset shVDR-3.PNG]

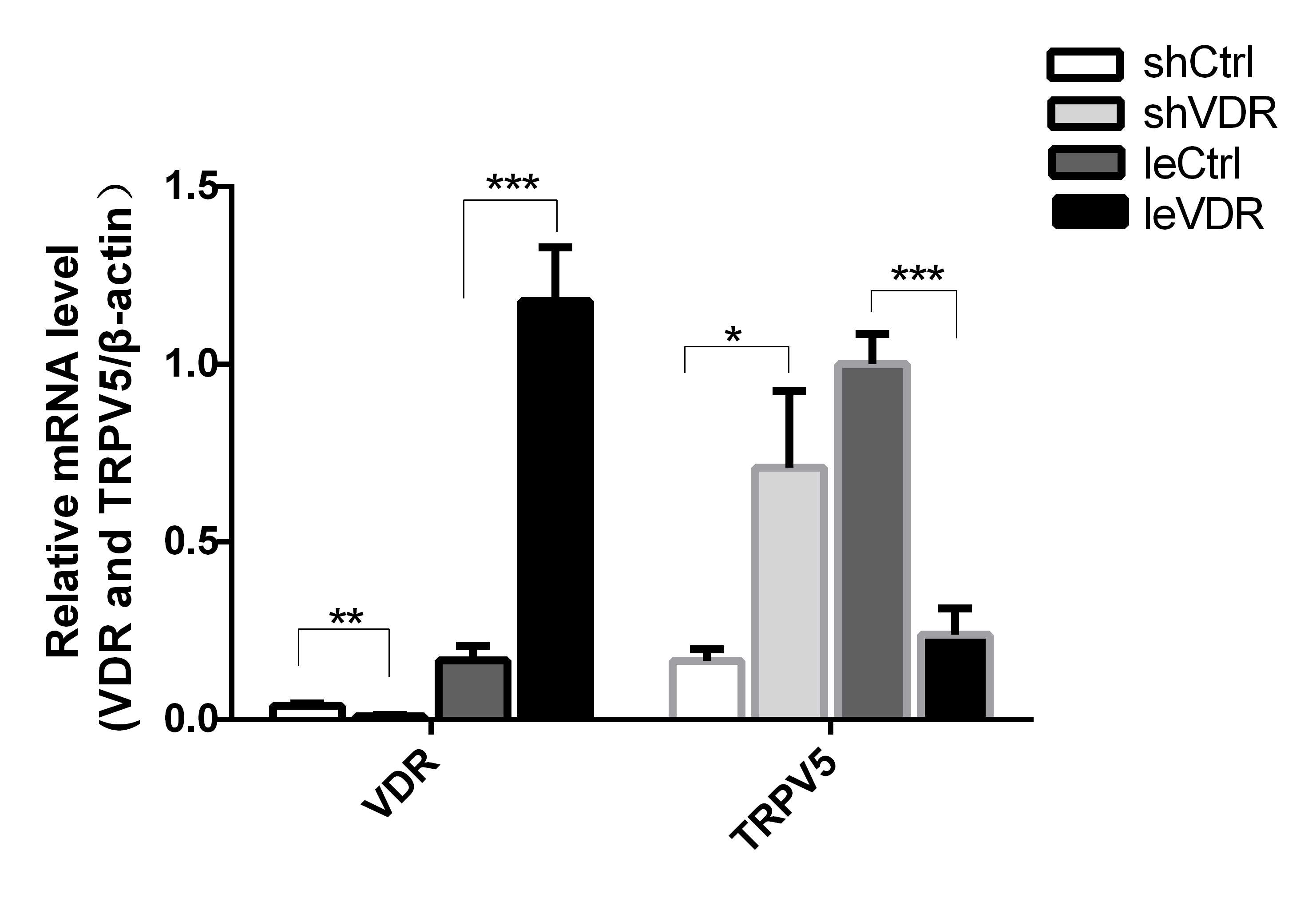

Supplement: S4 Fig — (ZIP) [file pone.0195844.s004.zip › S4_Fig4_File/S4_Fig4A-RTPCR-Caki-1.jpg]

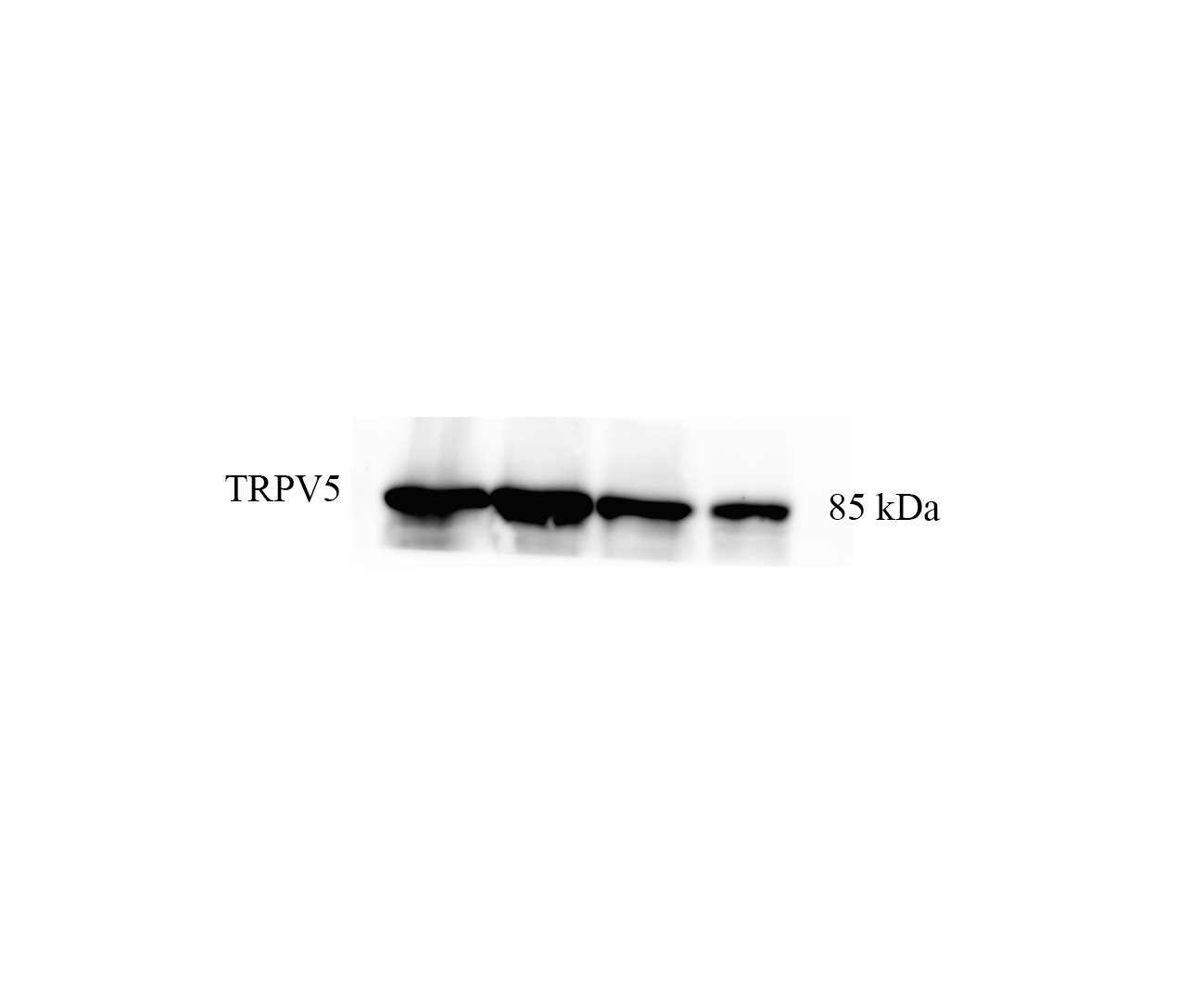

Supplement: S4 Fig — (ZIP) [file pone.0195844.s004.zip › S4_Fig4_File/S4_Fig4B-WB-TRPV5 (2).tif]

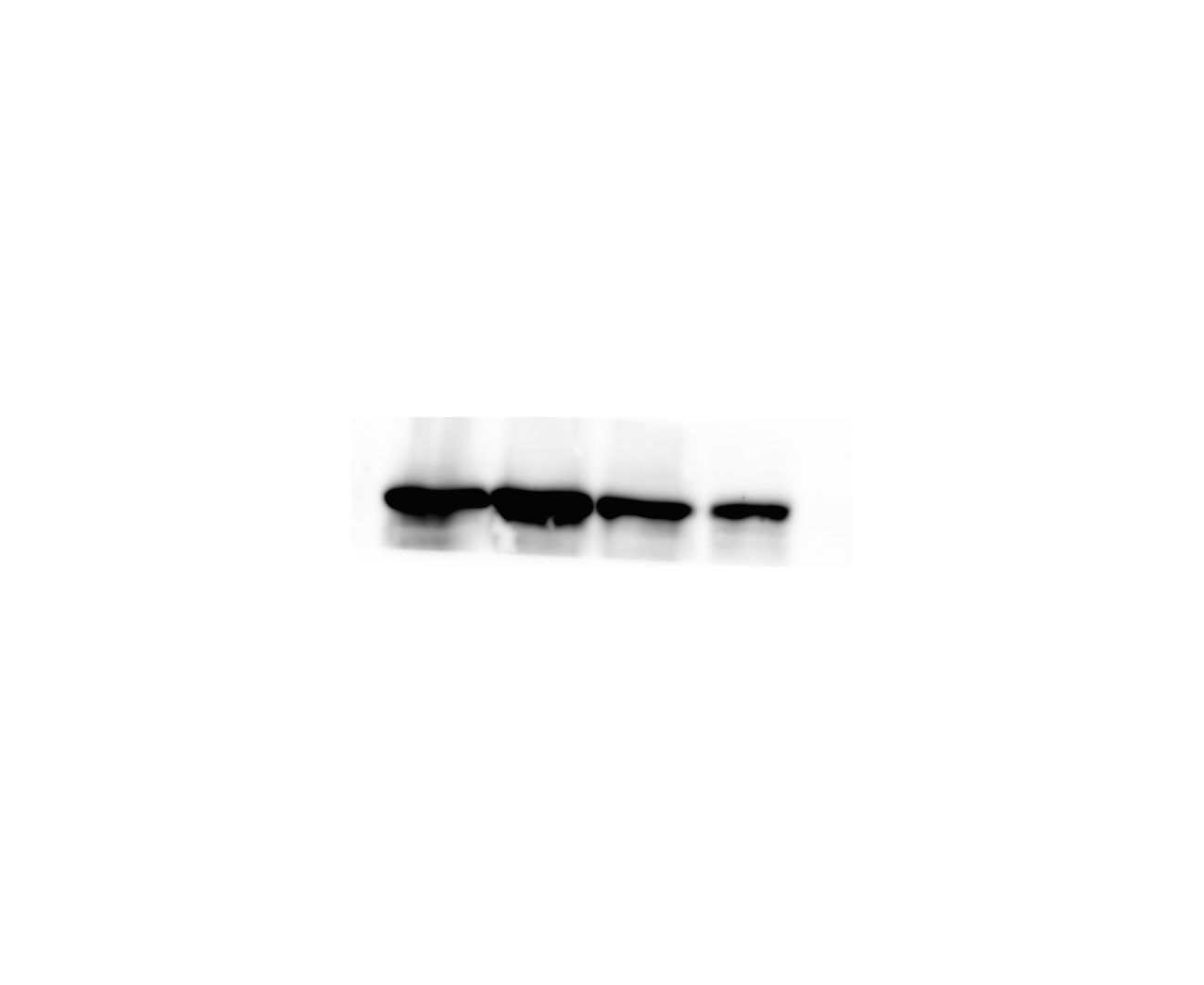

Supplement: S4 Fig — (ZIP) [file pone.0195844.s004.zip › S4_Fig4_File/S4_Fig4B-WB-TRPV5.tif]

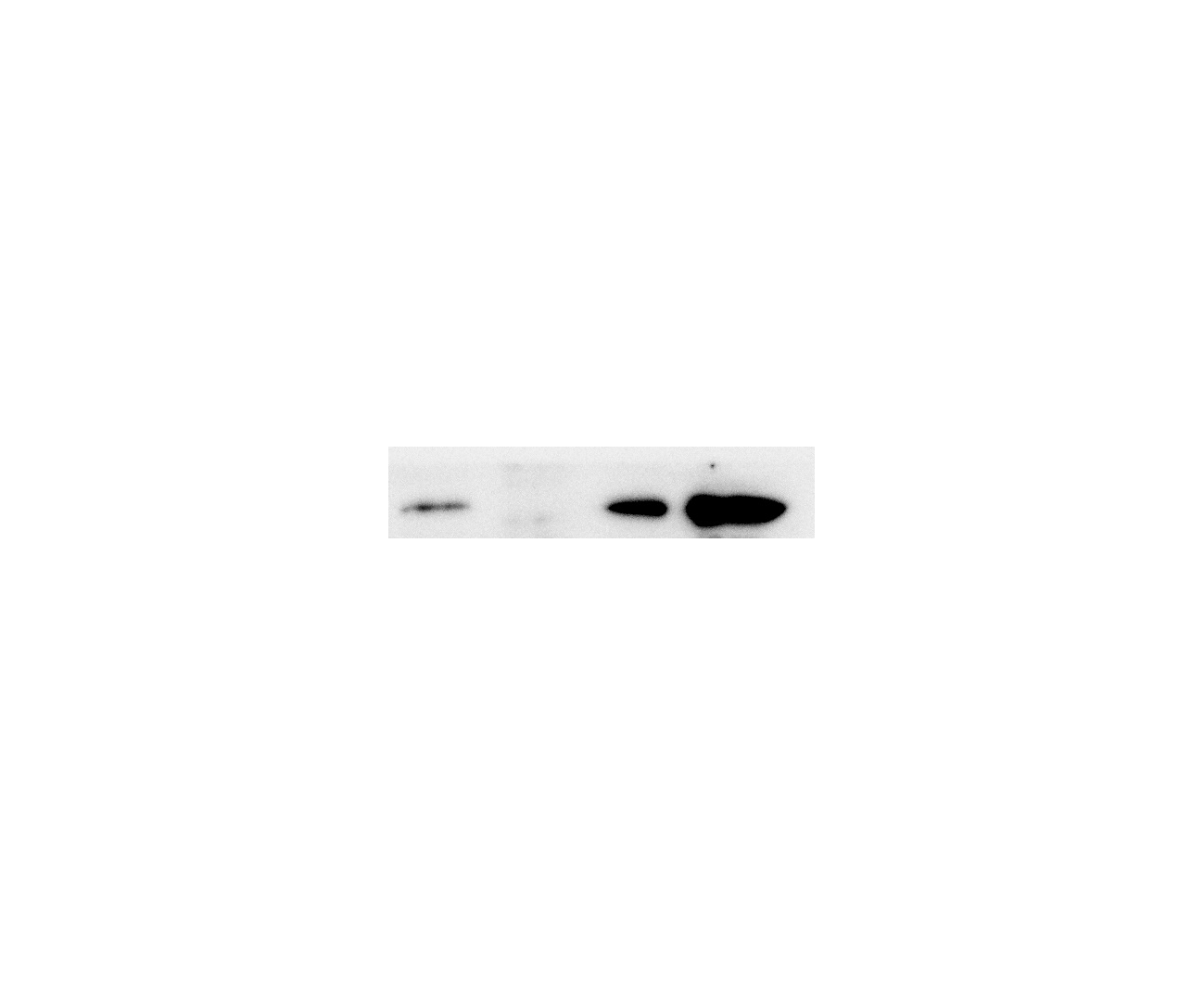

Supplement: S4 Fig — (ZIP) [file pone.0195844.s004.zip › S4_Fig4_File/S4_Fig4B-WB-VDR (2).tif]

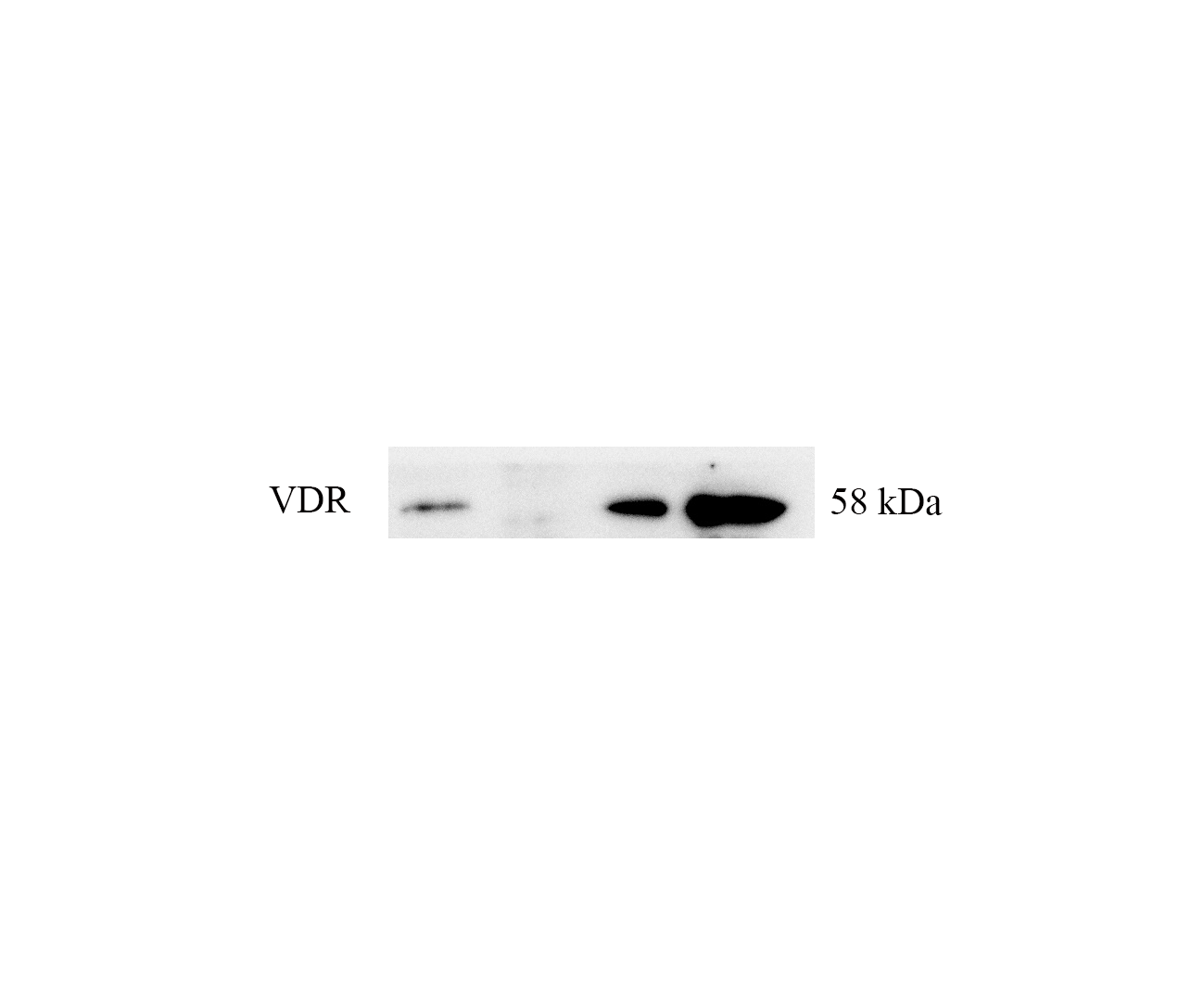

Supplement: S4 Fig — (ZIP) [file pone.0195844.s004.zip › S4_Fig4_File/S4_Fig4B-WB-VDR (3).tif]

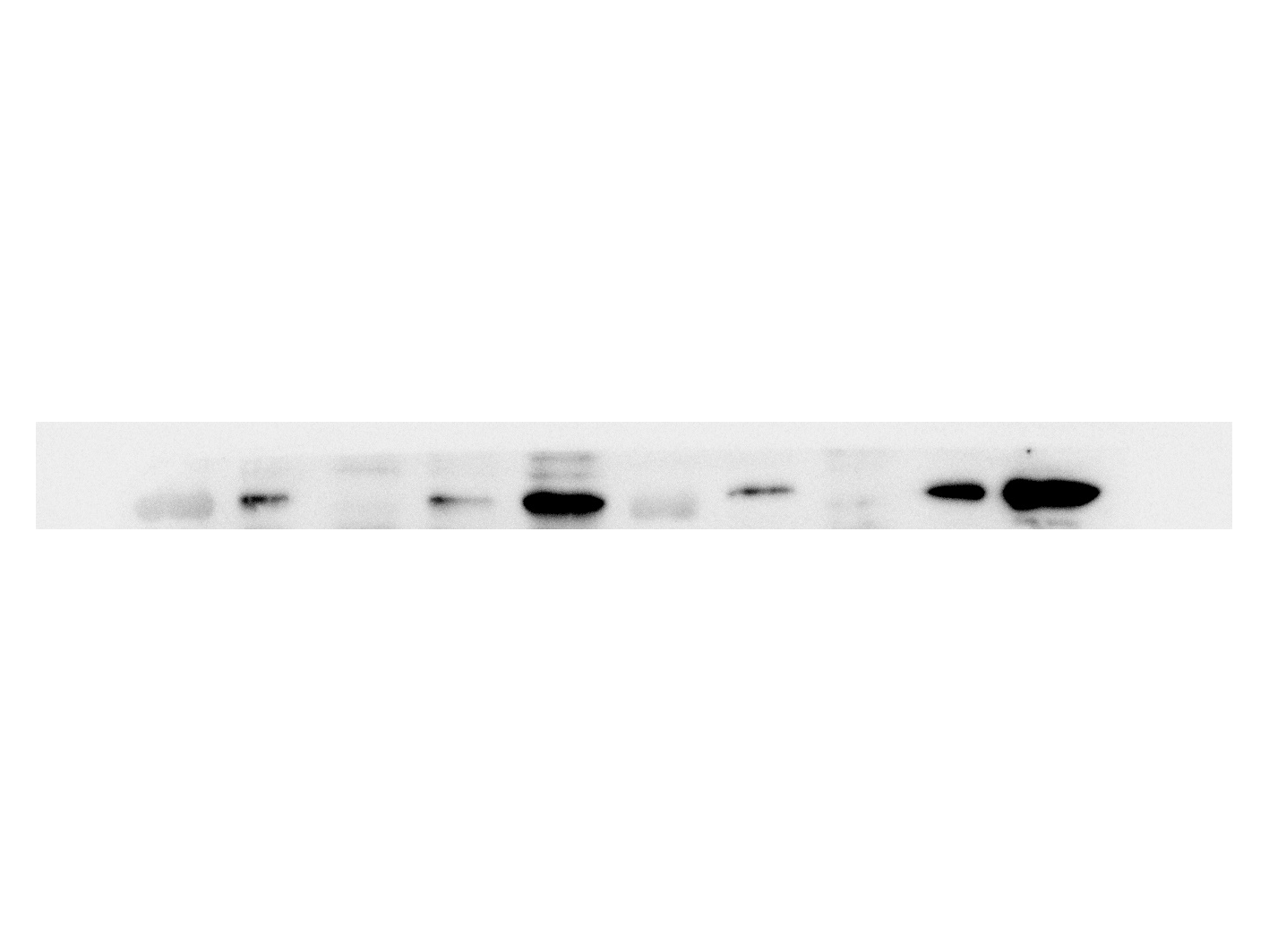

Supplement: S4 Fig — (ZIP) [file pone.0195844.s004.zip › S4_Fig4_File/S4_Fig4B-WB-VDR.tif]

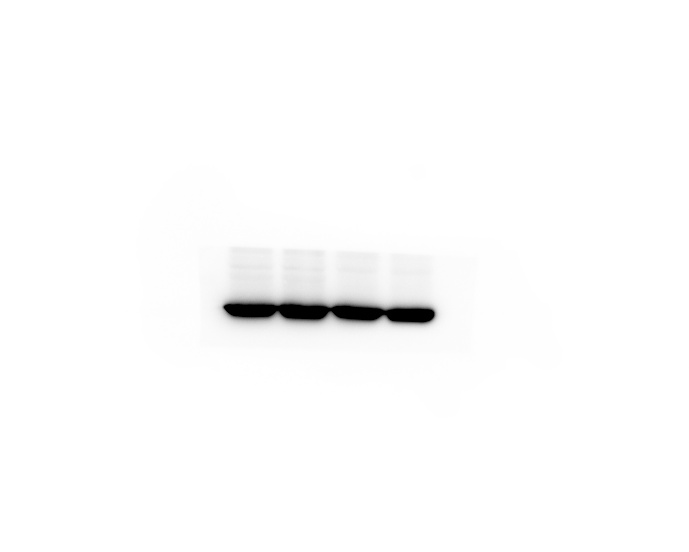

Supplement: S4 Fig — (ZIP) [file pone.0195844.s004.zip › S4_Fig4_File/S4_Fig4B-WB-a┬-actin.jpg]

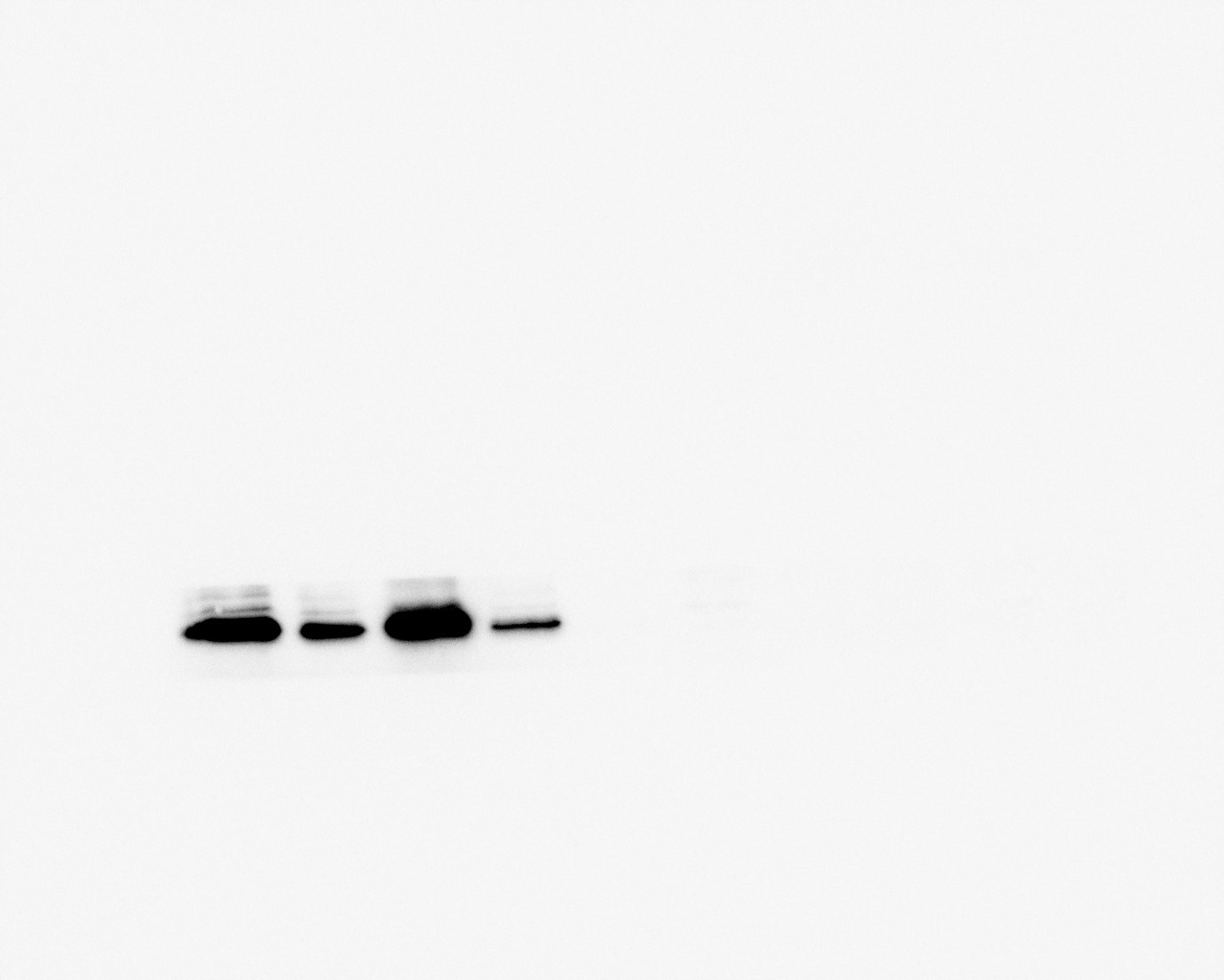

Supplement: S5 Fig — (ZIP) [file pone.0195844.s005.zip › S5_Fig5_File/S5_Fig5A-WB-TRPV5.jpg]

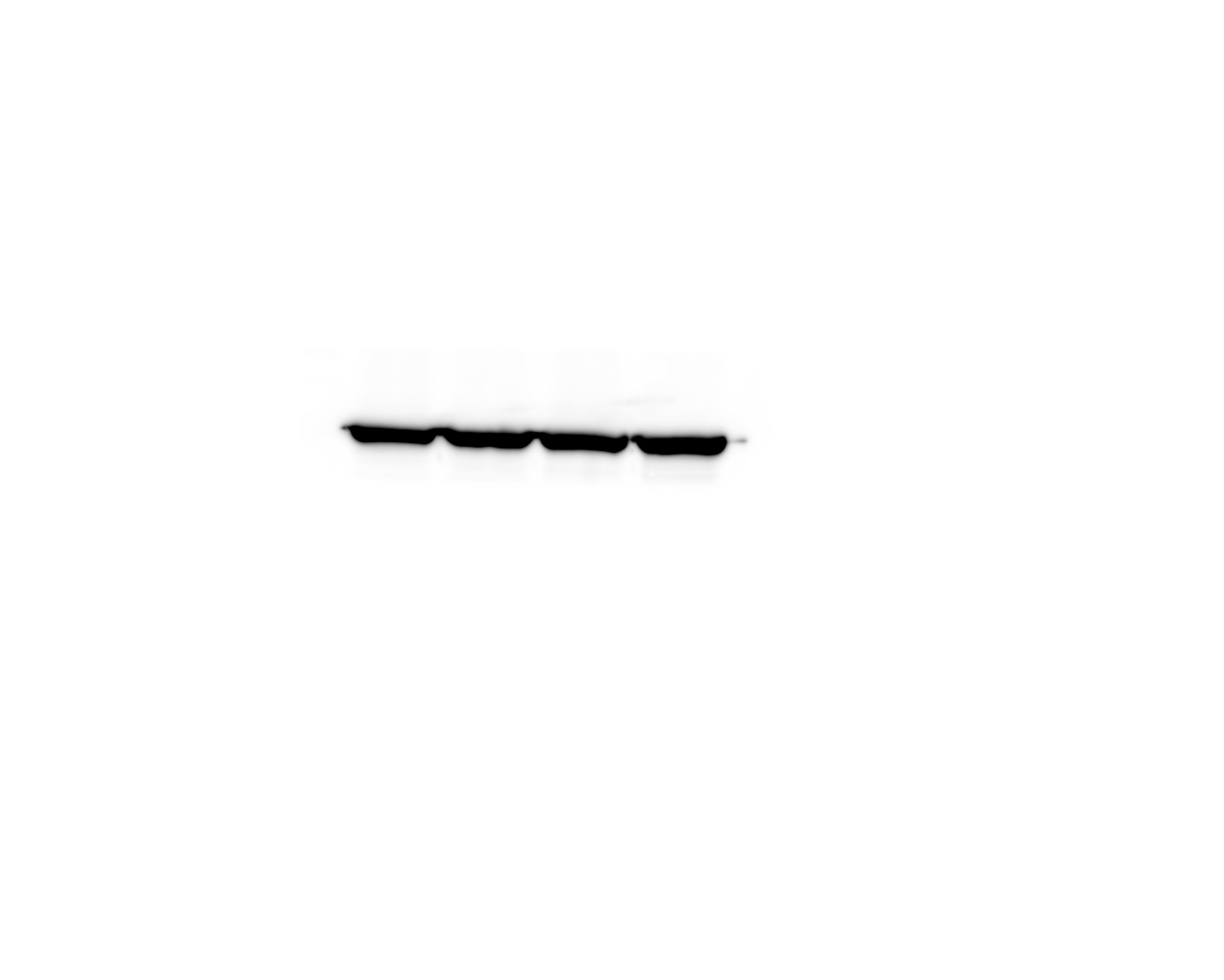

Supplement: S5 Fig — (ZIP) [file pone.0195844.s005.zip › S5_Fig5_File/S5_Fig5A-WB-a┬-actin (2).jpg]

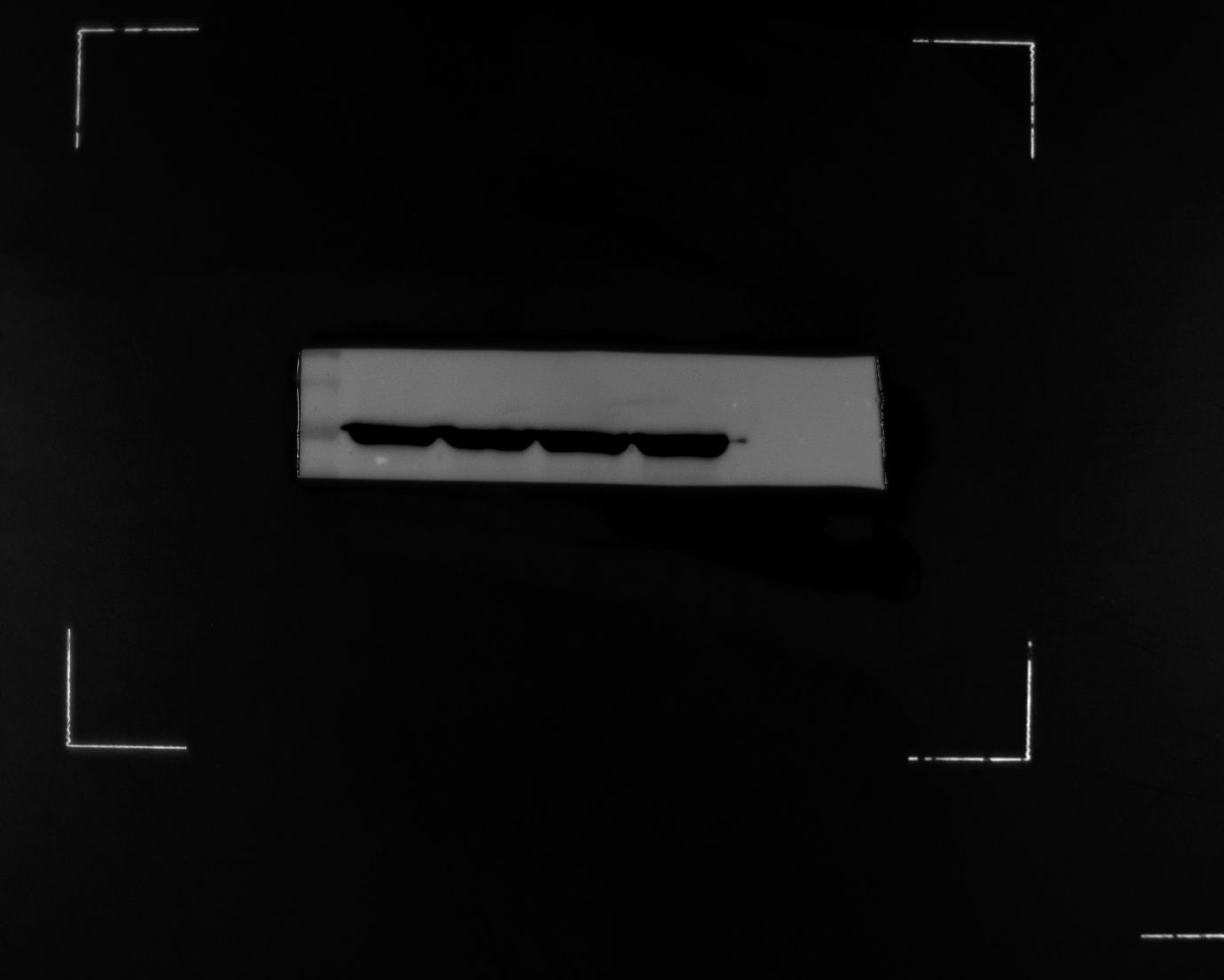

Supplement: S5 Fig — (ZIP) [file pone.0195844.s005.zip › S5_Fig5_File/S5_Fig5A-WB-a┬-actin.jpg]

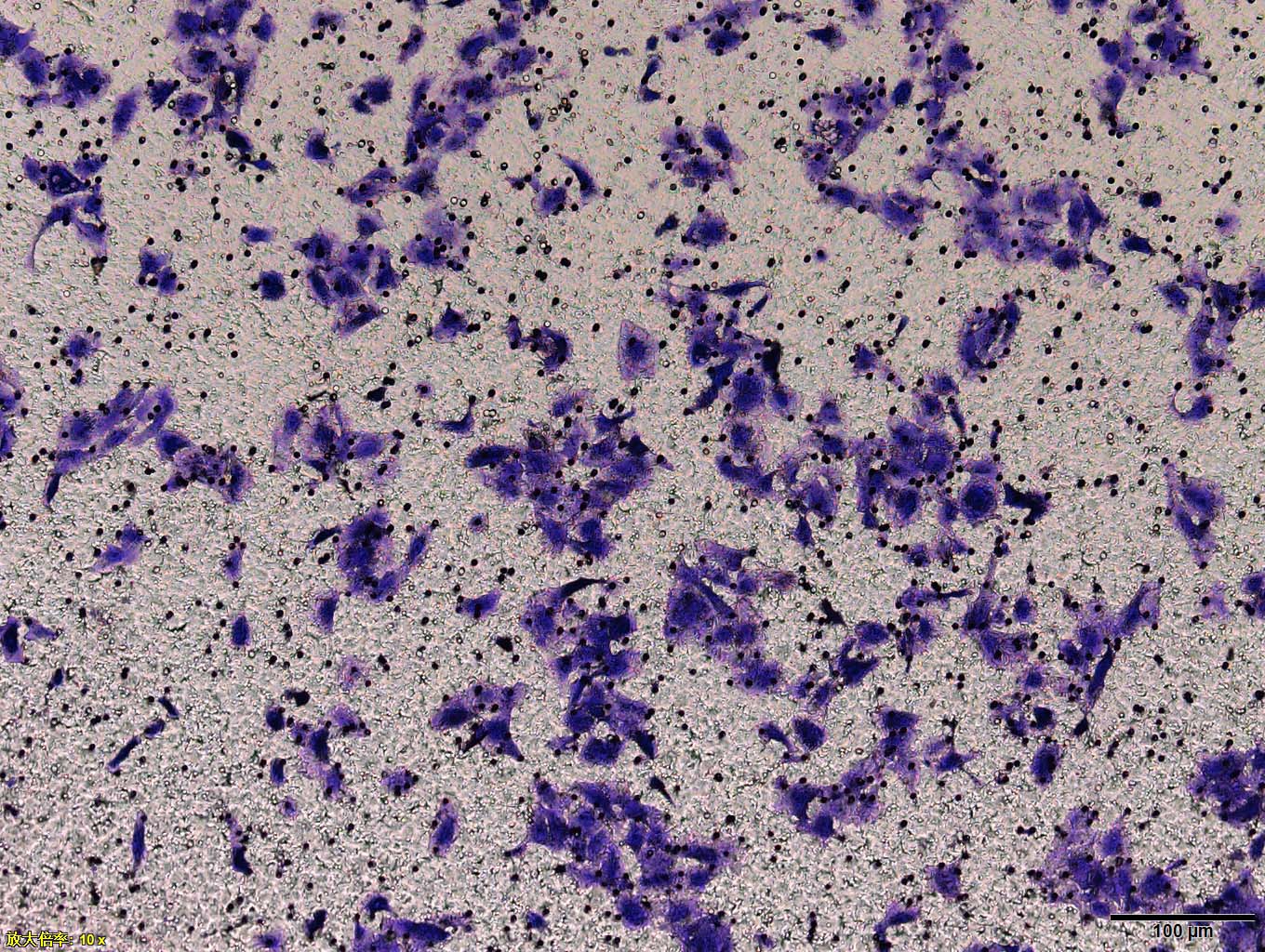

Supplement: S5 Fig — (ZIP) [file pone.0195844.s005.zip › S5_Fig5_File/S5_Fig5C_shCtrl í┴100.jpg]

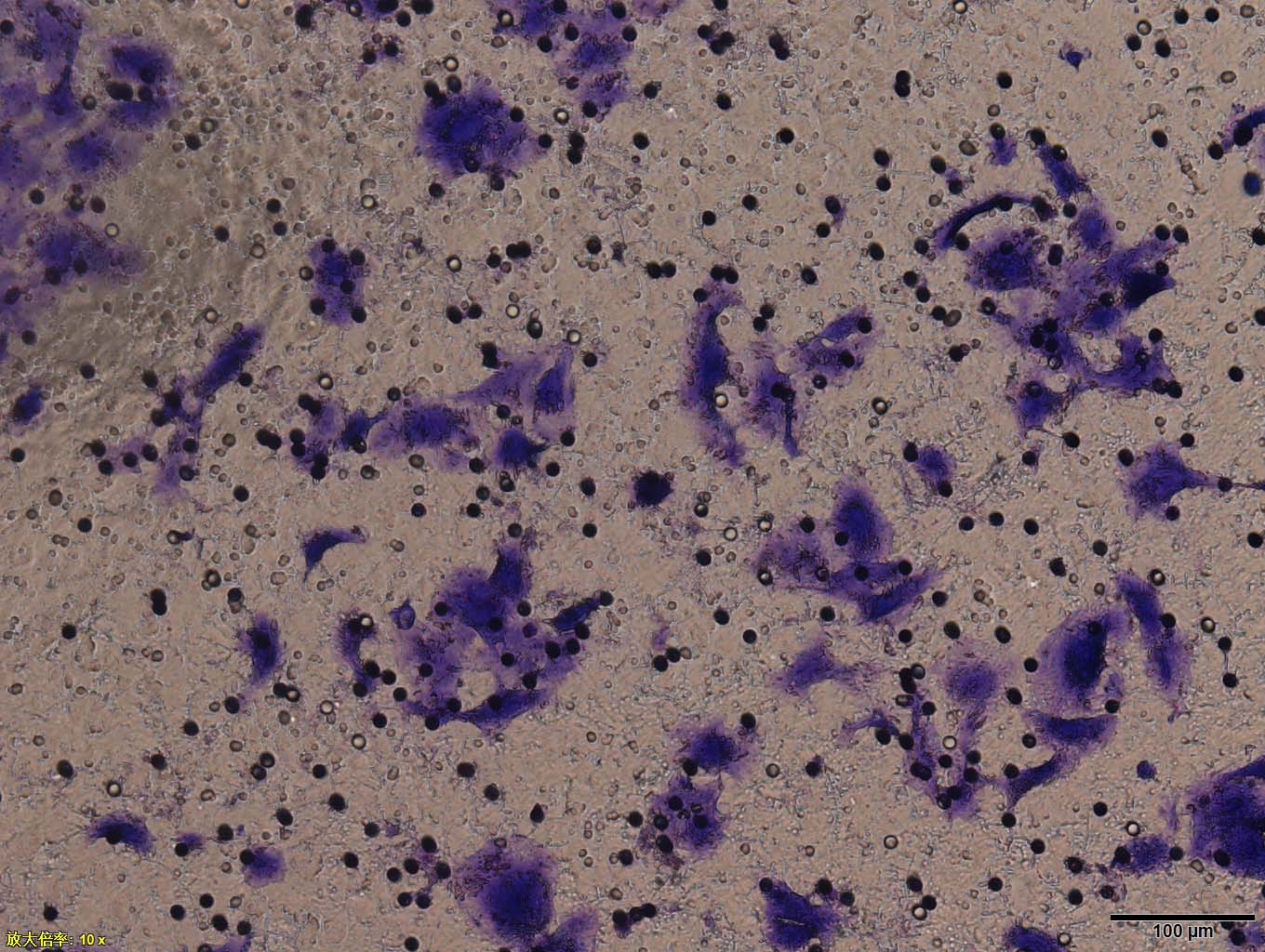

Supplement: S5 Fig — (ZIP) [file pone.0195844.s005.zip › S5_Fig5_File/S5_Fig5C_shCtrl í┴200 (1).jpg]

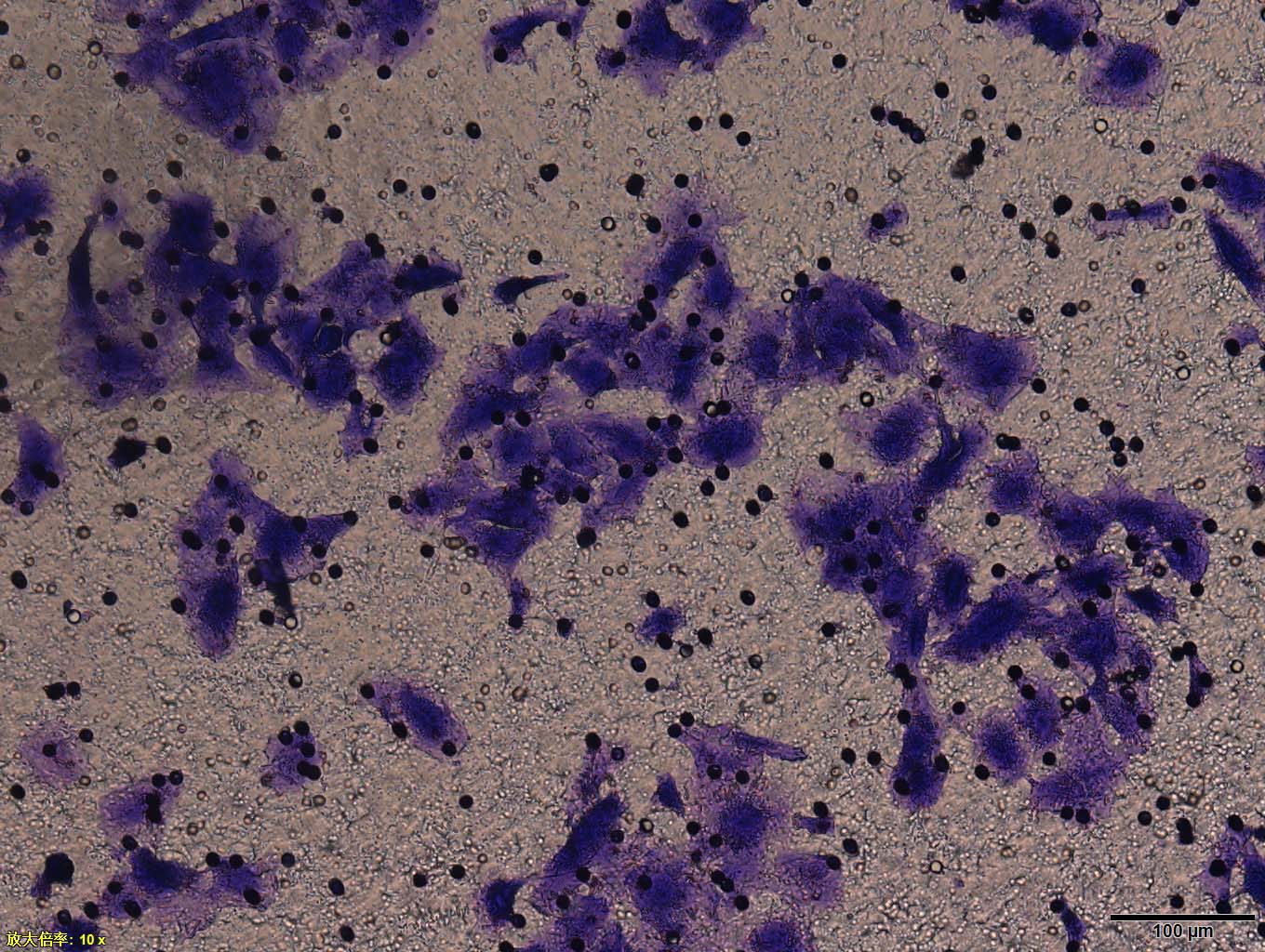

Supplement: S5 Fig — (ZIP) [file pone.0195844.s005.zip › S5_Fig5_File/S5_Fig5C_shCtrl í┴200 (2).jpg]

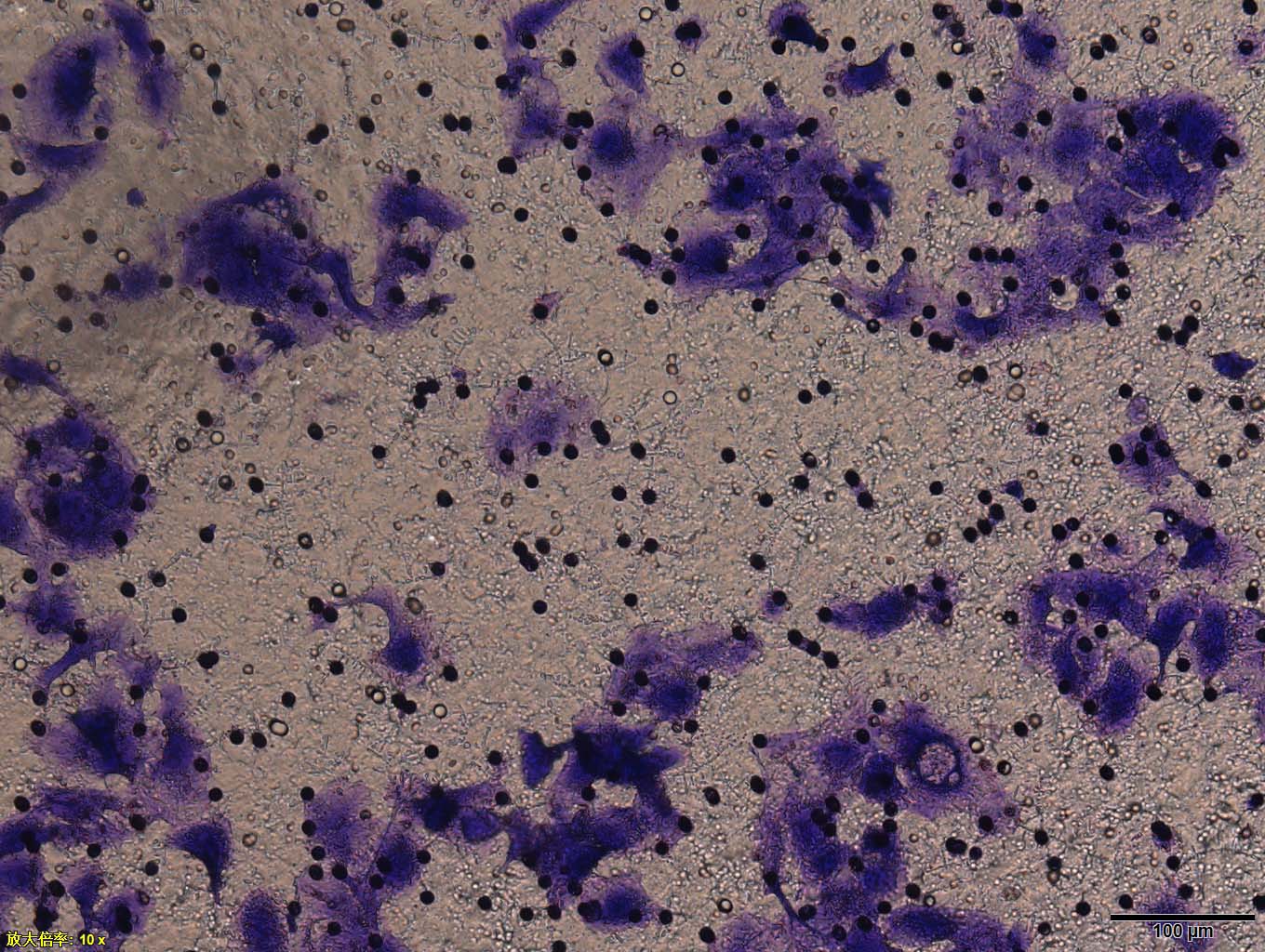

Supplement: S5 Fig — (ZIP) [file pone.0195844.s005.zip › S5_Fig5_File/S5_Fig5C_shCtrl í┴200 (3).jpg]

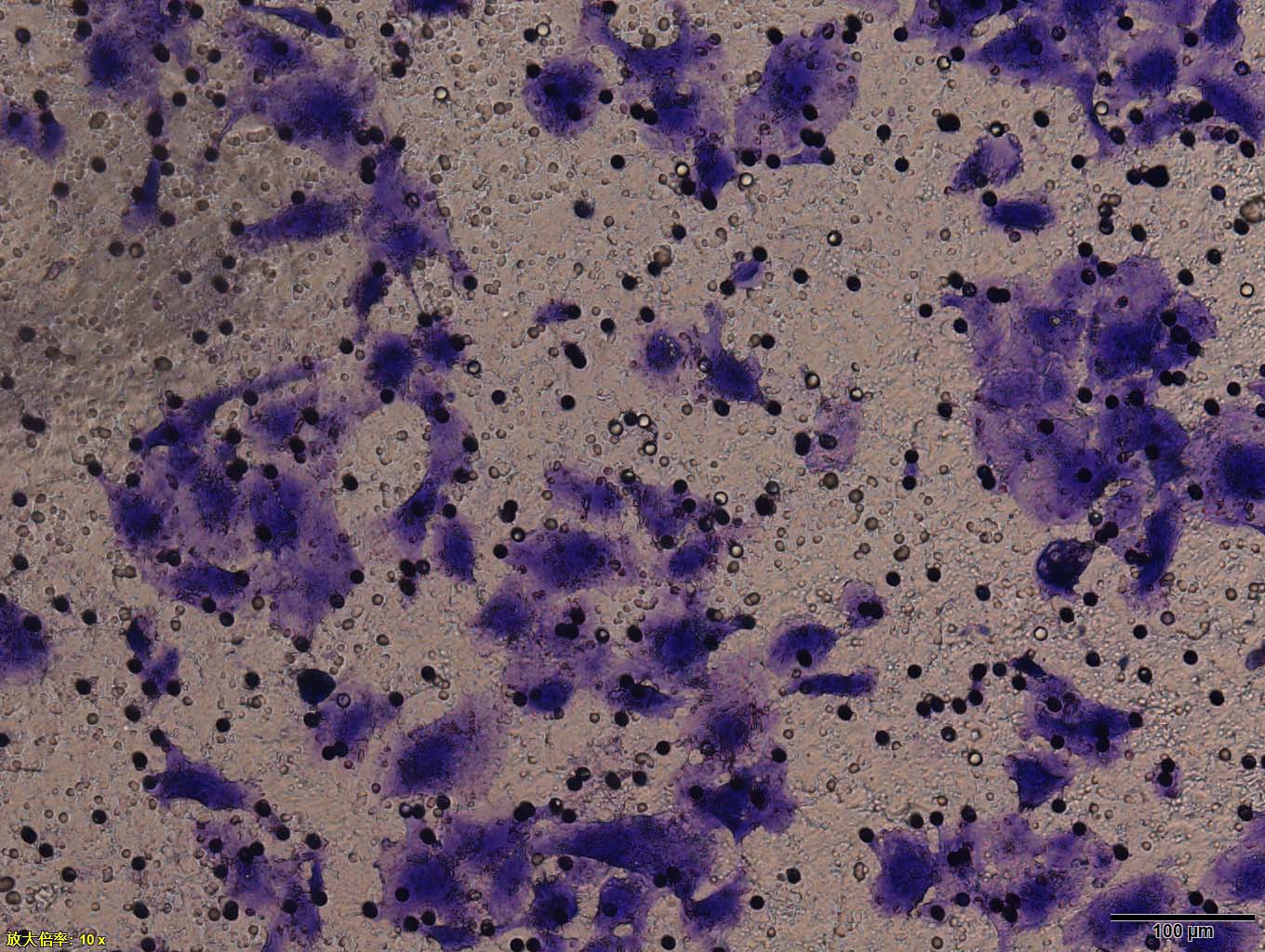

Supplement: S5 Fig — (ZIP) [file pone.0195844.s005.zip › S5_Fig5_File/S5_Fig5C_shCtrl í┴200 (4).jpg]

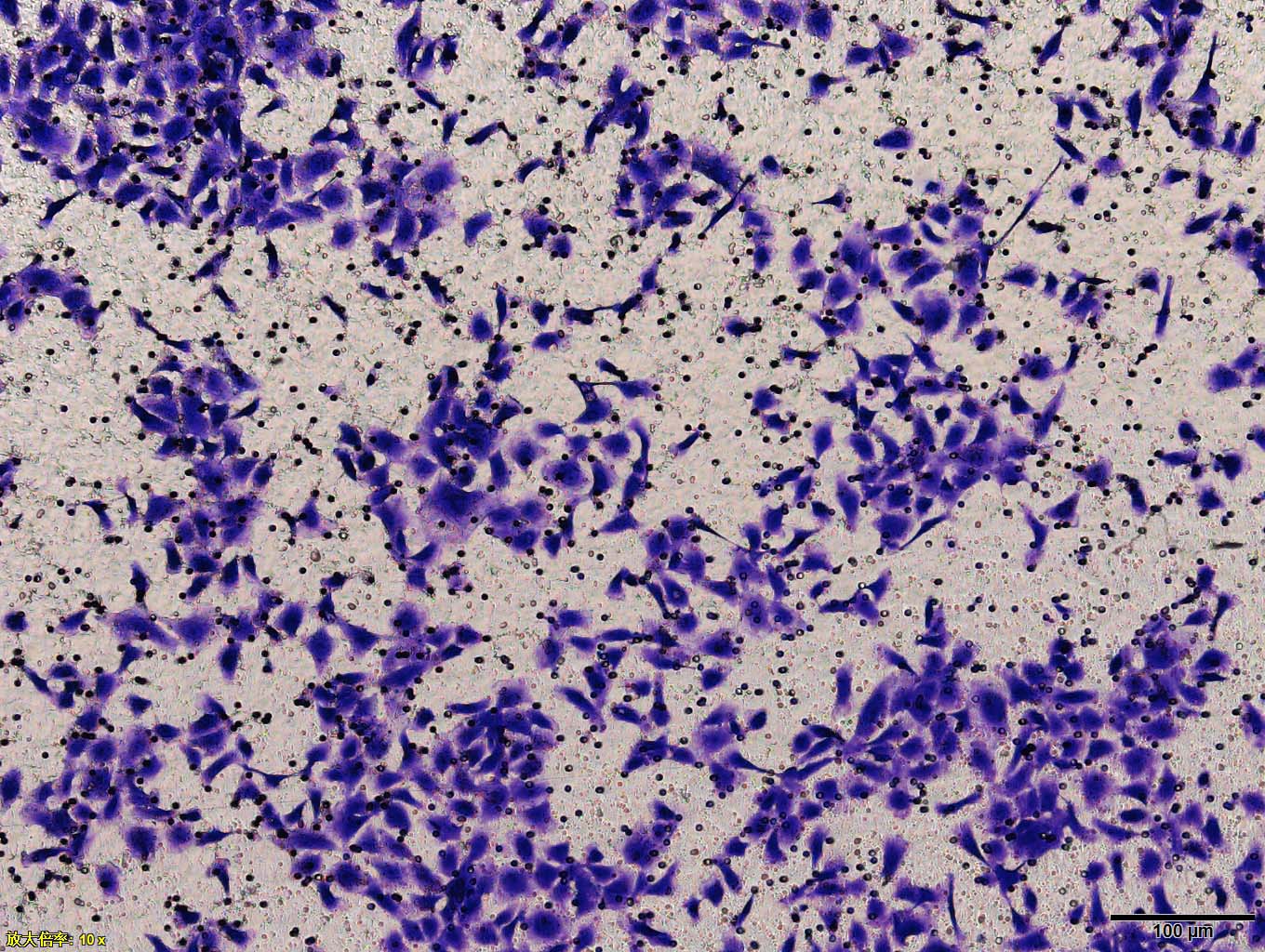

Supplement: S5 Fig — (ZIP) [file pone.0195844.s005.zip › S5_Fig5_File/S5_Fig5C_shVDR í┴100.jpg]

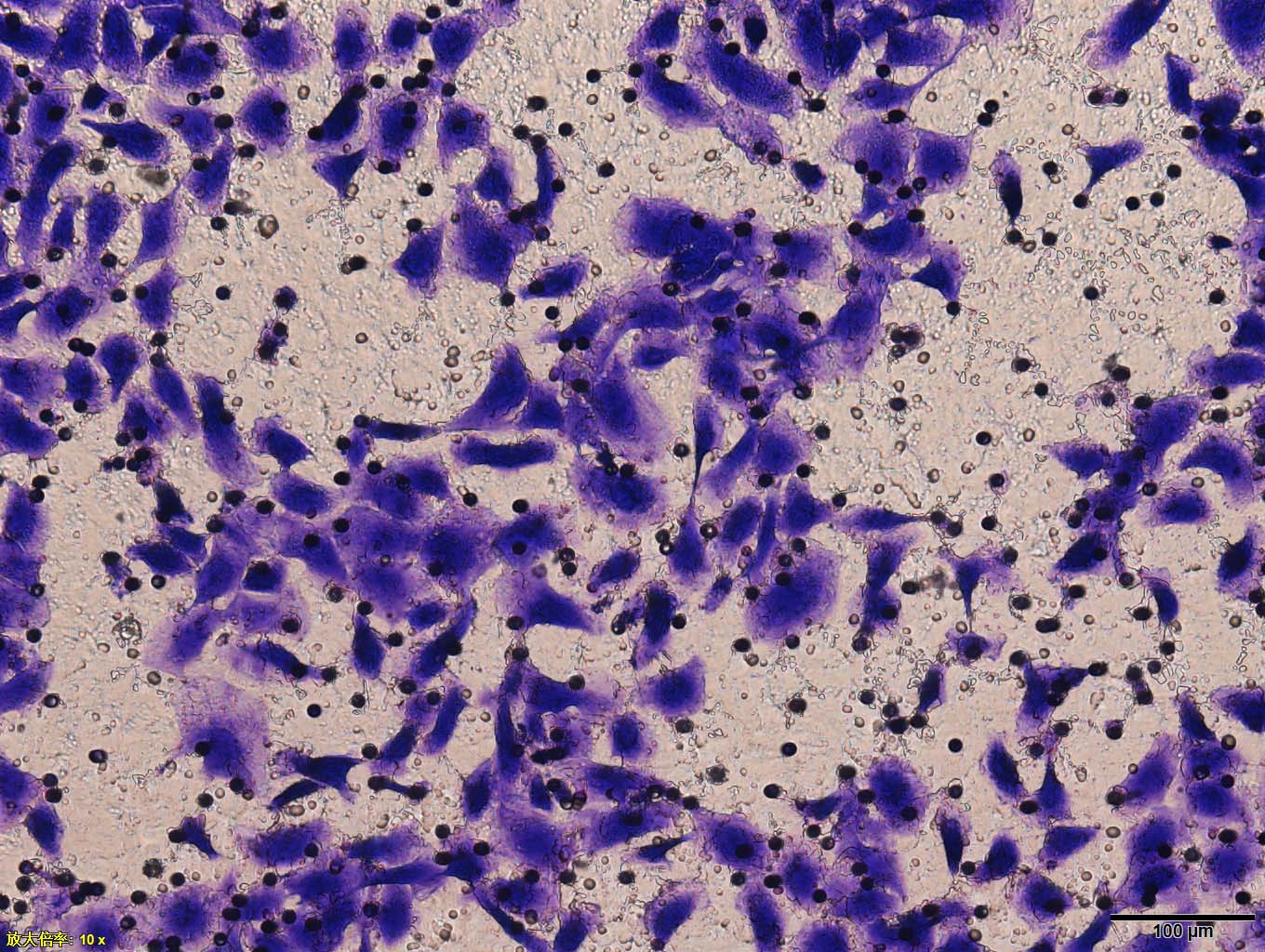

Supplement: S5 Fig — (ZIP) [file pone.0195844.s005.zip › S5_Fig5_File/S5_Fig5C_shVDR í┴200 (1).jpg]

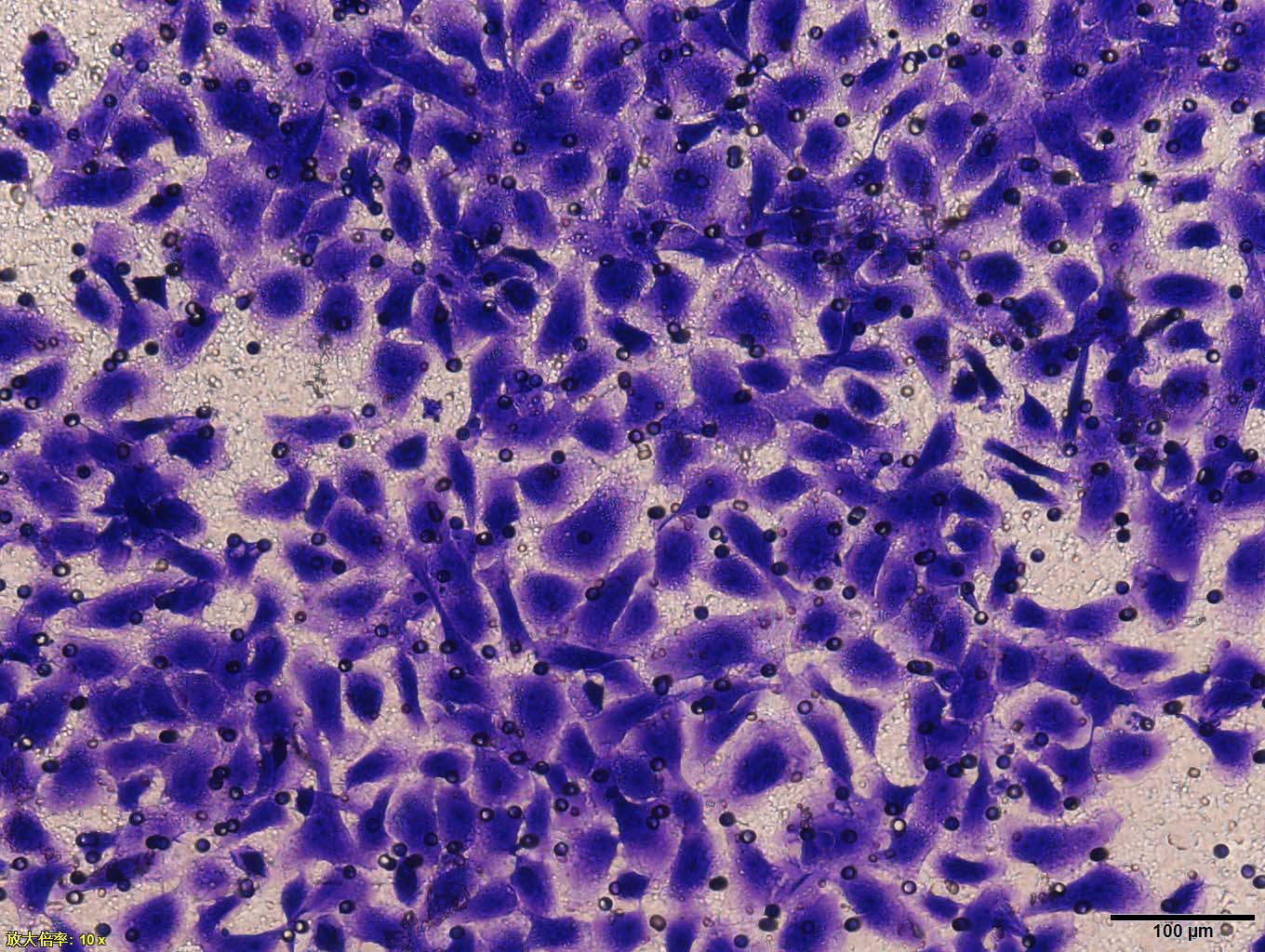

Supplement: S5 Fig — (ZIP) [file pone.0195844.s005.zip › S5_Fig5_File/S5_Fig5C_shVDR í┴200 (2).jpg]

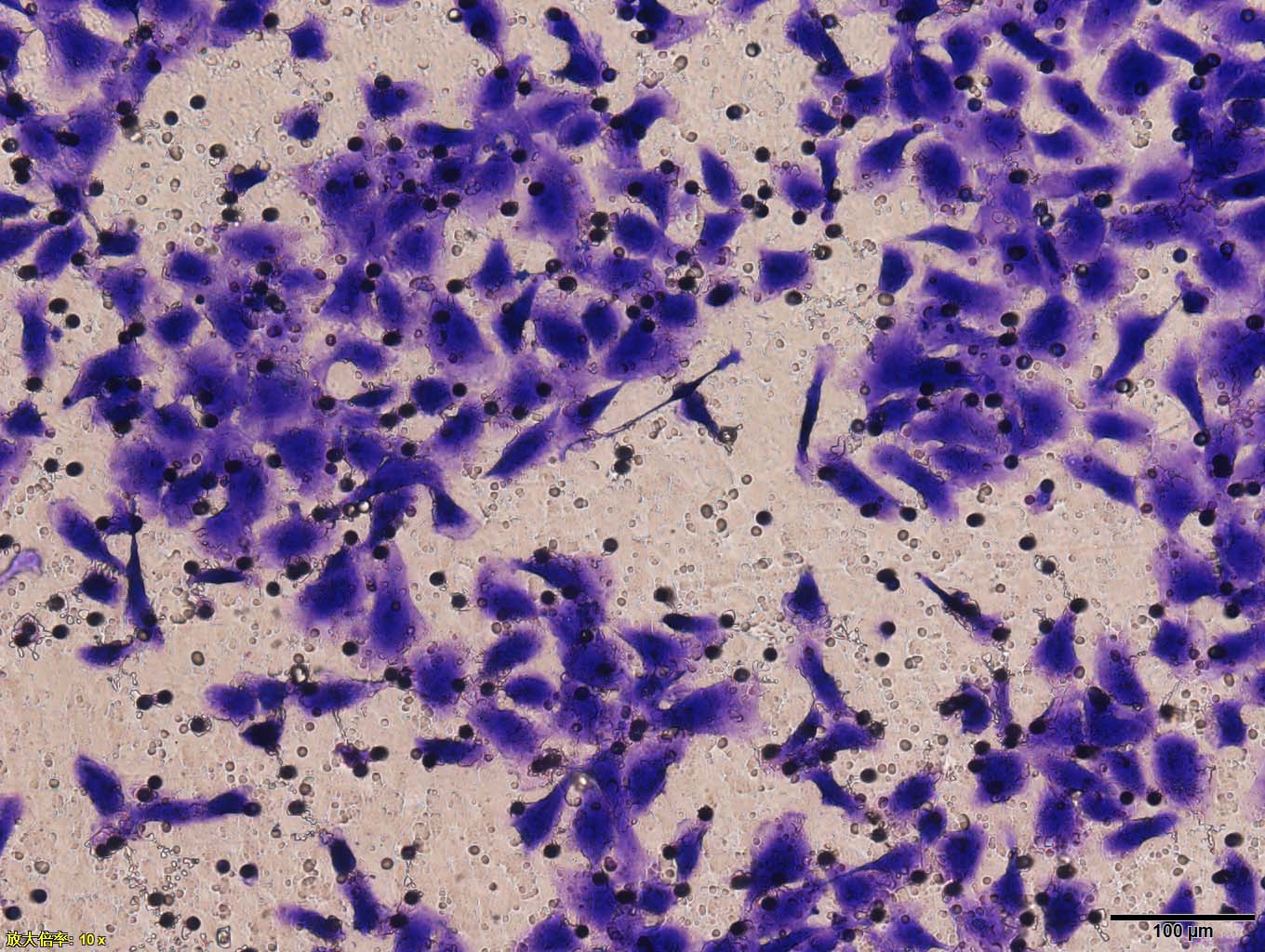

Supplement: S5 Fig — (ZIP) [file pone.0195844.s005.zip › S5_Fig5_File/S5_Fig5C_shVDR í┴200 (3).jpg]

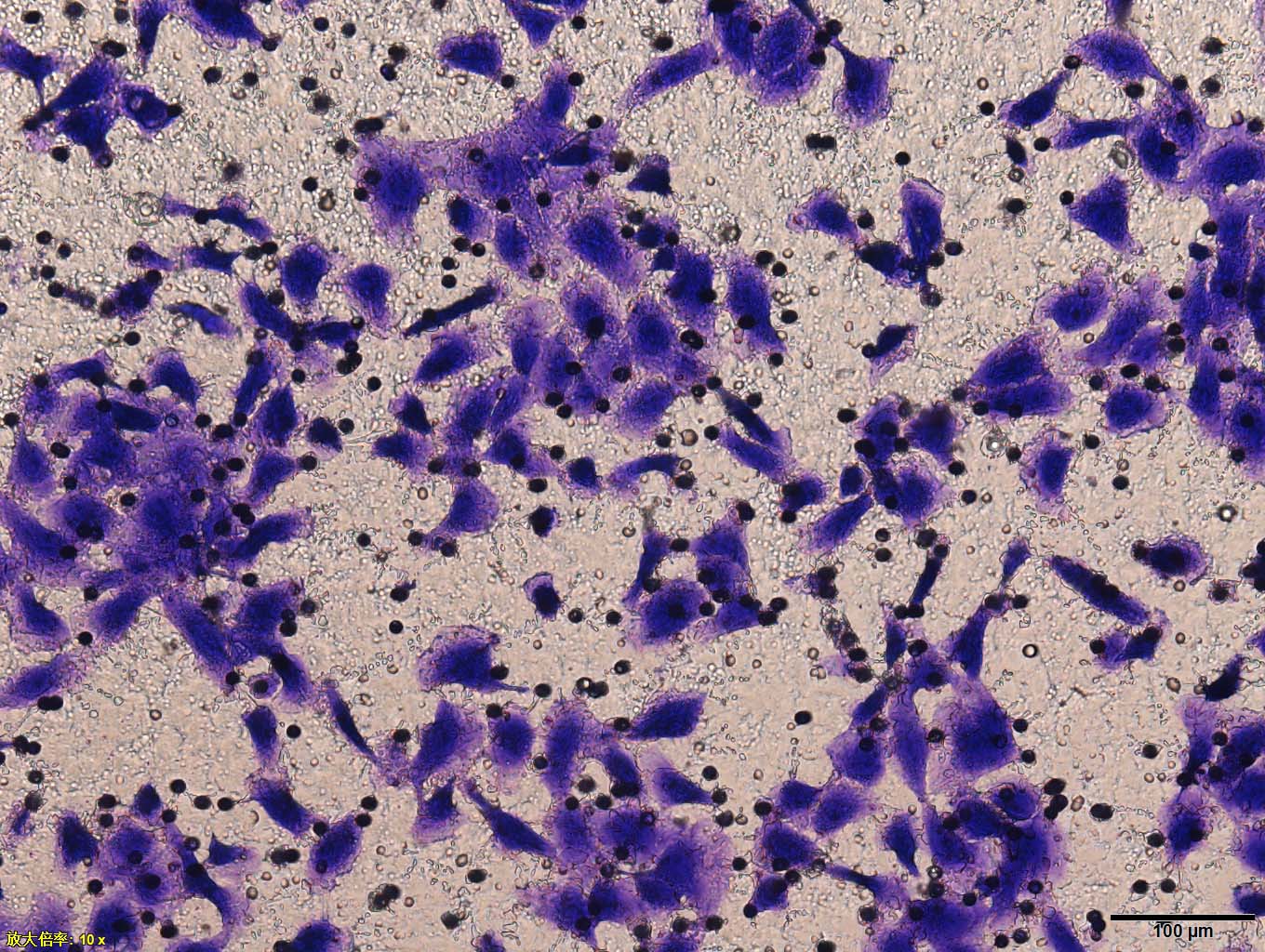

Supplement: S5 Fig — (ZIP) [file pone.0195844.s005.zip › S5_Fig5_File/S5_Fig5C_shVDR í┴200 (4).jpg]

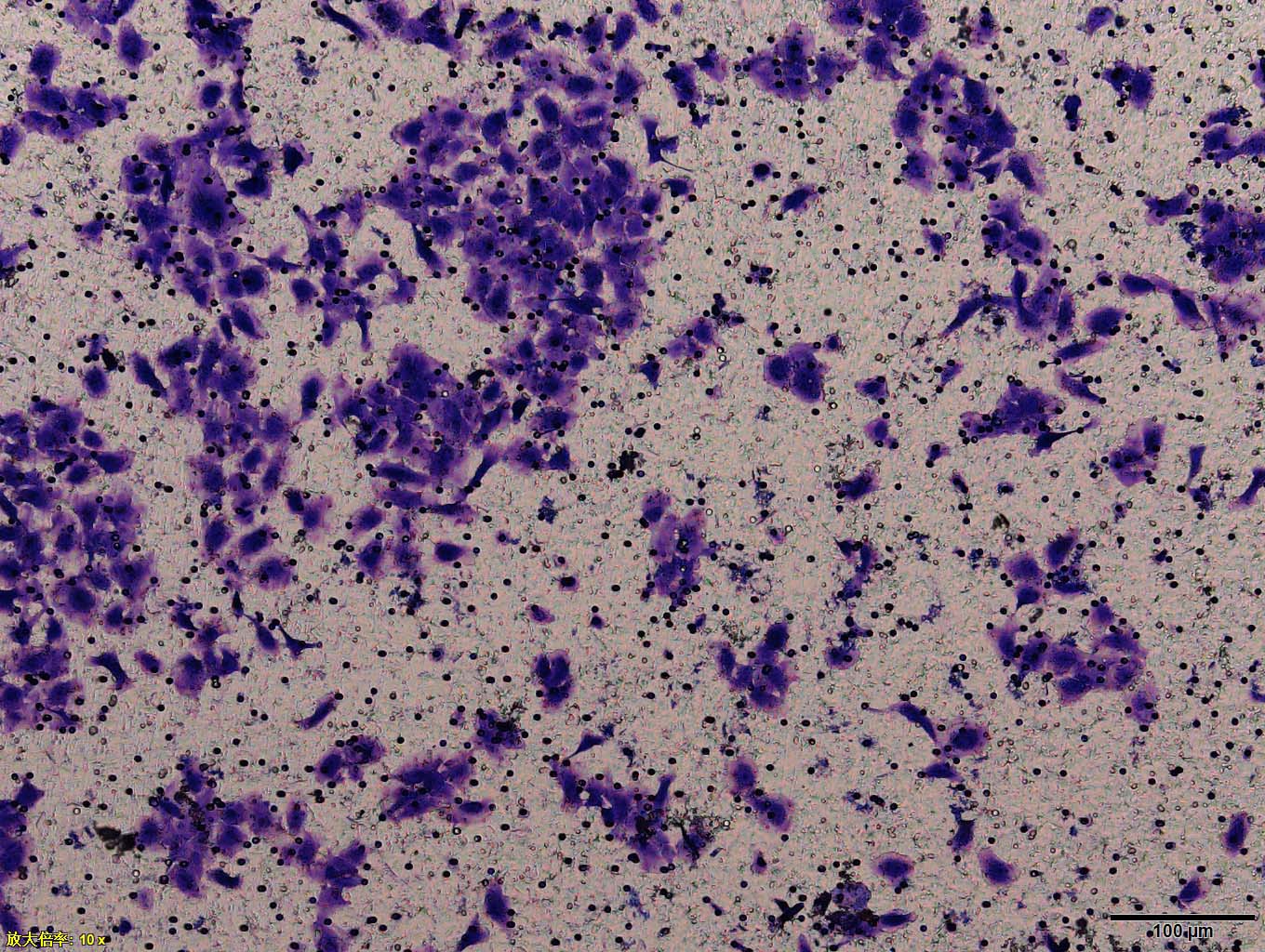

Supplement: S5 Fig — (ZIP) [file pone.0195844.s005.zip › S5_Fig5_File/S5_Fig5C_shVDR+TRPV5 í┴100.jpg]

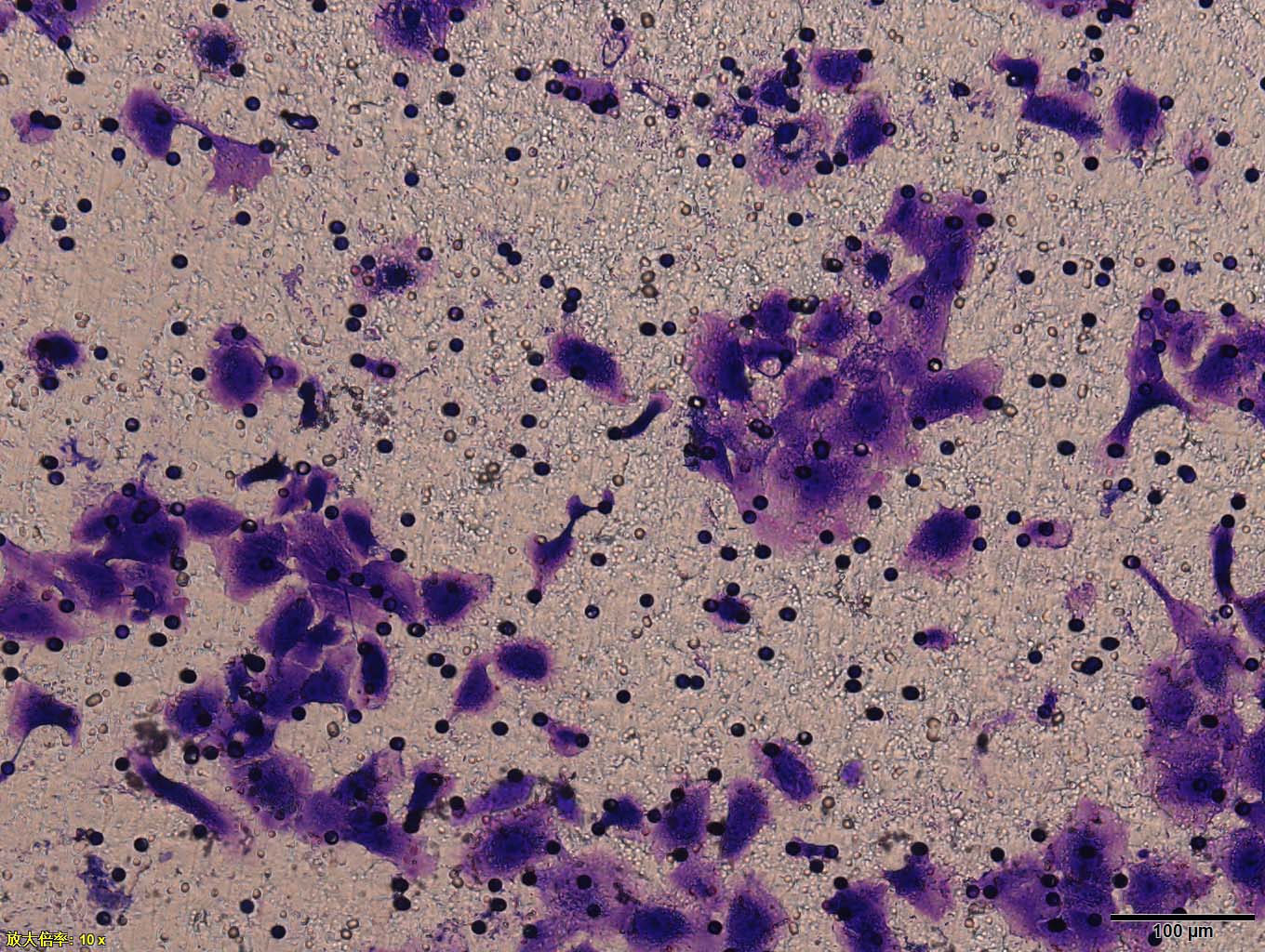

Supplement: S5 Fig — (ZIP) [file pone.0195844.s005.zip › S5_Fig5_File/S5_Fig5C_shVDR+TRPV5 í┴200 (1).jpg]

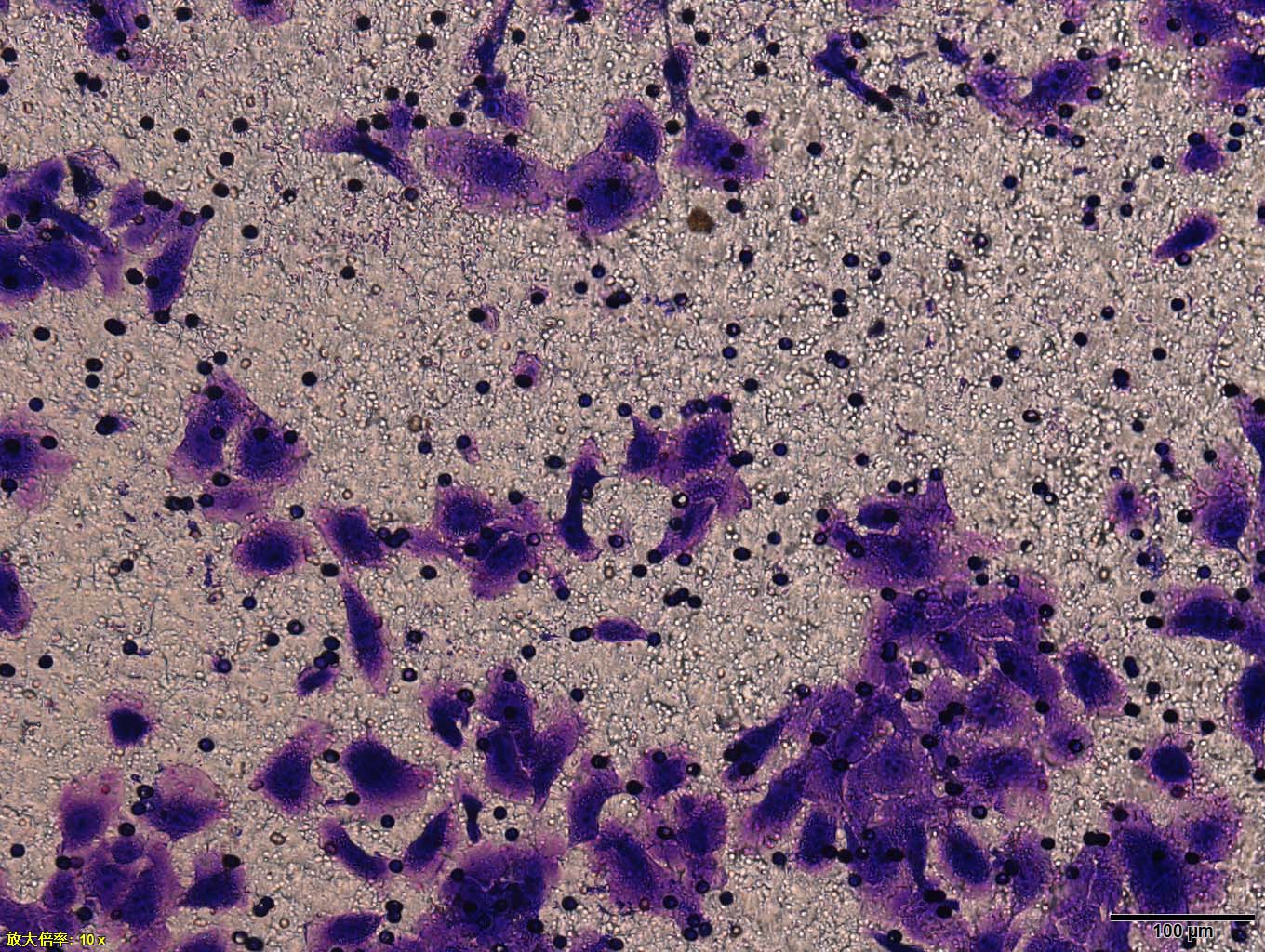

Supplement: S5 Fig — (ZIP) [file pone.0195844.s005.zip › S5_Fig5_File/S5_Fig5C_shVDR+TRPV5 í┴200 (2).jpg]

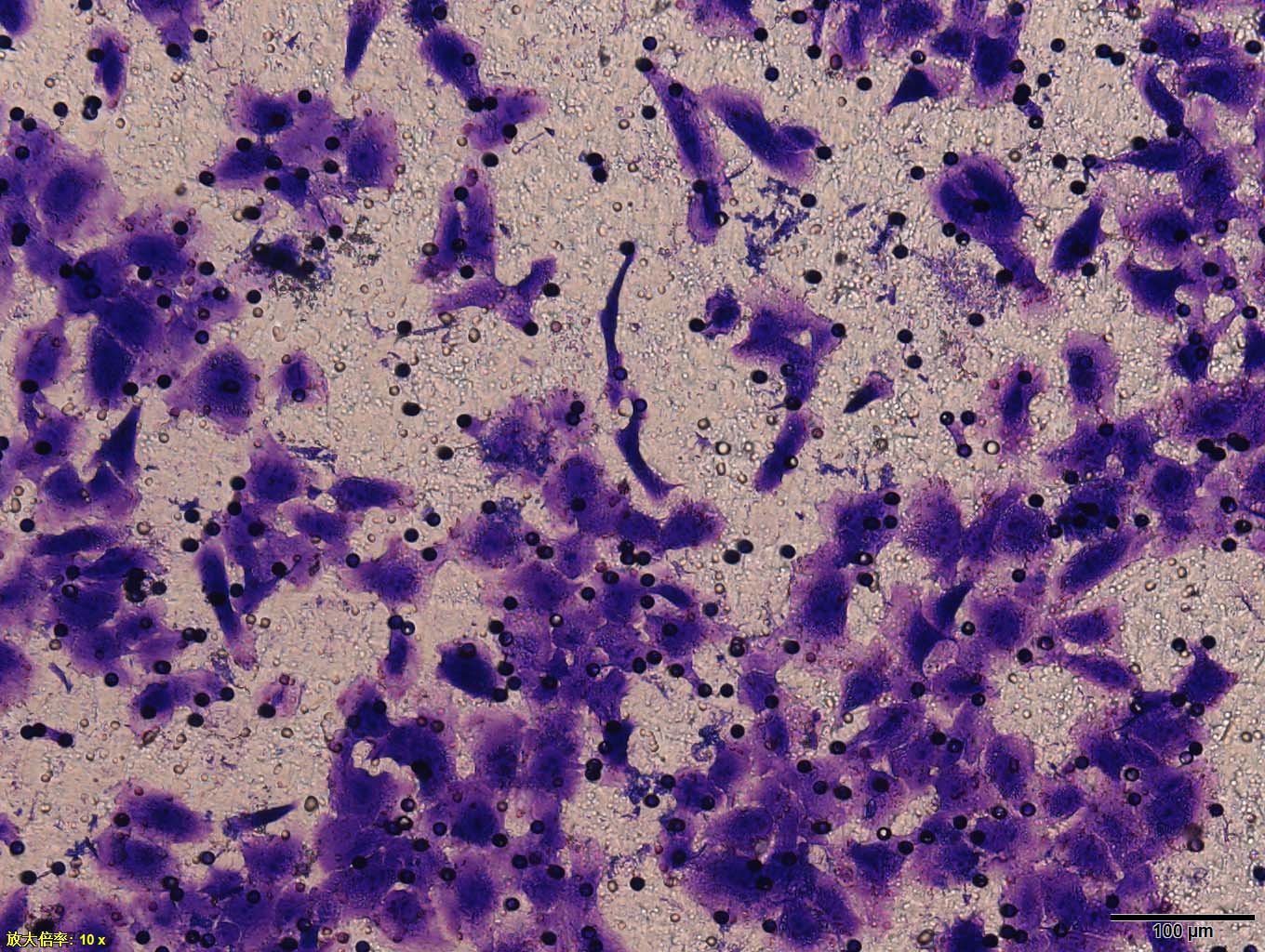

Supplement: S5 Fig — (ZIP) [file pone.0195844.s005.zip › S5_Fig5_File/S5_Fig5C_shVDR+TRPV5 í┴200 (3).jpg]

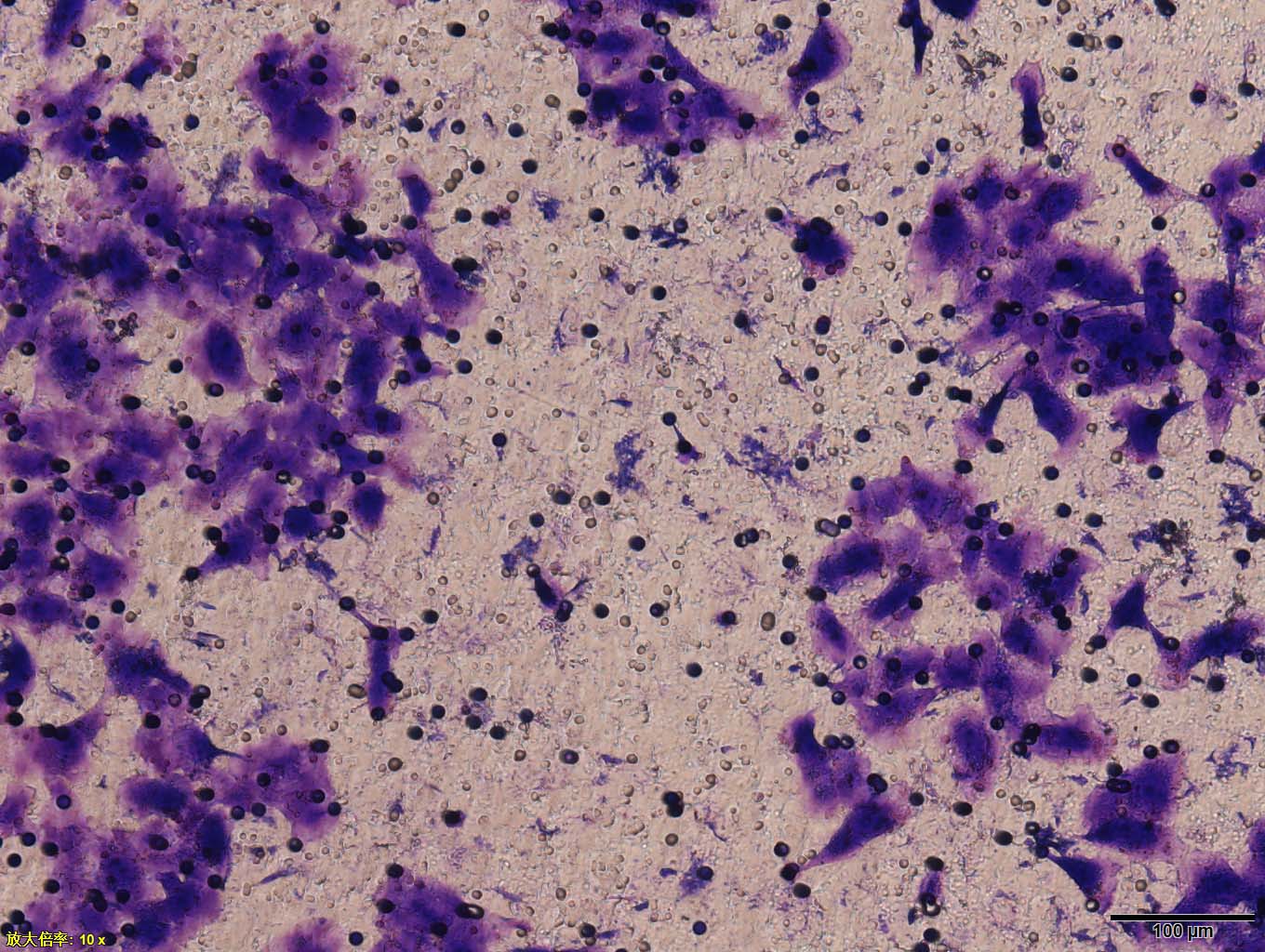

Supplement: S5 Fig — (ZIP) [file pone.0195844.s005.zip › S5_Fig5_File/S5_Fig5C_shVDR+TRPV5 í┴200 (4).jpg]

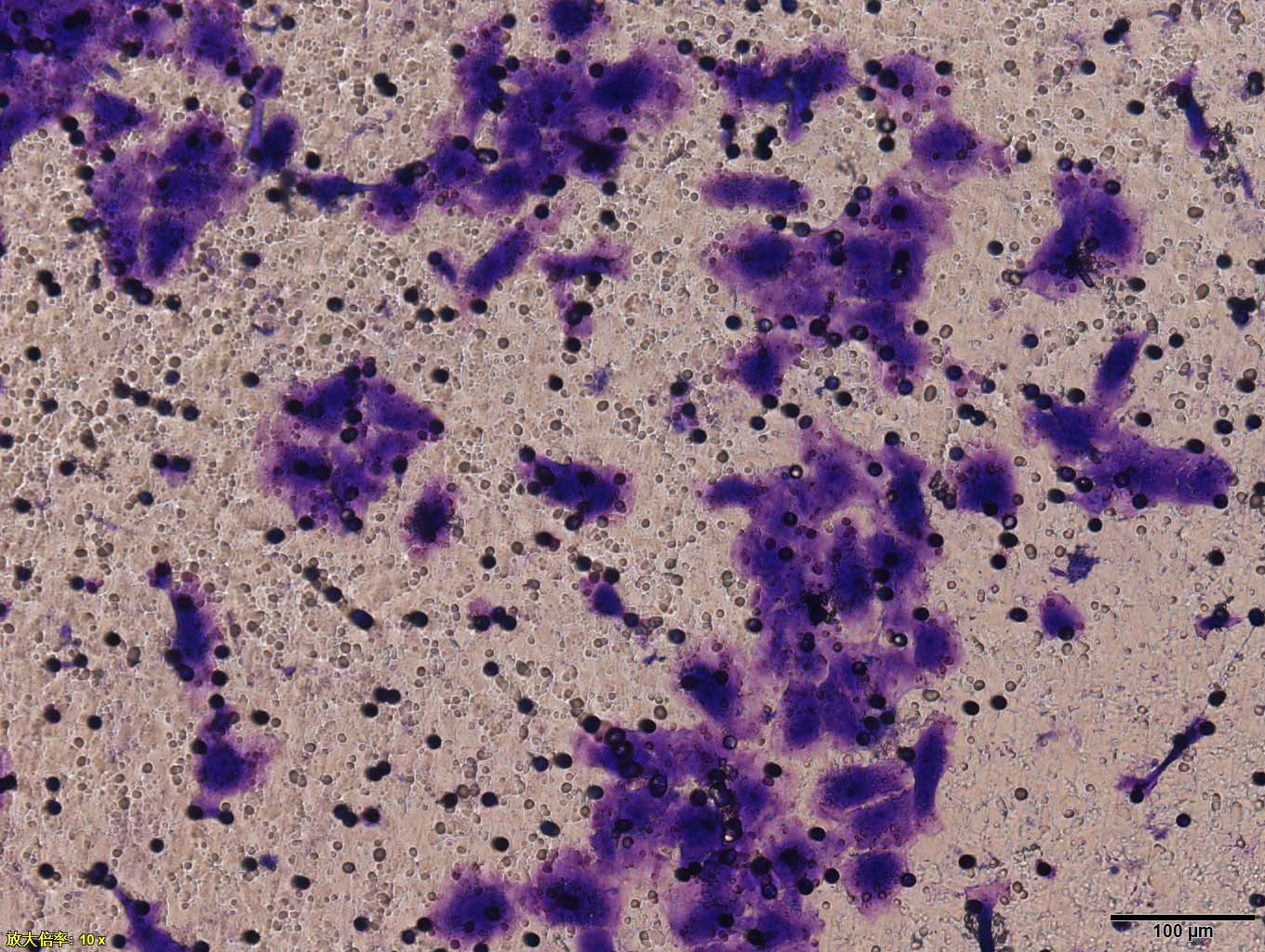

Supplement: S5 Fig — (ZIP) [file pone.0195844.s005.zip › S5_Fig5_File/S5_Fig5C_shVDR+TRPV5 í┴200 (5).jpg]

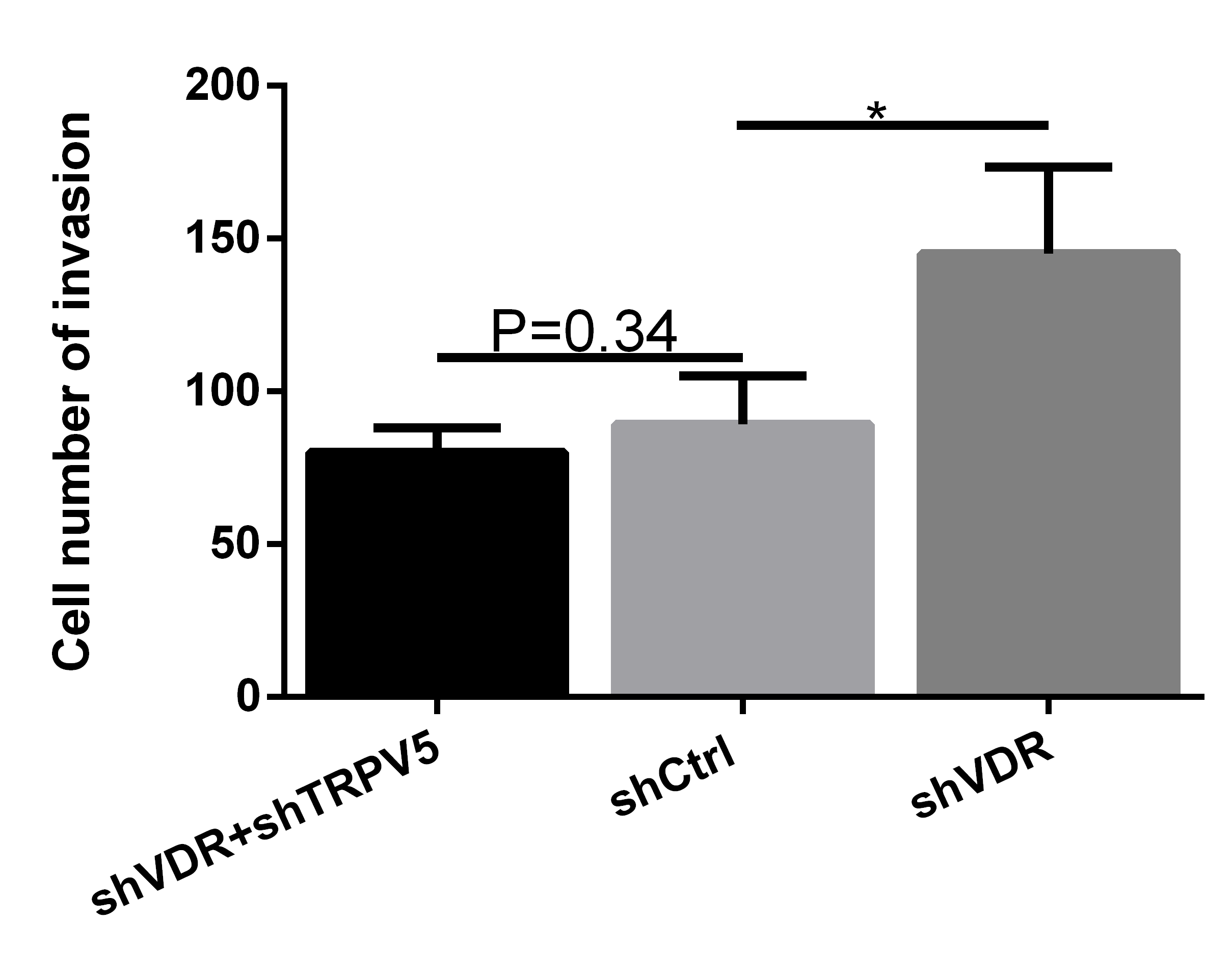

Supplement: S5 Fig — (ZIP) [file pone.0195844.s005.zip › S5_Fig5_File/S5_Fig5D_RT-PCR.tif]

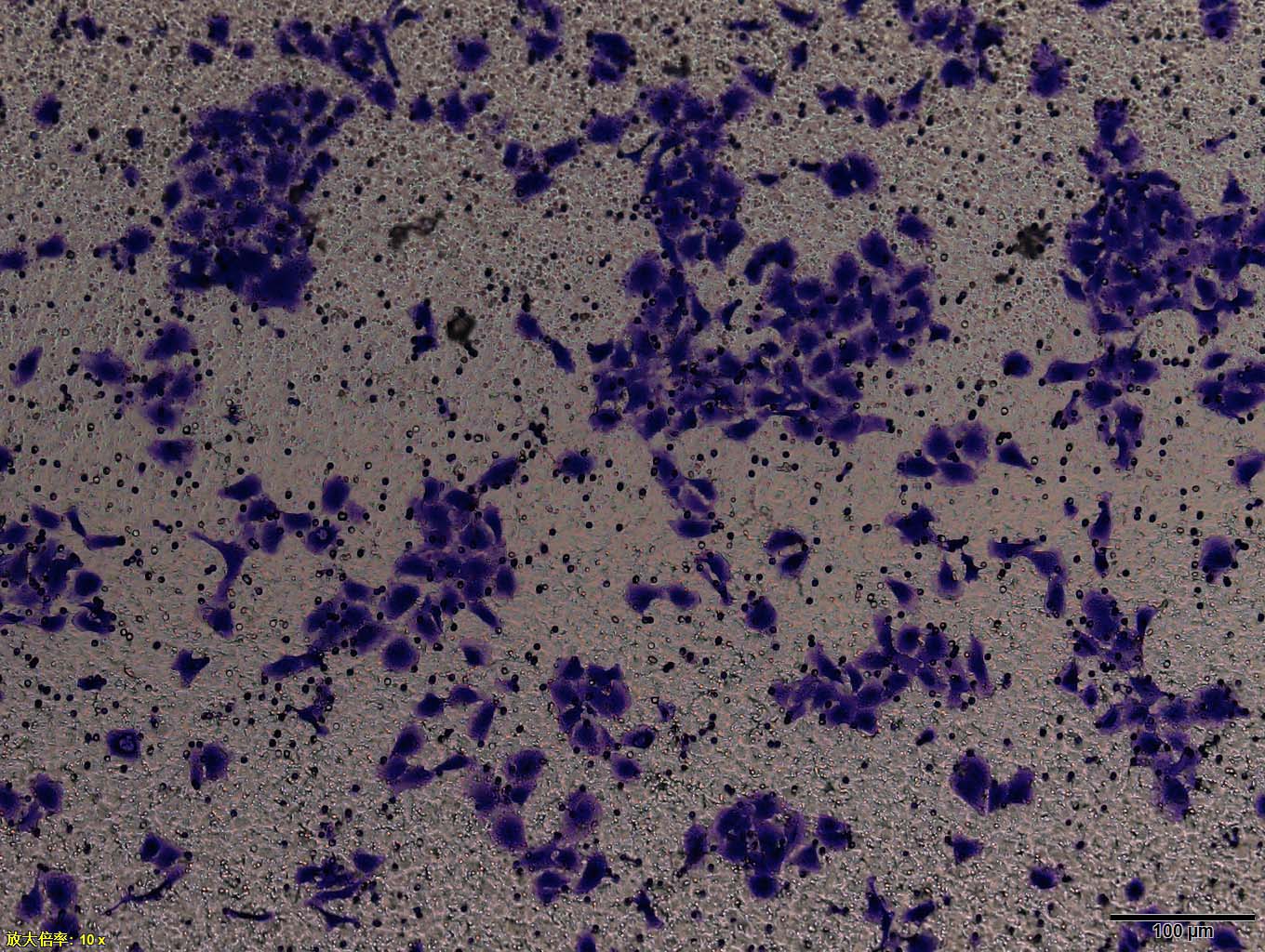

Supplement: S5 Fig — (ZIP) [file pone.0195844.s005.zip › S5_Fig5_File/S5_Fig5D_shCtrl í┴100.jpg]

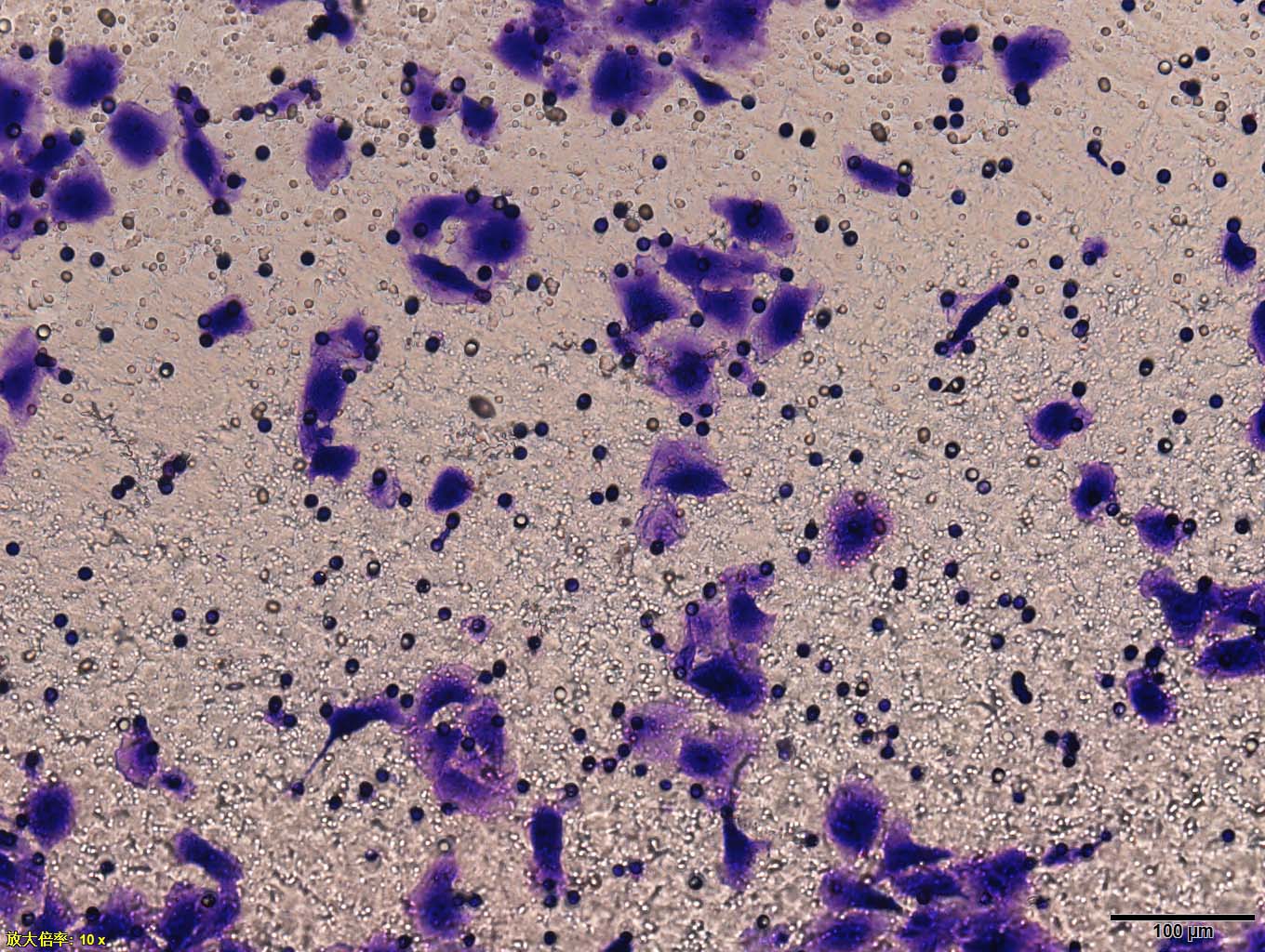

Supplement: S5 Fig — (ZIP) [file pone.0195844.s005.zip › S5_Fig5_File/S5_Fig5D_shCtrl í┴200 (1).jpg]

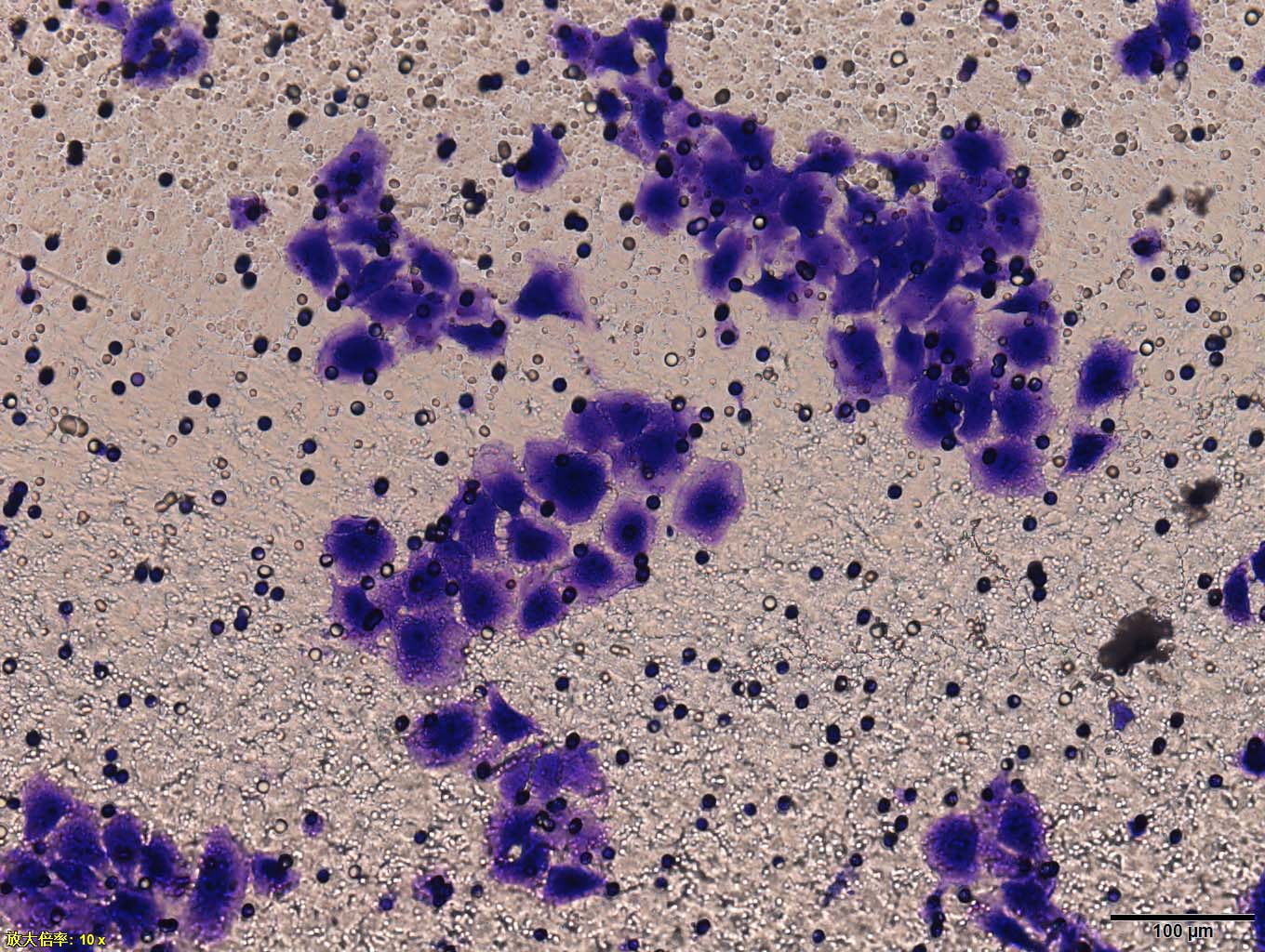

Supplement: S5 Fig — (ZIP) [file pone.0195844.s005.zip › S5_Fig5_File/S5_Fig5D_shCtrl í┴200 (2).jpg]

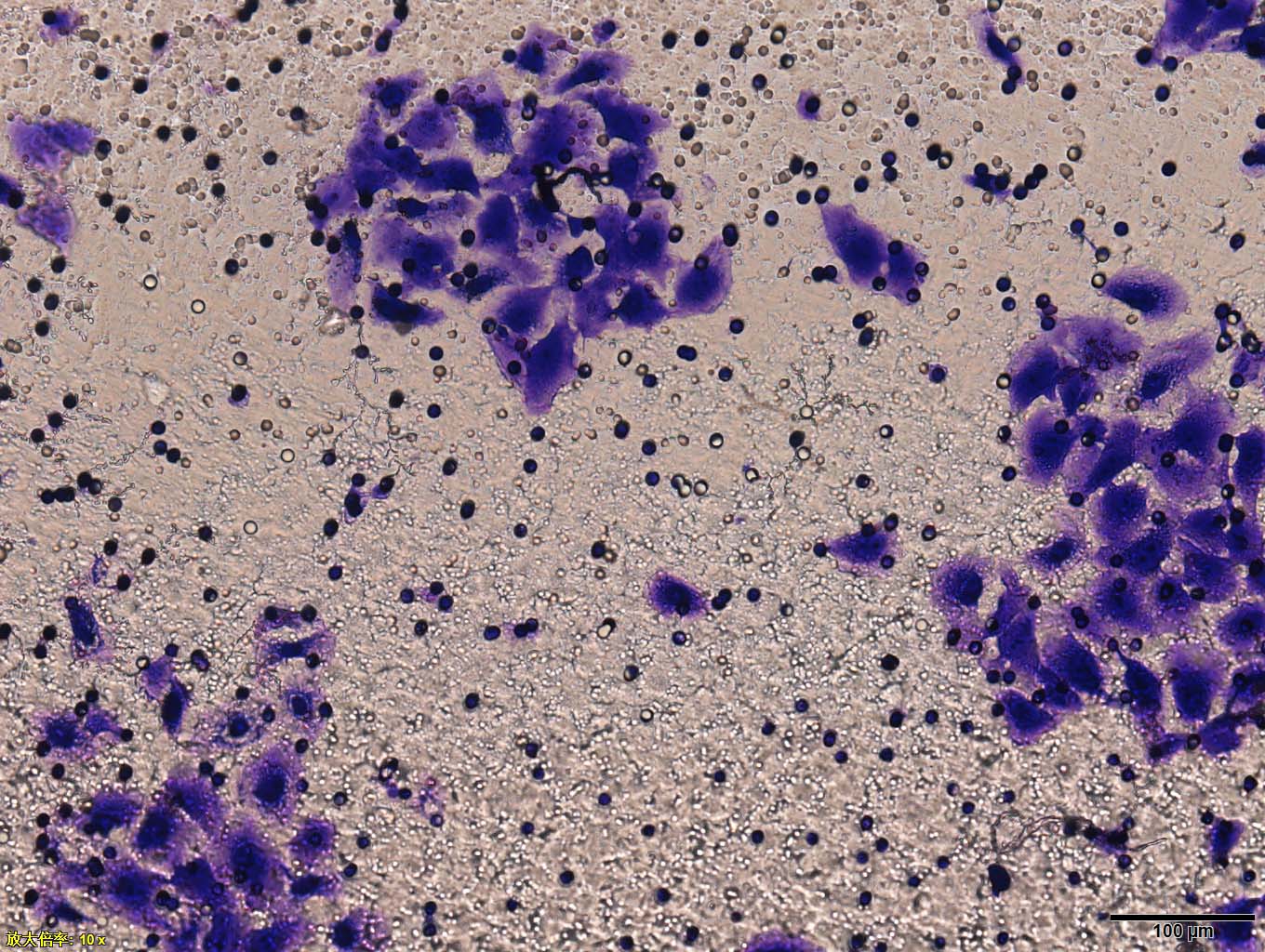

Supplement: S5 Fig — (ZIP) [file pone.0195844.s005.zip › S5_Fig5_File/S5_Fig5D_shCtrl í┴200 (3).jpg]

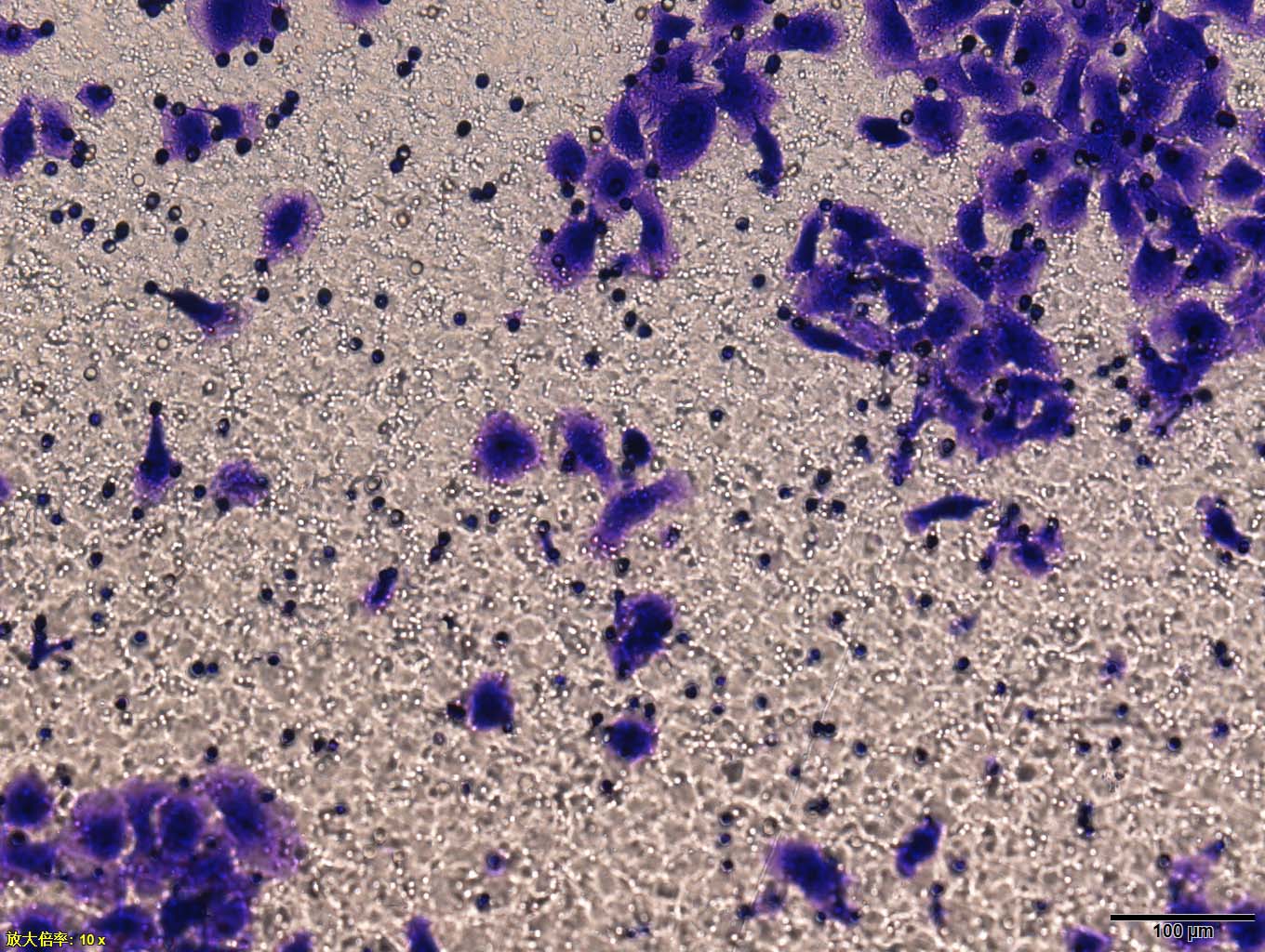

Supplement: S5 Fig — (ZIP) [file pone.0195844.s005.zip › S5_Fig5_File/S5_Fig5D_shCtrl í┴200 (4).jpg]

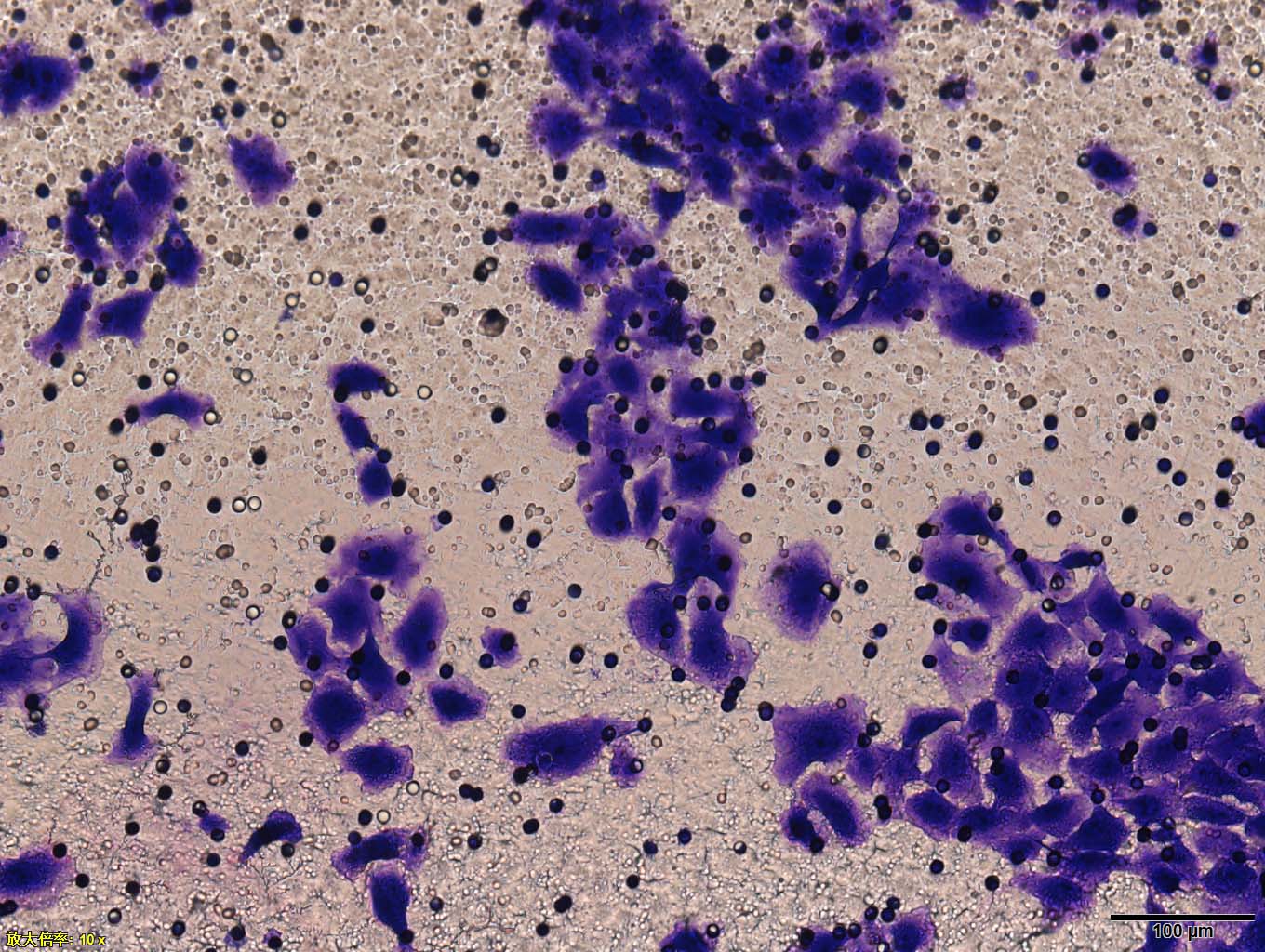

Supplement: S5 Fig — (ZIP) [file pone.0195844.s005.zip › S5_Fig5_File/S5_Fig5D_shCtrl í┴200 (5).jpg]

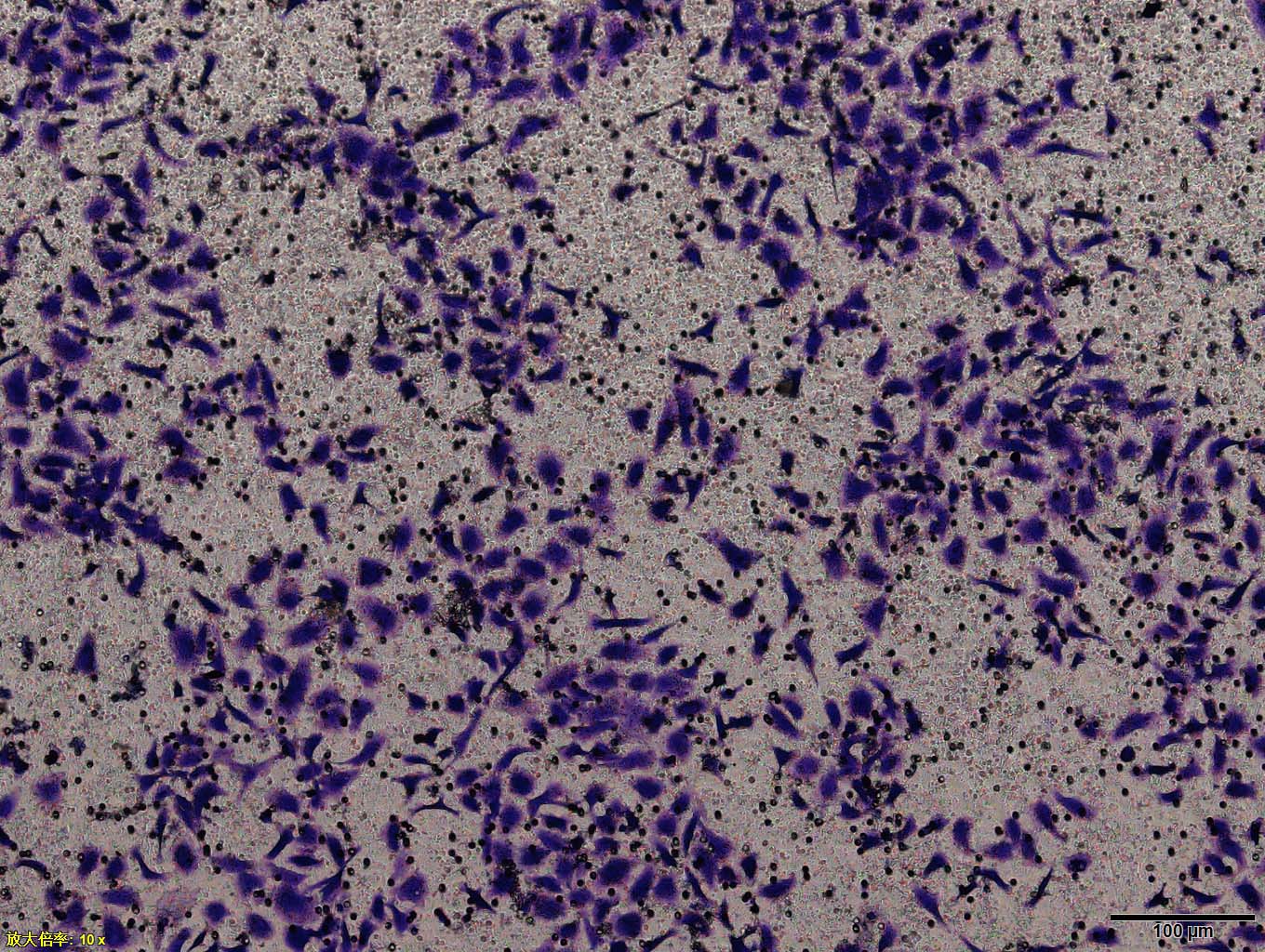

Supplement: S5 Fig — (ZIP) [file pone.0195844.s005.zip › S5_Fig5_File/S5_Fig5D_shVDR í┴100.jpg]

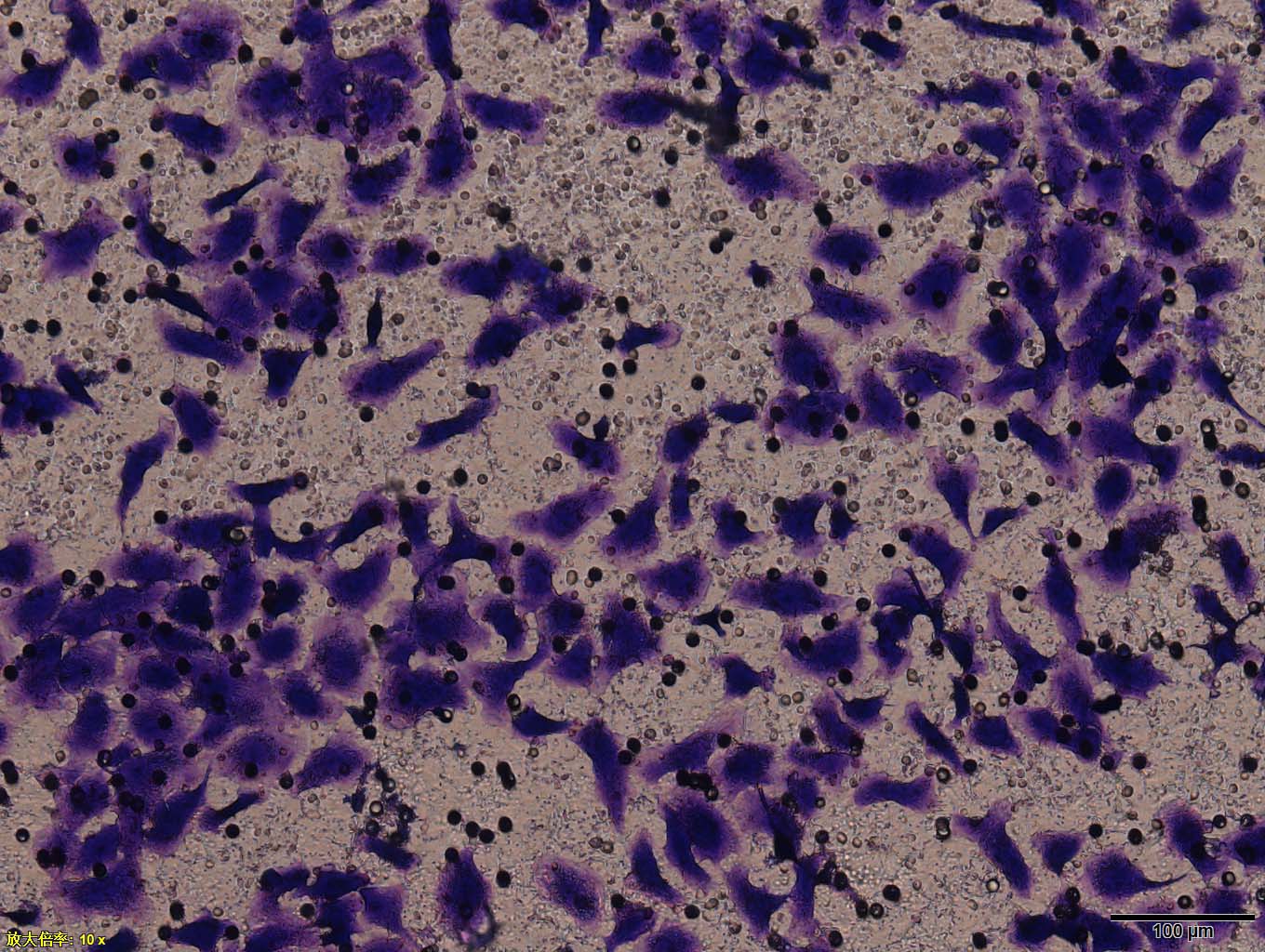

Supplement: S5 Fig — (ZIP) [file pone.0195844.s005.zip › S5_Fig5_File/S5_Fig5D_shVDR í┴200 (1).jpg]

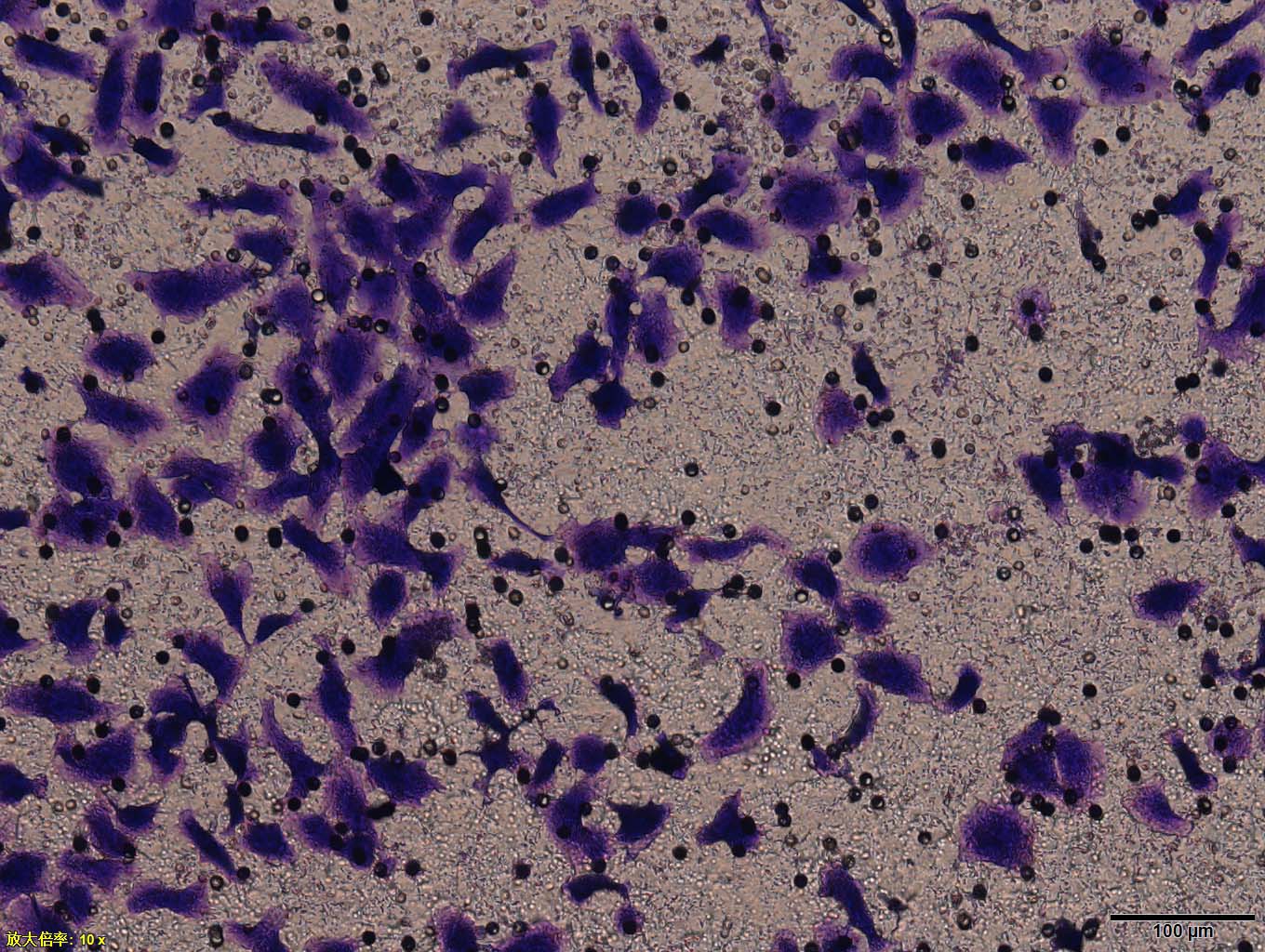

Supplement: S5 Fig — (ZIP) [file pone.0195844.s005.zip › S5_Fig5_File/S5_Fig5D_shVDR í┴200 (2).jpg]
