# Supplementary figures and images for: Vitamin D receptor suppresses proliferation and metastasis in renal cell carcinoma cell lines via regulating the expression of the epithelial Ca2+ channel TRPV5 (part 2 of 2)
Source: PLoS One. 2018 Apr 16;13(4):e0195844. doi: 10.1371/journal.pone.0195844 (PMC5901920; doi:10.1371/journal.pone.0195844)

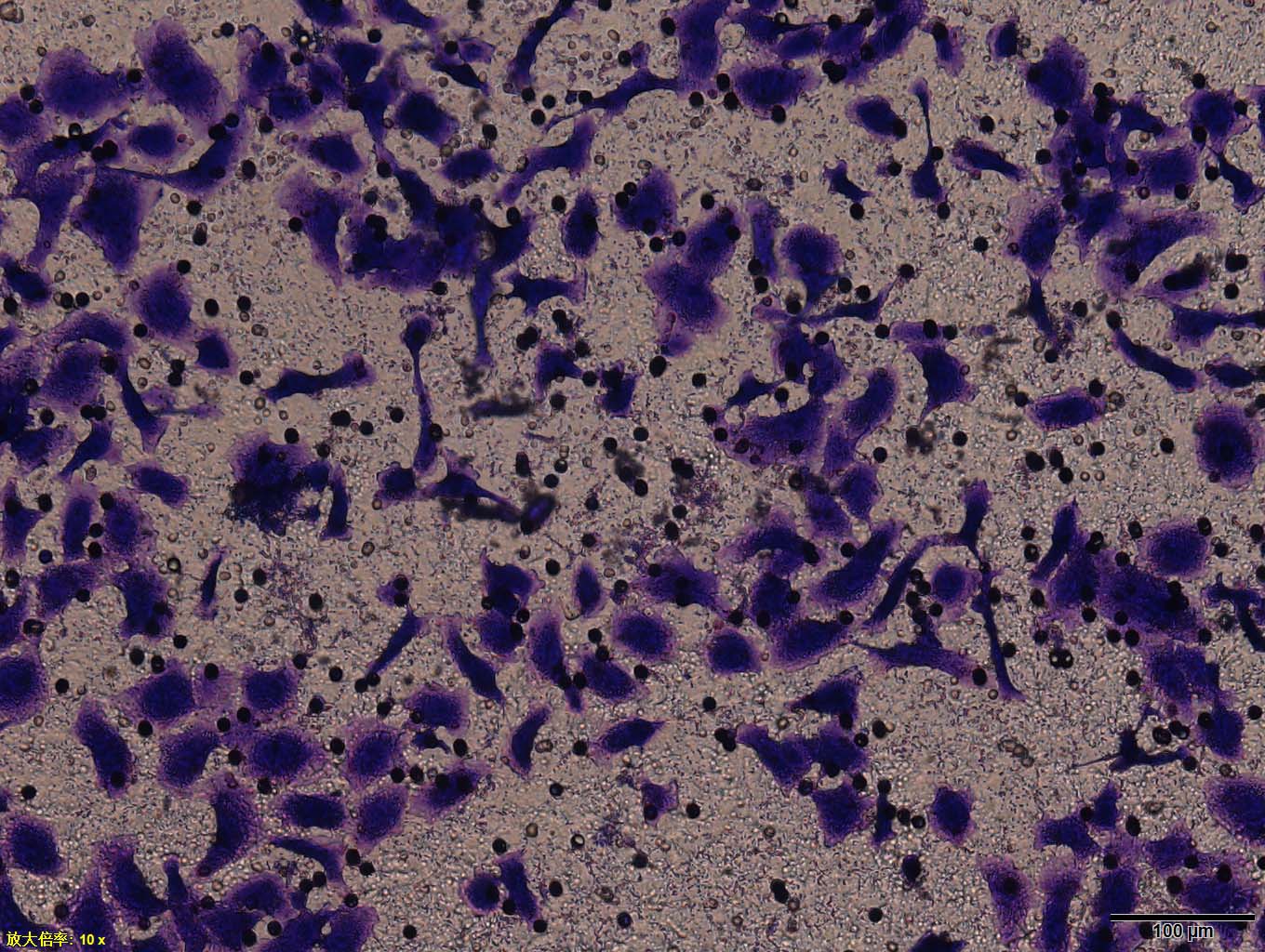

Supplement: S5 Fig — (ZIP) [file pone.0195844.s005.zip › S5_Fig5_File/S5_Fig5D_shVDR í┴200 (3).jpg]

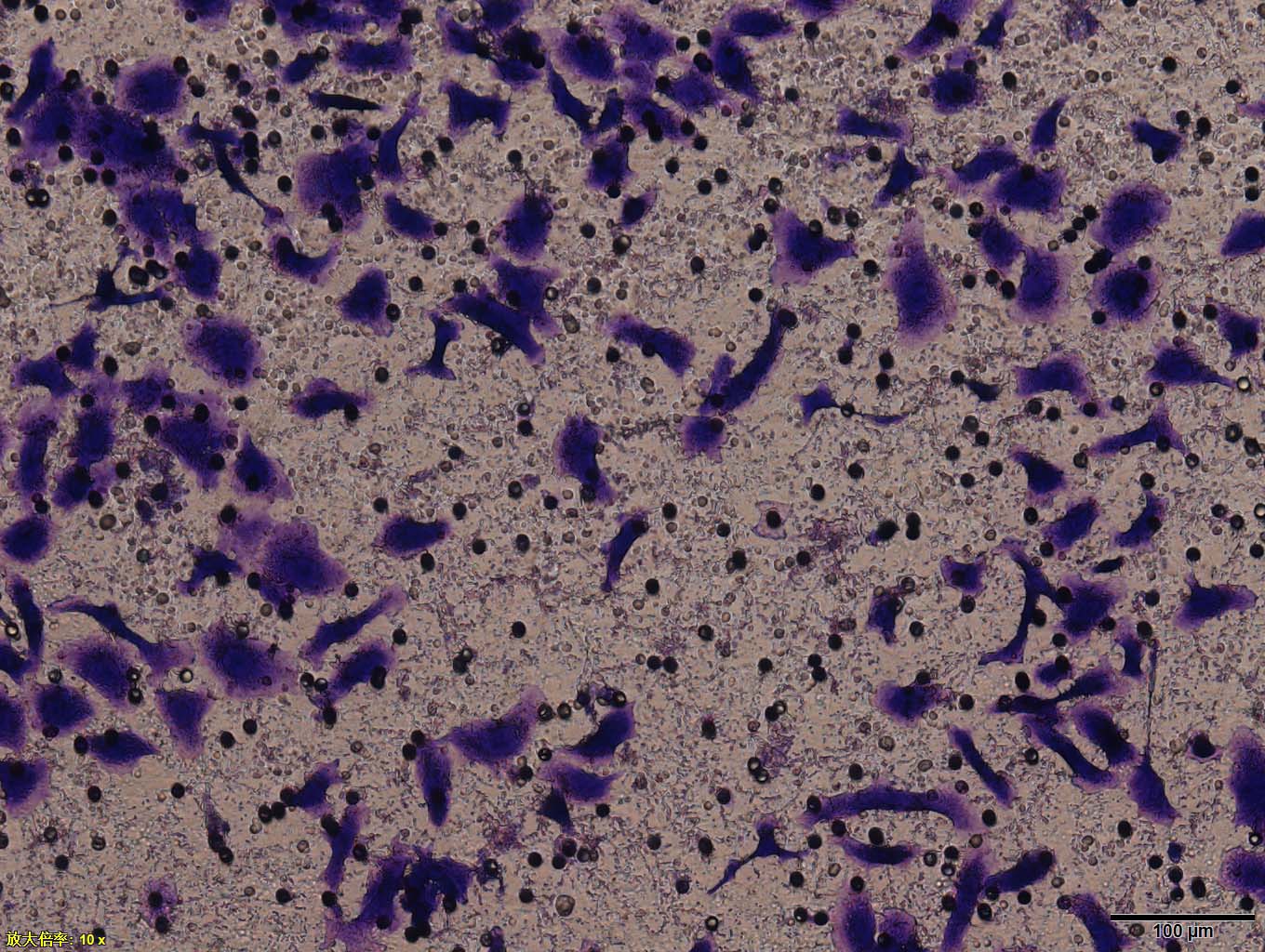

Supplement: S5 Fig — (ZIP) [file pone.0195844.s005.zip › S5_Fig5_File/S5_Fig5D_shVDR í┴200 (4).jpg]

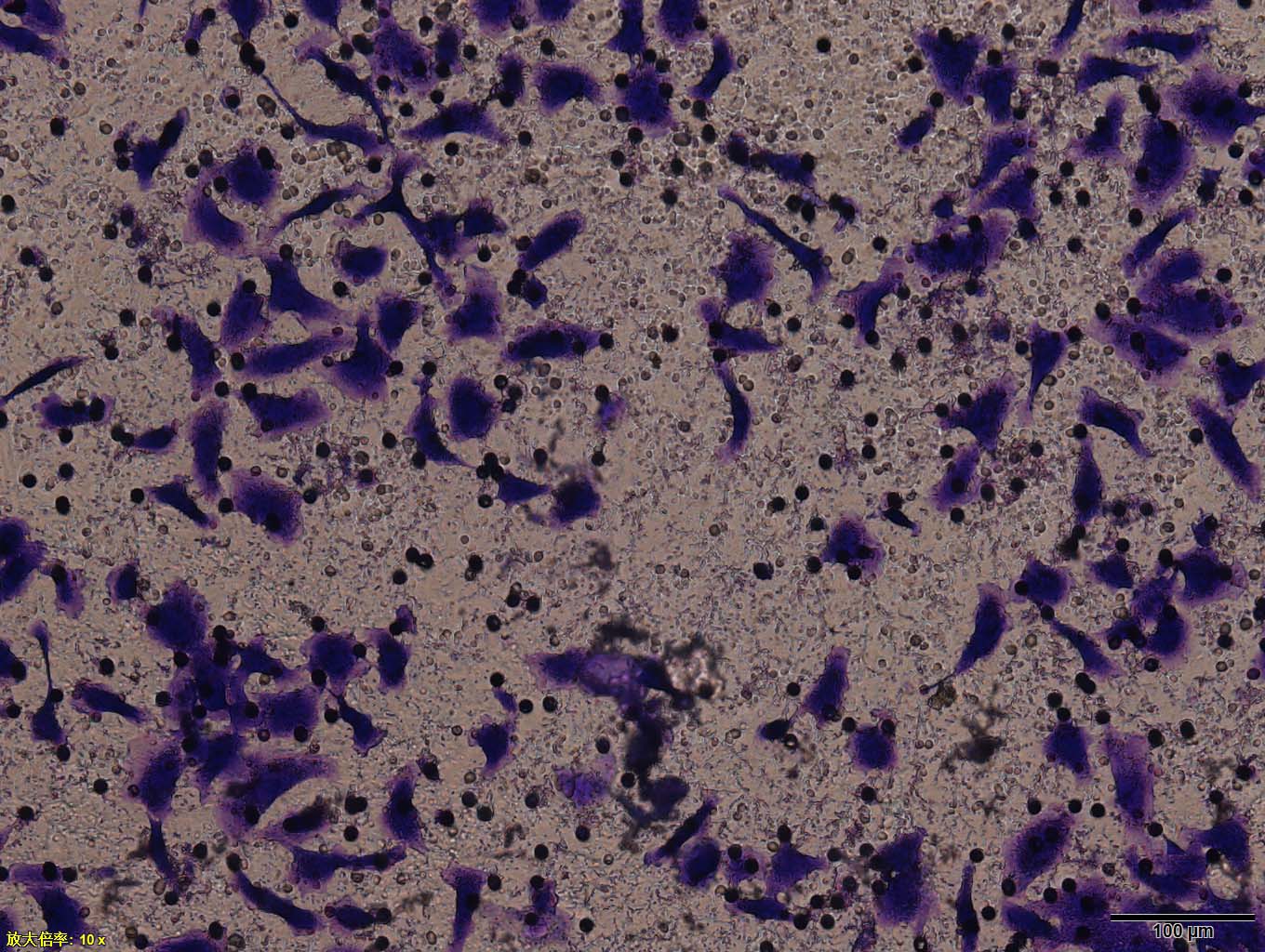

Supplement: S5 Fig — (ZIP) [file pone.0195844.s005.zip › S5_Fig5_File/S5_Fig5D_shVDR í┴200 (5).jpg]

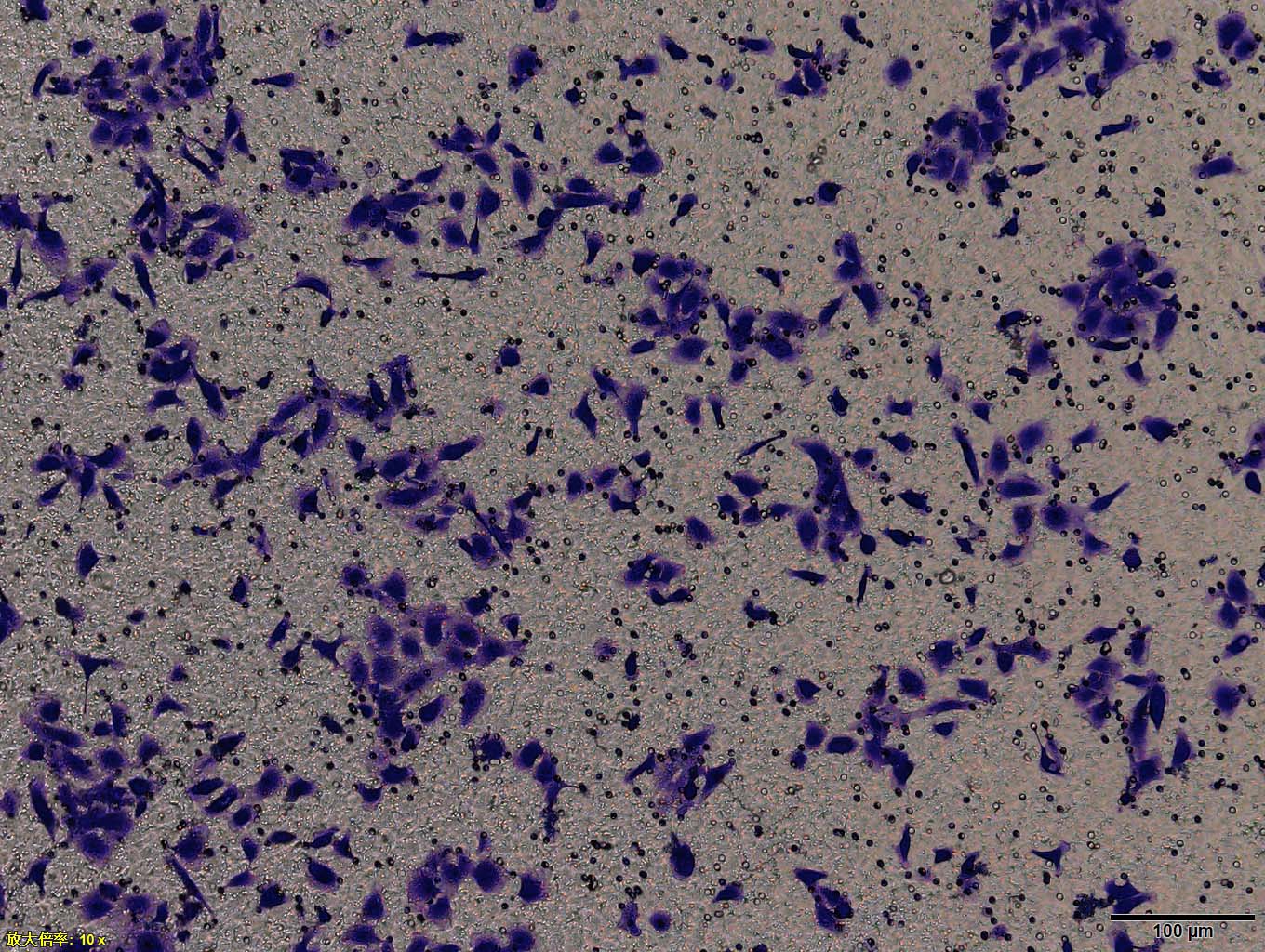

Supplement: S5 Fig — (ZIP) [file pone.0195844.s005.zip › S5_Fig5_File/S5_Fig5D_shVDR+TRPV5 í┴100.jpg]

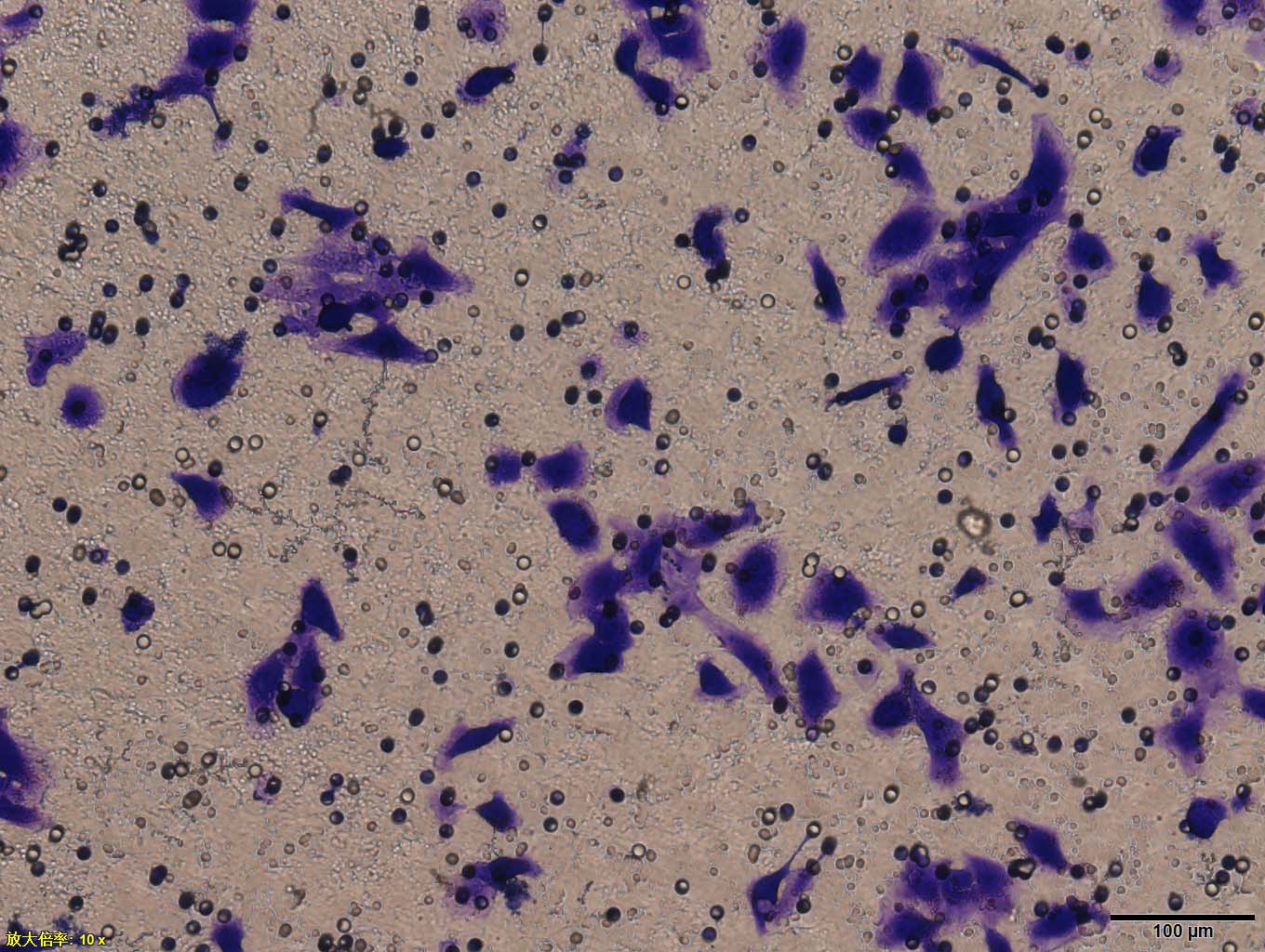

Supplement: S5 Fig — (ZIP) [file pone.0195844.s005.zip › S5_Fig5_File/S5_Fig5D_shVDR+TRPV5 í┴200 (1).jpg]

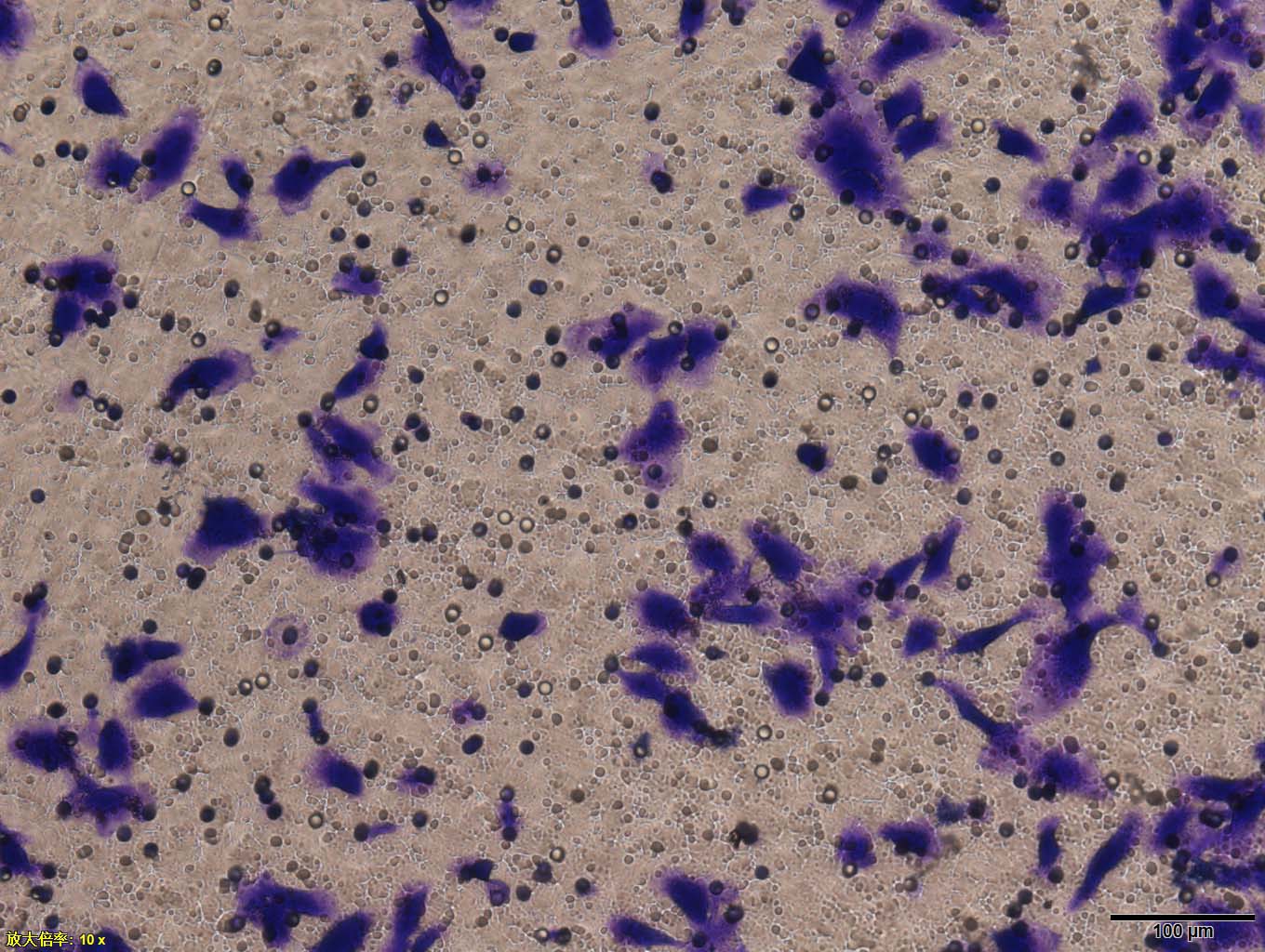

Supplement: S5 Fig — (ZIP) [file pone.0195844.s005.zip › S5_Fig5_File/S5_Fig5D_shVDR+TRPV5 í┴200 (2).jpg]

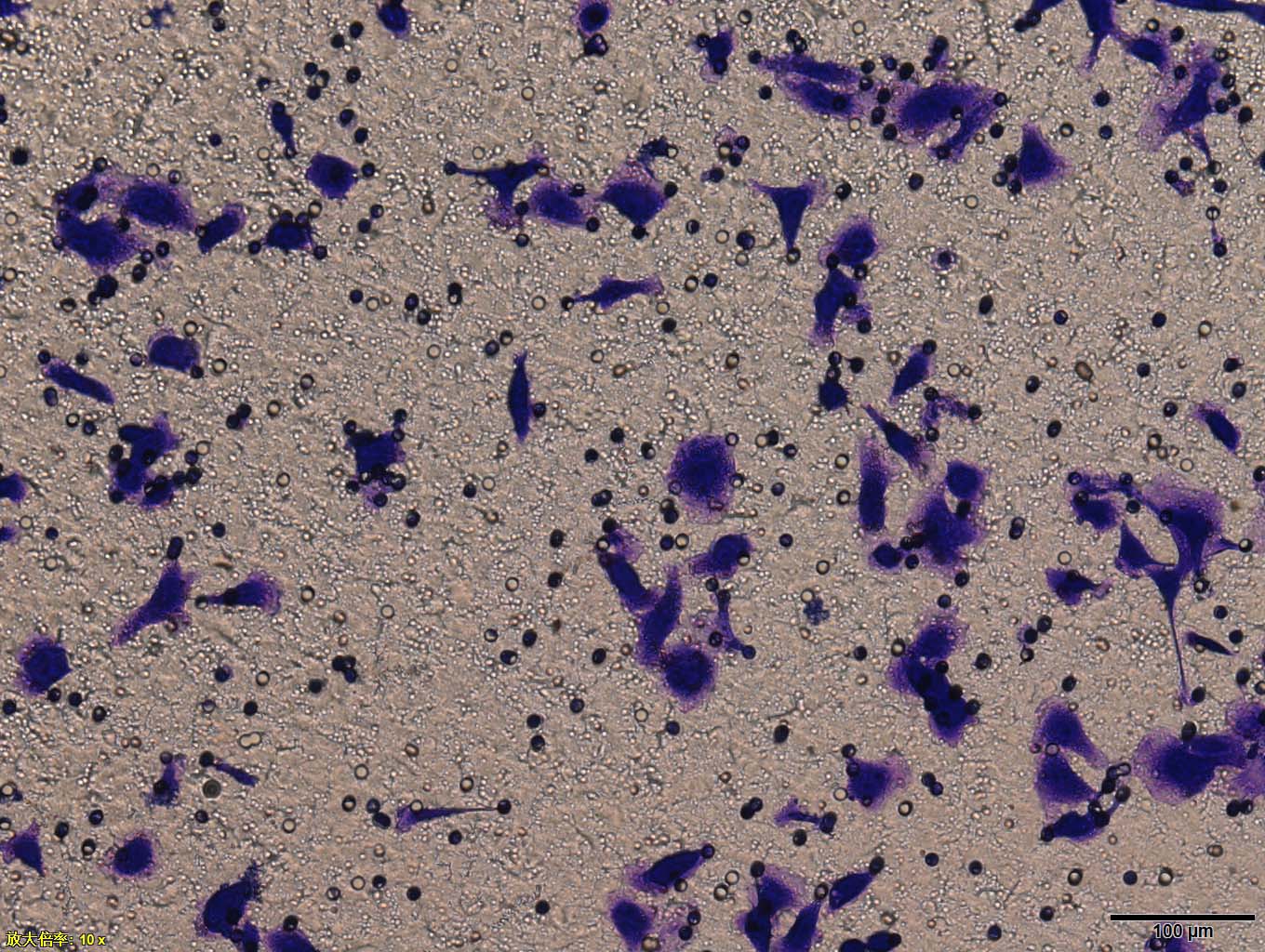

Supplement: S5 Fig — (ZIP) [file pone.0195844.s005.zip › S5_Fig5_File/S5_Fig5D_shVDR+TRPV5 í┴200 (3).jpg]

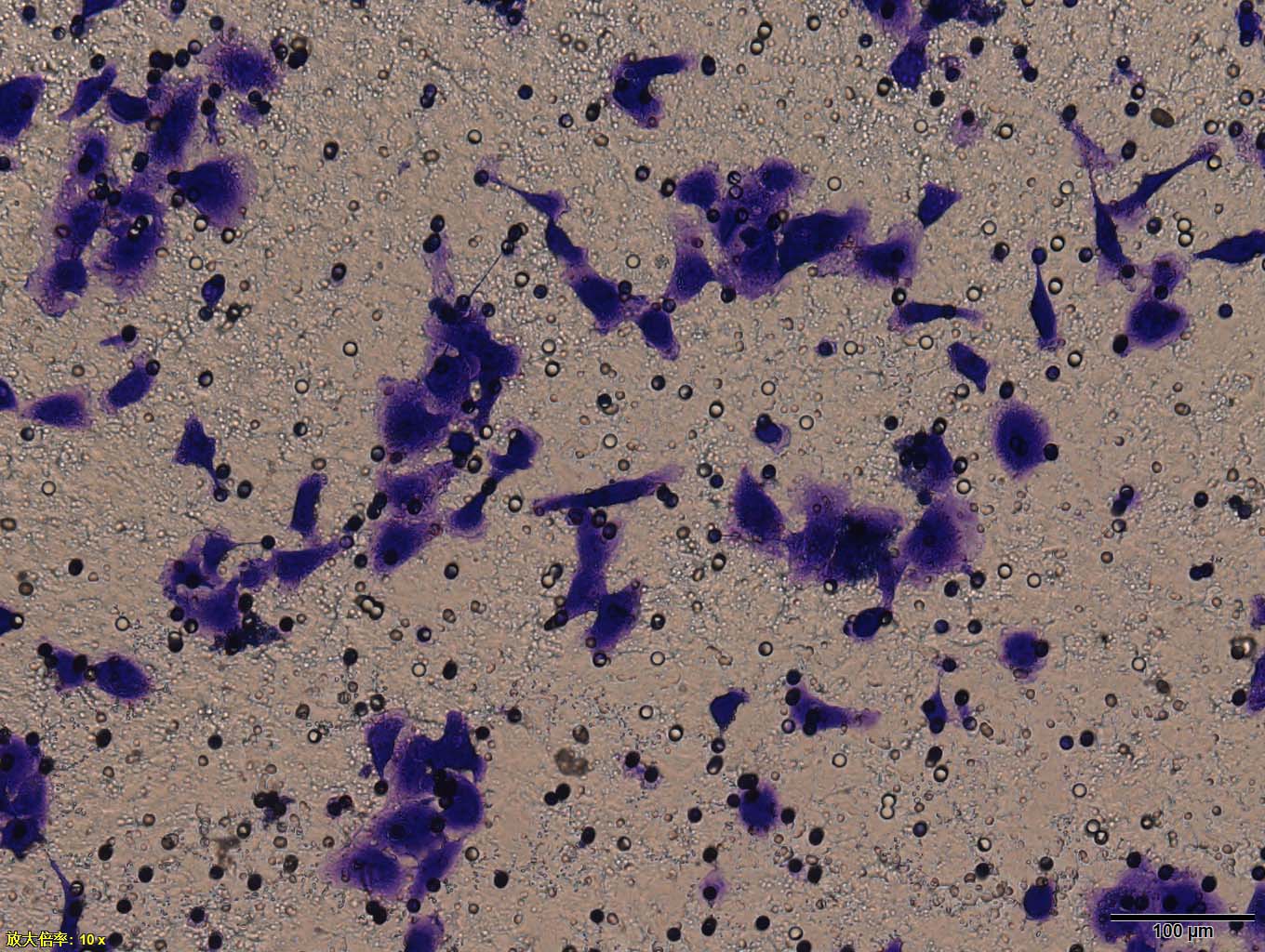

Supplement: S5 Fig — (ZIP) [file pone.0195844.s005.zip › S5_Fig5_File/S5_Fig5D_shVDR+TRPV5 í┴200 (4).jpg]

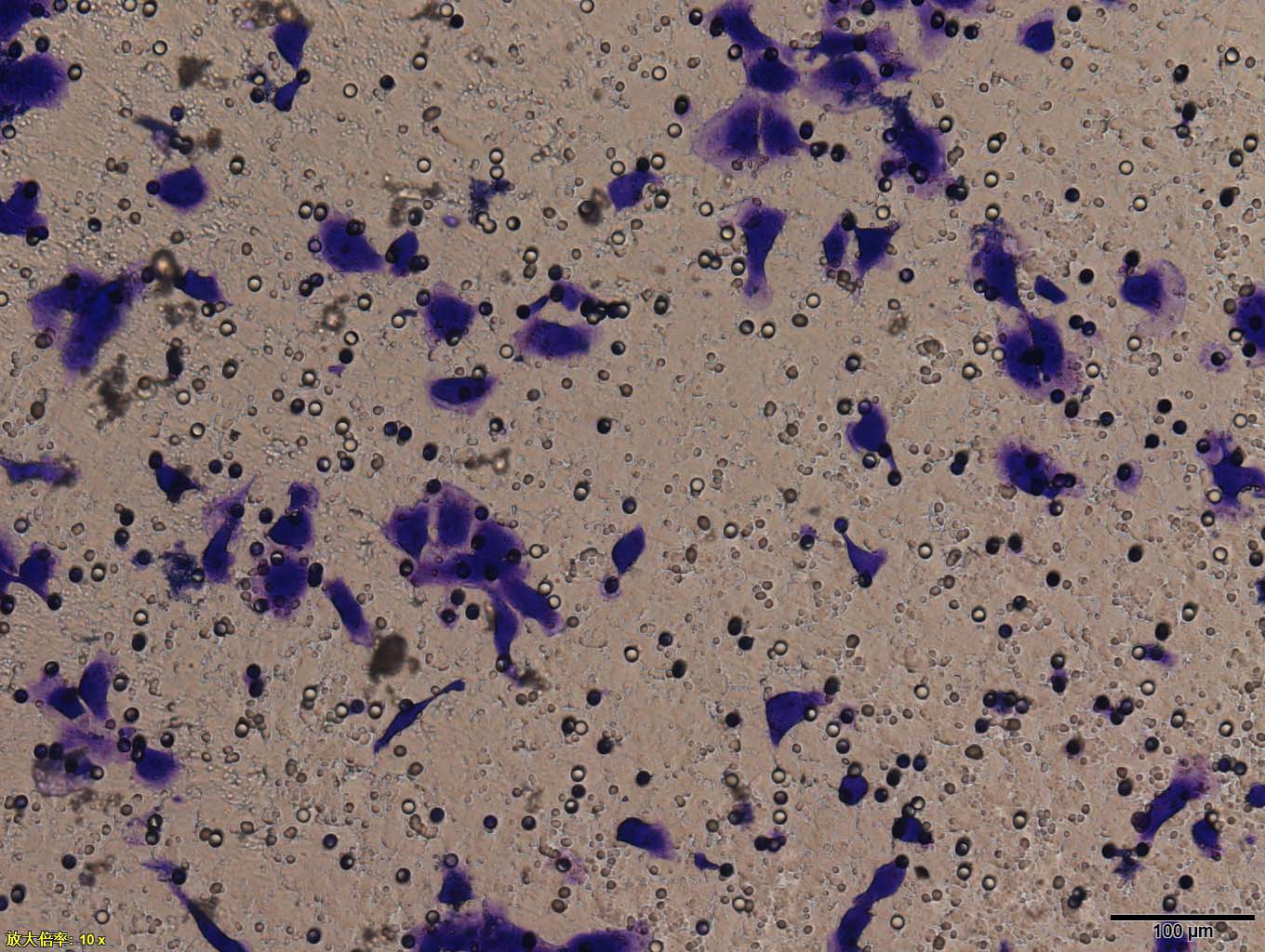

Supplement: S5 Fig — (ZIP) [file pone.0195844.s005.zip › S5_Fig5_File/S5_Fig5D_shVDR+TRPV5 í┴200 (5).jpg]

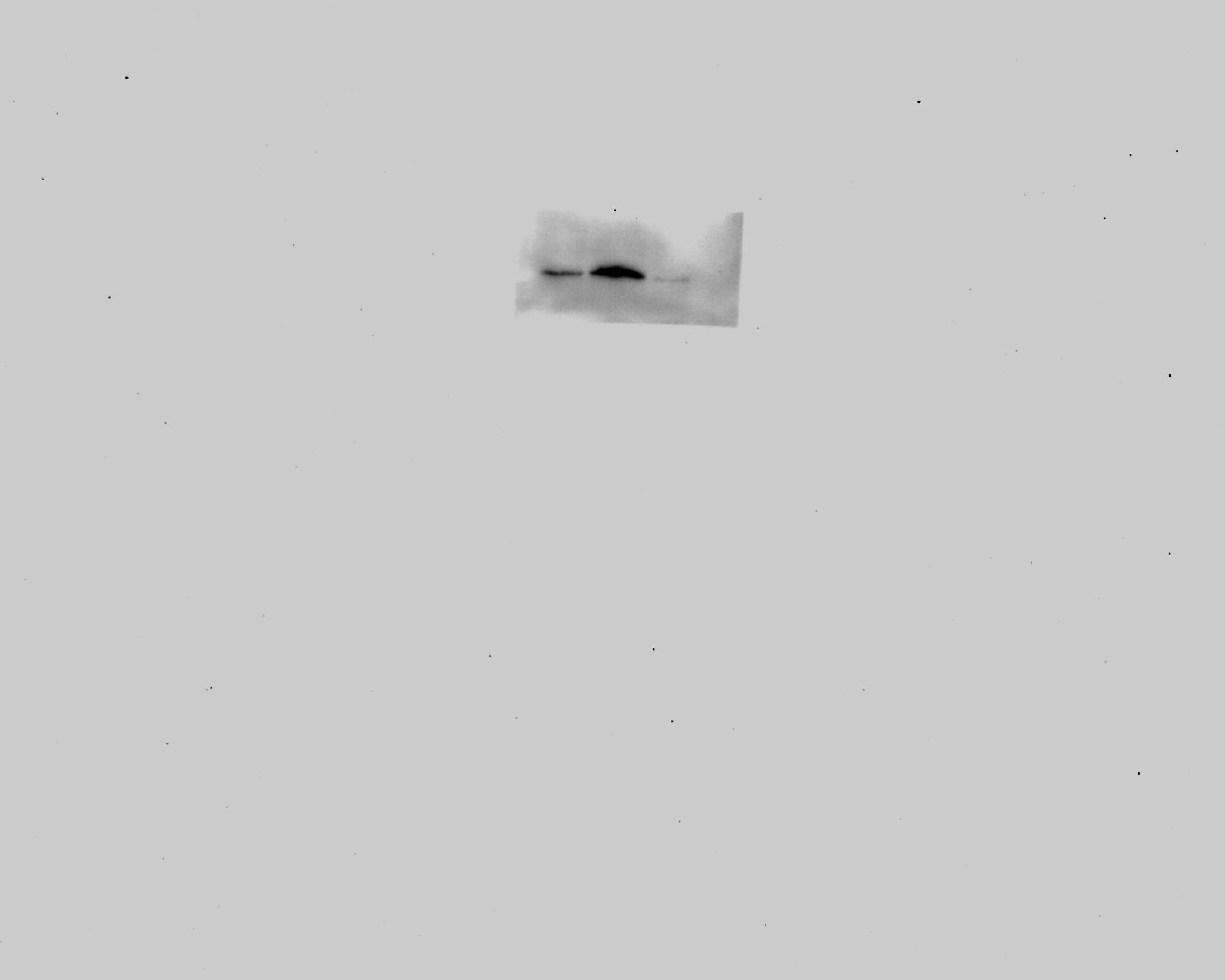

Supplement: S5 Fig — (ZIP) [file pone.0195844.s005.zip › S5_Fig5_File/S5_WB_TRPV5_shV+T.jpg]

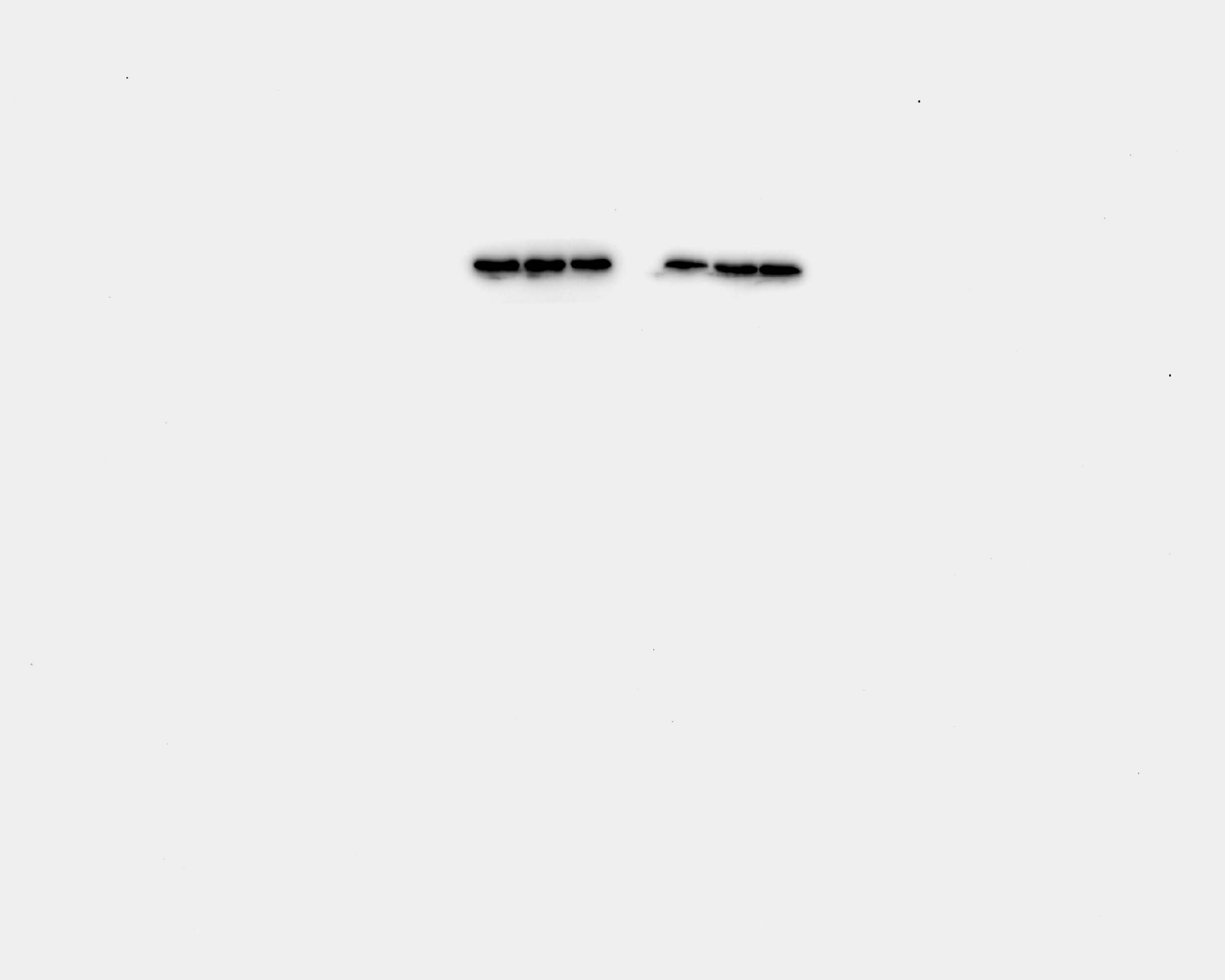

Supplement: S5 Fig — (ZIP) [file pone.0195844.s005.zip › S5_Fig5_File/S5_WB_a┬-actin_shV+T.jpg]

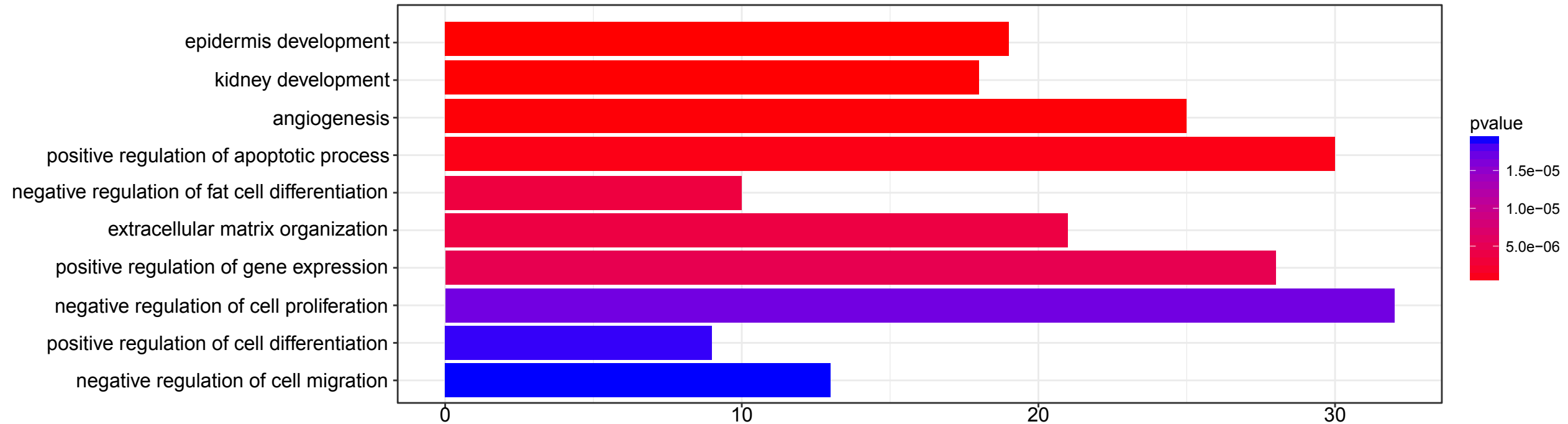

Supplement: S6 Fig — (ZIP) [file pone.0195844.s006.zip › S6_Fig6_File/caki-1leVDR.pvalue_0.05.allSigGene.BP.barplot.pdf]

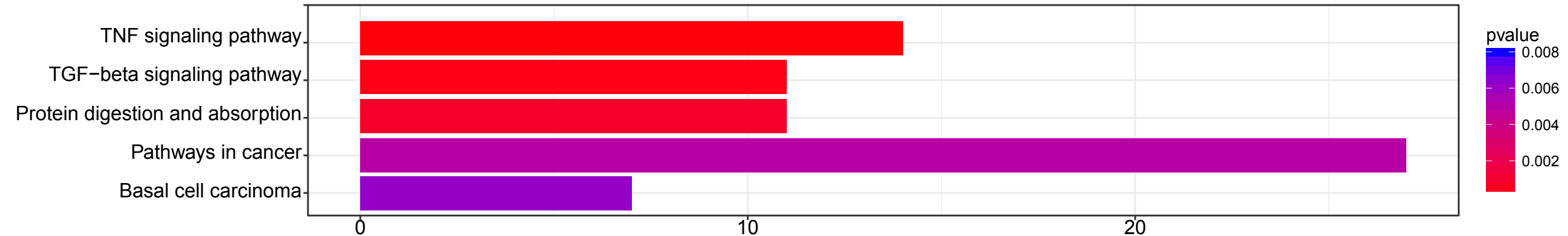

Supplement: S6 Fig — (ZIP) [file pone.0195844.s006.zip › S6_Fig6_File/caki-1leVDR.pvalue_0.05.allSigGene.KEGG.barplot.pdf]

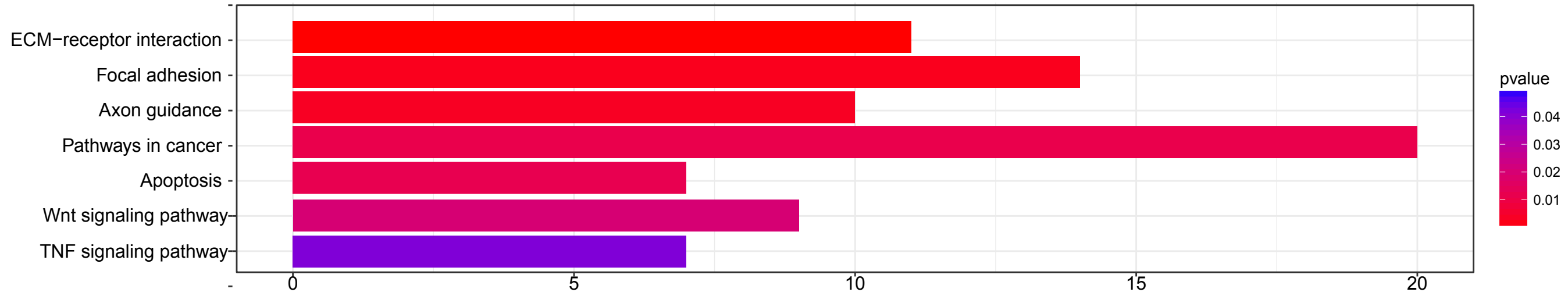

Supplement: S6 Fig — (ZIP) [file pone.0195844.s006.zip › S6_Fig6_File/Caki1shVDR.pvalue_0.05.allSigGene.KEGG.barplot.pdf]
